# The RNA helicase domain of NAT10 promotes the biogenesis of hypomodified ribosomes to enhance cancer cell proliferation

**DOI:** 10.1038/s41467-026-76383-w

**Published:** 2026-08-08

**Authors:** Mahmood H. Dalhat, Sharath Narayan, Maria Eleftheriou, James Russell, James Heslop, Hannah Serio, Khulood A. Alzahrani, Adam Suh, Ajay Edakkara, Sweta Raikundalia, Lea Landmann, Ryan Asby, Evangelia Stamou, Judith López, Yogeshkumar Malam, Demetrios Aspris, Emmely A. Patrasso, Stephanie Mou, George Giotopoulos, Walid T. Khaled, Brian J. P. Huntly, Fotios Sampaziotis, Konstantinos Tzelepis, Daniel Arango

**Affiliations:** 1 Department of Pharmacology, Feinberg School of Medicine, Northwestern University, Chicago, IL USA; 2 Robert H. Lurie Comprehensive Cancer Center, Feinberg School of Medicine, Northwestern University, Chicago, IL USA; 3 Driskill Graduate Program in Life Sciences, Feinberg School of Medicine, Northwestern University, Chicago, IL USA; 4 Cambridge Stem Cell Institute, University of Cambridge, Cambridge, UK; 5 Department of Haematology, University of Cambridge, Cambridge, UK; 6 Milner Therapeutics Institute, University of Cambridge, Puddicombe Way, Cambridge, UK; 7 MRC Toxicology Unit, University of Cambridge, Cambridge, UK; 8 Department of Pharmacology, University of Cambridge, Cambridge, UK

**Keywords:** RNA modification, Ribosome, Cancer, RNA

## Abstract

N-acetyltransferase 10 (NAT10) is a multifunctional enzyme that harbors RNA acetyltransferase and RNA helicase domains and has emerged as a therapeutic vulnerability in solid and hematological malignancies. By coupling Proteolysis Targeting Chimera-mediated degradation of NAT10 with a deep mutational scanning assay, followed by validations in biochemical assays, human cell lines, and female mouse xenografts, we find that the RNA helicase domain of NAT10 enhances cancer cell proliferation and tumor growth. This proliferative function of NAT10 is independent of RNA acetylation but requires its RNA-binding activity. The RNA helicase domain of NAT10 is required for 18S rRNA binding, promoting biogenesis of the 40S ribosomal subunit, while simultaneously interfering with the deposition of the conserved 18S rRNA modification m¹acp³Ψ. Loss of m¹acp³Ψ in 18S rRNA enhances cancer cell proliferation, revealing that NAT10 promotes the biogenesis of hypomodified ribosomes to facilitate tumor growth. These findings uncover a mechanism by which NAT10 promotes cancer cell proliferation and establish its RNA helicase domain as a potential therapeutic target.

## Abstract (web summary)

The mechanisms underlying the role of N-acetyltransferase 10 (NAT10) in cancer remain poorly characterised. Here, PROTAC-mediated degradation of NAT10 with a deep mutational scanning assay and functional validation reveal the oncogenic properties of the RNA helicase domain of NAT10.

## Introduction

The ribosome is a ribonucleoprotein complex responsible for protein synthesis^1^. Although once viewed as invariant machines, recent studies have demonstrated that ribosomes exhibit heterogeneity in both protein composition and ribosomal RNA (rRNA) modifications^2,3^, including the presence or absence of 1-methyl-3-(3-amino-3-carboxypropyl)-pseudouridine (m¹acp³Ψ)^4^. This highly conserved rRNA modification resides in the decoding center of the small ribosomal subunit (SSU, or 40S)^4–7^. Notably, loss of ribosomal m¹acp³Ψ serves as a pan-cancer biomarker^4^, and these hypomodified ribosomes preferentially translate proteins involved in ribosome biogenesis^4^, potentially establishing a positive feedback loop that supports increased ribosome production. However, the mechanisms underlying the loss of m¹acp³Ψ in cancer remain unknown.

N-acetyltransferase 10 (NAT10) is an RNA acetyltransferase enzyme implicated in several cancer-associated pathways, including cell proliferation, migration, and cellular stress responses^8^. NAT10 is not mutated in cancer^8^. Instead, its carcinogenic activity has consistently been associated with overexpression or mislocalization of the protein, which is observed across a range of tumor types, including hepatocellular carcinoma (HCC), acute myeloid leukemia (AML), pancreatic, gastric, and breast cancer, among others^8–18^. Clinically, NAT10 overexpression promotes tumor growth and metastasis and is associated with poor survival^8,10–18^, highlighting the therapeutic potential of targeting NAT10. Nevertheless, the mechanisms by which NAT10 promotes carcinogenesis remain poorly defined.

NAT10 contains a conserved GCN4-related N-terminal acetyltransferase (GNAT) domain that acetylates cytidine residues to form N4-acetylcytidine (ac⁴C), a modification found in 18S rRNA^19^, as well as in certain tRNAs^20^ and mRNAs^21^. While multiple studies suggest that NAT10 promotes carcinogenesis via RNA acetylation^12–17,22–29^, substantial inconsistencies persist. First, it remains unclear whether ac⁴C stoichiometry accumulates to physiologically significant levels in mRNAs^30,31^. Second, NAT10 requires co-adapters for RNA acetylation. The small nucleolar RNA (snoRNA) SNORD13 and the protein THUMPD1 serve as adapters to facilitate the acetylation of rRNA, tRNA, and mRNA^20,31–36^. Notably, the depletion of either SNORD13 or THUMPD1, and thus the loss of ac⁴C, does not impair cell proliferation or viability (Supplementary Fig. 1a)^20,33–36^. Third, although NAT10 is a multifunctional enzyme with a GNAT domain, an RNA helicase domain, a domain of unknown function (DUF1726), and a predicted tRNA binding domain (tRBD), previous studies have not systematically examined how its individual domains contribute to cancer. The first report introducing single-point mutations in NAT10, the widely used G641E mutant, suggested that loss of its acetyltransferase activity enhances cell proliferation and reduces cellular senescence^37^. Three subsequent studies in yeast indicated that the RNA helicase domain of NAT10, but not its acetyltransferase activity, was required for cell proliferation^20,32,38^. Many studies that followed those initial benchmarking reports used a single G641E mutation in the GNAT domain to conclude that the acetyltransferase activity promotes carcinogenesis^12,13,17,22–29,39–41^. However, a few studies, particularly those using different acetyltransferase mutants, such as H537A and R628A, have reached alternative conclusions, indicating that the acetyltransferase activity of NAT10 may affect cellular senescence or metastasis but not cell proliferation^16,18,20,37,42,43^.

In this study, we undertake an unbiased functional genomics approach using a proteolysis-targeting chimera (PROTAC) system and a deep mutational scanning assay to evaluate thousands of mutants across all domains of NAT10 and assess whether the proliferative mechanisms of NAT10 are cancer-type-specific or conserved across different biological contexts. Since NAT10 is functionally validated as a therapeutic vulnerability in HCC and AML^10–12,44–46^, these studies are performed in cellular and animal models of myeloid and liver cancers, representing solid and liquid malignancies, respectively. By integrating deep mutational scanning assays with cellular and animal models, we demonstrate that the RNA helicase domain of NAT10 is required for cell proliferation and tumor growth in both liver and myeloid cancers. Mechanistically, the RNA helicase domain of NAT10 is required for 18S rRNA binding, promoting biogenesis of the 40S ribosomal subunit, while concurrently interfering with m¹acp³Ψ deposition. Loss of m¹acp³Ψ, which is commonly observed in cancer^4^, increases cell proliferation, indicating that NAT10 promotes the production of hypomodified ribosomes to enhance cancer cell proliferation. In summary, this study reveals a mechanism by which NAT10 regulates ribosome biogenesis, m¹acp³Ψ deposition, and cancer cell proliferation, thereby providing a mechanistic basis for targeting the RNA helicase domain of NAT10 in cancer.

## Results

### Acute depletion of NAT10 with dTAG^V^−1 decreases cancer cell proliferation

**Fig. 1:**
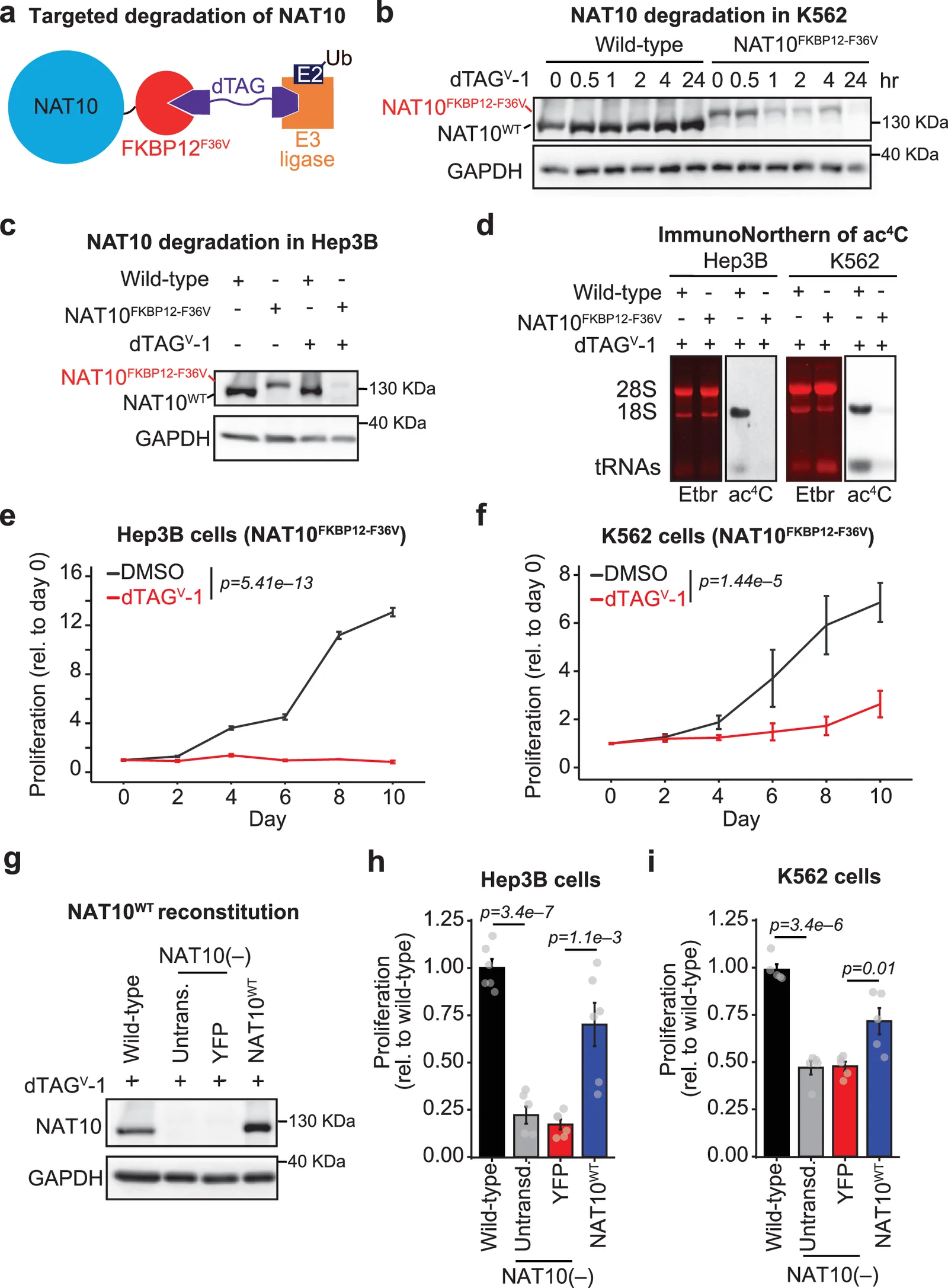
Inducible degradation of NAT10 decreases cancer cell proliferation. **a** Schematic of NAT10 degradation using the dTAG^V^-1 PROTAC system. **b** Western blot in wild-type and NAT10^FKBP12-F36V^ K562 cells treated with 100 nM dTAG^V^-1 at the indicated time points. NAT10^FKBP12-F36V^ migrates ~12 kDa above wild-type NAT10. Blots are representative of biological triplicates. **c** Western blot in wild-type and NAT10^FKBP12-F36V^ Hep3B cells treated with 100 nM dTAG^V^-1 for 24 h. Blots are representative of biological triplicates. **d** ImmunoNorthern blot of ac⁴C in total RNA from wild-type or NAT10^FKBP12-F36V^ cells treated with 100 nM dTAG^V^-1 for 72 h. This time frame allows for ac⁴C turnover after NAT10 degradation. Blots are representative of biological triplicates. **e**, **f** Cell proliferation over time in NAT10^FKBP12-F36V^ cells treated with 100 nM dTAG^V^-1. Data represent mean ± SEM. *n* = 3. Each data point represents an independent biological replicate. *p* values calculated by two-way ANOVA. **g** Western blot in NAT10^FKBP12-F36V^ Hep3B cells reconstituted with exogenous NAT10^WT^ or YFP [NAT10(–)]; wild-type or untransduced NAT10^FKBP12-F36V^ cells were used as controls. Blots are representative of biological triplicates. **h**, **i** Cell proliferation at day 8 post-dTAG^V^-1 treatment in NAT10^FKBP12-F36V^ cells reconstituted with exogenous NAT10^WT^ or YFP [NAT10(–)]. Wild-type or untransduced NAT10^FKBP12-F36V^ cells were used as controls. Each biological replicate corresponds to an independent transduction. Data represent mean ± SEM relative to the wild-type control. *n* = 6 for all groups in (**h**). *n* = 5 for all groups in (**i**). *p* values were calculated by two-tailed Student’s *t* test for the indicated comparisons. For (**b**–**i**), source data are provided as a Source Data file.

To examine the effect of acutely depleting NAT10 on cell proliferation, we developed a PROTAC system in cellular models of liver (Hep3B cells) and myeloid (K562 cells) cancers (Fig. 1a). Two distinct guide RNAs (gRNAs) targeting the 3′ untranslated region (3′UTR) of NAT10 and two homologous recombination vectors were used to introduce FKBP12^F36V^ and antibiotic resistance genes at the C terminus of endogenous NAT10 (Supplementary Fig. 1b, c). Successful integration of FKBP12^F36V^ into both alleles conferred resistance to hygromycin and G418 and resulted in an ~12 kDa increase in the molecular weight of NAT10 (Fig. 1b, c).

Treatment of NAT10^FKBP12-F36V^ cells with 100 nM dTAG^V^-1, which binds FKBP12^F36V^ and recruits an E3 ubiquitin ligase complex (Fig. 1a), induced NAT10 degradation within 24 h in NAT10^FKBP12-F36V^ cells, without affecting NAT10 levels in wild-type cells (Fig. 1b, c and Supplementary Fig. 1d). Furthermore, reduction of ac^4^C in total RNA confirmed the loss of NAT10 activity in NAT10^FKBP12-F36V^ cells treated with dTAG^V^-1 (Fig. 1d). Crucially, dTAG^V^-1 treatment decreased cell proliferation in NAT10^FKBP12-F36V^ cells (Fig. 1e, f), without affecting wild-type cell proliferation (Supplementary Fig. 1e, f). The proliferation defect in dTAG^V^-1-treated NAT10^FKBP12-F36V^ cells was rescued by re-expression of wild-type NAT10 (NAT10^WT^), but not by expression of a control protein (YFP, Fig. 1g–i). We noted that tagging endogenous NAT10 with FKBP12^F36V^ reduced protein levels, even in the absence of dTAG^V^-1 (Fig. 1b, c). This reduction likely resulted from altering the endogenous 3′UTR during FKBP12^F36V^ integration, leading to lower mRNA levels (Supplementary Fig. 1g). Nonetheless, NAT10^FKBP12-F36V^ cells remained viable and maintained proliferation in the absence of dTAG^V^-1 treatment (Supplementary Fig. 1h, i).

Together, these results demonstrate that targeted degradation of NAT10 is a robust tool for investigating the mechanisms by which NAT10 controls cell proliferation.

### Systematic interrogation of NAT10 functional domains by deep mutational scanning

NAT10 is a multifunctional enzyme containing a GNAT domain, an ATP-dependent RNA helicase domain, a predicted tRNA-binding domain (tRBD), a domain of unknown function (DUF1726), a coiled-coil (CC) motif, and two nuclear/nucleolar localization signals (Supplementary Fig. 2a). To systematically evaluate the role of each functional domain of NAT10 in cell proliferation, we performed site-saturation mutagenesis at 100 amino acid positions. Mutations were distributed across all domains of NAT10, with each residue substituted with all 20 possible amino acids or a premature stop codon. Residues were selected based on phylogenetic conservation (Supplementary Data 1), as conserved residues are more likely to be biologically relevant.

**Fig. 2:**
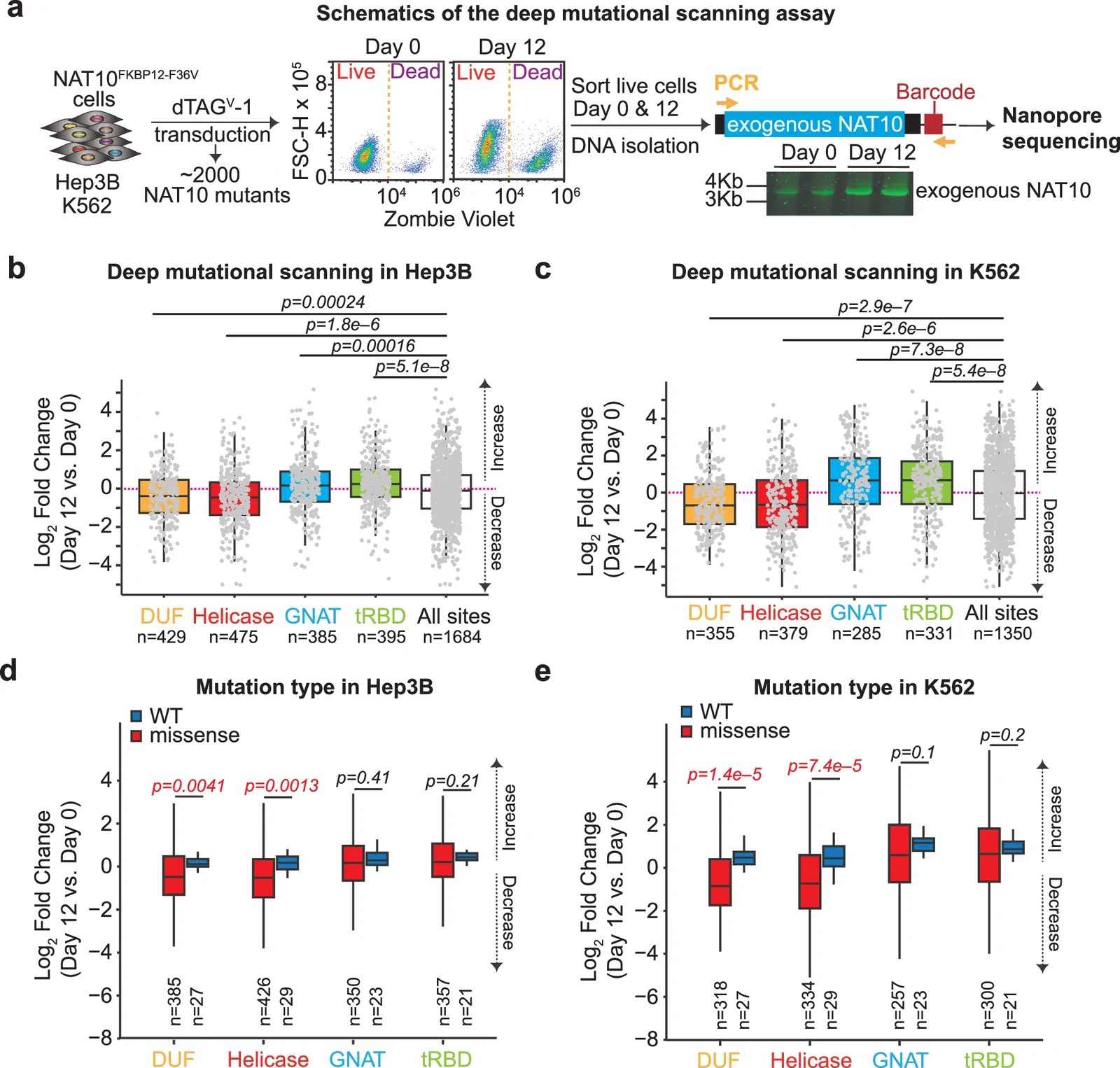
Functional profiling of thousands of NAT10 mutants. **a** Schematic of the deep mutational scanning workflow. NAT10^FKBP12-F36V^ cells were transduced with the site-saturation mutagenesis library. Two days after transduction, cells were treated with 100 nM dTAG^V^-1 and cultured for 12 days. Viable cells were sorted at days 0 and 12 post-treatment. Exogenous NAT10 was PCR-amplified and sequenced using Nanopore. Three biological replicates corresponding to independent transductions were performed. GNAT refers to the N-acetyltransferase domain. DUF refers to the domain of unknown function. tRBD refers to the predicted tRNA binding domain. **b**, **c** Box plots showing log₂(fold change) in mutant abundance (day 12 vs. day 0) for individual NAT10 variants, grouped by protein domain. Values < 0 indicate reduced cell fitness. Box plots show the median, the interquartile range (25th–75th percentile, the box), and whiskers extending to 1.5 times the interquartile range. *p* values were calculated by pairwise Wilcoxon rank-sum tests (two-sided), adjusted using the Benjamini-Hochberg method. *n* denotes the number of unique variants per domain. The data is derived from three biological replicates. **d**, **e** Box plots showing the log₂(fold change) for wild-type (WT) and missense mutations within each domain. Box plots show the median, the interquartile range (25th–75th percentile, the box), and whiskers extending to 1.5 times the interquartile range. *p* values were calculated by the Wilcoxon rank-sum test (two-sided) comparing WT and missense variants. *n* indicates the number of unique variants per group. The data is derived from three biological replicates. For (**b**–**e**), source data are provided in the Source Data file and Supplementary Data 3.

The site-saturation mutagenesis library was cloned into lentiviral expression vectors, yielding over 1900 unique mutants (Supplementary Fig. 2b, c). This library was transduced into Hep3B and K562 cells expressing NAT10^FKBP12-F36V^. Two days post-transduction, endogenous NAT10 was depleted with dTAG^V^-1, followed by fluorescence-activated cell sorting (FACS) of viable cells at both 0 and 12 days post-treatment (Fig. 2a). DNA was isolated from each sample, exogenous NAT10 was PCR amplified, and amplicons were sequenced using Nanopore (Fig. 2a). Each residue in the library is uniquely barcoded (Supplementary Fig. 2b and Supplementary Data 2), enabling identification of every mutant through its barcode and corresponding mutation.

In deep mutational dropout assays, cell proliferation is inferred from changes in mutant frequency over time^47^. Since cells carrying deleterious variants divide more slowly or fail to survive relative to other mutants in the population, reductions in frequency (dropouts) can be interpreted as impaired proliferative capacity^47^. To identify NAT10 mutations conferring a proliferative disadvantage, sequencing reads were binned into 100 distinct barcodes, each corresponding to a targeted residue in NAT10. To identify specific variants at each site, reads were aligned to the reference NAT10 sequence. For each read, the amino acid substitution was extracted, and the abundance of each mutant was counted at days 0 and 12 (Supplementary Data 3). A negative binomial statistical model was performed to identify significant changes in mutant frequency (Supplementary Data 3). When grouped by NAT10 domain, we observed that the representation of mutants in the RNA helicase and DUF1726 domains was significantly reduced relative to the GNAT and tRBD domains (Fig. 2b, c).

To account for potential confounding effects of premature stop codons, which may truncate protein domains and disproportionately disrupt function, we separated nonsense mutations from missense variants. Notably, every position in the library included wild-type sequences. Thus, we compared the effects of nonsense and missense mutations to the corresponding wild-type amino acids. Clones carrying premature stop codons dropped out across all domains (Supplementary Fig. 2d, e). In contrast, the representation of missense mutations was only significantly reduced in the DUF1726 and RNA helicase domains compared to wild-type clones (Fig. 2d, e), whereas missense mutations in the GNAT or tRBD domains had no significant effect (Fig. 2d, e). Together, these findings suggest that the RNA helicase and DUF1726 domains of NAT10 are relevant for cell proliferation in Hep3B and K562 cells.

### The RNA helicase domain of NAT10 is required for cell proliferation

**Fig. 3:**
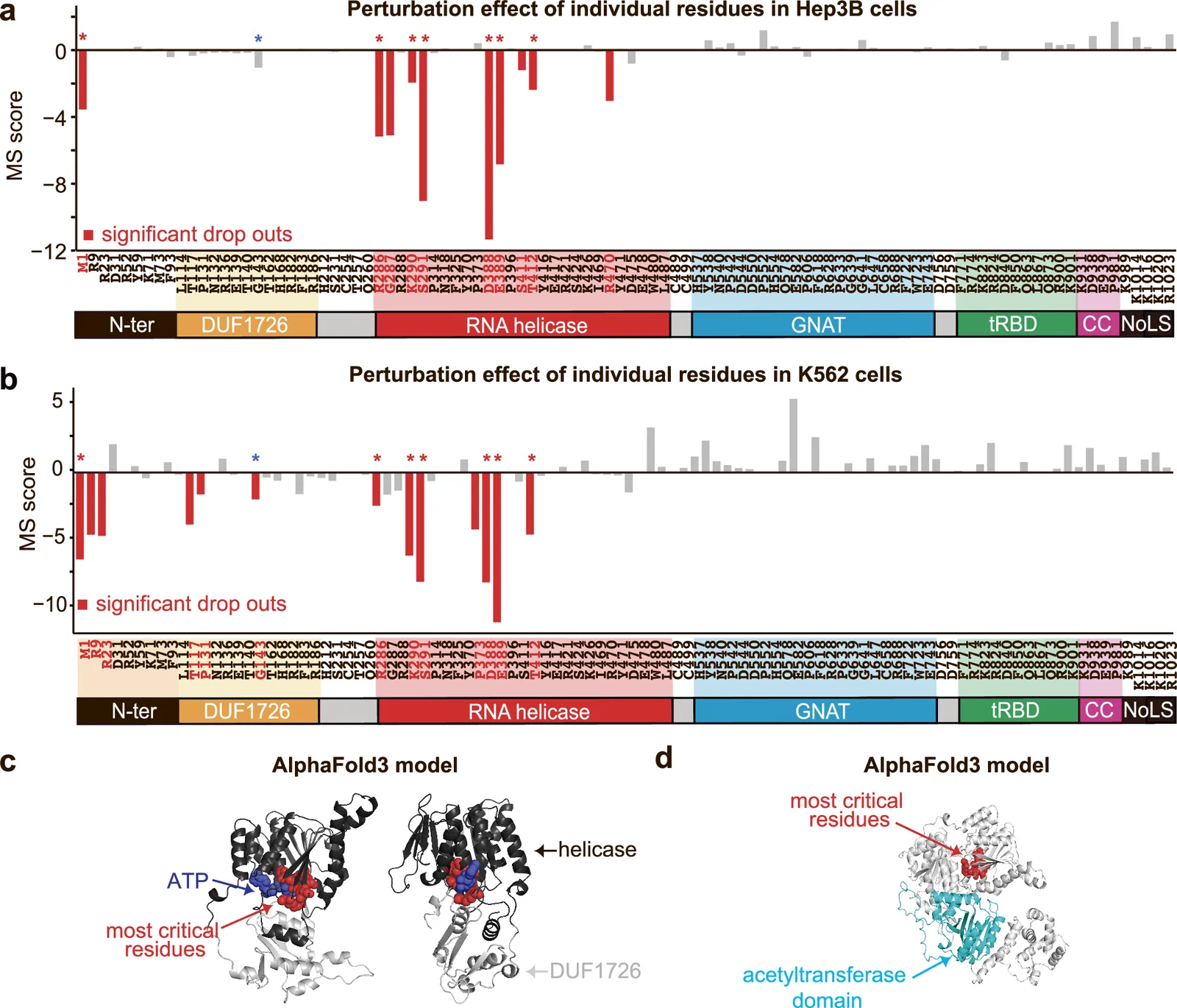
The RNA helicase domain of NAT10 is essential for cell proliferation. **a**, **b** Mutational scanning (MS) scores for each residue across all NAT10 domains in Hep3B (**a**) and K562 (**b**) cells. Red bars indicate residues essential for cell proliferation; red asterisks denote residues essential in both cell lines. Blue asterisk denotes residue G143. GNAT refers to the N-acetyltransferase domain. DUF refers to the domain of unknown function. The data is derived from three biological replicates. Source data are provided as a Source Data file and Supplementary Data 4. **c** AlphaFold3 model showing the DUF1726 domain (gray) and RNA helicase domain (black) of NAT10 in complex with ATP (blue). Red spheres highlight residues essential for proliferation. **d** AlphaFold3 model of NAT10 (excluding the CC and NoLS regions), with the GNAT domain depicted in cyan. Red spheres indicate residues essential for proliferation.

To pinpoint amino acid residues essential for NAT10 function, we calculated a mutational scanning (MS) score that integrates both the fold change in mutant abundance and the adjusted p-value for each residue (Supplementary Data 4). Seven candidate residues were reproducibly identified as critical for cell proliferation in both Hep3B and K562 cell lines: M1, R286, K290, S291, D388, E389, and T412 (Fig. 3a, b indicated with red *). As M1 encodes the start codon, its disruption leads to loss of full-length protein. In contrast, the remaining six residues are all located within the RNA helicase domain (Fig. 3a, b). Structural modeling revealed that R286, K290, S291, D388, E389, and T412 cluster within the ATP-binding pocket of the RNA helicase domain (Fig. 3c and Supplementary Movie 1). Notably, no residue in the GNAT domain significantly impacted proliferation, and all key helicase residues were structurally distant from the acetyltransferase domain (Fig. 3d).

To validate these findings, we introduced individual point mutations into the RNA helicase domain of NAT10 (Supplementary Fig. 3a). We focused on E389 and K290, which showed strong phenotypes in the deep mutational assay and displayed general intolerance to substitutions, except for chemically similar changes such as K290R (Supplementary Fig. 3b, c). The E389 residue is part of the DEAA (Walker B) motif that couples ATP hydrolysis with RNA binding and helicase function^48^. The K290 residue corresponds to a highly conserved lysine in the Walker A motif required for ATP binding and hydrolysis^20,48,49^. Structural prediction with AlphaFold3 indicated that K290 interacts with ATP, and substitutions such as K290V, K290A, or E389L alter this interaction (Supplementary Fig. 3d–g).

We also generated individual point mutations in the DUF1726 and GNAT domains. For the GNAT domain, we selected substitutions known to abrogate acetyltransferase activity: H537A, R628A, and G641E^16,20,37,43^. Although these residues exhibited mixed effects, with some individual variants increasing and others decreasing proliferation (Supplementary Fig. 4a, b), the overall effects were not statistically significant (Fig. 3a, b and Supplementary Data 4). For the DUF1726, we selected L114E and G143H. L114 is the closest residue to the ATP-binding site and positions K290 and E389 (Supplementary Fig. 4c, d). G143 exhibited a significant decrease in proliferative function in K562 cells and the largest reduction among DUF1726 residues in Hep3B, albeit not significantly (Fig. 3a, b, indicated with blue *). As with GNAT mutations, individual assessment of residues L114 and G143 revealed mixed effects, with some variants increasing and others decreasing proliferation (Supplementary Fig. 4e, f).

**Fig. 4:**
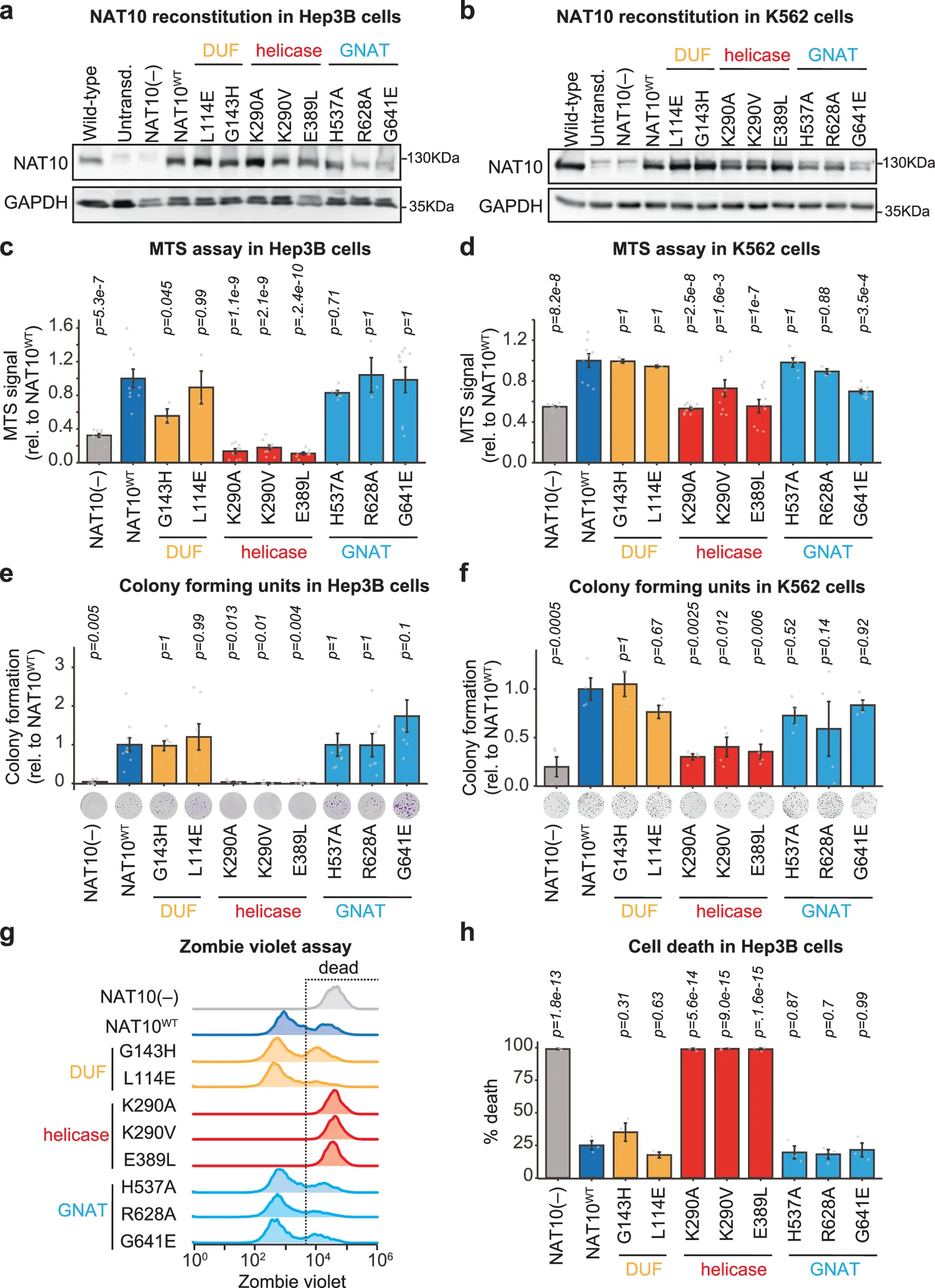
The RNA helicase domain of NAT10 is required for cell proliferation. **a**, **b** Western blot in NAT10^FKBP12-F36V^ Hep3B (**a**) and K562 (**b**) cells reconstituted with exogenous NAT10^WT^ or the indicated mutants. The wild-type and untransd. conditions refer to parental and NAT10^FKBP12-F36V^ cells, respectively, with no transduction or clone reconstitution. NAT10(–) refers to NAT10^FKBP12-F36V^ cells reconstituted with the non-oncogenic protein YFP. All other lanes refer to NAT10^FKBP12-F36V^ cells reconstituted with NAT10^WT^, NAT10^L114E^, NAT10^G143H^, NAT10^K290A^, NAT10^K290V^, NAT10^E389L^, NAT10^H537A^, NAT10^R628A^, or NAT10^G641E^. All cells were treated with 100 nM dTAG^V^-1. Cells were lysed five days after transduction and 24 h after dTAG^V^-1 treatment. GNAT refers to the N-acetyltransferase domain. DUF refers to the domain of unknown function. Blots were performed on four independent biological replicates. **c**, **d** Cell proliferation was measured ten days after dTAG^V^-1 treatment in NAT10^FKBP12-F36V^ Hep3B (**c**) and K562 (**d**) cells expressing exogenous NAT10^WT^ or the indicated NAT10 mutants. Data represent mean ± SEM. *n* = 9 for NAT10(-), NAT10^WT^, K290A, K290V, E389L, and G641E; *n* = 6 for H537A; *n* = 3 for G143H, L114E, and R628A. Each replicate represents an independent transduction. *p* values were calculated by one-way ANOVA with Dunnett’s post-hoc test relative to NAT10^WT^. **e** Colony formation assays in NAT10^FKBP12-F36V^ Hep3B cells expressing exogenous NAT10^WT^ or the indicated NAT10 mutants. Cultures were fixed and stained 10 days after dTAG^V^−1 treatment. Representative images are included below the bar plots. Data represent mean ± SEM. *n* = 10 for NAT10(-), NAT10^WT^, E389L; *n* = 9 for H537A; *n* = 7 for G143H, L114E, K290A, K290V, R628A, and G641E. Each replicate represents an independent transduction. *p* values were calculated by one-way ANOVA with Dunnett’s post-hoc test relative to NAT10^WT^. **f** Colony formation assays in NAT10^FKBP12-F36V^ K562 cells expressing exogenous NAT10^WT^ or the indicated NAT10 mutants. Cultures were fixed and stained 10 days after dTAG^V^-1 treatment. Representative images are included below the bar plots. Data represent mean ± SEM. *n* = 4 in all conditions. Each replicate represents an independent transduction. *p* values were calculated by one-way ANOVA with Dunnett’s post-hoc test relative to NAT10^WT^. **g**, **h** Cell viability was measured via flow cytometry using the Zombie violet assay in NAT10^FKBP12-F36V^ Hep3B expressing exogenous NAT10^WT^ or the indicated NAT10 mutants 10 days after dTAG^V^-1 treatment. Data represent mean ± SEM. *n* = 3. Each replicate represents an independent transduction. *p* values were calculated by one-way ANOVA with Dunnett’s post-hoc test relative to NAT10^WT^. For **a**–**h,** source data are provided as a Source Data file.

Western blotting confirmed expression of all NAT10 variants in both NAT10^FKBP12-F36V^ K562 and Hep3B cells (Fig. 4a, b), with some GNAT mutants exhibiting reduced protein expression compared to NAT10^WT^ (Fig. 4a, b). To evaluate the effects on cell proliferation, we transduced NAT10^FKBP12-F36V^ cells with each mutant, treated them with dTAG^V^-1, and quantified cell proliferation via colony formation assays, metabolic activity via MTS assays, and cell viability using Zombie violet staining (Fig. 4c–h). Reconstitution with NAT10^WT^ consistently increased proliferation and viability compared to NAT10(–) cells (Fig. 4c–h; blue bars). Remarkably, all RNA helicase mutants showed a significant and consistent impairment in proliferation across all orthogonal assays (Fig. 4c–h and Supplementary Fig. 5a, b; red bars). The severity of these RNA helicase mutants was most evident in colony formation assays (Fig. 4e), Zombie violet assays (Fig. 4g, h and Supplementary Fig. 5e) and light microscopy images (Supplementary Fig. 5c) in Hep3B cells. In K562 cells, the RNA helicase mutants led to a significant reduction in cell proliferation (Fig. 4d, f), but not in cell viability (Supplementary Fig. 5d). Together, these observations demonstrate that the RNA helicase domain of NAT10 is required for cell proliferation.

Conversely, mutations in the GNAT and DUF1726 domains produced diverse results, which were generally less significant, and the magnitude of the effect was less biologically relevant compared to the RNA helicase mutants. For example, the GNAT mutant H537A caused a minor but significant reduction in metabolic activity when the MTS assay was assessed 8 days after transduction in both Hep3B and K562 cells (Supplementary Fig. 5a, b), but not at 10 days after transduction (Fig. 4c, d). However, the magnitude and the significance of the H537A effect at day 8 were much smaller than in the RNA helicase mutants. No significant differences for H537A were observed in colony formation or Zombie violet assays (Fig. 4e–h; and Supplementary Fig. 5c, d). Furthermore, the GNAT mutant G641E and the DUF1726 mutant G143H caused a minor yet significant decrease in metabolic activity in only one cell line, but not the other, when the MTS assay was performed 10 days after transduction (Fig. 4c, d). No significant differences for G641E or G143H were observed in colony formation or Zombie violet assays (Fig. 4e–h; Supplementary Fig. 5c, d). These findings suggest that disruption of the GNAT and DUF1726 domains of NAT10 may cause mutant-, cell type-, and method-specific effects on cell proliferation, in stark contrast to the RNA helicase mutants, which exhibited severe defects in cell proliferation across all orthogonal assays.

We noted that NAT10 depletion or expression of RNA helicase mutants was more severe in Hep3B cells than in K562 cells. To evaluate an additional hepatocellular carcinoma cell line, we generated the NAT10^FKBP12-F36V^ PROTAC system in SNU-449 cells. Similar to the results in Hep3B, expression of RNA helicase mutants in SNU-449 cells resulted in a significant and severe impairment of colony formation (Supplementary Fig. 5f). In contrast, mutations in the GNAT or DUF1726 domains had no effect (Supplementary Fig. 5f).

Next, we evaluated whether overexpression of NAT10^WT^ is sufficient to increase cell proliferation. To test this, we expressed both NAT10^WT^ and RNA helicase mutants in wild-type Hep3B cells, which express normal (untagged) endogenous NAT10. Expression of NAT10^WT^ was sufficient to increase cell proliferation compared to the non-oncogenic protein YFP in Hep3B cells (Supplementary Fig. 5g). In contrast, expression of the RNA helicase mutants led to a significant decrease in cell proliferation compared to YFP control (Supplementary Fig. 5g). We applied this approach to investigate the effect of the RNA helicase mutant in an additional AML cell line, given that myeloid cells were generally refractory to FKBP^F36V^ tagging. Accordingly, we transduced OCI-AML3 cells with YFP, NAT10^WT^, or NAT10^E389L^ expression vectors and assessed cell proliferation. While NAT10^WT^ had a modest effect relative to the YFP control (Supplementary Fig. 5h, i), expression of the RNA helicase mutant NAT10^E389L^ significantly decreased cell proliferation, with a corresponding increase in the differentiation marker CD11b (Supplementary Fig. 5h–j).

Together, these findings indicate that the RNA helicase domain of NAT10 is required for cell proliferation, and that mutations in this domain can act as dominant-negative variants in cells with endogenous expression of wild-type NAT10.

### The RNA helicase domain of NAT10 is required for tumor growth

**Fig. 5:**
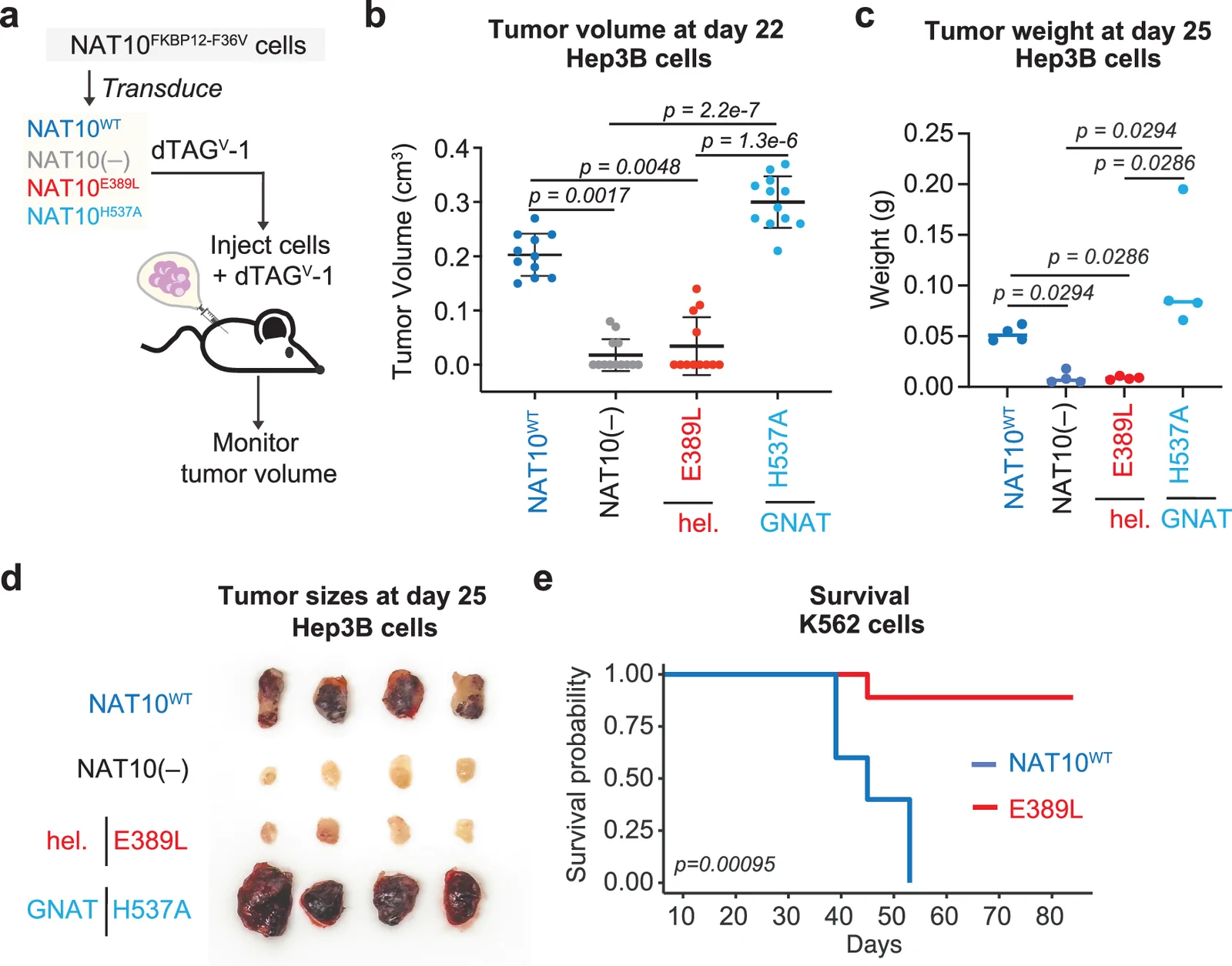
The RNA helicase domain of NAT10 is required for tumor growth. **a** Schematic of in vivo xenotransplantation using NAT10^FKBP12-F36V^ Hep3B cells reconstituted with exogenous NAT10^WT^, NAT10^E389L^ (helicase mutant), NAT10^H537A^ (GNAT mutant), or YFP [NAT10(–)]. **b** Tumor volumes at day 22 post-transplantation from Hep3B xenograft experiments. Data were compared using the Kruskal–Wallis test with Dunn’s multiple comparisons with Bonferroni correction (NAT10^WT^, *n* =  11; NAT10^E389L^, *n* =  13; NAT10^H537A^, *n* =  12; NAT10(–), *n* =  12). Data are shown as individual values with mean ± SD. **c** Tumor weights at day 25 for four representative mice per group from Hep3B xenograft experiments. Data were compared using pairwise two-sided Mann–Whitney U tests. Data are presented as individual values with the median. **d** Representative images of Hep3B tumors harvested from the animals shown in (**c**). **e** Kaplan-Meier survival probability over time for NAT10^FKBP12-F36V^ K562 xenografts expressing exogenous NAT10^WT^ (*n* = 5) or NAT10^E389L^ (*n* = 9). *p* values were calculated using the Log-rank test. For **b**, **c** and **e** source data are provided as a Source Data file.

To assess the role of the NAT10 RNA helicase domain in tumor growth in vivo, we performed xenograft studies using NAT10^FKBP12-F36V^ Hep3B cells, which showed a more consistent phenotype in cellular assays. Cells expressing NAT10^WT^, NAT10^E389L^, NAT10^H537A^, or NAT10(–) were injected subcutaneously into the flanks of immunodeficient NSG mice (Fig. 5a). To ensure efficient depletion of endogenous NAT10 in vivo, dTAG^V^-1 was administered at the time of xenotransplantation (Fig. 5a). Tumor volume and body weight were monitored at serial time points following engraftment. Consistent with the in vitro data, NAT10^WT^ supported robust tumor formation, whereas NAT10(–) and the NAT10^E389L^ RNA helicase mutant failed to initiate tumor growth (Fig. 5b–d; and Supplementary Fig. 6a, b). To determine whether inhibition of the RNA helicase domain of NAT10 impacts the overall survival using an additional cancer model, we injected NAT10^FKBP12-F36V^ K562 cells expressing NAT10^WT^ or the RNA helicase mutant NAT10^E389L^ via the tail vein in NSG mice and monitored animal survival over time. Results indicate that animals injected with NAT10^WT^ cells succumb to disease within 53 days post-transplantation (Fig. 5e), whereas 90% of NAT10^E389L^ animals remained healthy throughout the experiment. Together, these in vivo data confirm that the RNA helicase domain of NAT10 is essential for tumor growth in vivo, and its disruption prolongs survival.

Although disruption of the NAT10 helicase domain abolished tumor growth and prolonged survival in vivo, the therapeutic potential of NAT10 inhibition depends on its effects in non-malignant cells. To address this, normal CD34⁺ hematopoietic stem and progenitor cells (HSPCs) were isolated from human cord blood and transduced with two shRNAs targeting NAT10 or a vector control. Colony formation assays showed that NAT10 knockdown did not impair the clonogenic capacity of normal human HSPCs (Supplementary Fig. 7a). Next, we performed a comparative analysis in murine HSPCs isolated from isogenic Flt3^Itd/+^/Rosa26^cas9/+^ (normal phenotype)^50^, and NPM1^flox-cA/+^/Flt3^Itd/+^/Rosa26^cas9/+^ (malignant phenotype) mice. The Flt3^Itd/+^/Rosa26^cas9/+^ cells were further transduced with MLL-AF9 to generate malignant MLL-AF9 /Flt3^Itd/+^/Rosa26^cas9/+^ cells. Both NPM1^flox-cA/+^ and MLL-AF9 act as oncogenic drivers in the Flt3^Itd/+^ background. Since all the above isogenic models express Cas9 from the Rosa26 locus, we transduced them ex vivo with two gRNAs targeting murine Nat10 or with an empty vector control (Supplementary Fig. 7b). These vectors co-express Blue Fluorescent Protein (BFP). In this manner, proliferation capacity can be inferred from how the proportion of transduced (BFP + ) versus untransduced (BFP-) cells changes over time. Remarkably, we found that NAT10 depletion had negligible effects on the proliferation capacity of non-leukemic HSPCs (Supplementary Fig. 7c). In contrast, CRISPR/Cas9 ablation of NAT10 significantly and consistently suppressed both primary murine AML models (Supplementary Fig. 7d). We next performed colony formation assays in the same murine populations. The results were consistent: CRISPR/Cas9-mediated ablation of NAT10 specifically decreased clonogenic capacity in highly proliferative leukemic cells but not in non-leukemic normal-like HSPCs cells (Supplementary Fig. 7e, f).

To include a normal liver model, we used Intrahepatic Cholangiocyte Organoids (ICOs) derived from surgical resections of healthy livers. Organoids were transduced with two different shRNAs against NAT10 or a shRNA control. Notably, NAT10 knockdown had no effect on organoid growth or viability (Supplementary Fig. 8a–c), indicating that NAT10 knockdown is not toxic in normal liver cells.

Collectively, these findings indicate that targeting NAT10 significantly impairs proliferation in malignant cells but not in normal cells with low proliferative capacity.

### The RNA helicase domain of NAT10 is required for 18S rRNA binding and 40S biogenesis

**Fig. 6:**
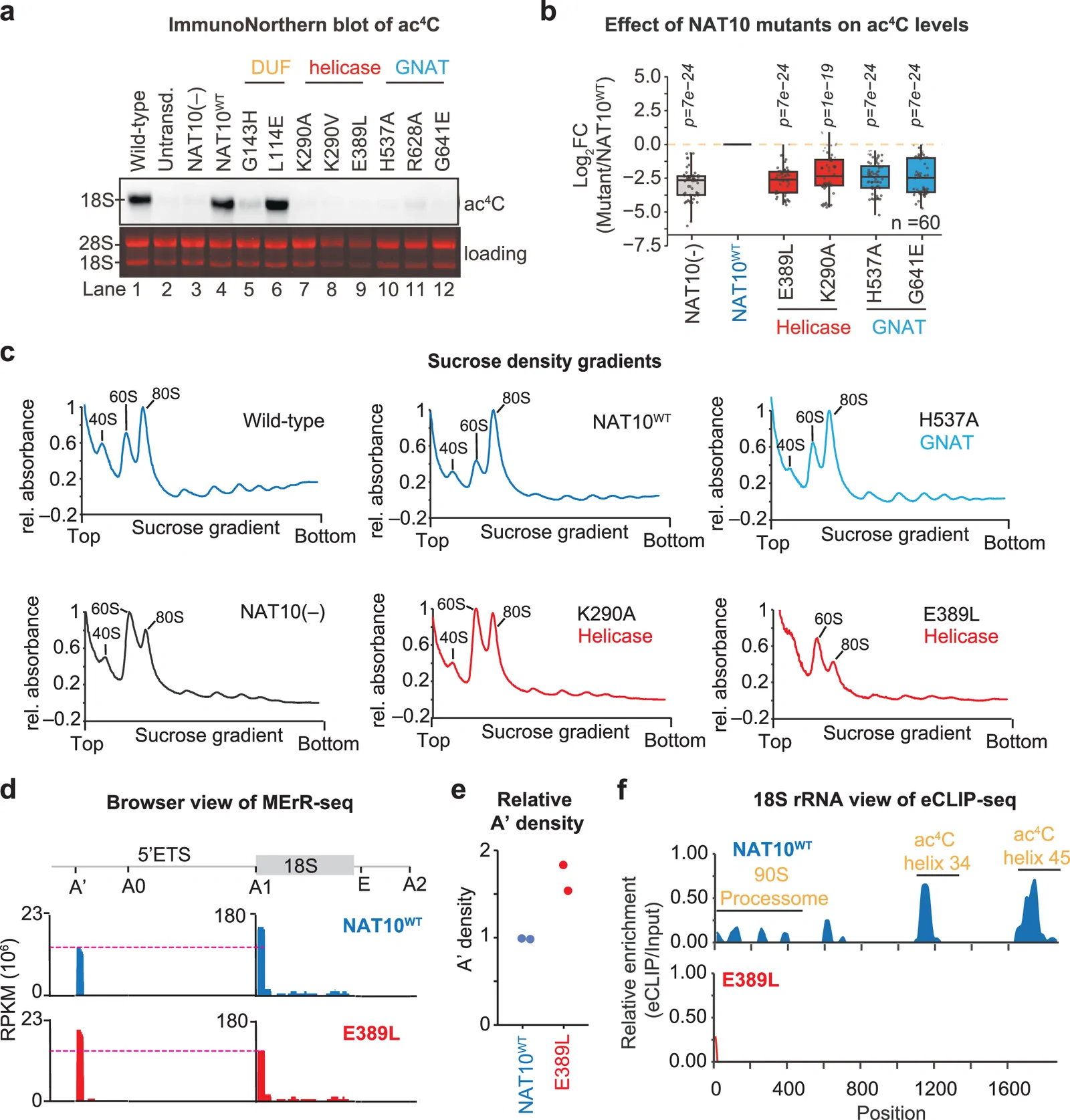
The RNA helicase domain of NAT10 is required for 18S rRNA binding and 40S biogenesis. **a** ImmunoNorthern blot of ac⁴C in NAT10^FKBP12-F36V^ Hep3B cells expressing exogenous NAT10^WT^ or the indicated mutants. Ethidium bromide staining of total RNA serves as a loading control. ImmunoNorthern Blots were performed in three independent biological replicates. **b** ac^4^C mapping was performed in two biological replicates per clone in two independent clones per group. Sites significantly decreased in the NAT10(–) condition compared to cells reconstituted with NAT10^WT^ were selected as candidate ac^4^C sites. This analysis identified 60 ac^4^C loci with mismatch rates higher than 1%. Box plots show the relative ac^4^C levels at these 60 sites in the RNA helicase and GNAT mutants, compared with NAT10^WT^. Box plots show the median, the interquartile range (25^th^–75^th^ percentile, the box), and whiskers extending to 1.5 times the interquartile range. *p* values were calculated by a two-sided Wilcoxon rank-sum test. **c** Ribosome profiles from sucrose density gradients in NAT10^FKBP12-F36V^ Hep3B cells expressing exogenous NAT10^WT^, the helicase domain mutants (E389L, K290A), or the GNAT domain mutant H537A. NAT10(–) and wild-type cells served as controls. Shown are representative profiles from three independent biological replicates. **d**, **e** RPKM values for reads mapped to the primary rRNA transcript in NAT10^WT^ (blue) and NAT10^E389L^ (red) cells (**d**). Annotated cleavage sites are indicated above each plot in (**d**). An alternative representation of data from (**d**), indicating the read density at the A′ site relative to the A₁ cleavage site, as determined by MErR-seq from two independent sequencing experiments, is shown in (**e**). **f** Browser views showing eCLIP-seq density peaks from NAT10^FKBP12-F36V^ Hep3B cells expressing exogenous NAT10^WT^ (blue) and NAT10^E389L^ (red). The values correspond to the average of three independent biological replicates. For **a**–**f**, source data are provided as a Source Data file.

Given the modest and inconsistent effects of GNAT mutants on cell proliferation and tumor growth, we next examined whether these mutations impair RNA acetylation. ImmunoNorthern blot analysis shows that re-expression of NAT10^WT^ restored 18S ac⁴C levels to those observed in wild-type cells, whereas ac⁴C was abolished in all GNAT domain mutants (Fig. 6a). Notably, mutations in the RNA helicase domain also abolished ac⁴C (Fig. 6a), indicating that the helicase domain of NAT10 is required for RNA acetylation.

To more comprehensively evaluate ac^4^C, we performed ac^4^C mapping using a combination of RetraC:T-seq and ac^4^C-seq^31,51^. In brief, total RNA was treated with NaCNBH_3_, followed by reverse transcription in the presence of the unnatural nucleotide 2-amino-dATP. This strategy reduces ac^4^C to a chemical form (tetrahydro-ac^4^C) that preferentially pairs with 2-amino-dATP (Supplementary Fig. 9a), resulting in C > T conversions at ac^4^C sites (Supplementary Fig. 9b). To identify high-confidence ac^4^C sites, we determined significantly enriched mismatches in NaCNBH_3_-treated samples compared to the respective mock-treated controls (Supplementary Fig. 9c, d). Because borohydride ions can chemically reduce other cytosine modifications, such as f^5^C and m^3^C, or induce chemical deamination^31,51,52^, we defined bona fide ac^4^C sites as those that were significantly decreased in NAT10(–) conditions compared to wild-type Hep3B or NAT10^FKBP12-F36V^ cells reconstituted with NAT10^WT^. This set of ac^4^C locations mapped to rRNA, tRNAs, and a small subset of snoRNAs and snRNAs (Supplementary Data 5). Importantly, the overall ac^4^C levels at these sites were decreased in NAT10(–) cells, cells expressing RNA helicase mutants, and cells expressing acetyltransferase mutants (Fig. 6b and Supplementary Fig. 9e, f).

ac^4^C mapping thus confirms that GNAT mutants are deficient in RNA acetylation and confirms that the RNA helicase domain of NAT10 is required for ac^4^C deposition. This observation aligns with previous studies demonstrating that the RNA helicase domain of NAT10 is essential for ac⁴C formation^20,32^. Notably, while all RNA helicase mutants consistently impaired proliferation, the acetyltransferase mutants did not, despite lacking RNA acetylation activity, indicating that the function of the RNA helicase domain of NAT10 in supporting cell proliferation is independent of its role in RNA acetylation.

NAT10 is known to promote biogenesis of the 40S ribosomal subunit^19,20,32^. However, the specific contributions of its RNA helicase and acetyltransferase domains to ribosome biogenesis in cancer remain unclear. To address this, we performed sucrose density gradient analyses in Hep3B cells expressing wild-type or mutant NAT10. NAT10(–) cells or expressing the RNA helicase mutants exhibited a reduction in the 40S peak, compared to cells expressing NAT10^WT^ or the GNAT mutant NAT10^H537A^ (Fig. 6c). Similar results were obtained in SNU-449 and K562 cells (Supplementary Fig. 10a, b). These findings suggest that the RNA helicase domain of NAT10 is required for 40S formation.

Biogenesis of the small ribosomal subunit (SSU, or 40S) begins with a primary rRNA transcript containing the 18S, 5.8S, and 28S rRNA segments, along with key regulatory regions such as the 5′ external transcribed spacer (5′ ETS)^53,54^. Following separation of the pre-40S component, the small subunit processome removes the 5′ ETS by cleaving at the A′, A₀, and A₁ sites to form a 21S precursor (Supplementary Fig. 10c). The 21S intermediate is processed into the 18S-E precursor, which is exported to the cytoplasm for further maturation into the final 40S subunit^53,54^. To identify the step at which the RNA helicase domain of NAT10 regulates 40S processing, we implemented a sequencing approach to map the 5′ ends of rRNA (Supplementary Fig. 10c). Briefly, a 5′ RNA adapter was ligated to intact rRNA prior to fragmentation, followed by ligation of a 3′ adapter, reverse transcription, and PCR amplification of libraries (Supplementary Fig. 10c). High sequencing coverage at the 5′ ends corresponding to the A′ and A₁ cleavage sites was obtained (Fig. 6d), allowing quantitation of a A′:A₁ read ratio. Compared to wild-type NAT10, the NAT10^E389L^ helicase mutant exhibited a higher A′:A₁ read ratio (Fig. 6d, e), indicating that the RNA helicase domain of NAT10 promotes A₁ cleavage.

To determine whether the RNA helicase mutants of NAT10 affect 18S rRNA binding, we performed enhanced crosslinking and RNA immunoprecipitation followed by sequencing (eCLIP-seq) (Supplementary Fig. 10d). Previous CLIP assays in Kre33^32^, the yeast ortholog of NAT10, identified three main interacting regions between NAT10 and 18S rRNA. Two of these binding regions are required for ac^4^C modification in helices 34 and 45 of 18S rRNA, while the third binding region is located in the 5’ region of 18S rRNA^32,36^ and occurs during the pre-A₁ cleavage step, when NAT10 is associated with the 90S processome^54–57^.

Our eCLIP-seq data from wild-type cells and cells reconstituted with NAT10^WT^ recapitulated these findings (Fig. 6f; and Supplementary Fig. 10e). We observed two main NAT10 binding regions upstream of the ac^4^C sites and additional binding at the 5’ region of 18S rRNA (90S processome, Fig. 6f; and Supplementary Fig. 10e). Importantly, NAT10 binding to 18S rRNA was markedly diminished in the RNA helicase mutants (Fig. 6f; and Supplementary Fig. 10e), indicating that this domain is required for NAT10 binding to 18S rRNA. Thus, the eCLIP-seq profile provides a mechanistic explanation for why disruption of the RNA helicase domain of NAT10 impairs both 40S biogenesis and ac^4^C modification; since RNA binding is required for both processes, loss of this function disrupts A₁ cleavage and ac^4^C deposition. It is crucial to note, however, that ac^4^C modification itself is not required for 40S ribosome biogenesis (Fig. 6c; and Supplementary Fig. 10a, b)^20,32–34^.

### NAT10 regulates m¹acp³Ψ deposition in 18S rRNA

Previous studies have shown that Kre33, the yeast ortholog of NAT10, can exert pleiotropic effects on 18S rRNA modifications^32^. We therefore sought to determine whether the RNA helicase domain of NAT10 regulates the landscape of 18S rRNA modifications using Nanopore direct RNA sequencing (DRS). Nucleotide modifications are known to alter the current intensity (CI) and dwell time (DT) during Nanopore DRS, and this approach has been used to detect dynamic changes in rRNA modifications^3,58^. Thus, we performed Nanopore DRS using an 18S rRNA-specific adapter in Hep3B cells expressing either wild-type NAT10 or RNA helicase mutants (Supplementary Fig. 11a). Sequencing reads were base-called using Dorado with high-accuracy models and aligned to the 18S rRNA reference using GraphMap^59^. Aligned reads were re-squiggled with nanoCEM^60^ to extract dwell time and current intensity at each annotated modification site in 18S rRNA (Supplementary Fig. 11a). Because adjacent nucleotides are known to influence the Nanopore signal^61^, dwell time and current intensity were quantified across a ± 4 nucleotide window centered on each modification (Supplementary Data 6).

**Fig. 7:**
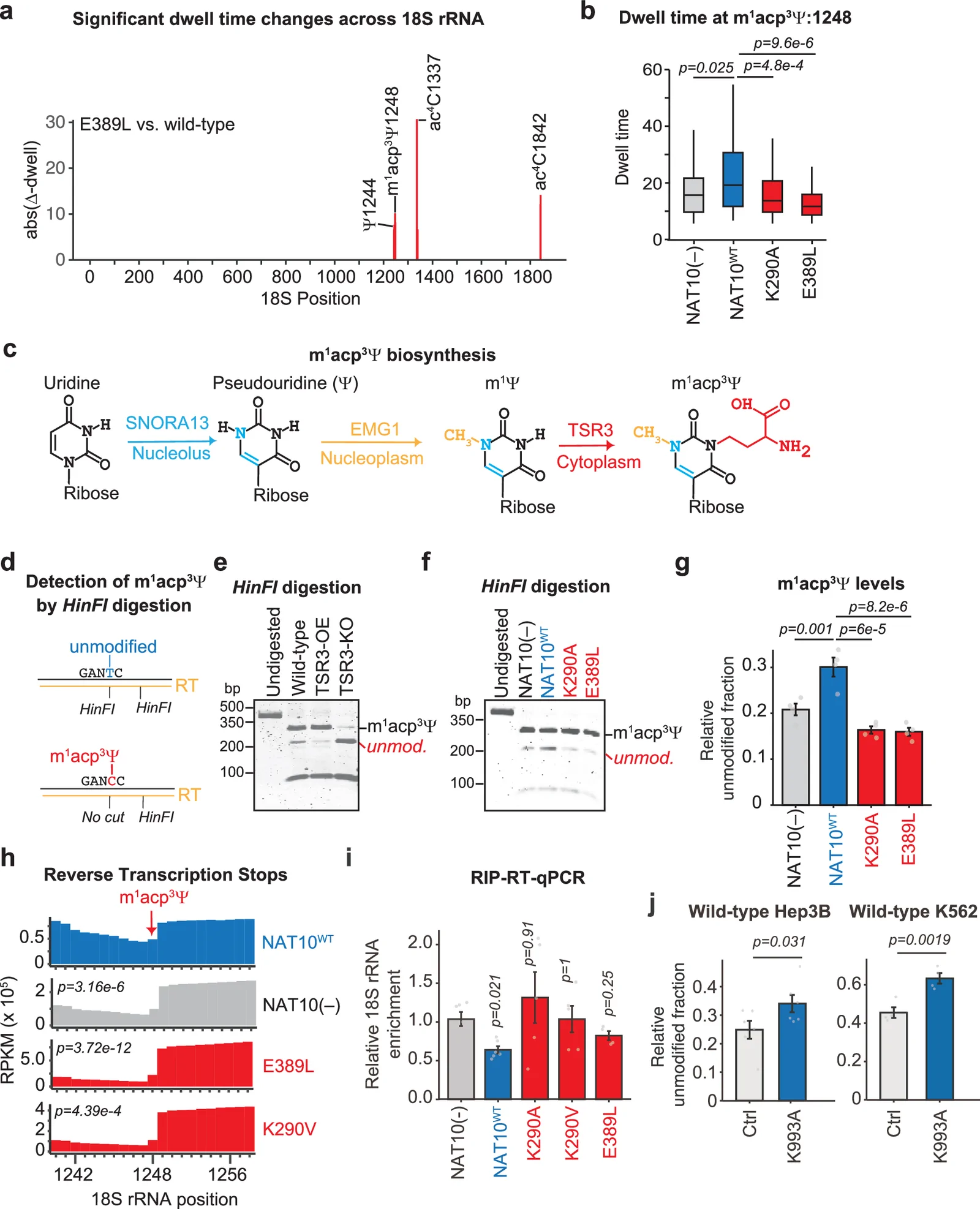
NAT10 regulates the m¹acp³Ψ modification in 18S rRNA. **a** Absolute differences in dwell time across the 18S rRNA transcript. Red Positions show sites that exhibited consistent, statistically significant differences across three independent biological groups [NAT10^K290A^, NAT10^E389L^, and NAT10(–)] relative to NAT10^WT^ in two independent biological replicates per group. Exact *p* values are provided in Supplementary Data 6. **b** Box plots showing dwell time at the m¹acp³Ψ site for the indicated genotypes. Box plots show the median, the interquartile range (25th–75th percentile, the box), and whiskers extending to 1.5 times the interquartile range. *n* = 87 for NAT10(–), *n* = 95 for NAT10^WT^, *n* = 132 for K290A, *n* = 68 for E389L. The *n* value represents the number of high-quality reads at the m¹acp³Ψ site processed by nanoCEM^60^ from two independent biological replicates per clone in three independent RNA helicase mutants. *p* values calculated via a two-sided Wilcoxon signed-rank test. **c** Schematic of the biosynthetic pathway for m¹acp³Ψ modification in 18S rRNA. **d** Schematic of the HinFI digestion assay to quantify relative m¹acp³Ψ levels in 18S rRNA. **e** Relative m¹acp³Ψ levels assessed by HinFI digestion of 18S rRNA RT-PCR amplicons from TSR3 overexpression (OE), TSR3 knockout (KO), or wild-type Hep3B cells. The gel is representative of three biological replicates. m¹acp³Ψ-modified (+) and unmodified fragments are indicated on the right. **f** HinFI digestion of 18S rRNA amplicons in NAT10^FKBP12-F36V^ Hep3B cells expressing exogenous NAT10^WT^ or the helicase mutants K290 and E389L. NAT10(–) cells served as control. Blots are representative of four biological replicates. **g** Densitometric quantification of data from (**f**). The relative unmodified fractions were calculated as unmod._band_/(m¹acp³Ψ_band_ + unmod_band_). Data represent mean ± SEM. *n* = 4. Each biological replicate represents an independent transduction. *p* values were calculated by one-way ANOVA with Dunnett’s post-hoc test relative to NAT10^WT^. **h** Browser views showing RPKMs from NAT10^WT^, NAT10^K290V^, NAT10^E389L^, and NAT10(–) cells in a ± 10 nucleotide window from the m¹acp³Ψ site. To compare RPKM distributions between genotypes, two-sample Kolmogorov–Smirnov (KS) tests were performed between each genotype relative to NAT10^WT^. **i** RIP-RT-qPCR was performed by immunoprecipitating TSR3 from NAT10(–) (*n* = 6), NAT10^WT^ (*n* = 6), NAT10^K290A^ (*n* = 5), NAT10^K290V^ (*n* = 5), and NAT10^E389L^ (*n* = 4), followed by 18S rRNA RT-qPCR. Values are calculated relative to the NAT10(–) condition. Each biological replicate represents an independent transduction. *p* values were calculated by a two-sided Welch’s ANOVA with Holm’s correction. **j** Relative unmodified fractions were determined by HinFI digestion of 18S rRNA amplicons in wild-type Hep3B cells expressing exogenous YFP (ctrl) or NAT10^K993A^. Data represent mean ± SEM. *n* = 4 for K562 (right) and *n* = 6 for Hep3B (left). Each biological replicate represents an independent transduction. *p* values were calculated using a one-tailed Student’s *t* test. For **a**, **b** and **e**–**j**, source data are provided as a Source Data file.

To ensure reproducibility, we selected events that showed significant differences in dwell time and current intensity across two helicase mutants (NAT10^K290A^ and NAT10^E389L^) and NAT10(–) cells compared to wild-type (Supplementary Data 6). This analysis revealed four 18S rRNA modifications consistently affected by the helicase domain of NAT10: ac⁴C1337, ac⁴C1842, Ψ1244, and m¹acp³Ψ1248 (Fig. 7a, b; and Supplementary Fig. 7b, c). The two ac⁴C sites serve as positive controls, as NAT10 directly catalyzes these modifications. In contrast, Ψ1244 and m¹acp³Ψ1248 are not direct substrates of NAT10. Notably, m¹acp³Ψ1248 is a dynamic rRNA modification, which is significantly reduced in cancer ribosomes compared to adjacent normal tissues^4^. The synthesis of m¹acp³Ψ involves three enzymatic steps. First, SNORA13 guides the isomerization of uridine into pseudouridine (Ψ)^7^. Next, EMG1 methylates Ψ to form m¹Ψ^62–64^. Finally, TSR3 transfers the aminocarboxypropyl (acp) group to form the fully modified base m¹acp³Ψ (Fig. 7c)^6^. Importantly, the addition of the acp³ moiety occurs in the cytoplasm during the final step of 40S maturation (Fig. 7c)^5^.

To validate the role of NAT10 in m¹acp³Ψ deposition identified via Nanopore DRS (Fig. 7b), we used a previously described assay^4^ that exploits the ability of the acp³ moiety to introduce mismatches during reverse transcription (Fig. 7d). Briefly, m¹acp³Ψ is located within a GAC**T**C motif that serves as a substrate for the restriction enzyme HinFI. m¹acp³Ψ induces mismatches during reverse transcription and PCR amplification, converting the HinFI recognition sequence from GAN**T**C to GAN**C**C, rendering it resistant to digestion (Fig. 7d). In contrast, absence of m¹acp³Ψ preserves the GANTC site, allowing HinFI digestion (Fig. 7d). A second HinFI site downstream of m¹acp³Ψ served as an internal control for digestion efficiency (Fig. 7d). To demonstrate the efficiency of this approach, we generated TSR3 overexpression and CRISPR/Cas9 knockout systems in Hep3B cells (Supplementary Fig. 7e). TSR3 knockout ablated m¹acp³Ψ levels, whereas TSR3 overexpression increased the fraction of modified 18S rRNA, as detected by the HinFI digestion assay (Fig. 7e).

We next used the HinFI digestion assay to determine the role of NAT10 in m¹acp³Ψ deposition. Because the aminocarboxypropyl moiety is deposited in the cytoplasm, we performed subcellular fractionation and measured m¹acp³Ψ levels specifically in cytoplasmic rRNA, thereby avoiding potential confounding effects from nucleolar or nuclear pre-40S precursors. In this assay, the relative proportion of unmodified rRNA (lacking the acp³ moiety at position 1248) was significantly increased in NAT10^WT^ compared to NAT10(–) cells (Fig. 7f, g), whereas expression of RNA helicase mutants significantly decreased the relative fraction of unmodified rRNA (Fig. 7f, g).

Additional evidence for the NAT10-dependent regulation of m¹acp³Ψ deposition was obtained from the input fraction of the eCLIP-seq data, where a clear reverse transcription stop was observed at the m¹acp³Ψ site (Fig. 7h). Notably, the magnitude of the reverse transcription stop, indicative of higher m¹acp³Ψ ratio, was significantly increased in RNA helicase mutants compared to NAT10^WT^ (Fig. 7h), indicating a higher proportion of unmodified rRNA in cells expressing wild-type NAT10 (Supplementary Fig. 11e). We next performed a primer extension assay using a fluorescent primer annealing downstream of the m¹acp³Ψ site. Reverse transcription with an AMV enzyme induces an RT-stop at the m¹acp³Ψ position (Supplementary Fig. 11f), which could be read through in the absence of the modification (Supplementary Fig. 11f). A higher read-through was observed in TSR3 knockout cells and Hep3B cells expressing NAT10^WT^ compared to NAT10(–) (Supplementary Fig. 11f), further indicating a higher proportion of unmodified 18S rRNA in cells expressing wild-type NAT10. However, the primer extension assay is noisier and less quantitative compared to the HinFI digestion assay.

We next sought to examine the mechanism by which NAT10 regulates the m¹acp³Ψ modification. First, we observed that NAT10 expression negatively correlates with TSR3 expression in Hep3B cells (Supplementary Fig. 11g). This reduction in TSR3 expression could lead to lower m^1^acp^3^ψ levels in cells expressing NAT10^WT^. Another hypothesis regarding how NAT10 affects m^1^acp^3^ψ deposition was formulated based on the eCLIP-seq data (Supplementary Fig. 12a, b). The two major 18S rRNA binding sites of NAT10 wrap around both ac^4^C sites and the m^1^acp^3^ψ position, which is spatially located proximal to ac^4^C:1337 in a pre-40S cryo-EM structure (Supplementary Fig. 12a-b). This observation raised the possibility that NAT10 binding to 18S rRNA could sterically hinder TSR3 from binding the pre-40S subunit, and, therefore, interfere with m^1^acp^3^ψ deposition. To test this, we performed a TSR3 RNA immunoprecipitation and 18S rRNA RT-qPCR assay (Supplementary Fig. 12c, d). Briefly, we generated a TSR3 knockout cell line on the NAT10^FBKP12-F36V^ background. We then co-transfected HA-tagged TSR3 with either a NAT10 overexpression vector or an empty vector. Next, we performed immunoprecipitation with anti-HA antibodies to pull down TSR3 and assessed its association with 18S rRNA using RT-qPCR. The results showed that NAT10 overexpression reduces the association of TSR3 with 18S rRNA, an effect that is reversed by the RNA helicase mutants (Fig. 7i).

NAT10 is predominantly localized to the nucleolus. In contrast, TSR3 is predominantly localized to the cytoplasm, where it catalyzes m^1^acp^3^ψ deposition^6^. This raises the question of how NAT10 could spatially influence TSR3 binding to 18S rRNA. Notably, NAT10 also localizes to the cytoplasm, particularly in cancers^10^. Subcellular fractionation experiments confirmed that NAT10 is present in the cytoplasm of Hep3B and K562 cells (Supplementary Fig. 12e). A co-immunoprecipitation assay showed that NAT10 and TSR3 interact (Supplementary Fig. 12f), further supporting the notion that both proteins are present in the same cellular compartment.

To test whether cytoplasmic NAT10 affects m^1^acp^3^ψ deposition, we identified a NAT10 clone that exhibits preferential localization to the cytoplasm by systematically mutating all lysine residues in the C-terminal nucleolar localization signal of NAT10 (Supplementary Fig. 12g, h). Overexpression of this cytoplasmic variant, NAT10^K993A^, in wild-type Hep3B and K562 cells, followed by the HinFI assay in cytoplasmic RNA fractions, showed that expression of NAT10^K993A^ increases the fraction of unmodified 18S rRNA (Fig. 7j).

Collectively, these findings indicate that NAT10 increases the proportion of unmodified (m^1^acp^3^ψ-deficient) 18S rRNA by reducing TSR3 expression and by interfering with TSR3 binding to 18S rRNA. While the effect of NAT10 on TSR3 expression is likely indirect, cytoplasmic NAT10 could directly interfere with the interaction between TSR3 and 18S rRNA by binding to both 18S rRNA and TSR3 (Supplementary Fig. 12a, b, f).

### Hypomodified ribosomes associate with polysomes and enhance cancer cell proliferation

**Fig. 8:**
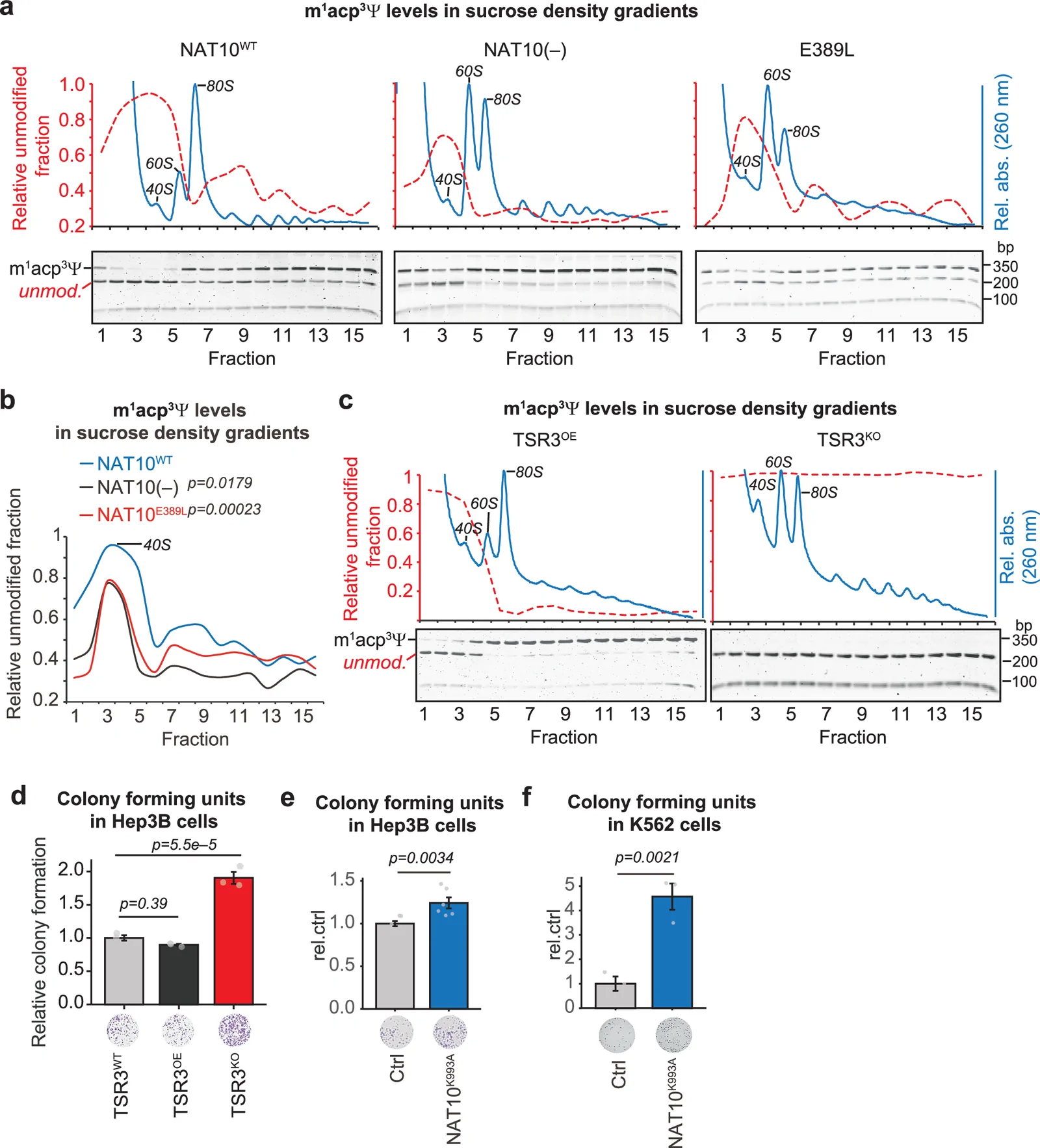
Hypomodified ribosomes are associated with polysomes and enhance cancer cell proliferation. **a** Ribosome profiles (blue lines) from sucrose density gradients in NAT10^FKBP12-F36V^ Hep3B cells expressing exogenous NAT10^WT^, NAT10^E389L^, or YFP [NAT10(–)]. Red dashed lines indicate the relative levels from the HinFI digestion assay, calculated as unmod._band_/(m¹acp³Ψ _band_ + unmod_band_). Lower panels: HinFI digestion of 18S rRNA amplicons from each gradient fraction. Each lane represents a different fraction. The sucrose density gradients were performed in four biological replicates and the HinFI assays were performed in three biological replicates, which are summarized in (**b**). **b** Averaged unmodified fraction from three biological replicates (*n* = 3) from (**a**). *p* values were calculated by two-way ANOVA. **c** Confirmation of TSR3 knockout (KO) by Sucrose density gradients and HinFI digestion assay. Ribosome profiles (blue lines) from sucrose density gradients in TSR3 overexpression (OE) and TSR3-KO cells were performed in three biological replicates, and the HinFI assay was performed in two independent biological replicates. The Red dashed lines indicate the relative unmodified levels derived from the HinFI digestion assay. **d** Quantification of colony formation assays in Hep3B cells expressing TSR3^WT^, TSR3^OE^, or TSR3^KO^. Representative images are shown below the bar plots. Data represent mean ± SEM. *n* = 3. Each biological replicate represents an independent transduction. *p* values were calculated by one-way ANOVA with Dunnett’s post-hoc test relative to TSR3^WT^. **e**, **f** Quantification of colony formation assays in wild-type Hep3B and K562 cells expressing exogenous YFP (ctrl) or NAT10^K993A^. Representative images are shown below the bar plots. Data represent mean ± SEM. *n* = 6 for Hep3B (**d**) and *n* = 3 for K562 (**f**). Each biological replicate represents an independent transduction. *p* values were calculated using a one-tailed Student’s *t* test. For (**a**–**f**), source data are provided as a Source Data file.

Reduced m¹acp³Ψ levels in rRNA may reflect a greater abundance of immature 40S subunits that are not yet competent for translation. To address this, we performed HinFI digestion assays on sucrose density gradient fractions. Absence of m¹acp³Ψ was predominantly observed in the free 40S fraction (Fig. 8a, b and Supplementary Fig. 12i), which contains pre-40S and non-translating mature 40S subunits. In the 40S subunit fractions, NAT10^WT^-expressing cells displayed a pronounced increase in unmodified rRNA compared to NAT10(–) or the RNA helicase mutant E389L (Fig. 8a, b). Notably, unmodified ribosomes were also observed in the polysome fractions, particularly in light polysomes from Hep3B cells expressing NAT10^WT^, compared to NAT10(–) or NAT10^E389L^-expressing cells (Fig. 8a, b). These findings suggest that ribosomes lacking m¹acp³Ψ may be loaded onto actively translating mRNAs. To assess whether these hypomodified ribosomes are competent for translation, we analyzed sucrose gradient fractions from TSR3 knockout cells, which exhibit normal polysome profiles (Fig. 8c). Remarkably, knockout of TSR3 resulted in fully unmodified (m¹acp³Ψ-null) ribosomes across all free subunit and polysome fractions (Fig. 8c), demonstrating that ribosomes lacking m^1^acp^3^Ψ are competent for translation.

Finally, to determine whether loss of m¹acp³Ψ levels affects cell proliferation, we performed colony formation assays in wild-type, TSR3 overexpression, and TSR3 knockout cells (Fig. 8d). TSR3 knockout, and thus complete loss of ribosomal m¹acp³Ψ, significantly enhanced cell proliferation (Fig. 8d). Overexpression of NAT10^K993A^, the cytoplasmic version of NAT10 that increases the fraction of unmodified ribosomes, also led to a significant increase in cell proliferation in wild-type Hep3B and K562 cells (Fig. 8e, f). Notably, K562 cells exhibited a higher increase in the proportion of unmodified ribosomes and cell proliferation upon expression of cytoplasmic NAT10^K993A^ (Fig. 7j; and Fig. 8f).

**Fig. 9:**
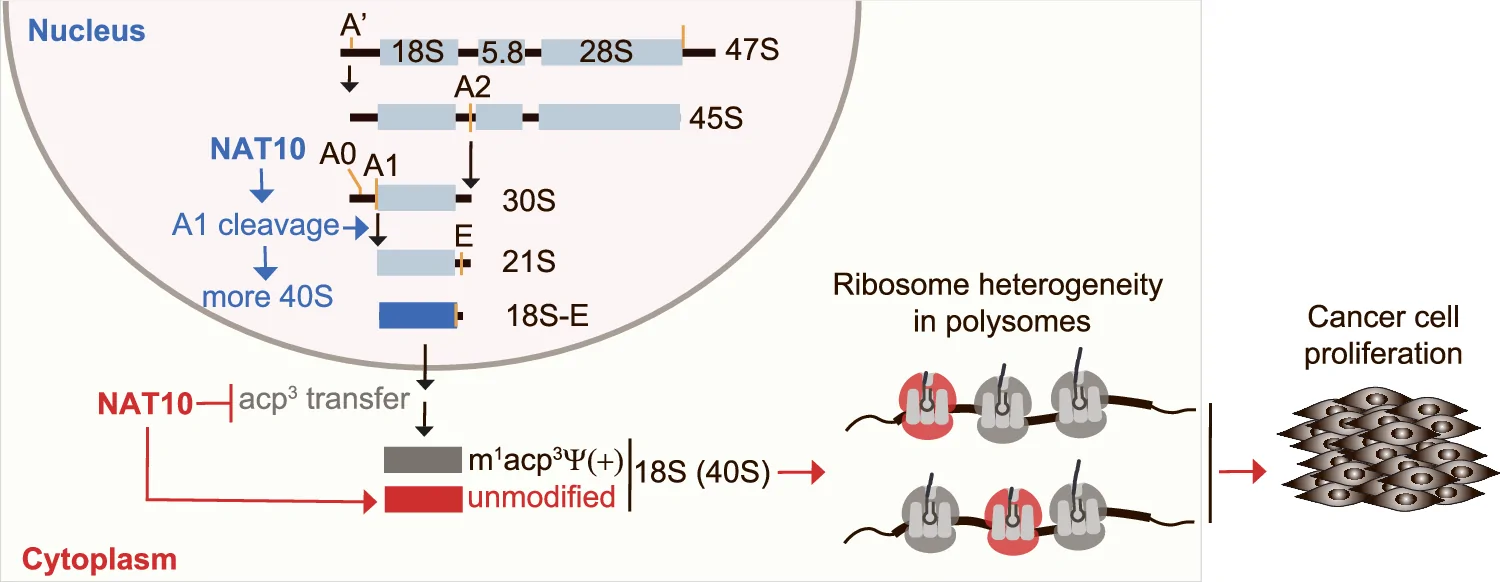
Model*.* The RNA helicase domain of NAT10 promotes 40S biogenesis in the nucleolus while reducing the TSR3-dependent transfer of acp3 to residue U1248 of 18S rRNA in the cytoplasm, resulting in the formation of hypomodified ribosome pools. The ribosomes lacking m¹acp³Ψ, which can be loaded into polysomes, contribute to ribosome heterogeneity and enhance cancer cell proliferation.

In summary, these findings indicate that the RNA helicase domain of NAT10 regulates 40S biogenesis by promoting A₁ cleavage in the nucleolus, while simultaneously reducing m¹acp³Ψ deposition in the cytoplasm (Fig. 9). Consequently, the overall increase in ribosome biogenesis and the increased ratio of unmodified 18S rRNA (lacking m¹acp³Ψ) results in a pool of hypomodified ribosomes that enhance cancer cell proliferation (Fig. 9). Given that loss of m¹acp³Ψ is a defining feature of cancer-associated ribosomes^4^, these findings implicate NAT10 in promoting this cancer-specific rRNA signature.

## Discussion

This study identifies the RNA helicase domain of NAT10 and its role in 40S biogenesis as the dominant mechanism in cancer cell proliferation and tumor growth. This conclusion is supported by the parallel analysis of thousands of NAT10 mutants across all domains of NAT10, followed by single-mutant validations in biochemical, cellular, and in vivo models of liver and myeloid cancers.

Several studies have examined NAT10 mutants, with particular emphasis on the GNAT domain variant G641E, reporting that G641E impairs cell proliferation, tumor growth, or metastasis in various cancer types^12,13,17,22–29,39–41^. However, studies using different GNAT mutants, such as H537A or R628A, have indicated that the acetyltransferase activity of NAT10 may not be required for cell proliferation^16,18,20,37,42,43^. We confirmed that G641E, H537A, and R628A eliminate the RNA acetyltransferase activity of NAT10. However, our data revealed inconsistent results for the GNAT mutants, exhibiting mutant-, cell-type-, and method-specific effects on cell proliferation that were generally less pronounced than those observed with the RNA helicase mutants. In contrast, the impact of RNA helicase mutants on cancer cell proliferation was robust and highly reproducible across all orthogonal assays and cell types.

Several studies have used the helicase mutant K290A^18,20,22,26,65^, which disrupts ac⁴C formation. This has led to the prevailing assumption that because K290A affects RNA acetylation, then ac^4^C must be required for cell proliferation. However, while the RNA helicase mutants decrease ac^4^C levels transcriptome-wide, the RNA helicase domain of NAT10 regulates cell proliferation by promoting 40S biogenesis, independently of RNA acetylation (Fig. 6). Importantly, given that ribosome production is required for cancer cell proliferation^66^, loss of 40S biogenesis explains the significant proliferative defect in cancer cells depleted of NAT10 or expressing the RNA helicase mutants. Nonetheless, this study does not exclude the possibility that the acetyltransferase activity of NAT10 may influence other oncogenic processes not examined in this manuscript, such as migration, metastasis, or epithelial-mesenchymal transition (EMT).

The physiological relevance of ac⁴C in 18S rRNA remains uncertain. One possibility is that ac⁴C serves context-dependent functions, not only in rRNA, but also in tRNA and mRNA. A recent study using THUMPD1 knockout mice suggests that tRNA acetylation contributes to selective translation under stress conditions^35^. Notably, tRNA acetylation is more prevalent in K562 than Hep3B (Fig. 1d). In addition, ac⁴C in mRNA regulates translation in position and transcript-specific manners^21,30^. This selectivity and context-specific roles of RNA acetylation may explain the mutant-, cell-type, and method-specific effects we observed with the acetyltransferase mutants.

While the deep mutational scanning assay suggests that the DUF1726 and N-terminal regions of NAT10 are relevant for cell proliferation, an in-depth characterization of these regions was not performed in this study. Notably, the ATP-binding pocket is located at the interface between the RNA helicase domain and the DUF1726 domain. Thus, the DUF1726 domain is likely required for RNA-helicase and RNA-binding functions, as previously suggested for the bacterial ortholog of NAT10^48,49^. The discrepancy between DUF1726 effects observed in deep mutational scanning and single-mutant validation may reflect differences in assay sensitivity, as variant effects are likely more detectable in competitive proliferation settings than in isolated single-mutant analyses. Nonetheless, the DUF1726 region shown in Fig. 2b,c spans the N-terminus region of NAT10 and includes the canonical start codon (M1), an alternative downstream start codon (M73; isoform ENST00000531159), and a nucleolar localization signal (residues 15–88), all of which would cumulatively contribute to the overall impact of this region on cell proliferation^10,43,67^.

Structural and biochemical analyses have shown that NAT10 associates with the 90S processome complex to promote A₁ cleavage^54–57^. The interaction of NAT10 with the 90S processome is mediated by a homodimer, where one monomer anchors to 18S rRNA, while the other remains available to engage other substrates^54–57^. Extensive remodeling of the 90S processome enables NAT10 to access distant helices 34 and 45, facilitating acetylation at both ac^4^C sites^36^. Thus, NAT10 binding to 18S rRNA is independently necessary for ribosome biogenesis and ac^4^C modification. Notably, mutations in the RNA helicase domain abolish NAT10 binding to 18S rRNA, providing a mechanistic explanation for the impairment of both A₁ cleavage and ac^4^C modification. Moreover, since NAT10 interaction with 18S rRNA requires homodimerization, the RNA helicase mutants could act as dominant negatives by altering homodimer function, thereby blocking or competing with endogenous NAT10 for rRNA binding.

This study revealed that NAT10 regulates the deposition of m¹acp³Ψ. NAT10 is typically localized to the nucleolus, but it is mislocalized to the cytoplasm in cancers^9,10^, where it can bind 18S rRNA near the m¹acp³Ψ modification site or directly interact with TSR3 (supplementary Fig. 12a, b, f), preventing TSR3 association with the 18S rRNA (Fig. 7i). Crucially, the effect of cytoplasmic NAT10 on m¹acp³Ψ deposition is likely independent of its nucleolar function, where it promotes ribosome biogenesis via A₁ cleavage. Thus, NAT10 concurrently increases both the total number of ribosomes and the proportion of m¹acp³Ψ-deficient 40S subunits, each mechanism contributing to enhanced cell proliferation (Fig. 9).

m¹acp³Ψ biosynthesis involves multiple sequential steps^6,62–64^. First, the SNORA13 H/ACA snoRNP complex isomerizes uridine to pseudouridine (Ψ). Second, EMG1 methylates the N¹ position of Ψ1248. Third, TSR3 transfers the aminocarboxypropyl group to m^1^Ψ1248. Although SNORA13 and TSR3 contribute to m¹acp³Ψ1248 formation, neither gene is essential^4–7^. Loss of SNORA13 leads to an alternative pathway that generates acp³U rather than m¹acp³Ψ^7^, while TSR3 deficiency results in the accumulation of hypomodified ribosomes lacking the acp^3^ moiety but containing m^1^Ψ^4–6^. Thus, the 1248 position of 18S rRNA is a heterogeneous residue capable of sustaining different base identities, including U, Ψ, m^1^Ψ, m^1^acp³Ψ, or acp³U. This property is exploited in cancer, which favors the assembly of cancer-specific ribosomes that lack the acp^3^ moiety at position 1248 of 18S rRNA^4^. Significantly, we found that ribosomes lacking m¹acp³Ψ are incorporated into polysomes, and a complete loss of m¹acp³Ψ through TSR3 knockout enhances cell proliferation.

Of significant physiological relevance, NAT10 knockdown impairs proliferation in cancer cells but not in normal cells with low proliferative capacity (Supplementary Fig. 7). Moreover, genetic disruption of the RNA helicase domain of NAT10 abolishes tumor formation in xenografts (Fig. 5), thereby establishing this domain as a potential therapeutic target in cancer. Yet, these results do not rule out the possibility that complete inhibition of NAT10 may affect other functions in normal cells. For instance, constitutive knockout of NAT10 is embryonically lethal, and a recent study shows that bi-allelic knockout of NAT10 affects normal hematopoiesis in mice^40^. In contrast, heterozygous loss of NAT10 has minimal impact on normal hematopoiesis^40^, but rather enhances healthspan in mice^68^, suggesting a systemic tolerance for partial NAT10 suppression. These data suggest a potential therapeutic window in which controlled inhibition of NAT10 can be exploited to enhance anti-tumor therapies.

Selective inhibition of RNA helicases has been achieved^69^, and efforts to therapeutically target them are advancing from preclinical validation to early-phase human trials. For instance, an inhibitor of DHX9 (ATX-559) was recently evaluated in a Phase 1/2 trial (NCT06625515), which was terminated due to adverse effects. In contrast, an inhibitor of eIF4A (Zotatifin/eFT226) remains in Phase 1/2 trials (NCT04092673) for solid tumors. Nonetheless, no RNA helicase inhibitor has yet received FDA approval for oncology. In the case of NAT10, the deep mutational scanning assay revealed a critical functional cluster within the ATP-binding pocket of the RNA helicase domain. This suggests that ATP-competitive inhibitors or ATP-pocket allosteric blockers could be used to target NAT10. Furthermore, because the RNA-binding activity of NAT10 is required for ribosome biogenesis and cell proliferation, inhibitors that disrupt this binding may offer an alternative therapeutic strategy.

In summary, we demonstrate that the RNA helicase domain of NAT10 promotes ribosome biogenesis and reduces m¹acp³Ψ modification, producing hypomodified ribosomes. These ribosomes enhance cancer cell proliferation. By linking NAT10-dependent ribosome remodeling to malignant growth, our results establish the RNA helicase domain as a potential therapeutic target in cancer.

## Methods

This study complies with all relevant ethical regulations. Collection, processing and experiments with cord blood samples were all performed under Cambridge Blood and Stem Cell Biobank ethics (REC reference:24/EE/0116). Cord blood was sourced from the Cambridge Blood and Stem Cell Biobank following a written informed consent. This consent does not allow the recording of race or ethnicity. All animal experiments were conducted under a UK Home Office project under the Animals Scientific Procedures Act 1986, Amendment Regulations (2012) and following ethical review by the University of Cambridge Animal Welfare and Ethical Review Body. The ethical review board that approved the use of liver samples was the NHS Health Research Authority, IRAS project ID 328327. All liver samples were obtained following a written informed consent. All other experiments were conducted under protocol BIO20250026, approved by the Northwestern University Institutional Biosafety Committee (IBC).

### Human cell lines

Hep3B (human hepatocellular carcinoma, Cat#:HB-8064, ATCC), SNU-449 (human hepatocellular carcinoma, Cat#:CRL-2234, ATCC), and HEK293T (human embryonic kidney, Cat#:CRL-3216, ATCC) cells were cultured in Dulbecco’s Modified Eagle Medium (DMEM, ThermoFisher Scientific) containing 4.5 g/L glucose, 110 mg/L sodium pyruvate, and L-glutamine (ThermoFisher Scientific) supplemented with 10% fetal bovine Serum (FBS, Corning), and 1% penicillin-streptomycin (Dot Scientific Inc). K562 (Human chronic myeloid leukemia, Cat#: CCL-243, ATCC) cells were cultured in Roswell Park Memorial Institute (RPMI 1640, HyClone) containing 2.05 mM L-glutamine, supplemented with 10% bovine calf serum (BCS, HyClone) and 1% penicillin-streptomycin (Dot Scientific Inc). OCI-AML3 (Human acute myeloid leukemia, Sanger Institute Cancer Cell Collection) cells were cultured in alpha-MEM (ThermoFisher Scientific) supplemented with 20% FBS and 1% penicillin-streptomycin. All cells were maintained at 37 °C and 5% CO₂ and passaged every three days. Cells were routinely confirmed to be mycoplasma-free. Cell lines were authenticated using the CLA-01_Cell Line Authentication service, a Short Tandem Repeat (STR) profiling method, from the Northwestern University sequencing core (NUSeq Core).

### Generation of NAT10^FKBP12-F36V^ cell lines

The proteolysis-targeting chimera (PROTAC) system was generated by integrating FKBP12^F36V^ in-frame at the C terminus of endogenous NAT10. CRISPR-Cas9-mediated cleavage of the 3′ untranslated region (3′UTR) of NAT10 was achieved using plasmid pSpCas9(BB)-2A-Puro (PX459, Addgene) containing pSpCas9-2A-GFP and two guide RNA (gRNA) sequences targeting NAT10 (pDA-59 and pDA-60; see Supplementary Data 7 for sequences). The gRNAs were cloned into PX459 using the BbsI restriction sites as previously described^70^. Homology-directed repair (HDR) donor plasmids contained 511 nucleotides (nt) of left homology arm and 438 nt of right homology arm flanking the NAT10 locus. The donor plasmid eliminated the stop codon of NAT10 and inserted a 2xHA-FKBP12^F36V^-P2A-Hygro^R^ (pDA-87) or 2xHA-FKBP12^F36V^-P2A-G418^R^ (pDA-86) cassette. The HR-donor plasmids were ordered from Twist Bioscience on the pTwist backbone.

Cells were seeded at 0.5 × 10⁶ cells per well in a 6-well plate and allowed to grow for 24 h. Next, cells were transfected with Lipofectamine™ 2000 Transfection Reagent (ThermoFisher Scientific) mixed with 0.5 µg pSpCas9(BB)-2A-Puro-NAT10_3′UTR_1 (pDA-59), 0.5 µg pSpCas9(BB)-2A-Puro-NAT10_3′UTR_2 (pDA-60), 1 µg HR-donor_1 (pDA-86), and 1 µg HR-donor_2 (pDA-87). Cells were then incubated at 37 °C for 24 h. After transfection, cells were treated with 1 µM KU-57788 [non-homologous end joining (NHEJ) inhibitor, MedChemExpress] for 48 h and then selected in the presence of 0.3 mg/mL hygromycin B (Corning) and 0.4 mg/mL G418 (Corning) for seven days. Successfully generated NAT10^FKBP12-F36V^ cells were maintained in medium containing 0.1 mg/mL hygromycin B and 0.1 mg/mL G418. Selected antibiotics were removed from the medium at least 24 h before each experiment.

### Generation of the site saturation mutagenesis library

A lentiviral plasmid containing full-length NAT10 (NM_024662.3) was purchased from VectorBuilder. The parental plasmid, named pLV[Exp]-mCherry:T2A:Bsd-EF1A > hNAT10 (pDA-98), contained NAT10 under the control of an EF1A promoter with a 3′ Woodchuck Hepatitis Virus Posttranscriptional Regulatory Element (WPRE). The vector expressed the fluorescent marker mCherry and the antibiotic blasticidin under the control of a CMV promoter, separated by a T2A peptide (Supplementary Fig. 2b).

For site-saturation mutagenesis, we selected 100 amino acid residues (Supplementary Data 1). Mutations were distributed across all domains of NAT10, with each residue substituted by all 20 possible amino acids or a premature stop codon. Residues for mutagenesis were selected based on phylogenetic conservation, with 88 sites conserved across species or shown to be functionally relevant in the literature, and the remaining 12 residues exhibiting limited conservation (Supplementary Data 1). A wild-type clone was maintained for every residue. Twist Bioscience produced the site-saturation mutagenesis library, including a unique barcode for each residue, and cloned it into pLV[Exp]-mCherry:T2A:Bsd-EF1A > hNAT10 (Supplementary Fig. 2b). The library was verified by next-generation sequencing, yielding a total of 1910 individual mutants (Supplementary Fig. 2c).

To amplify the lentiviral library, pooled plasmid DNA was electroporated into Endura DUOs electrocompetent bacteria (Lucigen) using a MicroPulser Electroporator (Bio-Rad). Four independent electroporations were performed, each using 100 ng plasmid DNA per 25 µL of electrocompetent bacteria. After electroporation, each sample was transferred to 5 mL SOC medium and incubated in an orbital shaker for 1 h at 225 rpm at 37 °C. A total of 2.5 mL cell suspension was then plated onto 15-cm agar dishes. Plates were incubated at 30 °C for 18 h. Bacterial monolayers were scraped into LB medium, and plasmid DNA was extracted using a HiSpeed Maxiprep Kit (Qiagen). Lentiviral particles were produced by co-transfecting the plasmid library with the packaging vectors psPAX2 (Addgene) and pMD2.G (Addgene) into HEK293T cells.

### Generation of individual NAT10 mutants

Individual lentiviral NAT10 mutants were generated by site-directed mutagenesis using the Q5 Site-Directed Mutagenesis Kit (New England Biolabs, NEB), with pLV[Exp]-mCherry:T2A:Bsd-EF1A > hNAT10 (pDA-98) as the parental plasmid and the primers listed in Supplementary Data 7. Mutations were confirmed by Sanger sequencing using the primers listed in Supplementary Data 7. Vectors were propagated in Stbl3 cells, purified by Maxiprep (Qiagen), and packed into lentiviral particles by co-transfection into HEK293T cells with the packaging vectors psPAX2 (Addgene) and pMD2.G (Addgene). All vectors used in this study are listed in Supplementary Data 7.

A GFP-NAT10 construct was cloned into the pcDNA™5/FRT expression vector (ThermoFisher Scientific) by Gibson assembly using the NEBuilder® HiFi DNA Assembly Master Mix (New England Biolabs). Individual mutants of the C-terminal nucleolar localization signal were generated by site-directed mutagenesis using the Q5 Site-Directed Mutagenesis Kit (New England Biolabs, NEB), with pcDNA™5/FRT > GFP-NAT10 (pDA-133) as the parental plasmid and the primers listed in Supplementary Data 7.

Individual mutants of FLAG-NAT10 were generated by site-directed mutagenesis using the Q5 Site-Directed Mutagenesis Kit (New England Biolabs, NEB), with pICE>FLAG-NAT10 (Addgene: 59365) as the parental plasmid and the primers listed in Supplementary Data 7.

### Transduction with NAT10 clones

Cells were seeded at a density of 2 × 10⁵ cells/mL in 10-cm tissue culture dishes with 10 mL of complete DMEM containing 10% FBS and incubated for 24 h at 37 °C with 5% CO₂. Cells were treated with Polybrene Transfection Reagent (Sigma-Aldrich, TR-1003) at 8 μg/mL for 10 min. Lentiviral particles were added to the cultures. After 24 h, the medium was replaced with fresh complete medium containing 5 μg/mL Blasticidin S HCl (ThermoFisher Scientific), and cells were selected for 5 days. To avoid biases related to random integration and selection, all biological replicates represent independent transductions.

### Generation of TSR3 overexpression (OE) and knockout (KO) cells

To generate TSR3-OE and KO cells, 0.25 × 10^6^ cells were seeded per well in 6-well plates and incubated for 24 h. For TSR3-OE, cells were treated with 8 μg/mL Polybrene (Sigma-Aldrich, TR-1003) for 10 min, followed by transduction with pLV[Exp]-EGFP-EF1A > TSR3 (pDA−182, Supplementary Data 7). Twenty-four h after transduction, the medium was replaced, and cells were incubated for an additional 3 days. For TSR3-KO, 2.5 µg of pRP[CRISPR]-EGFP-hCas9-U6 > TSR3 (pDA-183, Supplementary Data 7) and Lipofectamine™ 2000 Transfection Reagent were mixed with Opti-MEM and incubated at room temperature for 10 min. The mixture was added dropwise to cells in each well, then gently mixed and incubated for 3 days. After three days, the brightest EGFP-positive cells from both TSR3-OE and TSR3-KO populations were sorted using a SH800S Flow Cytometer/Cell Sorter (Sony). Sorted cells were reseeded and expanded polyclonally until TSR3 overexpression or knockout was verified by Western blot.

### Western blot

Cells were washed with cold PBS and lysed in 1 × Triton lysis buffer containing 50 mM Tris-HCl (pH 7.5), 150 mM NaCl, 2 mM EDTA, 1% Triton-X, 0.5 mM freshly prepared DTT, and 1 × freshly prepared protease inhibitor cocktail (ThermoFisher Scientific). Lysates were centrifuged at 15,500 × *g* for 10 min at 4 °C, and protein concentration was determined using either the Quick Start 1 × Bradford Reagent (Bio-Rad) or Pierce^TM^ BCA Protein Assay Kit (ThermoFisher Scientific). Twenty-five micrograms of protein were separated by 12% SDS-PAGE at 100 V for 2.5 h and transferred to a nitrocellulose membrane for 16 h. Membranes were blocked with 5% non-fat milk in Tris-buffered saline with 0.05% Tween-20 (TBST) for 2 h at room temperature. Membranes were incubated for 16 h at 4 °C with one of the following primary antibodies: rabbit polyclonal anti-NAT10 (1:2500, Cat#:13365-1-AP, Proteintech); rabbit polyclonal anti-NAT10 (1:2500, Cat#:PA5-31376, ThermoFisher Scientific); rabbit polyclonal anti-TSR3 (1:500, Cat#: PA5-83514, ThermoFisher Scientific); rabbit monoclonal anti-HA-Tag (C29F4) (1:2000, Cat#:3724, Cell Signaling Technology); mouse monoclonal anti-GAPDH (1:1000, clone 6C5, Cat#:sc-32233, Santa Cruz Biotechnology); mouse monoclonal anti-β-actin antibody (1:2000, Cat#:66009-1-Ig, Proteintech); polyclonal anti- β-tubulin antibody (1:2000, Cat#:10094-1-AP, Proteintech); or rabbit anti-Lamin A (LMNA) antibody (1:2000, Cat#:A303-433A, Fortis Life Sciences). Membranes were washed three times with 0.05% TBST and incubated with HRP-conjugated anti-rabbit IgG (1:5000, Cat#: 7074, Cell Signaling Technology) or HRP-conjugated anti-mouse IgG (1:5000, Cat#: 7076, Cell Signaling Technology) for 2 h at room temperature. Signals were detected using ECL Western Blotting Substrate (Promega) and visualized with a Sapphire multimode imager (Azure Biosystems).

### ImmunoNorthern blot

DNase-treated total RNA (4–10μg) was denatured at 65 °C for 10 min, then placed on ice for 1 min. Denatured RNA was separated by electrophoresis on a 1% agarose denaturing gel at 150 V for 20 min. The gel was imaged before capillary transfer to a membrane using 20 × SSC buffer for 16 h. Membranes were washed with 1 × PBS, crosslinked at 120 mJ/cm^2^ with UV_254nm_ (Fisherbrand), and then blocked with 5% non-fat milk in 0.05% Tween-20/PBS (PBST) for 2 h at room temperature. Membranes were incubated overnight at 4 °C with anti-ac⁴C antibody (1:1000, Abcam), in 2.5% non-fat milk. Membranes were washed three times with 0.05% PBST, then incubated for 2 h at room temperature with HRP-conjugated anti-rabbit IgG (1:2500, Cell Signaling Technology) in 2.5% non-fat milk. Membranes were washed three times with 0.05% PBST and developed using SuperSignal ELISA Femto Maximum Sensitivity Substrate (ThermoFisher Scientific). Blots were visualized using a Sapphire multimode imager (Azure Biosystems).

### Deep mutational scanning and nanopore sequencing (DMS-Nseq)

NAT10^FKBP12-F36V^ cells (1 × 10^6^) were transduced at limiting dilution with the site-saturation mutagenesis library to ensure that fewer than one clone was delivered per cell. Twenty-four h after transduction, cells were treated with 100 nM dTAG^V^-1. Viable cells were sorted at day 0 and day 12 after dTAG^V^-1 treatment using Zombie Violet™ Fixable Viability Kit (BioLegend) on a SH800S Cell Sorter (Sony). Three biological replicates were performed per cell line per time point. Genomic DNA was isolated from sorted cells using GeneJET Genomic DNA Purification Kit (ThermoFisher Scientific) according to the manufacturer’s protocol. Exogenous NAT10 was amplified by PCR from genomic DNA using primers DAP-514/DAP-515 (see Supplementary Data 7) and resolved on a 1% agarose gel. The fragment of interest was gel-extracted using the GeneJET Gel Extraction Kit (ThermoFisher Scientific) according to the manufacturer’s protocol. Amplicons were end-prepped using the NEBNext Ultra II End Repair/dA-Tailing Module (NEB). End-prepped DNA was barcoded using the Native Barcoding Kit 24 V14 (Oxford Nanopore, SQK-NBD114.24) and Blunt/TA Ligase Master Mix (NEB). Barcoded samples were pooled and ligated to sequencing motor proteins using Quick T4 DNA Ligase (NEB). The final library was purified with 0.4× AMPure XP beads (Beckman Coulter) and quantified using Qubit dsDNA Quantification Assay Kits (ThermoFisher Scientific). A DNA pool of 12 µL was mixed with 37.5 µL sequencing buffer (SB) and 25.5 µL library solution (LIS), yielding a final volume of 75 µL, which was then loaded onto a FLO-MIN114 flow cell (Oxford Nanopore) and sequenced on a Mk1B MinION.

### Analysis of the deep mutational scanning assay

POD5 files were base-called and demultiplexed using Dorado v0.5.3 with the dorado basecaller sup and --kit-name SQK-NBD114-24 settings. The first demultiplexing step generated twelve FASTQ files. Samples included three biological replicates each for day 0 and day 12 for both K562 and Hep3B cells. Next, each FASTQ file was then split according to custom barcodes corresponding to each residue. A custom script employing regular expressions (regex) in Biopython was used to search for barcode sequences in both sense and antisense orientations and to count the number of reads containing each barcode. The script allowed one mismatch per barcode and output only the best match for each read. The second demultiplexing step produced 100 distinct FASTQ files per sample.

Next, reads were aligned to the reference NAT10 sequence (NM_024662.3) using minimap2 v2.24^71^ with the -ax map-ont setting, and alignments were sorted and indexed with samtools v1.16.1^72^. Variant counts at each targeted residue were obtained using a custom Python script. The script enumerates all possible 64 codons and assigns each to its respective amino acid or stop codon. The count_trinucleotides_from_bam() function processes BAM files, validates each read, and extracts codon sequences from overlapping regions based on coordinates for each mutated residue provided in a BED file. The output consists of substitution counts for each barcode and residue (Supplementary Data 3, columns A–K).

Counts were analyzed using DESeq2^73^ in R v4.1.1 to compute log₂ fold-change values, with an adjusted *p* value threshold of 0.05 (Supplementary Data 3). To further characterize the distribution of mutations, barcodes were extracted and merged with domain annotations (DUF_N_ter, helicase, GNAT, or tRBD_CC_NoLS). Pairwise Wilcoxon rank-sum tests were performed to assess differences in mutation effects across protein domains (Fig. 2b, c). Mutations were further classified as stop codons, wild-type (WT), or missense variants, and a second statistical analysis was conducted to compare these groups within each NAT10 domain (Fig. 2d, e and Supplementary Fig. 2d, e).

To generate Fig. 3a, b wild-type (WT) and stop codon mutants were excluded from the dataset prior to aggregating barcode counts at day 0 and day 12. Summed barcode counts were used as input for differential analysis with DESeq2, following standard pre-filtering to retain barcodes with sufficient read depth. Significance was determined based on adjusted p-values (padj <0.05). The MS score was calculated as log₂ fold-change multiplied by the negative log₁₀ of the adjusted p-value. The final dataset, including barcode counts and DMS scores, is provided in Supplementary Data 4.

### MTS assay

Following transduction and selection with blasticidin S, 1 × 10³ cells were seeded in 96-well plates and allowed to adhere for 24 h. Cells were then treated with 100 nM dTAG^V^-1 and incubated for the indicated durations. At each endpoint, 5 µL CellTiter 96® AQueous One Solution was added per well, and plates were incubated at 37 °C for 1 h. Absorbance was recorded at 490 nm using a BioTek Synergy LX plate reader (Agilent).

### Zombie violet assay

Following transduction and selection with blasticidin S, 2.5 × 10^5^ cells were seeded in 6-well plates and allowed to sit for 24 h. Cells were then treated with 100 mM dTAG-V1 and allowed to grow for 10 days. To maintain cell nutrition, fresh media containing 100 nM dTAG-V1 was added every 3 days until the final day of cell collection. Cells were harvested and washed twice with 1 × PBS followed by staining with Zombie Violet™ Fixable Viability Kit (BioLegend®) at a dilution of 1:1000 at room temperature in the dark for 30 min. Cell viability was acquired at 10,000 events per sample using an SH800S Flow Cytometer/Cell Sorter (Sony). Results were analyzed using FlowJo (version 10).

### Competitive proliferation assays

Following transduction of OCI-AML3 cells and selection with blasticidin S, the percentage of transduced cells (mCherry + ) was measured using the CytoFlex LX instrument (Beckman Coulter). Then, 50% of mCherry+ cells were co-cultured with 50% of non-transduced cells for a competition assay. The levels of mCherry+ were measured every 3 days using the CytoFlex LX instrument. The data were analyzed using FlowJo (Version 10).

### OCI-AML3 differentiation

Following transduction of OCI-AML3 cells and selection with blasticidin S, the levels of the differentiation marker CD11b were measured three days post-selection. In brief, 0.1 × 10^4^ cells were blocked with a solution containing 2% FBS for 15 min on ice, then incubated with 1:200 anti-human-CD11b antibody (Clone 1CRF44, Cat.#. 982604, BioLegend) for 20 min. Cells were washed and resuspended in a flow cytometry buffer containing 0.1 μg/ml DAPI. Flow cytometry analysis was performed using a CytoFLEX LX instrument (Beckman Coulter) and analyzed using FloJo (Version 10).

### Colony formation assays in cancer cell lines

Following transduction and selection with blasticidin S, Hep3B and SNU-449 cells were seeded at a density of 1 × 10³ cells per well in 24-well plates. Cells were cultured for 10 days in the presence of 100 nM dTAG^V^-1, with fresh medium containing 100 nM dTAG^V^-1 added every three days. Cells were then washed with 1 × PBS (Corning) and fixed with 10% formaldehyde for 15 min. Cells were permeabilized with 100% methanol for 20 min, stained with 0.2% crystal violet (APExBIO) for 20 min, and washed five times with distilled water to remove residual stain. Plates were air-dried before imaging. Colonies were analyzed using ImageJ (v1.54c).

For K562, cells were seeded into a 12-well plate (1.0 × 10^4^ cells/well) containing H4034 methylcellulose (StemCell Technologies). The colonies were allowed to grow for 10 days. Colonies were stained with 100 μL of 5 mg/mL iodonitrotetrazolium chloride (INT, MedChemExpress) per well and incubated at 37 °C for 2 h. Images of colonies were captured and counted using ImageJ (v1.54c).

### Colony formation and competitive proliferation assays in murine hematopoietic progenitors

Bone marrow was freshly isolated from 6- to 10-week-old female Rosa26^Cas9/+^; Flt3^ITD/+^ mice or moribund Npm1^flox−cA/+^; Flt3^ITD/+^ mice. Bone marrow cells were subjected to erythrocyte lysis (BD Pharm Lyse, BD Biosciences), followed by magnetic bead selection of Lin⁻ cells using the Lineage Cell Depletion Kit (Miltenyi Biotec) according to the manufacturer’s instructions. Lin⁻ cells were cultured in X-VIVO 20 (Lonza) supplemented with 5% BIT serum (StemCell Technologies) 10 ng/mL IL3 (Peprotech), 10 ng/mL IL6 (Peprotech), and 50 ng/mL of SCF (Peprotech). Since Lin- cells from Rosa26^Cas9/+^, Flt3^ITD/+^; Rosa26^Cas9/+^ are normal, these cells were further transduced with viral particles expressing the retrovirus constructs pMSCV-MLL-AF9-IRES-YFP to induce an aggressive acute myeloid leukemia phenotype. Transduced cells were then sorted for YFP (for MLL-AF9). All primary murine cells were transduced with lentiviral vectors expressing gRNAs targeting NAT10 or with empty vectors as controls (see Supplementary Data 7).

For colony formation assays, 5000 lineage-negative cells expressing Cas9 and transduced with lentiviral vectors were plated in triplicate in 6-well plates containing M3434 methylcellulose medium (StemCell Technologies). Cells were collected from colonies after seven days, re-counted, and a further 5000 cells were replated. The same replating strategy was repeated weekly for up to three rounds.

For competitive proliferation assays, the percentage of blue fluorescent protein (BFP)-positive cells was measured between days 4 and 12 post-transduction using the CytoFlex LX instrument. The data were analyzed using FlowJo (Version 10) and normalized to the percentage of BFP-positive cells at day 4.

### Colony formation assays in human CD34⁺ hematopoietic cells

Cord blood-derived CD34⁺ cells were assessed for colony-forming efficiency in StemMACS HSC-CFU semi-solid medium (Miltenyi, 130-091-280) in the presence of either scrambled or NAT10-targeting shRNA vectors. Colonies were counted by microscopy 12–14 days after plating. All cord blood samples were obtained with informed consent under UK ethical approval (IRAS ref: 340167, formerly 149581; REC 07-MRE05-44).

### In vivo xenotransplantation experiments

For Hep3B xenograft experiments (Fig. 5), 16-week-old female NSG mice (NOD.Cg-Prkdc^scid Il2rg^tm1Wjl/SzJ; Charles River Laboratories) were injected subcutaneously in the left flank with 2 × 10⁶ Hep3B cells suspended in a 1:1 mixture of Matrigel (Corning, Cat# 354230) and PBS (Sigma, D8537; total volume 200 μL). The cell lines used were YFP vector control, NAT10 wild-type, RNA helicase domain mutant (E389L), and GNAT domain mutant (H537A). Cells were cultured under standard conditions as described above. Prior to transplantation, cells were treated in vitro with 100 nM dTAG^V^-1 (Tocris, Cat# 6914, Batch 6 A/298430) for 48 h. dTAG^V^-1 was also co-administered on the day of injection at a final concentration of 0.140 μg/mL in the PBS phase. Tumors were measured three times per week using a digital caliper (Faithfull). Tumor area was calculated as length × width, and volume as (length × width²)/2. All animals were culled when tumor area approached the humane endpoint limit (1.2 cm²) in any cohort, as specified in the animal license. The animals were euthanised using cervical dislocation followed by cessation of circulation. After culling, representative tumors from each cohort were excised, weighed, and imaged.

For K562 xenograft experiments (Fig. 5e), 12-week-old female NSG mice (NOD.Cg-Prkdc^scid Il2rg^tm1Wjl/SzJ; Charles River Laboratories) were injected intravenously with 0.5 × 10^6^ K562 cells. The two cell lines used were NAT10 wild-type and the RNA helicase mutant E389L. Cells were treated in vitro for 24 h with 100 nM dTAG^V^-1 (Tocris, Cat# 6914, Batch 6 A/298430) prior to transplantation, and dTAG^V^−1 was co-administered on the day of injection at a final concentration of 0.140 μg/mL. Mice were monitored daily, and weight measurements were taken twice per week. Animals were culled after humane endpoints were reached. The animals were euthanised using cervical dislocation followed by cessation of circulation.

Mice were housed under specific pathogen-free conditions in individually ventilated cages at the University of Cambridge Biomedical Services (UBS) facility. Cages were maintained on a 12 h:12 h light:dark cycle (lights on at 07:30) in a temperature- and humidity-controlled room (22.2  ±  1.1 °C; 30–70% relative humidity). All procedures were conducted in accordance with the Animals (Scientific Procedures) Act 1986 (UK) and were approved by the University of Cambridge Animal Welfare and Ethical Review Body. Randomization and blinding were not applied. Female animals were chosen in accordance with the approved animal protocol and in line with 3Rs. Using female mice avoids the severe physical injuries and chronic stress caused by aggression and fighting among co-housed male mice. These stress factors can unpredictably alter xenograft behavior and confound data. Thus, this study focuses on a single sex to maintain a highly controlled, uniform baseline to test our hypothesis. Because sex was not an experimental variable, the result can only be interpreted in the context of general in vivo tumor growth and must not be interpreted in the context of biological sex differences.

### Intrahepatic cholangiocyte organoids (ICOs)

Liver samples were obtained from Addenbrookes Hospital with informed consent and ethical approval from the healthy margins of surgical liver resections (REC: 23/NS/0106). Organoids were derived from human liver tissue and maintained as previously described^74,75^. Organoids were transduced with lentiviral particles encoding two different shRNAs against NAT10 or a scramble control. After one week of recovery, organoids were dissociated with TryplE (12605028, Gibco) to single cells, before replating and culturing in 1 µg/mL puromycin (J67236, ThermoFisher). Puromycin selection was maintained thereafter.

For viability assessment, 1 µg/mL doxycycline (D9891, Sigma) was added at five days post-split, and organoids were cultured in control or 1 µg/mL doxycycline-containing media for an additional three days. Cell viability was assessed using the PrestoBlue^TM^ HS reagent (P50201, ThermoFisher) following the manufacturer’s instructions. PrestoBlue^TM^ HS was added to the cells at 1:10 dilution in culture media and incubated for 1 h. The media was then transferred to black-walled 96-well plates, and fluorescence was determined by plate reader (SpectraMax M2^E^, Molecular Devices). Endpoint values were normalized to PrestoBlue^TM^ HS fluorescence readings taken immediately before adding doxycycline.

For immunofluorescence, organoid cultures were fixed with 4% PFA (w/v; 28908, ThermoFisher) for 20 min at 4 °C and subsequently washed 3 times in PBS. Fixed organoids were then permeabilized and blocked in PBS (14190-144, Gibco) supplemented with 0.1% Triton-X (w/v; A16046.AE, ThermoFisher) and 10% donkey serum (v/v; ab7475, Abcam) for 1 h. Samples were incubated with primary antibodies, goat anti-EpCAM (1:200, RnD Cat#: AF960), mouse anti-Cytokeratin 19 (1:500, antibodies.com Cat#: A86355), and rabbit anti-SOX9 (1:500, Abcam Cat#: ab185230) in PBS supplemented with 0.1% Triton-X and 1% donkey serum overnight at 4 °C. The following day, samples were washed three times for 45 min with PBS. Secondary antibodies Alexafluor Anti-Goat IgG 647 (1:1000, ThermoFisher Scientific, Cat#: A21206), Alexafluor Anti-mouse IgG 568 (1:1000, ThermoFisher Scientific, Cat#: 10037), and Alexafluor Anti-Rabbit IgG 488 (1:1000, ThermoFisher Scientific, Cat#: A21447) were added in PBS supplemented with 0.1% Triton-X and 1% donkey serum for 60 min at room temperature. DAPI (0.1 µg/mL; 40043, Biotium) was added to PBS and incubated for 20 min at room temperature. Samples were subsequently washed three times with PBS. Images were acquired using the Zeiss LSM 710 confocal microscope.

### Real-time quantitative polymerase chain reaction (RT-qPCR)

Total RNA (1 μg) was reverse transcribed to cDNA using SuperScript™ IV Reverse Transcriptase (ThermoFisher Scientific). Gene expression was measured by real-time quantitative PCR (RT-qPCR) with PowerUp™ SYBR™ Green Master Mix (Applied Biosystems). Thermal cycling conditions were as follows: 50 °C for 2 min, 95 °C for 2 min, followed by 40 cycles of 95 °C for 15 s and 60 °C for 1 min. Relative gene expression was calculated using the 2^−△△Ct^ method (Livak and Schmittgen). Expression values were normalized with GAPDH. Primer sequences are listed in Supplementary Data 7.

### Sucrose density gradients

Cells were seeded at a density of 2 × 10⁵ cells/mL in 10-cm tissue culture dishes with complete DMEM containing 10% FBS and incubated for 24 h at 37 °C with 5% CO₂. Cells were treated with 100 nM dTAG^V^-1 and incubated for an additional 48 h to deplete endogenous NAT10 and allow time for ribosome turnover. After incubation, cycloheximide (CHX) was added to the medium at a final concentration of 100 μg/mL before aspirating the medium. Plates were placed in liquid nitrogen and then transferred to ice. Lysis buffer (10 mM Tris-HCl [pH 7.4], 5 mM MgCl_2_, 100 mM KCl, 1% Triton X-100, 2 mM DTT, 100 μg/mL CHX, 1 × Murine RNase inhibitor, 1× protease inhibitor cocktail) was added to plates, and cells were scraped into the lysis buffer. Lysates were centrifuged at 15,500 × *g* for 10 min, and the supernatant was transferred to a new 1.5 mL tube, frozen in liquid nitrogen, and stored at −80 °C until fractionation. Sucrose gradients (10–40% sucrose, 10 mM Tris-HCl [pH 7.5], 75 mM KCl, 1.5 mM MgCl_2_ for K562 and Hep3B; 10–40% sucrose, 20 mM Tris-HCl [pH 7.5], 150 mM NaCl, 5 mM MgCl_2_, for SNU-449) were generated using a BioComp Gradient Master. Cell lysates were loaded onto gradients and centrifuged at 168,660 × *g* in a Beckmann Coulter SW41 Ti rotor for 2 h at 4 °C. Fractionation, recording of 260 nm absorbance, and fraction collection were performed using a BioComp Density Gradient Fractionation system.

### RNA isolation from polysome fractions

RNA from each collected fraction was isolated by mixing 500 µL of the fraction with 500 µL Trizol, followed by addition of 200 µL chloroform. The mixture was incubated at room temperature for 5 min and centrifuged at 12,000 × *g* for 15 min at 4 °C. The aqueous phase was transferred to a new 1.5 mL Eppendorf tube, followed by addition of 500 µL 100% ice-cold isopropanol and 3 µL linear acrylamide. The mixture was thoroughly mixed and incubated at −20 °C for 1 h, then centrifuged at 20,000 × *g* for 1 h at 4 °C. The supernatant was discarded, and the pellet was washed with 500 µL 100% ice-cold ethanol by centrifugation at 20,000 × *g* for 10 min at 4 °C. Ethanol was removed, and the RNA pellet was air-dried at room temperature for 5 min, then resuspended in 100 µL nuclease-free water. RNA was heated at 65 °C for 5 min, immediately placed on ice, and mixed thoroughly. Standard DNase treatment was performed, and RNA was isolated again using the modified Trizol extraction procedure described above.

### Isolation of cytoplasmic RNA

NAT10^FKBP12-F36V^ cells were seeded at a density of 0.25 × 10⁶ cells per well in 6-well plates and incubated for 24 h. Cells were then transduced with lentiviral plasmids carrying NAT10^WT^ or the indicated mutations. Following transduction, cells were selected with 5 µg/mL Blasticidin (ThermoFisher Scientific) for 5 days. Cells were then grown for 48 h in antibiotic-free medium, treated with 100 nM dTAG^V^-1, and incubated for additional 48 h to deplete endogenous NAT10 and allow time for ribosome turnover. On the final day of treatment, culture medium was removed, and cells were washed three times with 1 × PBS to remove dead cells. Live cells were detached by trypsinization, transferred to a 15-mL tube, and centrifuged at 330 × *g* for 5 min. The cell pellet was gently resuspended in ice-cold 1 × PBS, transferred to a pre-chilled 1.5-mL tube, and centrifuged at 720 × *g* for 5 min.

The cell pellet was resuspended in 500 µL ice-cold Hypotonic Buffer A (10 mM HEPES, pH 7.9, 1.5 mM MgCl_2_, 10 mM KCl, 0.5 mM DTT, 1 × protease inhibitor, and 40 U/mL Murine RNase inhibitor) and incubated on ice for 20 min, followed by lysis using a 2 mL Dounce homogenizer (30 strokes). The lysate was centrifuged at 720 × *g* for 5 min at 4 °C, and 350 µL of the supernatant was collected as the cytoplasmic fraction, taking care not to disturb the pellet. The pellet was resuspended in 300 μL Buffer S1 (250 mM sucrose, 10 mM MgCl_2_, 1× protease inhibitor, 40 U/mL Murine RNase inhibitor) and overlaid onto a 300 μL cushion of Buffer S2 (350 mM sucrose, 0.5 mM MgCl_2_, 1× protease inhibitor, 40 U/mL Murine RNase inhibitor), followed by centrifugation at 3000 × *g* for 10 min at 4 °C. The supernatant was discarded, and the pellet (nuclei) was resuspended in 100 µL 1× RIPA Buffer (50 mM Tris, pH 7.4, 150 mM NaCl, 1% Triton X-100, 0.5% sodium deoxycholate, 0.1% SDS, 1 × protease inhibitor, 40 U/mL Murine RNase inhibitor) and incubated on ice for 20 min. The mixture was centrifuged at 15,500 × *g* for 5 min at 4 °C. The supernatant was collected as the nuclear fraction, and the pellet was discarded. From the cytoplasmic fraction, 200 µL was used to validate NAT10 expression by western blot, while 100 µL was mixed with TRIzol Reagent (ThermoFisher Scientific) to isolate cytoplasmic RNA, followed by removal of contaminating genomic DNA using DNase I (NEB). All steps were performed on ice, and all centrifugation steps were carried out at 4 °C unless otherwise specified.

### Detection of m¹acp³Ψ by HinFI digestion

The presence of m¹acp³Ψ was assessed by reverse transcription using an 18S rRNA-specific primer, followed by PCR amplification with 18S-specific primers and subsequent digestion of the amplicon with HinFI. Briefly, 0.5 µg RNA from cytoplasmic fractions or 0.1 µg RNA from sucrose density gradient fractions was mixed with 1 µL of 10 µM 18S RNA-specific antisense primer (DAP-704, Supplementary Data 7) and 0.5 mM dNTPs, then incubated at 65 °C for 5 min. The mixture was immediately placed on ice for 1 min, then 0.5 µL Superscript III (ThermoFisher Scientific) and 0.5 µL Murine RNase inhibitor (NEB) were added, and the reaction was incubated at 55 °C for 1 h, followed by heat inactivation at 75 °C for 15 min. cDNA was diluted 1:10 with nuclease-free H_2_O, and 2 μL of diluted cDNA was mixed with 0.5 µM each of primers DAP-703 and DAP-704 and Q5 High-Fidelity DNA Polymerase (NEB). Amplicons (5 µL) were digested with 10 U HinFI in 1× rCutSmart buffer (NEB) at 25 °C for 5 s, 37 °C for 90 min, and 80 °C for 20 min. Digestion products were resolved on a 2.5% agarose gel and visualized with 1× SYBR Safe DNA Gel Stain (ThermoFisher Scientific). Densitometry of DNA bands was performed using ImageJ. The relative unmodified fraction (m¹acp³Ψ-negative) was calculated as unmod._band_/(m¹acp³Ψ_band_ + unmod_band_).

### RNA immunoprecipitation and RT-qPCR

NAT10^FKBP12-F36V^ Hep3B cells with TSR3 knockout were seeded at a density of 2.0 × 10⁶ cells in a 10 cm dish prior to transfection. Cells were co-transfected using Lipofectamine™ 2000 Transfection Reagent (Thermofisher Scientific) and plasmids encoding HA-tagged TSR3 together with one of the following FLAG-tagged NAT10 constructs: wild-type NAT10^WT^, helicase-deficient NAT10 mutants, or FLAG-stop (empty) control plasmid (See Supplementary Data 7 for plasmid list). After transfection, cells were incubated for 24 h to allow for protein expression. Transfected cells were then treated with 100 nM dTAG^V^-1 for 48 h. Before harvesting, cells were crosslinked with 0.1% formaldehyde, then incubated on ice for 5 min. Crosslinked cells were quenched with 75 mM glycine and incubated for 5 min on ice. Following two washes of cells with 1× ice-cold PBS, 1 mL of lysis buffer (20 mM Tris pH 7.5, 150 mM NaCl, 5 mM MgCl_2_, 1% Triton X−100, 100 µg/mL cycloheximide, 1 mM DTT) was added to the dish, and the cells were scraped into a fresh 1.5 mL Eppendorf tube. The lysate was cleared at 15,500 × *g* for 10 min at 4 °C. The cleared lysate was treated with 5 µL Turbo DNase and 10 µL RNase I (1:25 dilution in iced cold 1 × PBS), then incubated at 37 °C for 5 min. Digested lysate was immediately placed on ice and treated with 11 µL Murine inhibitor RNase (NEB). Following protein quantification, 25 µg of the sample was stored as input, while 500 µg was used for immunoprecipitation overnight at 4 °C using anti-HA antibodies (Cell Signaling, Cat#:3724S) or isogenic IgG as a control (Cell Signaling, Cat#:3724S) loaded onto magnetic Protein G beads (NEB). Next, beads were separated on magnetic rack and subsequently washed four times with 1 mL Wash buffer (20 mM Tris pH 7.5, 300 mM NaCl, 2 mM MgCl_2_, 1% Triton X-100, 1× protease inhibitor cocktail) followed by another four washes in 1 mL high salt wash buffer (20 mM Tris pH 7.5, 500 mM NaCl, 2 mM MgCl_2_, 1% Triton X-100, 1× protease inhibitor cocktail). After the last wash, all traces of wash were magnetically removed from beads, followed by resuspension in 100 uL of decrosslinking buffer (50 mM Tris-Cl, pH 7.0, 5 mM EDTA, 10 mM dithiothreitol (DTT), 1% SDS, 1 µl of murine RNase inhibitor) and incubated at 70 °C for 45 min to reverse crosslink. After decrosslinking, samples were separated on a magnet. 25 µL of the sample was used for western blotting, while 75 µL was used to isolate RNA using the Trizol method. 18S rRNA levels were determined by RT-qPCR using primers DAP-827 and DAP-828 (Supplementary Data 7).

### Co-immunoprecipitation

NAT10^FKBP12-F36V^ Hep3B cells stably transduced with either NAT10^WT^ or YFP control [NAT10(–) were seeded at a density of 2.0 × 10⁶ cells. Cells were then transfected with a plasmid encoding HA-tagged TSR3 using Lipofectamine™ 2000 Transfection Reagent (Thermofisher Scientific). After a 24 h incubation, transfected cells were treated with 100 nM dTAG^V^-1 for 48 h. Cell pellets were lysed in 1 mL lysis buffer (20 mM Tris, pH 7.5, 150 mM NaCl, 2 mM MgCl_2_, 1% Triton X-100, 1× protease inhibitor cocktail) and incubated at 4 °C for 20 min with rotation. Lysate was centrifuged at 15,500 × *g* for 10 min at 4 °C. After protein quantification, 25 µg of the sample was reserved as input, and 1 mg of protein was split into two aliquots for HA-tag and IgG immunoprecipitations. Prewashed Magnetic Protein G beads (NEB) were transferred into separate tubes and incubated with either anti-HA-tag or Isogenic IgG antibodies on a rotator for 1 hr. 500 µg of sample was added to antibody-coupled beads and incubated overnight at 4 °C with rotation. Beads were washed four times with 1 mL wash buffer (20 mM Tris pH 7.5, 300 mM NaCl, 2 mM MgCl_2_, 1% Triton X-100, 1× protease inhibitor cocktail) and another four washes in 1 mL high salt wash buffer (20 mM Tris pH 7.5, 500 mM NaCl, 2 mM MgCl_2_, 1% Triton X-100, 1× protease inhibitor cocktail). After the last wash, all traces of wash buffer were removed, and the beads were resuspended in 1× Laemmli buffer and incubated at 95 °C for 5 min to disrupt the immunocomplexes. Beads were separated on a magnet, and eluent was used for western blotting using anti-HA tag goat polyclonal antibody (1:5,000; Fortis Life Sciences, Cat# A190−138A), and anti-NAT10 polyclonal antibody (1:2,500; Proteintech, Cat# 13365-1-AP), followed by HRP-linked anti-rabbit IgG secondary antibody (1:5,000; Cell Signaling Technology, Cat# 7074) to assess the interaction between TSR3 and NAT10.

### Primer extension assay

Primer extension was performed using AMV reverse transcriptase (Promega). 1 µg of RNA was mixed with 1 pmol of IRD-800-labelled primer (DAP-775, Supplementary Data 7) and incubated at 65 °C for 5 min and then placed on ice for 1 min. RT reaction components were added to the mixture according to the manufacturer’s protocol, and the mixture was incubated at 42 °C for 1 h. The reaction product was separated on 12.5% TBU-gel at 300 V in 4 °C. Gel was visualized using a Sapphire multimode imager (Azure Biosystems).

### eCLIP-seq

The eCLIP-seq protocol was performed as described in Blue et al. ^76^. with minor modifications. eCLIP-seq was performed in wild-type (*n* = 3), NAT10^(–)^ (*n* = 2), NAT10^WT^ (*n* = 3), NAT10^E389L^ (*n* = 3), and NAT10^K290V^ (*n* = 3) Hep3B cells. From each condition, two fractions, Input and CLIP, were sequenced. Briefly, cell were crosslinked at 400,000 µJ/cm2 UV_254nm_ (Fisherbrand™ UV Crosslinker), harvested, and lysed in cold lysis buffer [50 mM Tris-HCl pH 7.4, 100 mM NaCl, 1% (vol/vol), Igepal CA-630, 0.1% (vol/vol) SDS and 0.5% (wt/vol) sodium deoxycholate, and 5.5 µL 200× protease inhibitor cocktail III], followed by sonication in a Bioruptor (30 s on/30 s off at 4 °C for 5 min). Lysates were treated with Turbo DNase I and RNase I, clarified by centrifugation, and incubated overnight at 4 °C with anti-NAT10 antibodies (Proteintech Cat. #:13365-1-AP) pre-bound to M-280 sheep anti-rabbit IgG magnetic beads (ThermoFisher Scientific). After overnight incubation, input and supernatant fractions were collected. Beads were washed twice High-Salt Wash Buffer [50 mM Tris-HCl pH 7.4, 1 M NaCl, 1% (vol/vol) Igepal CA-630, 1 mM EDTA, 0.1% (vol/vol) SDS and 0.5% (wt/vol) sodium deoxycholate] and three times with 500 µL Wash Buffer [20 mM Tris-HCl pH 7.4, 10 mM MgCl_2_, 0.2% (vol/vol) Tween-20 and 5 mM NaCl]. RNA ends were dephosphorylated (5 µL 10× FastAP Buffer, 2 µL murine RNase inhibitor, 2 µL Turbo DNase, 3 µL FastAP enzyme, 38 µL nuclease-free water) and end-repaired [20 µL 10× PNK Buffer, 4 µL PNK enzyme (10000 U/mL), 126 µL nuclease-free water] at 37 °C, 1,200 rpm for 20 min in Thermomixer.

The first adapter ligation was performed on beads for the immunoprecipitated fraction and in solution for the Input fraction, using 1X RNA ligase buffer, 70 U T4 RNA ligase (high-concentration enzyme), and 100 pmol RNA adapter (DAP-610, Supplementary Data 7), for 75 min at room temperature. Complexes were separated in two parallel 4-12% Bis-Tris gels. One gel served as a diagnostic control, and the other gel was used for RNA isolation. Gels were transferred overnight to nitrocellulose membranes. Western blot was peformed using mouse anti-NAT10 primary antibodies (1:1000 in 5% milk + 0.05% TBST, Santa Cruz, sc-271770), HRP-conjugated secondary antibody (anti-mouse, 1:5000 in 5% milk + 0.05% TBST, Cell Signaling Technology, #7076), and visualized by enhanced chemiluminescence using the ECL Western Blotting Substrate (Promega) on a Sapphire Biomolecular Imager (Azure Biosystems).

Using the Western blot as a guide, a region of the RNA membrane starting from the observed size of NAT10 and extending to ~75 kDa larger was cut with a clean razor blade, diced into squares, and transferred to cold 1.5 mL containing 150 μL of Proteinase K master mix [100 mM Tris-HCl pH 7.4, 50 mM NaCl, 10 mM EDTA, and 0.2% (vol/vol) SDS, 30 μL Proteinase K (0.8 U/μl)]. RNA was purified using the RNA Clean and Concentrator Kit (Zymo Research), and reverse transcribed using Maxima H minus (ThermoFisher Scientific) and primer DAP-611 (Supplementary Data 7) at 55 °C for 20 min.

A 3’ cDNA adapter containing a UMI of 10 randomized nucleotides (DAP-612, Supplementary Data 7) was ligated using 1X RNA ligase buffer and 30 U T4 RNA ligase high-concentration enzyme. Final libraries were generated by PCR using Q5® High-Fidelity 2X Master Mix (NEB) and NEBNext® Multiplex Oligos for Illumina® (NEB), gel-purified, and verified on a TapeStation 4200 System (Agilent). Quantified libraries were pooled and sequenced on an Illumina NovaSeq X Plus system with 150-bp paired-end reads.

Raw reads were pre-processed to remove low-quality bases, adapter sequences, and a stretch of at least 10 consecutive guanine (G) nucleotides at the 3’ end using cutadapt/v4.2^77^. FastQC/v0.11.5 was used as a quality control check. The unique molecular identifier (UMI) was extracted using UMI-tools/v1.1.5^78^. Reads were aligned to the human genome (hg38) using STAR/v2.7.9a in local alignment mode^79^. Separately, reads were aligned to a small genome containing rRNA and snoRNA sequences using Bowtie2/v2.5.4^80^. All alignment files were sorted and indexed using samtools/v1.6^72^. All alignments were deduplicated using UMI-tools/v1.1.5. These alignments were converted to bigwig files normalized by RPKMs using Deeptools/v3.5.1^81^.

BigWig files were then processed in R/v4.4.0 using the rtracklayer and GenomicRanges packages. Signal was extracted across a defined region for 18S rRNA (GL000220.1, positions 109,078–110,946). Input-normalized signal tracks were generated by subtracting matched input controls from three independent biological replicates. Each track was subsequently scaled to its maximum value, resulting in normalized signals ranging from 0 to 1. Replicates were grouped by experimental condition, and the mean was calculated at each position, smoothed, and visualized using ggplot2.

For analysis of an RT-stop at m¹acp³Ψ in 18S rRNA, RPKM signal was extracted from BigWig files with a 10-nucleotide window (GL000220.1, positions 110,315–110,335). For each genotype, RPKM values were aggregated across three biological replicates, and the mean signal was plotted at each genomic position using ggplot2. To compare RPKM distributions between genotypes, two-sample Kolmogorov–Smirnov (KS) tests were performed between each genotype relative to NAT10^WT^. To calculate the relative unmodified fraction, two subregions were defined: upstream of m¹acp³Ψ (GL000220.1, positions 110,315–110,324) and downstream of m¹acp³Ψ (GL000220.1, 110,326–110,335), and the relative unmodified fractions were calculated as upstream/(upstream + downstream) for each replicate. The relative signal percentages were compared between groups using a Welch’s *t* test (unequal variances).

### Nanopore direct RNA sequencing (DRS)

Direct RNA sequencing was performed according to the manufacturer’s protocol (Oxford Nanopore, SQK-RNA004) in two biological replicates from wild-type, NAT10(–), NAT10^E289L^, and NAT10^K290A^ Hep3B cells. Briefly, 1 µg of total RNA was ligated to an 18S rRNA reverse transcription adapter (DAP-660 and DAP-661, Supplementary Data 7) using T4 DNA Ligase (NEB) at room temperature for 10 min. The ligated RNA was reverse transcribed using Induro^®^ Reverse Transcriptase (NEB) at 60 °C for 30 min, followed by enzyme inactivation at 70 °C for 10 min. The reaction mixture was purified using 1.8× volume of CleanNGS beads (CleanNA) according to the manufacturer’s protocol. The reverse-transcribed RNA was then ligated to the RNA ligation adapter (RLA) from the Direct RNA Sequencing Kit (Oxford Nanopore, SQK-RNA004) at room temperature for 10 min, followed by purification with a 0.4× volume of CleanNGS beads (CleanNA). A total of 12 μL of RNA library was mixed with 37.5 µL sequencing buffer and 25.5 µL library solution, yielding a final volume of 75 µL. The RNA library was loaded onto a FLO-MIN004RA flow cell (Oxford Nanopore) and sequenced on a MinION Mk1B device. Each sample was sequenced independently in biological duplicates.

Base calling was performed using Dorado (v0.8.1) with the rna004_130bps_sup@v5.1.0 model. Reads were aligned to the RNA45SN5 reference (NT_167214.1) using graphmap/v0.5.2^59^ with the following parameters: --mapq -1 -x sensitive -z 1 -K fastq --min-read-len 0 -A 6 -k 5. For re-squiggling, POD5 files were converted to BLOW5 format using Blue-crab/v0.2.0. NanoCEM/v 0.0.6.1^60^ was then run with the following settings: current_events_magnifier f5c_re --rna --norm --pore rna004 for every annotated modified position in 18S rRNA. This script extracts the dwell time, median current intensity, mean current intensity, and standard deviation of current intensity within a ± 4 nt window surrounding each modified position for every individual read. Statistical differences in dwell time and current intensity at each position between wild-type (NAT10^WT^), NAT10(–), NAT10^E289L^, and NAT10^K290A^ were assessed using the Wilcoxon rank-sum test. 18S rRNA modification positions with significant differences within a ± 4 nt window were extracted. Only positions reproducible across all comparisons (NAT10(–) vs. NAT10^WT^, NAT10^E289L^ vs. NAT10^WT^, and NAT10^K290A^ vs. NAT10^WT^) were selected as confident modification sites.

### Mapping the ends of rRNA and sequencing (MErR-seq)

MErR-seq was performed in two biological replicates from wild-type and NAT10^E289L^ Hep3B cells. Total RNA was dephosphorylated using FastAP Thermosensitive Alkaline Phosphatase (ThermoFisher Scientific) and T4 Polynucleotide Kinase (NEB). The 3′ ends of dephosphorylated RNA were ligated to a 3′ adapter (DAP-410, Supplementary Data 7) using 1 µL T4 RNA ligase 2, Truncated KQ (NEB), 0.5 µL Murine RNase Inhibitor (NEB), and 7 µL PEG 8000 (50%, NEB) followed by incubation at room temperature for 3 h with gentle mixing every 30 min. Next, 0.5 µL 5´-deadenylase (NEB) and 0.5 µL RecJ exonuclease (Biosearch) were added directly to the ligation mixture and incubated at 45 °C for 30 min to degrade unligated adapter. The 3′ adapter contained a spacer to prevent self-ligation of RNA. The ligated RNA was immediately purified using the RNA Clean & Concentrator Kit (Zymo Research) according to the manufacturer’s protocol. The purified RNA was then 5′ phosphorylated using T4 Polynucleotide Kinase (NEB), incubated at 37 °C for 30 min, and purified using 1.8× CleanNGS beads (CleanNA) according to the manufacturer’s protocol. The phosphorylated RNA was ligated to a 5′ RNA adapter (DAP-697, Supplementary Data 7) containing a unique molecular identifier (UMI) of ten random nucleotides. Ligation was performed using 1.8 µL T4 RNA Ligase 1 (ssRNA Ligase), High Concentration (NEB), and incubated at room temperature for 2 h on a hula mixer. Ligated RNA was purified with 15 µL MyONE silane beads and recovered in 40 µL nuclease-free water.

Five microliters of NEBNext^®^ Magnesium RNA Fragmentation buffer were added to 40 µL of 5′-ligated RNA, and the mixture was incubated at 94 °C for 5 min, then placed on ice for 1 min. Next, 5 µL NEBNext RNA Fragmentation Stop Solution (NEB) was added. The fragmented RNA was purified using 1.8× CleanNGS beads (CleanNA) according to the manufacturer’s protocol and eluted in nuclease-free water. To capture the 5′ end, fragmented RNA was dephosphorylated by adding 3 µL FastAP (Thermofisher Scientific), 3 µL T4 Polynucleotide Kinase (NEB), and 1 µL Murine RNase Inhibitor (NEB), followed by incubation at 37 °C for 30 min. The dephosphorylated RNA was ligated to a 3′ RNA adapter (DAP-610, Supplementary Data 7) using 1.2 µL T4 RNA Ligase 1 (ssRNA Ligase), High Concentration (NEB), followed by incubation at room temperature for 1 h. Ligated RNA was purified using 15 µL MyONE Silane beads and eluted in 20 µL nuclease-free water, then further purified with 1.8× CleanNGS beads (CleanNA) and eluted in 8 µL nuclease-free water. The 5′ end was reverse transcribed by adding 0.6 µL SuperScript III (ThermoFisher Scientific) and a 3′-specific adapter primer (DAP-611, Supplementary Data 7) in a total reaction volume of 20 µL, followed by incubation at 55 °C for 20 min. Excess primers were removed by adding 3 µL ExoSAP-IT (ThermoFisher Scientific) and incubating at 37 °C for 12 min, followed by enzyme inactivation at 85 °C for 5 min. The mixture was cleaned with 1.8× AMPure XP beads (Beckman Coulter) and eluted in 21 µL nuclease-free water. cDNA was PCR-amplified using Q5® High-Fidelity 2X Master Mix (NEB) and NEBNext® Multiplex Oligos for Illumina®, with each sample assigned a unique indexed primer. Thermocycling conditions were as follows: initial denaturation at 98 °C for 30 s; six cycles of 98 °C for 15 s, 68 °C for 30 s, and 72 °C for 40 s; eight cycles of 98 °C for 15 s and 72 °C for 60 s; and a final extension at 72 °C for 1 min. PCR products were purified with 0.9× AMPure XP beads (Beckman Coulter) and eluted in 12 µL nuclease-free water. One microliter from each library was quantified using the Qubit™ dsDNA Quantification Assay Kit (ThermoFisher Scientific). Library quality was assessed using High Sensitivity D5000 reagents and ScreenTape (Agilent). Quantified libraries were pooled and sequenced on an Illumina NovaSeq X Plus system with 150-bp paired-end reads.

Raw reads were pre-processed using Cutadapt (v4.2) to remove low-quality bases, adapter sequences from both 3′ adapter ligation steps, and stretches of at least 10 consecutive guanine (G) nucleotides at the 3′ end^77^. This step was necessary because the sequencing instrument occasionally reported high-quality Gs in the absence of signal at the end of reads. Reads were aligned to the RNA45SN5 reference (NT_167214.1), which was split into separate reference sequences corresponding to each cleavage site (5ETS, A0, A1, 18S, E, A2, B1, 5_8S, ITS2, 28S, 3ETS) using Bowtie2 (v2.5.4)^80^. All alignment files were sorted and indexed using samtools (v1.6)^72^. The first-in-pair (R1) reads were extracted using samtools to obtain sequences containing 5′-end information. Alignments were converted to BigWig files and normalized by reads per kilobase per million mapped reads (RPKM) using deepTools (v3.5.1)^81^. BigWig files were visualized using Integrative Genomics Viewer (IGV, v2.19.1)^82^. RPKM values within a 50-nt window from the start site of each cleavage site (5ETS, A0, A1, 18S, E, A2, B1, 5_8S, ITS2, 28S, 3ETS) were extracted from BigWig files using the rtracklayer package in R. The mean RPKM values within each 50-nt window were calculated and compared across samples.

### Mapping of ac^4^C

ac^4^C sites were mapped using a combination of RetraC:T-seq and ac^4^C-seq^31,51^ in two biological replicates from NAT10(–), NAT10^WT^, NAT10^E389L^, NAT10^K290A^, NAT10^H537A^, and NAT10^G641E^ Hep3B cells. From each condition, two fractions, mock-treated and NaCNBH_3_, were sequenced. Total RNA was partially depleted of ribosomal RNA using the NEBNext® rRNA Depletion Kit v2 (New England Biolabs), with antisense DNA probes reduced to 50% of the manufacturer’s recommended amount. RNA was then purified using the Zymo Clean & Concentrator kit in place of SPRI bead cleanup. This modified protocol enabled retention of substantial amounts of rRNA and tRNA, facilitating detection of ac^4^C in these molecules. RNA was then fragmented, dephosphorylated, and ligated to a 3’ pre-adenylated DNA adapter (DAP-756, Supplementary Data 7). After ligation, each sample was split into two. One fraction was incubated with 100 mM NaCNBH_3_ in a 100 mM HCl solution for 5 min at room temperature. The other fraction was mock-treated in a 100 mM HCl solution for 5 min at room temperature. Reactions were neutralized, reprecipitated using the sodium acetate method, and cleaned up using the Zymo Clean and Concentrator columns. RNA was then reverse transcribed using the Induro reverse transcriptase (New England Biolabs) and an adapter-specific antisense primer (DAP-611, Supplementary Data 7). cDNA was purified using MyOne Silane beads and ligated to a 3’ cDNA adapter containing a 10-nucleotide unique molecular identifier (DAP-612, Supplementary Data 7). Ligated cDNA was purified using MyOne Silane beads and the final library was amplified using Q5® High-Fidelity 2X Master Mix (NEB) and NEBNext® Multiplex Oligos for Illumina®, with each sample assigned a unique indexed primer. Quantified libraries were pooled and sequenced on an Illumina NovaSeq X Plus system with 150-bp paired-end reads.

Raw reads were pre-processed by removing adapters, low-quality bases, and stretches of at least 10 consecutive guanine (G) nucleotides at the 3’ end with Cutadapt (v4.2). The 10-nucleotide UMI was extracted with umi-tools (v1.1.6). Processed reads were aligned to the human genome (GRCh38.110), sorted and indexed by samtools (v1.6), and deduplicated with umi-tools (v1.1.6). Samtools mpileup was used to generate a read pileup at each genomic position, and read counts were extracted using (https://github.com/IARCbioinfo/mpileup2readcounts) (v1.0.0). A Python (v2.7.13) script was used to append strand information to each position. Per position, the non-reference nucleotide with the highest mismatch frequency was selected as the dominant mismatch. We require positions to have at least 30 reads; the mismatch nucleotide must have at least 5 reads; and the mismatch frequency must be above 1% (high-confidence sites) or 0.1% (low-confidence sites). A Fisher’s Exact Test with Benjamini-Hochberg correction identified significantly enriched mismatches in mock-treated and NaCNBH_3_-treated samples relative to each other in the wild-type Hep3B cells and NAT10^FKBP12-F36V^ cells reconstituted with NAT10^WT^ (Supplementary Fig. 9c, d). The selected sites must be significant in both biological replicates. Next, we selected sites that were significantly decreased in NAT10(–) conditions relative to wild-type Hep3B cells and to NAT10^FKBP12-F36V^ cells reconstituted with NAT10^WT^, when treated with NaCNBH_3_. The selected sites must be significant in both replicates. Using the sites that significantly decreased under NAT10(–) conditions, we determined their relative changes in each genotype compared with NAT10^WT^ when treated with NaCNBH_3_. A Wilcoxon test with Benjamini-Hochberg correction was performed between all genotype groups for overall reduction in ac^4^C levels, and a Fisher’s Exact Test with Benjamini-Hochberg correction for individual sites.

Genomic coordinates corresponding to significant ac^4^C sites were processed using a custom Python pipeline to sequentially intersect with multiple reference annotations using BEDTools/v2.30.0 intersect in strand-specific mode. Sites were first mapped to tRNA annotations, and unmapped sites were subsequently intersected with RepeatMasker annotations. The remaining unmapped sites were then intersected with gene annotations from the human genome (GRCh38, Ensembl v110). The final results are reported in Supplementary Data 5. Because rRNA and tRNA genes have multiple copies in the genome, known sites at positions 1337 and 1842 of 18S rRNA, and at position 12 of tRNA^Leu^ and tRNA^Ser,^ appear several times in Supplementary Data 5.

### Structural modeling

A structural model of NAT10 bound to ATP was obtained from AlphaFold3 (https://alphafoldserver.com) and imported into PyMol (v3.1.1) for processing and visualization. The N-terminal region (residues 1–106) and the GNAT, tRBD, and C-terminal regions (residues 489–1025) of NAT10 were hidden in all representations. The DUF domain (residues 107–220) was colored light gray, and the helicase domain (residues 221–488) was colored dark gray. Key residues (R286, K290, S291, D388, E389, and T412) identified as significant in DMS-Nseq were displayed as red spheres. ATP was shown as blue spheres. A 360-frame animation was generated by rotating the model one degree per frame along the y-axis. For Supplementary Fig. 4c, residues L114, T117, and G143 were displayed as red spheres.

To visualize the interaction of ATP with crucial NAT10 residues, AlphaFold3 models of mutated NAT10 in complex with ATP were loaded into PyMol, with residues K290–S291 and D388–E389 highlighted in stick representation and color-coded by atom type, while ATP was shown in yellow. The view was centered on the ATP-binding site, and potential hydrogen bond interactions within 3.5 Å between ATP and the selected residues were assessed using PyMOL distance measurements. For Supplementary Fig. 4d, residues L114, T117, and G143 were highlighted in stick representation and color-coded by atom type.

For visualization of NAT10 binding to pre-40S, the cryo-EM model of the human pre-40S ribosomal subunit was downloaded from the PDB (ID: 6ZXH)^83^ and loaded into PyMOL (v3.1.1). The 18S rRNA chain was displayed with m¹acp³Ψ at position 1248, and ac⁴C at positions 1337 and 1842 indicated as spheres. The binding regions of NAT10 were defined from the eCLIP-seq data, including residues 1100–1240 (major peak 1) and residues 1620–1850 (major peak 2).

### Statistical analysis and reproducibility

Statistical analyses for xenograft experiments and primary ex-vivo cell experiments were performed using GraphPad Prism (v10). All other statistical analyses and plotting were performed in R (v4.4.0) and included in the Data Source File. In summary, two-group comparisons were performed using Student’s *t* tests when variances were equal and Welch’s *t* tests when variances were unequal. For non-parametric two-group comparisons, Mann–Whitney U tests or Wilcoxon signed-rank tests were applied. The Kolmogorov–Smirnov test was used to compare distributions between genotypes. For multiple-group comparisons, one-way ANOVA was used for normally distributed data with equal variances, Welch’s ANOVA was applied when variances were unequal, and the Kruskal–Wallis test was used for non-parametric data. Survival curves were compared using the Log-rank test. The specific statistical tests, post hoc corrections for multiple analyses, whether one-sided or two-sided, graph types, variables, sample sizes (n), and *p* values are indicated in the figures and their legends. No statistical method was used to predetermine sample size. For in vivo experiments, the sample sizes were chosen based on previous studies^84^, and the numbers were chosen to balance statistical power with practical and ethical considerations (3 R). For in vivo experiments, animals were excluded from the study due to unrelated conditions, e.g., kinked tail or severe fight wounds. Age and sex matched littermates were used within the animal experiments. Researchers were not blinded. However, for animal experiments, the technicians who provided the majority of animal care and monitored disease progression were blinded to the group allocation. For the cord blood experiment (Fig. S7a), sex ratios were balanced with 3 cord bloods from 2 female donors and 1 from a male donor. The Investigators were not blinded to any other experiment.

### Reporting summary

Further information on research design is available in the Nature Portfolio Reporting Summary linked to this article.

## Supplementary information

Supplementary Information

Description of Additional Supplementary Information

Supplementary Movie 1

Supplementary Data 1

Supplementary Data 2

Supplementary Data 3

Supplementary Data 4

Supplementary Data 5

Supplementary Data 6

Supplementary Data 7

Reporting Summary

Transparent Peer Review file

## Source data

Source Data

## Data Availability

The transcriptomics and deep sequencing data generated in this study have been deposited in the Gene Expression Omnibus (GEO) under accession codes GSE291931 (DMS-Nseq), GSE291932 (Nanopore direct RNA sequencing), GSE291928 (MErR-seq), GSE291934 (eCLIP-seq), and GSE325346 (ac^4^C mapping) and are publicly available. All other data supporting the findings of this study are provided in the Supplementary Figures, the Supplementary Data, and the Source Data file. Source data are provided with this paper.

## Code availability

Analysis scripts are available on github.com/arangolab and Zenodo under DOIs https://doi.org/10.5281/zenodo.20799580^85^, https://doi.org/10.5281/zenodo.20784953^86^, https://doi.org/10.5281/zenodo.20799456^87^, https://doi.org/10.5281/zenodo.21026515^88^, and https://doi.org/10.5281/zenodo.20801474^89^.

## References

[1] Khatter, H., Myasnikov, A. G., Natchiar, S. K. & Klaholz, B. P. Structure of the human 80S ribosome. *Nature* **520**, 640–645 (2015). 25901680 10.1038/nature14427

[2] Genuth, N. R. & Barna, M. Heterogeneity and specialized functions of translation machinery: from genes to organisms. *Nat. Rev. Genet* **19**, 431–452 (2018). 29725087 10.1038/s41576-018-0008-z PMC6813789

[3] Milenkovic, I. & Novoa, E. M. Dynamic rRNA modifications as a source of ribosome heterogeneity. *Trends Cell Biol.* **35**, 604–614 (2025). 39500673 10.1016/j.tcb.2024.10.001

[4] Babaian, A. et al. Loss of m¹acp³Ψ ribosomal RNA modification is a major feature of cancer. *Cell Rep.* **31**, 107611 (2020). 32375039 10.1016/j.celrep.2020.107611

[5] Huang, H., Parker, M. & Karbstein, K. The modifying enzyme Tsr3 establishes the hierarchy of Rio kinase binding in 40S ribosome assembly. *RNA* **28**, 568–582 (2022). 35031584 10.1261/rna.078994.121 PMC8925970

[6] Meyer, B. et al. Ribosome biogenesis factor Tsr3 is the aminocarboxypropyl transferase responsible for 18S rRNA hypermodification in yeast and humans. *Nucleic Acids Res*. **44**, 4304–4316 (2016). 27084949 10.1093/nar/gkw244 PMC4872110

[7] Cheng, Y. et al. A non-canonical role for a small nucleolar RNA in ribosome biogenesis and senescence. *Cell* **187**, 4770–4789.e4723 (2024). 38981482 10.1016/j.cell.2024.06.019 PMC11344685

[8] Dalhat, M. H., Narayan, S., Serio, H. & Arango, D. Dissecting the oncogenic properties of essential RNA-modifying enzymes: a focus on NAT10. *Oncogene* **43**, 1077–1086 (2024). 38409550 10.1038/s41388-024-02975-9 PMC11092965

[9] Shen, Q. et al. NAT10, a nucleolar protein, localizes to the midbody and regulates cytokinesis and acetylation of microtubules. *Exp. Cell Res*. **315**, 1653–1667 (2009). 19303003 10.1016/j.yexcr.2009.03.007

[10] Tan, Y. et al. Loss of nucleolar localization of NAT10 promotes cell migration and invasion in hepatocellular carcinoma. *Biochem Biophys. Res. Commun.* **499**, 1032–1038 (2018). 29634924 10.1016/j.bbrc.2018.04.047

[11] Tschida, B. R. et al. Sleeping beauty insertional mutagenesis in mice identifies drivers of steatosis-associated hepatic tumors. *Cancer Res*. **77**, 6576–6588 (2017). 28993411 10.1158/0008-5472.CAN-17-2281 PMC5712258

[12] Zhang, S. et al. NAT10-mediated mRNA N(4)-acetylcytidine reprograms serine metabolism to drive leukaemogenesis and stemness in acute myeloid leukaemia. *Nat. Cell Biol.* **26**, 2168–2182 (2024). 39506072 10.1038/s41556-024-01548-y PMC11628400

[13] Liao, L. et al. Lysine 2-hydroxyisobutyrylation of NAT10 promotes cancer metastasis in an ac4C-dependent manner. *Cell Res*. **33**, 355–371 (2023). 36882514 10.1038/s41422-023-00793-4 PMC10156899

[14] Zhang, Y. et al. NAT10 promotes gastric cancer metastasis via N4-acetylated COL5A1. *Signal Transduct. Target Ther.* **6**, 173 (2021). 33941767 10.1038/s41392-021-00489-4 PMC8093205

[15] Xie, R. et al. NAT10 drives cisplatin chemoresistance by enhancing ac4C-associated DNA repair in bladder cancer. *Cancer Res*. **83**, 1666–1683 (2023). 36939377 10.1158/0008-5472.CAN-22-2233

[16] Amin, R. et al. NAT10 promotes cancer metastasis by modulating p300/CBP activity through chromatin-associated tRNA. *Mol. Cell* **85**, 4526–4544.e4518 (2025). 41344331 10.1016/j.molcel.2025.11.010 PMC12709618

[17] Huang, L. et al. N(4)-acetylcytidine modification of ITGB5 mRNA mediated by NAT10 promotes perineural invasion in pancreatic ductal adenocarcinoma. *J. Exp. Clin. Cancer Res*. **44**, 103 (2025). 40119353 10.1186/s13046-025-03362-2 PMC11929334

[18] Chen, J. F. et al. An in vivo screen identifies NAT10 as a master regulator of brain metastasis. *Sci. Adv.* **11**, eads6021 (2025). 40138393 10.1126/sciadv.ads6021 PMC11939035

[19] Ito, S. et al. Human NAT10 is an ATP-dependent RNA acetyltransferase responsible for N4-acetylcytidine formation in 18 S ribosomal RNA (rRNA). *J. Biol. Chem.* **289**, 35724–35730 (2014). 25411247 10.1074/jbc.C114.602698 PMC4276842

[20] Sharma, S. et al. Yeast Kre33 and human NAT10 are conserved 18S rRNA cytosine acetyltransferases that modify tRNAs assisted by the adaptor Tan1/THUMPD1. *Nucleic Acids Res*. **43**, 2242–2258 (2015). 25653167 10.1093/nar/gkv075 PMC4344512

[21] Arango, D. et al. Acetylation of cytidine in mRNA promotes translation efficiency. *Cell* **175**, 1872–1886.e1824 (2018). 30449621 10.1016/j.cell.2018.10.030 PMC6295233

[22] Deng, M. et al. Helicobacter pylori-induced NAT10 stabilizes MDM2 mRNA via RNA acetylation to facilitate gastric cancer progression. *J. Exp. Clin. Cancer Res*. **42**, 9 (2023). 36609449 10.1186/s13046-022-02586-w PMC9817303

[23] Wang, G. et al. NAT10-mediated mRNA N4-acetylcytidine modification promotes bladder cancer progression. *Clin. Transl. Med*. **12**, e738 (2022). 35522942 10.1002/ctm2.738 PMC9076013

[24] Liu, Y. et al. m(6)A-driven NAT10 translation facilitates fatty acid metabolic rewiring to suppress ferroptosis and promote ovarian tumorigenesis through enhancing ACOT7 mRNA acetylation. *Oncogene* **43**, 3498–3516 (2024). 39390256 10.1038/s41388-024-03185-z

[25] Liu, H. et al. Targeting N4-acetylcytidine suppresses hepatocellular carcinoma progression by repressing eEF2-mediated HMGB2 mRNA translation. *Cancer Commun. (Lond.)* **44**, 1018–1041 (2024). 39030964 10.1002/cac2.12595 PMC11492314

[26] Ding, M. et al. N-acetyltransferase 10 facilitates tumorigenesis of diffuse large B-cell lymphoma by regulating AMPK/mTOR signalling through N4-acetylcytidine modification of SLC30A9. *Clin. Transl. Med*. **14**, e1747 (2024). 38961519 10.1002/ctm2.1747 PMC11222071

[27] Wei, W. et al. NAT10-mediated ac4C tRNA modification promotes EGFR mRNA translation and gefitinib resistance in cancer. *Cell Rep.* **42**, 112810 (2023). 37463108 10.1016/j.celrep.2023.112810

[28] Li, H. et al. Mechanistic study of N-acetyltransferase 10 deficiency enhancing olaparib sensitivity in triple negative breast cancer by inhibiting RAD51 N4-acetylcytidine modification. *iScience* **28**, 112860 (2025). 40606759 10.1016/j.isci.2025.112860 PMC12221513

[29] Wang, Q. et al. Glucose homeostasis controls N-acetyltransferase 10-mediated ac4C modification of HK2 to drive gastric tumorigenesis. *Theranostics* **15**, 2428–2450 (2025). 39990211 10.7150/thno.104310 PMC11840738

[30] Arango, D. et al. Direct epitranscriptomic regulation of mammalian translation initiation through N4-acetylcytidine. *Mol. Cell* **82**, 2797–2814.e2711 (2022). 35679869 10.1016/j.molcel.2022.05.016 PMC9361928

[31] Sas-Chen, A. et al. Dynamic RNA acetylation revealed by quantitative cross-evolutionary mapping. *Nature* **583**, 638–643 (2020). 32555463 10.1038/s41586-020-2418-2 PMC8130014

[32] Sharma, S. et al. Specialized box C/D snoRNPs act as antisense guides to target RNA base acetylation. *PLoS Genet*. **13**, e1006804 (2017). 28542199 10.1371/journal.pgen.1006804 PMC5464676

[33] Bortolin-Cavaille, M. L. et al. Probing small ribosomal subunit RNA helix 45 acetylation across eukaryotic evolution. *Nucleic Acids Res*. **50**, 6284–6299 (2022). 35648437 10.1093/nar/gkac404 PMC9226516

[34] Thalalla Gamage, S. et al. Antisense pairing and SNORD13 structure guide RNA cytidine acetylation. *RNA* **28**, 1582–1596 (2022). 36127124 10.1261/rna.079254.122 PMC9670809

[35] Thalalla Gamage, S. et al. Transfer RNA acetylation regulates in vivo mammalian stress signaling. *Sci. Adv.* **11**, eads2923 (2025). 40106564 10.1126/sciadv.ads2923 PMC11922055

[36] Hafner, J. et al. Sni445 recruits box C/D snoRNPs snR4 and snR45 to guide ribosomal RNA acetylation by Kre33. *Nucleic Acids Res.* **54**, gkag030 (2026). 10.1093/nar/gkag030 PMC12848939 41603732

[37] Larrieu, D., Britton, S., Demir, M., Rodriguez, R. & Jackson, S. P. Chemical inhibition of NAT10 corrects defects of laminopathic cells. *Science* **344**, 527–532 (2014). 24786082 10.1126/science.1252651 PMC4246063

[38] Taoka, M. et al. RNA cytidine acetyltransferase of small-subunit ribosomal RNA: identification of acetylation sites and the responsible acetyltransferase in fission yeast, Schizosaccharomyces pombe. *PLoS One* **9**, e112156 (2014). 25402480 10.1371/journal.pone.0112156 PMC4234376

[39] Wang, H. et al. NAT10 triggers colorectal cancer progression via promoting PPAN-regulated DNA damage repair. *Oncogene* **45**, 558–576 (2026). 41455833 10.1038/s41388-025-03661-0

[40] Huang, F. et al. Nat10-mediated ac4C epitranscriptomics orchestrates hematopoietic stem/progenitor cell fate determination via translation control. *Sci. Adv.* **12**, eady0301 (2026). 41499488 10.1126/sciadv.ady0301 PMC12778038

[41] Yu, C. et al. NAT10 promotes vascular remodelling via mRNA ac4C acetylation. *Eur. Heart J.* **46**, 288–304 (2025). 39453784 10.1093/eurheartj/ehae707

[42] Shi, L. et al. NAT10 regulates heart development and function by maintaining the expression of genes related to fatty acid beta-oxidation and heart contraction. *Cell Death Differ.* **33**, 358–373 (2026). 40946112 10.1038/s41418-025-01577-6 PMC12881536

[43] Zhou, M. et al. Structure of the NAT10 acetyltransferase and mechanism of tRNA acetylation. *Nat. Commun*. **17**, 7763 (2026). 10.1038/s41467-026-74222-6 PMC13433802 42321159

[44] Li, Q. et al. NAT10 is upregulated in hepatocellular carcinoma and enhances mutant p53 activity. *BMC Cancer* **17**, 605 (2017). 28859621 10.1186/s12885-017-3570-4 PMC5579925

[45] Ma, R. et al. Up regulation of NAT10 promotes metastasis of hepatocellular carcinoma cells through epithelial-to-mesenchymal transition. *Am. J. Transl. Res*. **8**, 4215–4223 (2016). 27830005 PMC5095314

[46] Pan, Z. et al. Role of NAT10-mediated ac4C-modified HSP90AA1 RNA acetylation in ER stress-mediated metastasis and lenvatinib resistance in hepatocellular carcinoma. *Cell Death Discov.* **9**, 56 (2023). 36765042 10.1038/s41420-023-01355-8 PMC9918514

[47] Hietpas, R., Roscoe, B., Jiang, L. & Bolon, D. N. Fitness analyses of all possible point mutations for regions of genes in yeast. *Nat. Protoc.* **7**, 1382–1396 (2012). 22722372 10.1038/nprot.2012.069 PMC3509169

[48] Chimnaronk, S. et al. RNA helicase module in an acetyltransferase that modifies a specific tRNA anticodon. *EMBO J.* **28**, 1362–1373 (2009). 19322199 10.1038/emboj.2009.69 PMC2683049

[49] Ikeuchi, Y., Kitahara, K. & Suzuki, T. The RNA acetyltransferase driven by ATP hydrolysis synthesizes N4-acetylcytidine of tRNA anticodon. *EMBO J.* **27**, 2194–2203 (2008). 18668122 10.1038/emboj.2008.154 PMC2500205

[50] Lee, B. H. et al. FLT3 mutations confer enhanced proliferation and survival properties to multipotent progenitors in a murine model of chronic myelomonocytic leukemia. *Cancer Cell* **12**, 367–380 (2007). 17936561 10.1016/j.ccr.2007.08.031 PMC2104473

[51] Relier, S., Schiffers, S., Beiki, H. & Oberdoerffer, S. Enhanced ac4C detection in RNA via chemical reduction and cDNA synthesis with modified dNTPs. *RNA* **30**, 938–953 (2024). 38697668 10.1261/rna.079863.123 PMC11182010

[52] Beiki, H. et al. Detection of ac4C in human mRNA is preserved upon data reassessment. *Mol. Cell* **84**, 1611–1625.e1613 (2024). 38640896 10.1016/j.molcel.2024.03.018 PMC11353019

[53] Dorner, K., Ruggeri, C., Zemp, I. & Kutay, U. Ribosome biogenesis factors-from names to functions. *EMBO J.* **42**, e112699 (2023). 36762427 10.15252/embj.2022112699 PMC10068337

[54] Singh, S., Vanden Broeck, A., Miller, L., Chaker-Margot, M. & Klinge, S. Nucleolar maturation of the human small subunit processome. *Science* **373**, eabj5338 (2021). 34516797 10.1126/science.abj5338 PMC8744464

[55] Cheng, J., Kellner, N., Berninghausen, O., Hurt, E. & Beckmann, R. 3.2-A-resolution structure of the 90S preribosome before A1 pre-rRNA cleavage. *Nat. Struct. Mol. Biol.* **24**, 954–964 (2017). 28967883 10.1038/nsmb.3476

[56] Kornprobst, M. et al. Architecture of the 90S Pre-ribosome: A Structural View on the Birth of the Eukaryotic Ribosome. *Cell* **166**, 380–393 (2016). 27419870 10.1016/j.cell.2016.06.014

[57] Cheng, J. et al. Thermophile 90S Pre-ribosome structures reveal the reverse order of co-transcriptional 18S rRNA subdomain integration. *Mol. Cell* **75**, 1256–1269.e1257 (2019). 31378463 10.1016/j.molcel.2019.06.032

[58] Delgado-Tejedor, A. et al. Native RNA nanopore sequencing reveals antibiotic-induced loss of rRNA modifications in the A- and P-sites. *Nat. Commun.* **15**, 10054 (2024). 39613750 10.1038/s41467-024-54368-x PMC11607429

[59] Sovic, I. et al. Fast and sensitive mapping of nanopore sequencing reads with GraphMap. *Nat. Commun.* **7**, 11307 (2016). 27079541 10.1038/ncomms11307 PMC4835549

[60] Guo, Z. et al. Nanopore current events magnifier (nanoCEM): a novel tool for visualizing current events at modification sites of nanopore sequencing. *NAR Genom. Bioinform* **6**, lqae052 (2024). 38774513 10.1093/nargab/lqae052 PMC11106030

[61] Jain, M. et al. Improved data analysis for the MinION nanopore sequencer. *Nat. Methods* **12**, 351–356 (2015). 25686389 10.1038/nmeth.3290 PMC4907500

[62] Meyer, B. et al. The Bowen–Conradi syndrome protein Nep1 (Emg1) has a dual role in eukaryotic ribosome biogenesis, as an essential assembly factor and in the methylation of Psi1191 in yeast 18S rRNA. *Nucleic Acids Res*. **39**, 1526–1537 (2011). 20972225 10.1093/nar/gkq931 PMC3045603

[63] Thomas, S. R., Keller, C. A., Szyk, A., Cannon, J. R. & Laronde-Leblanc, N. A. Structural insight into the functional mechanism of Nep1/Emg1 N1-specific pseudouridine methyltransferase in ribosome biogenesis. *Nucleic Acids Res*. **39**, 2445–2457 (2011). 21087996 10.1093/nar/gkq1131 PMC3064781

[64] Wurm, J. P. et al. The ribosome assembly factor Nep1 responsible for Bowen–Conradi syndrome is a pseudouridine-N1-specific methyltransferase. *Nucleic Acids Res*. **38**, 2387–2398 (2010). 20047967 10.1093/nar/gkp1189 PMC2853112

[65] Su, K. et al. NAT10 resolves harmful nucleolar R-loops depending on its helicase domain and acetylation of DDX21. *Cell Commun. Signal* **22**, 490 (2024). 39394182 10.1186/s12964-024-01869-3 PMC11468200

[66] Hwang, S. P. & Denicourt, C. The impact of ribosome biogenesis in cancer: from proliferation to metastasis. *NAR Cancer* **6**, zcae017 (2024). 38633862 10.1093/narcan/zcae017 PMC11023387

[67] Sleiman, S. & Dragon, F. Recent advances on the structure and function of RNA acetyltransferase Kre33/NAT10. *Cells* **8**, 1035 (2019). 10.3390/cells8091035 PMC6770127 31491951

[68] Balmus, G. et al. Targeting of NAT10 enhances healthspan in a mouse model of human accelerated aging syndrome. *Nat. Commun.* **9**, 1700 (2018). 29703891 10.1038/s41467-018-03770-3 PMC5923383

[69] Selvaratnam, L., Willson, T. M. & Schapira, M. Structural chemistry of helicase inhibition. *J. Med. Chem.* **68**, 4022–4039 (2025). 39933052 10.1021/acs.jmedchem.4c01909 PMC11873931

[70] Ran, F. A. et al. Genome engineering using the CRISPR-Cas9 system. *Nat. Protoc.* **8**, 2281–2308 (2013). 24157548 10.1038/nprot.2013.143 PMC3969860

[71] Li, H. Minimap2: pairwise alignment for nucleotide sequences. *Bioinformatics* **34**, 3094–3100 (2018). 29750242 10.1093/bioinformatics/bty191 PMC6137996

[72] Li, H. et al. The sequence alignment/Map format and SAMtools. *Bioinformatics* **25**, 2078–2079 (2009). 19505943 10.1093/bioinformatics/btp352 PMC2723002

[73] Love, M. I., Huber, W. & Anders, S. Moderated estimation of fold change and dispersion for RNA-seq data with DESeq2. *Genome Biol.* **15**, 550 (2014). 25516281 10.1186/s13059-014-0550-8 PMC4302049

[74] Tysoe, O. C. et al. Isolation and propagation of primary human cholangiocyte organoids for the generation of bioengineered biliary tissue. *Nat. Protoc.* **14**, 1884–1925 (2019). 31110298 10.1038/s41596-019-0168-0

[75] Sampaziotis, F. et al. Cholangiocyte organoids can repair bile ducts after transplantation in the human liver. *Science* **371**, 839–846 (2021). 33602855 10.1126/science.aaz6964 PMC7610478

[76] Blue, S. M. et al. Transcriptome-wide identification of RNA-binding protein binding sites using seCLIP-seq. *Nat. Protoc.* **17**, 1223–1265 (2022). 35322209 10.1038/s41596-022-00680-z PMC11134598

[77] Martin, M. Cutadapt removes adapter sequences from high-throughput sequencing reads. *EMBnet. J.* **17**, 10–12 (2011).

[78] Smith, T., Heger, A. & Sudbery, I. UMI-tools: modeling sequencing errors in Unique Molecular Identifiers to improve quantification accuracy. *Genome Res.* **27**, 491–499 (2017). 28100584 10.1101/gr.209601.116 PMC5340976

[79] Dobin, A. et al. STAR: ultrafast universal RNA-seq aligner. *Bioinformatics* **29**, 15–21 (2013). 23104886 10.1093/bioinformatics/bts635 PMC3530905

[80] Langmead, B. & Salzberg, S. L. Fast gapped-read alignment with Bowtie 2. *Nat. Methods* **9**, 357–359 (2012). 22388286 10.1038/nmeth.1923 PMC3322381

[81] Ramirez, F. et al. deepTools2: a next generation web server for deep-sequencing data analysis. *Nucleic Acids Res*. **44**, W160–W165 (2016). 27079975 10.1093/nar/gkw257 PMC4987876

[82] Robinson, J. T. et al. Integrative genomics viewer. *Nat. Biotechnol.* **29**, 24–26 (2011). 21221095 10.1038/nbt.1754 PMC3346182

[83] Ameismeier, M. et al. Structural basis for the final steps of human 40S ribosome maturation. *Nature* **587**, 683–687 (2020). 33208940 10.1038/s41586-020-2929-x

[84] Orellana, E. A. et al. METTL1-mediated m(7)G modification of Arg-TCT tRNA drives oncogenic transformation. *Mol. Cell* **81**, 3323–3338.e3314 (2021). 34352207 10.1016/j.molcel.2021.06.031 PMC8380730

[85] Serio, H., Dalhat, M. H. & Arango, D. Nanopore Direct RNA sequencing. The RNA helicase domain of NAT10 promotes the biogenesis of hypomodified ribosomes to enhance cancer cell proliferation. *Zenodo*10.5281/zenodo.20799580 (2026).42702606

[86] Dalhat, M. H., Serio, H. & Arango, D. DMS-Nseq. The RNA helicase domain of NAT10 promotes the biogenesis of hypomodified ribosomes to enhance cancer cell proliferation. *Zenodo*10.5281/zenodo.20784953 (2026).42702606

[87] Serio, H., Narayan, S., Dalhat, M. H. & Arango, D. eCLIP-seq. The RNA helicase domain of NAT10 promotes the biogenesis of hypomodified ribosomes to enhance cancer cell proliferation. *Zenodo*10.5281/zenodo.20799456 (2026).42702606

[88] Dalhat, M. H. & Arango, D. MErR-seq. The RNA helicase domain of NAT10 promotes the biogenesis of hypomodified ribosomes to enhance cancer cell proliferation. *Zenodo*10.5281/zenodo.21026515 (2026).42702606

[89] Serio, H., Dalhat, M. H. & Arango, D. ac4C mapping. The RNA helicase domain of NAT10 promotes the biogenesis of hypomodified ribosomes to enhance cancer cell proliferation. *Zenodo*10.5281/zenodo.20801474 (2026).42702606

