# Supplementary material for: The RNA helicase domain of NAT10 promotes the biogenesis of hypomodified ribosomes to enhance cancer cell proliferation
Source: Nat Commun. 2026 Aug 8;17:9529. doi: 10.1038/s41467-026-76383-w (PMC13547379; doi:10.1038/s41467-026-76383-w)
Supplement: Supplementary file 1 [file 41467_2026_76383_MOESM11_ESM.pdf]

nature portfolio

Corresponding author(s): Daniel Arango and Konstantinos Tzelepis

Last updated by author(s): Jul 6, 2026

# Reporting Summary

Nature Portfolio wishes to improve the reproducibility of the work that we publish. This form provides structure for consistency and transparency in reporting. For further information on Nature Portfolio policies, see our Editorial Policies and the Editorial Policy Checklist.

## Statistics

For all statistical analyses, confirm that the following items are present in the figure legend, table legend, main text, or Methods section.

| n/a | Confirmed | |
|---|---|---|
| ☐ | ☒ | The exact sample size ($n$) for each experimental group/condition, given as a discrete number and unit of measurement |
| ☐ | ☒ | A statement on whether measurements were taken from distinct samples or whether the same sample was measured repeatedly |
| ☐ | ☒ | The statistical test(s) used AND whether they are one- or two-sided<br>*Only common tests should be described solely by name; describe more complex techniques in the Methods section.* |
| ☐ | ☒ | A description of all covariates tested |
| ☐ | ☒ | A description of any assumptions or corrections, such as tests of normality and adjustment for multiple comparisons |
| ☐ | ☒ | A full description of the statistical parameters including central tendency (e.g. means) or other basic estimates (e.g. regression coefficient) AND variation (e.g. standard deviation) or associated estimates of uncertainty (e.g. confidence intervals) |
| ☐ | ☒ | For null hypothesis testing, the test statistic (e.g. $F$, $t$, $r$) with confidence intervals, effect sizes, degrees of freedom and $P$ value noted<br>*Give P values as exact values whenever suitable.* |
| ☒ | ☐ | For Bayesian analysis, information on the choice of priors and Markov chain Monte Carlo settings |
| ☒ | ☐ | For hierarchical and complex designs, identification of the appropriate level for tests and full reporting of outcomes |
| ☒ | ☐ | Estimates of effect sizes (e.g. Cohen's $d$, Pearson's $r$), indicating how they were calculated |

*Our web collection on statistics for biologists contains articles on many of the points above.*

## Software and code

Policy information about availability of computer code

| | |
|---|---|
| Data collection | No specific software used for data collection. |
| Data analysis | Analysis scripts are available on github.com/arangolab and Zenodo under DOIs 10.5281/zenodo.20799580, 10.5281/zenodo.20784953, 10.5281/zenodo.20799456, 10.5281/zenodo.21026515, and 10.5281/zenodo.20801474. The software used in this study are FastQC v 0.11.5, Deeptools v 3.5.1, Bowtie2 v 2.5.4, Cutadapt v 4.2, Samtools v 1.6, STAR v 2.7.9a, UMI-tools v 1.1.5, Bedtools v 2.29.2, Integrative Genomics Viewer v 2.19.1, sra-toolkit v 3.0.0, JACUSA2 v 2.0.4, RStudio v 4.4.0, ImageJ v 1.54c, GraphPad Prism (v10), limma v3.64.3, PyMOL v 3.1.1, Dorado v 0.8.1, Dorado v 0.5.3, graphmap v 0.5.2, blue-crab v 0.2.0, nanoCEM v 0.0.6.1, minimap2 v 2.24. |

For manuscripts utilizing custom algorithms or software that are central to the research but not yet described in published literature, software must be made available to editors and reviewers. We strongly encourage code deposition in a community repository (e.g. GitHub). See the Nature Portfolio guidelines for submitting code & software for further information.

## Data

Policy information about availability of data

All manuscripts must include a data availability statement. This statement should provide the following information, where applicable:

- Accession codes, unique identifiers, or web links for publicly available datasets
- A description of any restrictions on data availability
- For clinical datasets or third party data, please ensure that the statement adheres to our policy

The transcriptomics and deep sequencing data generated in this study have been deposited in the Gene Expression Omnibus (GEO) under accession codes

GSE291931 (DMS-Nseq), GSE291932 (Nanopore direct RNA sequencing), GSE291928 (MErR-seq), GSE291934 (eCLIP-seq), and GSE325346 (ac4C mapping) and are publicly available. All other data supporting the findings of this study are provided in the Supplementary Figures, the Supplementary Data, and the Source Data file.

## Research involving human participants, their data, or biological material

Policy information about studies with human participants or human data. See also policy information about sex, gender (identity/presentation), and sexual orientation and race, ethnicity and racism.

| | |
|---|---|
| Reporting on sex and gender | For cord blood experiment, sex ratios were balanced with 3 cord bloods from 2 female donors and 1 from a male donor. |
| Reporting on race, ethnicity, or other socially relevant groupings | Cord bloods were sourced from the Cambridge Blood and Stem Cell Biobank following a written informed consent. This consent does not allow recording of race or ethnicity. |
| Population characteristics | All cord bloods were collected at birth from full term neonates. |
| Recruitment | All cord bloods were collected under Cambridge Blood and Stem Cell Biobank ethics (REC reference: 24/EE/0116). |
| Ethics oversight | Collection, processing and experiments with cord blood samples were all performed under Cambridge Blood and Stem Cell Biobank ethics (REC reference: 24/EE/0116). The ethical review board that approved the use of liver samples was the NHS Health Research Authority, IRAS project ID 328327. |

Note that full information on the approval of the study protocol must also be provided in the manuscript.

# Field-specific reporting

Please select the one below that is the best fit for your research. If you are not sure, read the appropriate sections before making your selection.

☒ Life sciences ☐ Behavioural & social sciences ☐ Ecological, evolutionary & environmental sciences

For a reference copy of the document with all sections, see nature.com/documents/nr-reporting-summary-flat.pdf

# Life sciences study design

All studies must disclose on these points even when the disclosure is negative.

| | |
|---|---|
| Sample size | No statistical method was used to predetermine sample size. For in vivo experiments, the sample sizes were chosen based on previous studies, and the numbers were chosen to balance statistical power with practical and ethical considerations (3R). In some cellular assays such as those presented in Figures 4 and Supplementary Figure 5, subset of clones were assayed at different times, and because data exclusion was not performed, the end point ended up having differences in sample size (between n=3 and n-9). |
| Data exclusions | For in vivo experiments, animals were excluded from the study due to unrelated conditions, e.g. kinked tail or severe fight wounds. |
| Replication | The number of replicates is indicated in each figure legend. |
| Randomization | Animals were assigned randomly into experimental groups by the investigators prior to treatment. For in vivo studies, age and sex matched littermates were used within an animal experiment. For all in vitro and cell-based experiments, cells were seeded from the same passage and stock to ensure homogeneity across groups. Experimental conditions were run in parallel to minimize inter-experimental variability. Biological replicates were performed on independent days using cells from the same passage range. Due to the random integration of lentiviral vectors, independent transductions were performed throughout the study to further ensure biological replicates. Covariates such as passage number, seeding density, transductions, antibiotic selection, and culture conditions were kept consistent across all groups. |
| Blinding | Researchers were not blinded. However, for animal experiments, the technicians who provided the majority of animal care and monitored disease progression were blinded to the group allocation. All in vitro, biochemical, and cellular analyses were performed from beginning to end by the same investigators. In this context, investigators conducting the experiments were aware of the sample identities. |

# Reporting for specific materials, systems and methods

We require information from authors about some types of materials, experimental systems and methods used in many studies. Here, indicate whether each material, system or method listed is relevant to your study. If you are not sure if a list item applies to your research, read the appropriate section before selecting a response.

## Materials & experimental systems

| n/a | Involved in the study | |
|---|---|---|
| | x | Antibodies |
| | x | Eukaryotic cell lines |
| x | | Palaeontology and archaeology |
| | x | Animals and other organisms |
| x | | Clinical data |
| x | | Dual use research of concern |
| x | | Plants |

## Methods

| n/a | Involved in the study | |
|---|---|---|
| x | | ChIP-seq |
| | x | Flow cytometry |
| x | | MRI-based neuroimaging |

# Antibodies

| | |
|---|---|
| Antibodies used | anti-NAT10 (1:2500, Cat#:13365-1-AP, Proteintech); rabbit polyclonal anti-NAT10 ( 1:2500, Cat#:PA5-31376, ThermoFisher Scientific); mouse monoclonal anti-NAT10 primary (1:400, sc-271770, Santa Cruz); rabbit polyclonal anti-TSR3 (1:500, ThermoFisher Scientific, Cat#: PA5-83514); rabbit monoclonal anti-HA-Tag (1:2000, C29F4) (Cat#:3724, Cell signaling) mouse monoclonal anti-GAPDH (1:1000, clone 6C5, Cat#:sc-32233, Santa Cruz Biotechnology); mouse anti-β-actin monoclonal antibody (1:2000, Cat#:66009-1-Ig, Proteintech); b-tubulin polyclonal antibody (1:2000, Proteintech, Cat#: 10094-1-AP), mouse monoclonal anti-ac4C (1:1000, Cat#: ab252215, Abcam); anti-HA tag goat polyclonal antibody (1:5000, Cat# A190-138A, Fortis Life Sciences); goat anti-EpCAM (1:200, Cat#: AF960, RnD); mouse anti-Cytokeratin 19 (1:500, Cat#: A86355, antibodies.com ); rabbit anti-SOX9 (1:500, Cat#: ab185230, Abcam); ; anti-human-CD11b (1:200, Cat#: 982604, BioLegend), Alexafluor Anti-Goat IgG 647 (1:1000, ThermoFisher Scientific, Cat#: A21206), Alexafluor Anti-mouse IgG 568 (1:1000, ThermoFisher Scientific, Cat#: 10037), and Alexafluor Anti-Rabbit IgG 488 (1:1000, ThermoFisher Scientific, Cat#: A21447) |
| Validation | For Western blots, anti-NAT10, anti-TSR3, anti-HA, and anti-ac4C antibodies were validated using overexpression and knock-out controls. All other antibodies were used as loading control. |

# Eukaryotic cell lines

Policy information about cell lines and Sex and Gender in Research

| | |
|---|---|
| Cell line source(s) | Hep3B (ATCC, Cat# HB-8064), K562 (ATCC, Cat#: CCL-243), HEK293T (ATCC, Cat# CRL-3216), SNU-449 (ATCC, Cat# CRL-2234). The OCI-AML3 cell line was obtained from the Sanger Institute Cancer Cell Collection |
| Authentication | We authenticated the cell lines using the CLA-01_Cell Line Authentication service from the NU sequencing core. This is a Short Tandem Repeat (STR) profiling method. |
| Mycoplasma contamination | We test all cell lines in the lab for mycoplasma contamination in a monthly basis using the ATCC Universal Mycoplasma Kit Cat # 30-1012K |
| Commonly misidentified lines (See ICLAC register) | HEK293T. It was used for virus production. |

# Animals and other research organisms

Policy information about studies involving animals; ARRIVE guidelines recommended for reporting animal research, and Sex and Gender in Research

| | |
|---|---|
| Laboratory animals | The mouse strain NOD.Cg-Prkdcscid Il2rgtm1WjI/SzJ from Charles River Laboratories was used, as described in the Methods section. |
| Wild animals | N/A |
| Reporting on sex | Female animals were used for in vivo studies. Female animals were chosen in accordance with the approved animal protocol and in line with 3Rs. Using female mice avoids the severe physical injuries and chronic stress caused by aggression and fighting among co-housed male mice. These stress factors can unpredictably alter xenograft behavior and confound data. Thus, this study focuses on a single sex to maintain a highly controlled, uniform baseline to test our hypothesis. Because sex was not an experimental variable, the result can only be interpreted in the context of general in vivo tumor growth and must not be interpreted in the context of biological sex differences. |
| Field-collected samples | N/A |
| Ethics oversight | All experiments were conducted under a UK Home Office project (under the Animals (Scientific Procedures) Act 1986, Amendment Regulations (2012)) and following ethical review by the University of Cambridge Animal Welfare and Ethical Review Body. |

Note that full information on the approval of the study protocol must also be provided in the manuscript.

## Plants

| | |
|---|---|
| Seed stocks | Not applicable |
| Novel plant genotypes | Not applicable |
| Authentication | Not applicable |

## Flow Cytometry

### Plots

Confirm that:

- [x] The axis labels state the marker and fluorochrome used (e.g. CD4-FITC).
- [x] The axis scales are clearly visible. Include numbers along axes only for bottom left plot of group (a 'group' is an analysis of identical markers).
- [ ] All plots are contour plots with outliers or pseudocolor plots.
- [x] A numerical value for number of cells or percentage (with statistics) is provided.

### Methodology

| | |
|---|---|
| Sample preparation | Cells were harvested and washed twice with 1× PBS followed by staining with Zombie Violet™ Fixable Viability Kit (BioLegend®) at a dilution of 1:1000 at room temperature in the dark for 30 min. |
| Instrument | SH800S Flow Cytometer |
| Software | FlowJo |
| Cell population abundance | > 5,000 cells |
| Gating strategy | remove debris (FSC-A/SSC-A), exclude doublets (FSC-H/FSC-A), gate live/dead cells (Brilliant Violet). Boundaries for positive and negative cells are shown in Fig. 4g and Supplementary Fig. 5e |

- [x] Tick this box to confirm that a figure exemplifying the gating strategy is provided in the Supplementary Information.
