# Supplementary material for: The RNA helicase domain of NAT10 promotes the biogenesis of hypomodified ribosomes to enhance cancer cell proliferation
Source: Nat Commun. 2026 Aug 8;17:9529. doi: 10.1038/s41467-026-76383-w (PMC13547379; doi:10.1038/s41467-026-76383-w)
Supplement: Supplementary file 2 [file 41467_2026_76383_MOESM12_ESM.pdf]

nature portfolio

Peer Review File

The RNA helicase domain of NAT10 promotes the biogenesis of hypomodified ribosomes to enhance cancer cell proliferation

Corresponding Author: Dr Konstantinos Tzelepis

This file contains all reviewer reports in order by version, followed by all author rebuttals in order by version.

Attachments originally included by the reviewers as part of their assessment can be found at the end of this file.

Version 0:

Reviewer comments:

Reviewer #1

(Remarks to the Author)
This manuscript by Dalhat et al. investigates the role of NAT10 in cancer cell proliferation, with a focus on its helicase and acetyltransferase activities. The authors propose a non-canonical function for NAT10, suggesting that its helicase activity—but not its acetyltransferase activity—is critical for cancer cell proliferation, and they link this to 40S biogenesis via regulation of A1 cleavage and m1acp3ψ modification on 18S rRNA. While the study attempts to challenge existing paradigms regarding NAT10 functions, the experimental evidence is inadequate to support its claims. The inconsistencies in data, methodological limitations, and failure to address contradictory literature render the manuscript unsuitable for publication in its current form. Significant additional experimentation and rigorous validation would be required to substantiate the conclusions.
Major Concerns:
1. Multiple studies across various cancer types (e.g., PMID: 36792757; PMID: 36792757; PMID: 36792757; PMID: 39506072) have demonstrated that both the helicase and acetyltransferase activities of NAT10 are essential for its oncogenic functions, including proliferation and metastasis. These studies have also identified ac4C-modified RNAs as key mediators of NAT10-driven oncogenesis. In addition to RNA ac4C substrates, acetylation of certain proteins have also proved to be the critical targets of NAT10 in cancer cells. In sum, these findings consistently demonstrate that the acetyltransferase of NAT10 is required for its oncogenesis function on promoting cancer cell proliferation and metastasis. However, the authors' claim that the acetyltransferase activity is dispensable in this manuscript, which is not sufficiently supported. The manuscript does not adequately address this contradiction or provide robust evidence to justify the observed discrepancies.
2. The use of MTT assays as the primary method to assess cell proliferation is insufficient, as this assay determines cell viability, which is a composite of cell proliferation, metabolism, and death. Careful evaluation of NAT10 variants' effects on proliferation, cell cycle progression, apoptosis, and metabolic activity is necessary to draw definitive conclusions.
3. The authors should paid attentions to the inconsistent results across experiments. In Figure 4e, the colony forming assays show conflicting results for GNAT domain mutants (H537A, R628A, and G641E). While quantitative data suggest minimal impact, qualitative images indicate dramatic differences (e.g., G641E enhancing clonogenicity, while H537A and R628A impair it). Furthermore, the H537A mutant exhibits in vitro growth suppression in Figure 4c and 4e but promotes in vivo tumor growth in Figure 5e. Additionally, Supplemental Figure 5 shows variable effects of GNAT mutants on cell viability, which the authors overlook in their interpretation. Overall, such discrepancies are not explained and further undermine the reliability of the conclusions.
4. The authors' conclusion regarding the function of the DUF1726 domain suffers from a fundamental logical flaw. The data demonstrate that mutations in this domain generally impair cell proliferation, mirroring the phenotypic pattern observed with helicase domain mutations (Fig2.b). This similarity should strongly suggest that DUF1726 is equally essential for cell survival. However, the authors selectively focused on two atypical non-phenotypic mutants to define the domain's functon(Fig4), an approach that constitutes severe selection bias in the argumentation.
5. The unusually high staining of G641E in Figure 4e may indicate technical issues such as cell contamination or assay artifacts, which require clarification.
6. The authors' conclusion that NAT10 specifically affects cancer cells without impacting "normal" or "non-malignant" cells is

not sufficiently supported. Primarily, the use of colony formation assays in cord blood CD34+ HSPCs and FLT3ITD/+ mouse HSPCs to assess "normal" cell growth is inappropriate, as this assay evaluates stem cell differentiation potential rather than proliferation, and the FLT3ITD/+ HSPC model itself is predisposed to malignant transformation. Furthermore, deriving a generalizable conclusion from data involving only one cell line per species (Supplementary Figure 6; lines 248-250) constitutes an overinterpretation. Substantial evidence from additional normal cells is required to validate this central claim.
7. The observed phenomenon—where both mutants reduce global ac4C levels (Supplementary Figure 5C) yet only the helicase mutant inhibits proliferation—may lie in their distinct RNA substrate specificities. The proliferation defect could be driven by the helicase mutant's impact on a specific set of proliferation-critical RNAs, while the GNAT mutant affects unrelated targets. However, since the study only measures ac4C on 18S rRNA, a comprehensive assessment using mass spectrometry and epitranscriptomic sequencing is required to definitively map the respective RNA targets of each domain and resolve this model.
8. The effects of helicase domain mutations on RNA binding and ATP-dependent helicase activity are not directly assessed, leaving a critical gap in understanding the mechanistic basis of the proposed functions.
9. The link between NAT10 helicase activity, m1acp3ψ modification, and cancer cell proliferation appears correlative rather than causal. The authors do not provide functional evidence (e.g., site-directed mutagenesis or programmable editing of m1acp3ψ sites) to establish causality. Moreover, the downstream translational targets influenced by m1acp3ψ modification and their potential relevance to cancer growth remain unexplored. Importantly, the mechanistic role of NAT10 in regulating m1acp3ψ modification is unclear. It should be clarified whether NAT10 directly catalyzes this modification on rRNA or indirectly affects the m1acp3ψ biosynthetic pathway by modulating the expression of genes such as TSR3. Additional experiments are recommended to elucidate this key mechanism.
10. The study would be strengthened by a more direct test of the necessity of the helicase domain. Can the phenotype be rescued by specifically expressing the wild-type helicase domain in NAT10-depleted cells?

Reviewer #2

(Remarks to the Author)
I co-reviewed this manuscript with one of the reviewers who provided the listed reports. This is part of the Nature Communications initiative to facilitate training in peer review and to provide appropriate recognition for Early Career Researchers who co-review manuscripts.

Reviewer #3

(Remarks to the Author)
Summary
Dalhat et al. present an impressive and technically sophisticated study dissecting the oncogenic functions of the dual-activity enzyme NAT10. Using a PROTAC system (dTAGV-1) for acute protein degradation, deep mutational scanning coupled to Nanopore sequencing (DMS-Nseq), and in vivo xenograft models, they conclude that the RNA helicase domain, rather than the acetyltransferase domain, is essential for tumor growth.
Mechanistically, the authors show that helicase activity promotes the biogenesis of ribosomes deficient in $m^1acp^3\Psi$, a hypomodification characteristic of cancer ribosomes. These hypomodified ribosomes remain translationally competent and enhance proliferation.
The work is conceptually bold and methodologically rigorous, redefining NAT10’s oncogenic mechanism. However, several mechanistic gaps and validation steps should be addressed before publication.

Major Comments
1. Direct evidence for RNA helicase activity of NAT10
The central claim is that NAT10’s helicase activity, rather than its acetyltransferase function, drives tumorigenesis. However, the manuscript lacks direct biochemical evidence showing that the tested mutants are helicase-deficient.
We therefore advise the authors to either temper the title and conclusions—referring to the RNA helicase domain as the principal driver of the observed phenotypes—or include additional biochemical data (e.g., ATPase or duplex-unwinding assays) confirming loss of enzymatic helicase activity in the mutants.
2. Link between helicase activity and $m^1acp^3\Psi$ deposition
The study demonstrates that helicase mutants alter $m^1acp^3\Psi$ levels, but the mechanistic connection remains speculative. The authors should investigate whether NAT10 physically or functionally interacts with TSR3, EMG1, or SNORA13 (e.g., co-IP or proximity-labeling assays). Such data would directly link NAT10 helicase activity to $m^1acp^3\Psi$ modulation.
3. Quantification of $m^1acp^3\Psi$
Because $m^1acp^3\Psi$ occurs primarily in 18S rRNA, its stoichiometry should be validated orthogonally by LC–MS/MS. Alternatively, Nanopore cDNA-based approaches such as Nano3P-seq (Begik et al., Nat Methods 2023; Nat Protoc 2025) or FIRST-seq (Begik et al., bioRxiv 2025) could provide site-specific quantification.
In addition, the HinFI assay assumes SuperScript II always produces a C-mismatch at $m^1acp^3\Psi$, which is not universally true; this limitation should be acknowledged or validated using an alternative reverse transcriptase.

Minor Comments
• Clarify whether all helicase mutants (K290A, E389L) abolish both ATP binding and unwinding activity, or if residual ATPase activity remains.
• Cite Milenkovic & Novoa, Trends Cell Biol 2024 earlier in the Introduction when discussing rRNA-modification heterogeneity (currently only cited later).
• The Discussion could briefly consider helicase-specific inhibitors (e.g., ATP-pocket allosteric blockers) and the feasibility

of selective targeting.
• Reference 3 (Milenkovic & Novoa, 2024) lacks pagination; update DOI or page numbers before publication.
• DUF1726 domain: While DMS-Nseq shows depletion of DUF1726 mutants, this domain is not characterized. A short discussion of predicted structure or possible function would strengthen the paper.
• Cell-type differences: Some GNAT mutants show proliferation defects only in K562 but not Hep3B. Discuss potential lineage-specific dependencies (myeloid vs hepatic) and whether distinct tRNA ac^4C patterns between these lines could explain this behavior.
• Software transparency: Specify exact versions and parameters for GraphMap and Dorado used in alignment and base-calling.

• Figure 1a – Header clarity
I suggest changing the panel header to "PROTAC system" or "NAT10 PROTAC construct/system" to clearly indicate the nature of the experimental design.
• Clarify the acronym "DMS-Nseq"
The current acronym is potentially confusing, as "DMS-seq" commonly refers to dimethyl sulfate–based RNA structure probing (see, e.g., Nat Methods 2017, Nat Methods 2023, bioRxiv 2025).
To avoid ambiguity, I recommend adopting a more explicit abbreviation such as DeepMutS-seq (Deep Mutational Scanning via Nanopore sequencing) or similar, and updating figure legends and text accordingly.
• M1 mutation interpretation (Fig. 3a–b)
The authors identify M1 as critical for proliferation, consistent with total loss of protein. However, it is unclear why M1 mutants do not show the highest DMS score in Fig. 3a–b.
Please elaborate on this apparent discrepancy — for example, whether normalization, barcode representation, or statistical filtering could understate the signal for this residue.
• Reduced protein levels in GNAT mutants
The GNAT-domain variants show consistently lower protein abundance. Similar to the reduced NAT10-PROTAC levels attributed to decreased mRNA abundance, the authors should provide experimental evidence or a plausible mechanistic hypothesis (e.g., altered folding, stability, or translation efficiency) explaining these differences.
• Supporting citations for sequence-context effects (line 287)
The statement "Because adjacent nucleotides are known to influence the Nanopore signal…" would benefit from supporting references — for example, prior benchmarking of context-dependent current or dwell-time effects in Nanopore sequencing studies.

Overall Assessment
This manuscript provides compelling genetic and mechanistic evidence that NAT10's helicase domain—rather than its acetyltransferase function—drives tumorigenesis by promoting the formation of hypomodified ribosomes. Addressing the biochemical validation of helicase activity and clarifying the $m^1acp^3\Psi$ quantification methodology would substantially strengthen the conclusions.

Reviewer #4

(Remarks to the Author)
I co-reviewed this manuscript with one of the reviewers who provided the listed reports. This is part of the Nature Communications initiative to facilitate training in peer review and to provide appropriate recognition for Early Career Researchers who co-review manuscripts.

Reviewer #5

(Remarks to the Author)
In this manuscript, Dalhat et al., combine PROTAC degradation with deep mutational scanning to access the functionality of distinct domains of NAT10, an RNA helicase. The authors use both myeloid and liver cancer models and find that NAT10's RNA helicase activity is critical for sustaining cell proliferation and tumor growth. Mechanistically, this activity promotes the generation of ribosomes with reduced $m^1acp^3\Psi$ modification, thereby enhancing proliferative capacity. These findings uncover a previously unrecognized, acetylation-independent function of NAT10 in regulating ribosome biogenesis and suggest that its RNA helicase activity represents a promising therapeutic vulnerability in cancer.

Overall, the figures are clear and well-organized, and the data generally support the authors claims. However, the current version of this manuscript lacks validation in other cell culture and in vivo models, as well as clarifying statements such that it doesn't warrant publication in Nature Communications at this time. Could the authors address the following:

1. General.
a. Could the authors provide a stronger rationale for using HCC and myeloid cell lines for this study?
b. Could the others explain in more detail why they chose to pick mutations based on phylogenetic conservation. How has what we have learned from the study relevant to other species? Include this in the discussion.
c. In the discussion, could the authors discuss helicases in general as a therapeutic targets. Any successes or failures? Have any drugs made it to the clinic?
d. Do you see these mutations in human genetic data? If so, what is the frequency of alterations?
e. Are there specific genetic backgrounds (KRAS, RTK, MYC, TP53 exc) that NAT10 is a good target for?

2. Experimental Design.
a. The authors use one HCC (HepB3) and one myeloid (K562) cell culture model. This reviewer would like to see the authors validate their findings in additional HCC and myeloid models both in vitro and in vivo.
b. Since this is a cancer vulnerability, the authors should determine whether NAT10 is also important for cellular transformation in these models. Is the helicase activity relevant in that context?
3. Figures
a. Supp Fig. 1a – Show SNORD13 from DepMap, since you draw a conclusion.
b. Fig. 1d – Label northern blot with dTAGV-1 so it's clear that NAT10FKBP12-F36V worked
i. Explain why you don't see tRNA in the HepB3 vs K562
c. Fig. 1e,f – What is relative proliferation describing?
d. Fig. 1h,I – Add the time point (d) for the rescues
e. Fig. 4c,d – One graph for all conditions
f. Fig. 4d,e – The images look stretched out
i. Show the rescue for K562 in the colony formation assay
g. Supp. Fig. 6d – What does plating mean here? Why do the graphs decrease after plating? Explain in the text.
h. Fig. 6a – Explain in greater detail the differences between the two helicase mutations, since the phenotypes are distinct in the assay (NAT10K2790A vs NAT10E389L)

Reviewer #6

(Remarks to the Author)
In the present manuscript, Dalhat MH et al. show that the RNA helicase activity of NAT10, rather than its acetyltransferase function, drives tumor growth. Using PROTACs, deep mutational scanning with Nanopore sequencing, and in vivo models, they reveal that NAT10 alters rRNA modifications, leading to reduced $m^1acp^3\Psi$ levels, a feature common in cancer. This loss promotes cancer cell proliferation by generating aberrant ribosomes.

The findings are intriguing and the paper is well organized. However, some issue this limit its publication

1) The western blot in Figure 1C is not convincing. It remains unclear whether dTAGV-1 treatment specifically degrades NAT10^FKBP12-F36V or also affects endogenous NAT10 protein levels in wild-type (non-tagged) cells. If the latter, what is the underlying mechanism? Furthermore, the gel appears curved, which distorts the migration pattern and compromises the accurate assessment of molecular weight and band intensity.
2) Throughout the sections describing the DMS-Nseq screen, the authors claim reduced variance in log-fold change (logFC) with decreased cell viability, which is conceptually unclear. Was cell proliferation directly inferred from sequencing read counts? If so, this should be explicitly stated and justified. Alternatively, if a proliferation assay was conducted in parallel, the authors should provide this information. Otherwise, the claims regarding cell viability should be revised for accuracy.
3) In the third paragraph of the Results section, the authors assert that "The ATP-dependent RNA helicase activity of NAT10 is essential for cell proliferation." However, this conclusion is not substantiated experimentally. No biochemical assays (such as ATPase, helicase activity assays or RNA binding assay w/o ATP)are provided to demonstrate that this specific enzymatic function is required for the observed phenotype. Including such experiments would significantly strengthen the claim.
4) Regarding Figure 1D, the authors conclude that "ablation of 18S rRNA and tRNA ac^4C acetylation confirmed the loss of NAT10 activity in NAT10^FKBP12-F36V cells treated with dTAGV." However, in Hep3B cells, the tRNA ac^4C signal appears absent both in the wild-type and dTAGV-treated conditions, raising questions about the sensitivity and specificity of the readout. Additionally, the status of ac^4C modification on mRNA following NAT10 degradation is not addressed. Clarifying whether mRNA ac^4C is also affected would provide a more comprehensive picture of NAT10's enzymatic activity in this context.
5) The claim that NAT10 knockdown (KD) is safe in normal cells is intriguing, but the evidence is limited to CD34+ hematopoietic stem and progenitor cells (HSPCs). Given that in vivo experiments in Figure 5 utilize Hep3B hepatocellular carcinoma cells, it is important to evaluate the effect of NAT10 KD in normal hepatocytes to assess tissue-specific toxicity and better inform therapeutic relevance.
6) The reduction of $m^1acp^3\Psi$ upon NAT10 depletion is shown only in Hep3B cells, however some points regarding the molecular mechanism remain unclear. Is the reduction due to a direct interaction between NAT10 and TSR3? Does NAT10 regulate TSR3 expression at the transcriptional, post-transcriptional, or epigenetic level? Furthermore, does a similar modulation of $m^1acp^3\Psi$ occur in other cell types such as K562? Additional mechanistic insight and broader validation would help contextualize this observation.
7) The abstract would benefit from clearer organization. The central findings are not immediately evident, and it is not clear which specific cancer types were studied. Moreover, the abstract omits mention of NAT10's previously established oncogenic role via ac^4C RNA modification, which would help position the current findings within the broader literature.
8) While the manuscript centers on the oncogenic function of NAT10 in AML and HCC, it does not acknowledge earlier reports linking NAT10 to additional malignancies such as pancreatic ductal adenocarcinoma (PDAC), breast cancer, and gastric cancer. Incorporating citations to recent studies demonstrating NAT10's involvement in these cancers would better situate the work in the existing field and underscore its translational potential.
9) Finally, the manuscript lacks a schematic or figure summarizing the proposed molecular mechanism.

Reviewer #7

(Remarks to the Author)
I co-reviewed this manuscript with one of the reviewers who provided the listed reports. This is part of the Nature

Communications initiative to facilitate training in peer review and to provide appropriate recognition for Early Career Researchers who co-review manuscripts.

Version 1:

Reviewer comments:

Reviewer #1

(Remarks to the Author)
The authors have done an good job in addressing my concerns and strengthening the logical rigor of this study.

Reviewer #3

(Remarks to the Author)
I thank the authors for taking my comments into consideration. I am happy to say that the authors addressed all my concerns fairly. I have no more comments.

Reviewer #4

(Remarks to the Author)
I co-reviewed this manuscript with one of the reviewers who provided the listed reports. This is part of the Nature Communications initiative to facilitate training in peer review and to provide appropriate recognition for Early Career Researchers who co-review manuscripts.

Reviewer #5

(Remarks to the Author)
In this revised manuscript, Dalhat et al., combine PROTAC degradation with deep mutational scanning to access the functionality of distinct domains of NAT10, an RNA helicase. The authors have addressed most of the original concerns raised by this reviewer by adding more models and clarifying text. Though, the reviewer does have a couple of minor comments to help strengthen the paper and improve clarity.

1. Label Fig. 4 with dTAGv-1. It's not clear from the figure layout that cells were treated with the dTAGv-1 and then NAT mutants were expressed in the NAT10 (-) background.
2. Label Fig. 5 with the cell line that was transduced (Hep3B). I would consider showing some of the AML data in the main figure set, since it supports the observations in the liver model.

The authors do acknowledge inconsistencies within their models, however, despite this acknowledgment, the variability across their models could remain a potential impediment to publication. For example, although several comparisons between mutations reach statistical significance, the magnitude of the effects raise concerns regarding biological significance (Fig. 4 and Supp Fig. 5). In a specific example, the authors discuss H537A by commenting "For example, the GNAT mutant H537A caused a significant reduction in metabolic activity when the MTS assay was assessed 8 days after transduction in both Hep3B and K562 cells (Supplementary Fig. 5a,b), but not at 10 days after transduction (Fig. 4c,d)." This statement doesn't support the data found in the figures. There are also some inconsistencies with their sucrose gradient data (Fig. 6 and Supp. Fig 10).

Reviewer #6

(Remarks to the Author)
The reviewer's comments have been addressed satisfactorily. In my opinion, the manuscript is now suitable for publication in its current form.

Reviewer #7

(Remarks to the Author)
I co-reviewed this manuscript with one of the reviewers who provided the listed reports. This is part of the Nature Communications initiative to facilitate training in peer review and to provide appropriate recognition for Early Career Researchers who co-review manuscripts.

Version 2:

Reviewer comments:

Reviewer #5

(Remarks to the Author)
The authors have addressed my remaining concerns.

Open Access This Peer Review File is licensed under a Creative Commons Attribution 4.0 International License, which permits use, sharing, adaptation, distribution and reproduction in any medium or format, as long as you give appropriate credit to the original author(s) and the source, provide a link to the Creative Commons license, and indicate if changes were made.
In cases where reviewers are anonymous, credit should be given to 'Anonymous Referee' and the source.
The images or other third party material in this Peer Review File are included in the article's Creative Commons license, unless indicated otherwise in a credit line to the material. If material is not included in the article's Creative Commons license and your intended use is not permitted by statutory regulation or exceeds the permitted use, you will need to obtain permission directly from the copyright holder.
To view a copy of this license, visit https://creativecommons.org/licenses/by/4.0/

Revision Plan

Reviewer #1, expertise in NAT10, RNA modifications and cancer (Remarks to the Author):

This manuscript by Dalhat et al. investigates the role of NAT10 in cancer cell proliferation, with a focus on its helicase and acetyltransferase activities. The authors propose a non-canonical function for NAT10, suggesting that its helicase activity—but not its acetyltransferase activity—is critical for cancer cell proliferation, and they link this to 40S biogenesis via regulation of A1 cleavage and m1acp3ψ modification on 18S rRNA. While the study attempts to challenge existing paradigms regarding NAT10 functions, the experimental evidence is inadequate to support its claims. The inconsistencies in data, methodological limitations, and failure to address contradictory literature render the manuscript unsuitable for publication in its current form. Significant additional experimentation and rigorous validation would be required to substantiate the conclusions.

We thank the reviewer(s) for all the constructive comments. We provide a point-by-point response below.

Major Concerns:
1. Multiple studies across various cancer types (e.g., PMID: 36792757; PMID: 36792757; PMID: 36792757; PMID: 39506072) have demonstrated that both the helicase and acetyltransferase activities of NAT10 are essential for its oncogenic functions, including proliferation and metastasis. These studies have also identified ac4C-modified RNAs as key mediators of NAT10-driven oncogenesis. In addition to RNA ac4C substrates, acetylation of certain proteins have also proved to be the critical targets of NAT10 in cancer cells. In sum, these findings consistently demonstrate that the acetyltransferase of NAT10 is required for its oncogenesis function on promoting cancer cell proliferation and metastasis. However, the authors' claim that the acetyltransferase activity is dispensable in this manuscript, which is not sufficiently supported. The manuscript does not adequately address this contradiction or provide robust evidence to justify the observed discrepancies.

We thank the reviewer(s) for their comments and understand that their skepticism is rooted in the literature. This body of literature emerged mainly after we first demonstrated that NAT10 acetylates mRNAs and that ac4C enhances mRNA stability and translation efficiency (Arango et al., 2018)[1]. Although our previous studies did not address the function of the distinct domains of NAT10 in cancer cell proliferation, the first report introducing single point mutations (the widely used G641E mutant) of human NAT10 suggested that loss of its acetyltransferase activity enhances cell proliferation and reduces cellular senescence (Larrieu et al, 2014)[2]. Three subsequent studies in yeast demonstrated that the RNA helicase domain of NAT10, but not its acetyltransferase activity, was essential for cell proliferation (Taoka et al., 2014; Sharma et al., 2015; Sharma et al., 2017)[3-5]. Many studies that followed those initial benchmarking reports used a single G641E mutation in the GNAT domain to conclude that

the acetyltransferase activity of NAT10 promotes cancer.[6-21] Notably, a few studies, particularly those using different acetyltransferase mutants, such as H537A and R628A, have indicated that the acetyltransferase activity of NAT10 may affect cellular senescence or metastasis but not cell proliferation.[2, 5, 22-25] Moreover, ablation of the known molecular adapters of NAT10 — SNORD13 (snR4 and snR45 in yeast) and THUMPD1, which are required for RNA acetylation, has no reported effect on cellular fitness (Supplementary Fig. 1a).[5, 26-29]

As this literature review highlights, there are conflicting reports and no definitive paradigm regarding the functional domain of NAT10 that is required for cell proliferation. Given these discrepancies and the inherent risk of establishing paradigms from single-mutant analyses, we undertook an unbiased functional genomics study using a deep mutational scanning assay. To our knowledge, our deep mutational scanning approach represents the first systematic effort to dissect NAT10 biology.

The parallel analysis of ~1,900 mutants indicated that critical residues in the RNA helicase domain—particularly those in the ATP-binding pocket—are essential for cell proliferation. In contrast, mutations in the acetyltransferase or C-terminal domains are generally dispensable. Nevertheless, not surprisingly, certain single mutants in the acetyltransferase domain did impact proliferation, underscoring the importance of not relying on single-mutant analyses to establish functional paradigms. While we regard the deep mutational scanning assay as an agnostic, robust, and rigorous approach, our findings were independently validated using multiple single-mutant clones and orthogonal methods. Collectively, these results provide compelling evidence that the RNA helicase domain is required for cell proliferation, while the acetyltransferase domain may not be essential for this process.

Because we did not evaluate other oncogenic processes—such as migration, metastasis, or epithelial-mesenchymal transition (EMT)—we recognize that our initial conclusion may have been overly broad in characterizing the RNA helicase domain of NAT10 as an oncogenic driver. In response, we have revised our language to state precisely that the RNA helicase domain of NAT10 is required for cell proliferation. Although the acetyltransferase activity of NAT10 is less critical in our experimental settings, and consistent with the reviewers' suggestion, we now explicitly acknowledge that the acetyltransferase activity of NAT10 can exert mutant-, cell-type-, and method-specific effects on cell proliferation, whereas the RNA helicase mutants consistently exhibit a significant and essential function on cell proliferation across all orthogonal proliferation assays. In addition, we explicitly acknowledge that the acetyltransferase activity of NAT10 may regulate other oncogenic processes not examined in this study. Notably, the deep mutational library described here will enable rigorous, agnostic testing of the different domains of NAT10 in these additional processes.

2. The use of MTT assays as the primary method to assess cell proliferation is insufficient, as this assay determines cell viability, which is a composite of cell proliferation, metabolism, and

death. Careful evaluation of NAT10 variants' effects on proliferation, cell cycle progression, apoptosis, and metabolic activity is necessary to draw definitive conclusions.

We thank the reviewer(s) for their comment but we respectfully disagree with it, as it is inaccurately suggesting that our conclusions relied primarily on an MTT assay. In fact, our findings were supported by several orthogonal approaches. First, the deep mutational scanning assay provided an unbiased and robust assessment of over 1,900 mutants in competitive proliferation assays. Second, MTS and colony-formation assays corroborated the results of the deep mutational analysis. Third, *in vivo* tumor growth studies validated our cell culture findings. These independent lines of evidence consistently demonstrate that cancer cell proliferation and tumor growth are significantly reduced in the presence of RNA helicase mutants. Nevertheless, following the reviewer(s) suggestions, we have further strengthened our conclusions by including additional cell death and competitive proliferation assays, and have added results in two additional cancer cell lines. Collectively, these data show that RNA helicase mutants significantly impair cell proliferation and viability, whereas GNAT mutants have either a less significant effect or no effect on cell proliferation and viability.

3. The authors should paid attentions to the inconsistent results across experiments. In Figure 4e, the colony forming assays show conflicting results for GNAT domain mutants (H537A, R628A, and G641E). While quantitative data suggest minimal impact, qualitative images indicate dramatic differences (e.g., G641E enhancing clonogenicity, while H537A and R628A impair it). Furthermore, the H537A mutant exhibits in vitro growth suppression in Figure 4c and 4e but promotes in vivo tumor growth in Figure 5e. Additionally, Supplemental Figure 5 shows variable effects of GNAT mutants on cell viability, which the authors overlook in their interpretation. Overall, such discrepancies are not explained and further undermine the reliability of the conclusions.

We welcome the reviewer's comments. In the revised manuscript, we have provided a more detailed discussion of the inconsistencies associated with the GNAT mutants. Importantly, we explicitly acknowledge that the acetyltransferase activity of NAT10 may have context-specific effects on cell proliferation and viability. However, rigorous conclusions must be grounded in consistent, reproducible evidence. The impact of RNA helicase mutants on cancer cell proliferation was robust and highly reproducible across all orthogonal assays, in clear contrast to the acetyltransferase mutants, which exhibited mutant-, cell type-, and assay-specific effects. Even when GNAT mutants affected proliferation, the magnitude of the effect was less significant than that observed with RNA helicase domain mutations. Thus, the reproducibility and the statistical significance of the findings with RNA helicase mutants strongly support the conclusion that the RNA helicase domain of NAT10 is essential for tumor growth. We nonetheless recognize that our data does not exclude the possibility that the acetyltransferase activity of NAT10 may influence other oncogenic processes not examined in this manuscript, such as migration, metastasis, or EMT.

4. The authors' conclusion regarding the function of the DUF1726 domain suffers from a fundamental logical flaw. The data demonstrate that mutations in this domain generally impair cell proliferation, mirroring the phenotypic pattern observed with helicase domain mutations (Fig2.b). This similarity should strongly suggest that DUF1726 is equally essential for cell survival. However, the authors selectively focused on two atypical non-phenotypic mutants to define the domain's function (Fig4), an approach that constitutes severe selection bias in the argumentation.

We thank the reviewer(s) for their comments and acknowledge that we were unclear in explaining the rationale for selecting DUF1726 mutants. To clarify, we would like to mention that the ATP-binding pocket is located at the interface between the RNA helicase domain and the DUF1726 domain (Revised Fig. 3c and Supplementary Fig. 4c), and thus, the DUF1726 domain is likely required for the RNA helicase and RNA binding functions of NAT10, as previously suggested by the Suzuki group in the bacterial ortholog of NAT10.[30, 31]

We chose L114 and G143 for the following reasons:

- In Figures 3a and 3b of the revised manuscript, which consider the cumulative effects of all mutations at each residue, G143 exhibited a significant decrease in K562 cells and the largest reduction among DUF1726 residues in Hep3B, albeit not significantly (Revised Fig. 3a,b, blue asterisk).
- Among the top DUF1726 missense variants from Figures 2b and 2c (Revised Supplementary Data 3), G143 is the top residue in Hep3B, further justifying its selection for additional validation.
- In our mutational library, the DUF1726 residues closest to the ATP binding site are L114, T117, and G143 (Revised Supplementary Fig. 4c), with L114 being closest to the crucial residues K290 and E389 (Revised Supplementary Fig. 4d).
- Individual visualization of L114 and G143 residues revealed that most variants led to decreased proliferative activity, while some showed increased representation in the deep mutational screen (Revised Supplementary Fig. 4e,f). The G143H variant showed more than a four-fold decrease (log2FC = -2) in both Hep3B and K562, while L114E resulted in an approximately two-fold decrease in K562 and Hep3B (log2FC = -1) (Revised Supplementary Fig. 4e,f).
- We also considered T117, which is located near the ATP-binding pocket (Revised Supplementary Fig. 4c) and was significant in Figure 3b. However, T117 was variable at the individual-mutant level, with T117G dropping out in one cell line and enriching in another (Supplementary Data 3). Thus, we decided not to follow up on this residue.

Based on the above observations, we decided to follow up with L114 and G143. We did not expect these sites to be atypical non-phenotypic mutants. Instead, we initially expected these residues to affect low-throughput cell proliferation assays. However, the experimental results did not confirm this expectation, although G143 did display effects in some assays (Revised

Fig. 4c). As to why there are differences in the deep mutational scanning and the single-mutant validation assays, the effects of DUF1726 variants may be more apparent in competitive proliferation assays than in isolated single-mutant analyses. For example, no direct interactions between L114 and G143 with ATP are observed in structural modeling (Supplementary Fig. 4c-d), which may reduce the significant impact of these mutants.

Although we explained above the rationale to select L114 and G143, we recognize that the reviewer's primary concerns pertain to Figure 2b. It is important to clarify that in Figures 2a and 2b, the DUF1726 box plot includes residues from the most N-terminal region of NAT10 including the canonical start codon (M1), a downstream alternative start codon (M73 isoform ENST00000531159), and another nucleolar localization signal (residues 15-88),[25, 32, 33] all of which would cumulatively contribute to the overall impact of this region on competitive proliferation assays.

To finalize the response to this comment, while characterizing more single DUF1726 mutants may be useful, a more exhaustive evaluation of multiple DUF1726 mutants is out of the scope and would not alter our central conclusion that the RNA helicase domain of NAT10 is required for cancer cell proliferation. Accordingly, we have revised the results and discussion to clarify the rationale for selecting the DUF1726 mutants and to address inconsistencies in deep mutational scanning and single-mutant validation outcomes as explained above.

5. The unusually high staining of G641E in Figure 4e may indicate technical issues such as cell contamination or assay artifacts, which require clarification.

We appreciate the reviewer's concern. To address it, we performed additional replicates in Hep3B cells, ensuring that plates were not fully confluent at the endpoint. We also added colony formation assays in SNU-449 and K562 cells. Consistently, the RNA helicase mutants impaired colony formation in all cell lines, whereas the GNAT and DUF1726 clones had no significant effects relative to $NAT10^{WT}$ in colony formation assays (Fig. 4e,f, and Supplementary Fig. 5e). Some variability across replicates is expected due to differences in transduction, as fresh transductions were required for each replicate because stable cell lines expressing the RNA helicase mutants cannot be established.

6. The authors' conclusion that NAT10 specifically affects cancer cells without impacting "normal" or "non-malignant" cells is not sufficiently supported. Primarily, the use of colony formation assays in cord blood CD34+ HSPCs and FLT3ITD/+ mouse HSPCs to assess "normal" cell growth is inappropriate, as this assay evaluates stem cell differentiation potential rather than proliferation, and the FLT3ITD/+ HSPC model itself is predisposed to malignant transformation. Furthermore, deriving a generalizable conclusion from data involving only one cell line per species (Supplementary Figure 6; lines 248-250) constitutes an

overinterpretation. Substantial evidence from additional normal cells is required to validate this central claim.

We thank the reviewer(s) for their suggestion. To address the reviewer(s) concern that colony formation assays are inappropriate to assess normal cell proliferation, we incorporated a competitive proliferation assay (Revised Supplementary Fig. 7b-d). Briefly, primary murine cells were transduced with either a gRNA-control or two different gRNA-NAT10 expressing vectors, both of which co-express Blue Fluorescent Protein (BFP). In this manner, the proportion of transduced versus untransduced cells can be examined via flow cytometry at different time points post-transduction. To compare normal-like cells with highly proliferative malignant cells, we isolated primary murine HSPCs from isogenic *Flt3*$^{ltd/+}$*/Rosa26*$^{cas9/+}$ (normal phenotype),[34] and *NPM1*$^{flox\text{-}cA/+}$*/Flt3*$^{ltd/+}$*/Rosa26*$^{cas9/+}$ (malignant) mice. The *Flt3*$^{ltd/+}$*/Rosa26*$^{cas9/+}$ cells were further transduced with MLL-AF9 to generate highly proliferative *MLL-AF9 /Flt3*$^{ltd/+}$*/Rosa26*$^{cas9/+}$ cells. Both NPM1$^{flox\text{-}cA/+}$ and MLL-AF9 act as oncogenic drivers in the *Flt3*$^{ltd/+}$ background. The experimental results were consistent: CRISPR/Cas9-mediated ablation of NAT10 specifically decreased cell proliferation and clonogenic capacity in highly proliferative malignant cells but not in normal-like cells with low proliferative capacity (Revised Supplementary Fig. 7c-f).

To include an additional normal model, we turned to Intrahepatic Cholangiocyte Organoids (ICOs) derived from surgical resections of healthy livers. Organoids were transduced with two different inducible shRNAs against NAT10 or a shRNA control. NAT10 knockdown was induced with doxycycline. Induction of NAT10 knockdown had no effect on organoid growth (Revised Supplementary Fig. 8a). Importantly, microscopy inspection of organoids revealed no cytotoxic effects of NAT10 knockdown (Revised Supplementary Fig. 8b,c). These results indicate that NAT10 knockdown is not toxic in normal liver cells.

Collectively, these findings indicate that NAT10 targeting impairs cell proliferation in malignant cells, but not in normal cells with low-proliferative capacity. However, these results do not exclude the possibility that complete inhibition of NAT10 may affect other functions in normal cells. For instance, constitutive knockout of NAT10 is embryonically lethal, and a recent study shows that bi-allelic knockout of NAT10 affects normal hematopoiesis in mice.[17] In contrast, heterozygous loss of NAT10 has minimal impact on normal hematopoiesis,[17] but rather enhances healthspan in mice[35] , suggesting a systemic tolerance to partial NAT10 suppression. Therefore, there is a potential therapeutic window in which controlled inhibition of NAT10 can be exploited to enhance anti-tumor therapies. Accordingly, we have revised the results and discussion section to avoid overinterpretations.

7. The observed phenomenon—where both mutants reduce global ac4C levels (Supplementary Figure 5C) yet only the helicase mutant inhibits proliferation—may lie in their distinct RNA substrate specificities. The proliferation defect could be driven by the helicase mutant's impact on a specific set of proliferation-critical RNAs, while the GNAT mutant affects

unrelated targets. However, since the study only measures ac4C on 18S rRNA, a comprehensive assessment using mass spectrometry and epitranscriptomic sequencing is required to definitively map the respective RNA targets of each domain and resolve this model.

We performed epitranscriptomic mapping as recommended by the reviewer(s). For this purpose, we used a combination of RetraC:T-seq and ac4C-seq.[36, 37] Briefly, total RNA was treated with $NaCNBH_3$ under acidic conditions at room temperature for 5 minutes, followed by reverse transcription in the presence of the unnatural nucleotide 2-amino-ATP. This strategy reduces ac4C to a chemical form (tetrahydro-ac4C) that preferentially pairs with 2-amino-dATP (Revised Supplementary Fig. 9a), resulting in a C>T mismatch at ac4C sites (Revised Supplementary Fig. 9b). Consequently, this method enables base-resolution mapping and quantitative assessment of ac4C sites.

To identify high-confidence ac4C sites, we applied the following criteria:

1) We first identified significantly enriched mismatches in $NaCNBH_3$-treated samples compared to the respective mock-treated controls. Sites must be independently obtained in two biological replicates for further consideration. This initial filtering revealed a prevalence of C>T mismatches specific to the $NaCNBH_3$ treatment (Revised Supplementary Fig. 9c-d).
2) Because sodium borohydride can also chemically reduce other cytosine modifications such as f^5C and m^3C, to identify bona fide ac^4C sites, we selected those that were significantly decreased in the NAT10(-) conditions compared to parental Hep3B or cells reconstituted with $NAT10^{WT}$. This set of highly confident ac^4C locations mapped to rRNA, tRNAs, and a small subset of snoRNAs and snRNAs (Revised Supplementary Data 5).
3) Importantly, the modification levels of these 162 loci were decreased in NAT10(-) cells, the cells expressing RNA helicase mutants, and the cells expressing acetyltransferase mutants (Revised Supplementary Fig. 9e).
4) We next selected sites with C>T conversion rates higher than 1% in $NAT10^{WT}$ cells (Revised Fig. 6b), which are likely to be biologically relevant sites. Notably, all of them were commonly decreased in at least one RNA helicase mutant and an acetyltransferase mutant (Revised Supplementary Fig. 9f).

In summary, epitranscriptomic mapping further demonstrates that the RNA helicase domain of NAT10 is required for ac4C deposition. Importantly, these findings reinforce our initial conclusion that ac4C deposition (the acetyltransferase activity of NAT10) cannot explain the specific effect observed with RNA helicase mutants.

Nevertheless, we reported in our initial submission that the RNA helicase domain of NAT10 — unlike its acetyltransferase activity — is essential for biogenesis of the 40S ribosome subunit (Revised Fig. 6c-f). Importantly, a widely accepted paradigm is that ribosome biogenesis is essential for cell proliferation.[38] When ribosome biogenesis is impaired, it is highly unlikely that other substrates could explain the lack of cell proliferation resulting from ribosome deficiency.

Thus, the specific substrate of the RNA helicase domain of NAT10 is the biogenesis of the 40S ribosome subunit.

As to how NAT10 regulates biogenesis of the 40S ribosome subunit, it is well established that a NAT10 dimer interacts with the 90S processome, the multimolecular complex responsible for processing of the 40S ribosome subunit, where it is considered an assembly factor.[39-42] In other words, NAT10 is essential for recruiting crucial factors involved in A1 cleavage.[39-42] We added new data showing that mutations in the RNA helicase domain of NAT10 impair 18S rRNA binding (Revised Fig. 6f and Supplementary Fig. 10e), demonstrating that this domain is required for rRNA binding, A1 cleavage, and formation of mature 40S subunits (Revised Fig. 6).

To finalize the response to this comment, we would argue that in any context in which NAT10 is implicated, it is imperative to first control for the possibility that the observed phenotype is attributable to defects in ribosome biogenesis. This crucial control has rarely been included in the literature implicating NAT10 in oncogenesis.

8. The effects of helicase domain mutations on RNA binding and ATP-dependent helicase activity are not directly assessed, leaving a critical gap in understanding the mechanistic basis of the proposed functions.

To address the effect of RNA helicase mutants on RNA binding, we performed enhanced crosslinking and RNA immunoprecipitation followed by sequencing (eCLIP-seq). Previous CLIP assays in Kre33,[4] the yeast ortholog of NAT10, identified three main interacting regions between NAT10 and 18S rRNA. Two of these regions are located near the ac4C modification sites in helices 34 and 45 of 18S rRNA, while the third is at the 5' region of 18S rRNA. Notably, NAT10 binds the 5' region of 18S rRNA during the pre-A1 cleavage step, when it is associated with the 90S processome.[39-42] Interaction of NAT10 with the 90S processome is mediated by a homodimer, where one monomer anchors to 18S rRNA, while the other remains available to engage other substrates.[39-42] Pertschy and colleagues recently suggested that extensive remodeling of the 90S processome enables NAT10 to access distant helices 34 and 45, facilitating acetylation at both ac4C sites.[29] Collectively, these studies indicate that NAT10 interacts with pre-18S rRNA at distinct stages to mediate both A1 cleavage and ac4C modification. Thus, the RNA binding activity of NAT10 is independently necessary for ribosome biogenesis and ac4C modification.

Our eCLIP-seq data from wild-type cells and cells reconstituted with $NAT10^{WT}$ recapitulated these findings (Revised Fig. 6f and Supplementary Fig. 10e). We observed two main NAT10 binding regions upstream of the ac4C sites and additional binding at the 5’ region of 18S rRNA, the contact point during the pre-A1 90S processome stage (Revised Fig. 6f and Supplementary Fig. 10e). Importantly, NAT10 binding to 18S rRNA was markedly diminished in the presence of the RNA helicase mutants (Revised Fig. 6f and Supplementary Fig. 10e), indicating that these mutations abolish the RNA binding capability of NAT10. This observation provides a mechanistic explanation for why the RNA helicase mutants of NAT10 impair both

A1 cleavage and ac4C modification: since RNA binding is required for both processes, loss of this function disrupts both ribosome biogenesis and ac4C modification. It is crucial to note, however, that ac4C modification itself is not required for 40S ribosome biogenesis (Fig. 6c).[4, 5, 26, 27]

On another note, we attempted to assess the ATPase activity of NAT10. However, these experiments required highly pure NAT10, which we were unable to obtain. Initially, we performed a pull-down of 3xFLAG-tagged NAT10 from HEK293 and Hep3B cells, as described in prior studies,[2, 43] followed by in vitro ATPase assays. Unfortunately, the assays produced high background in the control samples lacking NAT10, making the assays useless. We then unsuccessfully attempted to purify recombinant His-tagged NAT10 from several bacterial strains either as a full-length protein or as a truncated version lacking the intrinsically disordered regions (IDR). All attempts resulted in cleaved NAT10.

Given that the eCLIP-seq data provide convincing evidence that the RNA helicase mutants disrupt NAT10 binding to 18S rRNA, its main cellular substrate, we have modified the title and text to focus the manuscript on the 18S rRNA-binding activity of the RNA helicase of NAT10.

9. The link between NAT10 helicase activity, m1acp3ψ modification, and cancer cell proliferation appears correlative rather than causal. The authors do not provide functional evidence (e.g., site-directed mutagenesis or programmable editing of m1acp3ψ sites) to establish causality. Moreover, the downstream translational targets influenced by m1acp3ψ modification and their potential relevance to cancer growth remain unexplored. Importantly, the mechanistic role of NAT10 in regulating m1acp3ψ modification is unclear. It should be clarified whether NAT10 directly catalyzes this modification on rRNA or indirectly affects the m1acp3ψ biosynthetic pathway by modulating the expression of genes such as TSR3. Additional experiments are recommended to elucidate this key mechanism.

We appreciate the reviewer's thoughtful comments and acknowledge that our first submission lacked a mechanism for how NAT10 regulates m1acp3ψ deposition. We provide additional data that sheds light on such a mechanism.

The two major binding sites of NAT10 identified by eCLIP-seq wrap around both ac4C sites and the m1acp3ψ position, which is spatially located proximal to ac4C:1337 in a pre-40S cryo-EM structure (Supplementary Fig. 12a-b). This observation raised the possibility that NAT10 binding to 18S rRNA could sterically hinder TSR3 from binding 18S rRNA and therefore interfere with m1acp3ψ deposition. To test this, we performed a TSR3 RNA immunoprecipitation and 18S rRNA RT-qPCR assay (Supplementary Fig. 12c-d). Our results showed that NAT10 expression reduces TSR3 association with 18S rRNA, an effect reversed by the RNA helicase mutants (Fig. 7i).

NAT10 is predominantly localized to the nucleolus, where it promotes ribosome biogenesis. In contrast, TSR3 is predominantly localized to the cytoplasm, where it catalyzes m1acp3ψ deposition during the final step of 40S biogenesis. This raises the question of how NAT10 could spatially influence TSR3 binding to 18S rRNA. Notably, NAT10 also localizes to the cytoplasm, particularly in cancers. Our subcellular fractionation experiments confirmed that NAT10 is present in the cytoplasm of Hep3B and K562 cells (Supplementary Fig. 12e). A co-immunoprecipitation assay showed that NAT10 and TSR3 interact, further supporting the notion that both proteins are in contact in the same cellular compartment (Supplementary Fig. 12f). Together, these observations suggest that cytoplasmic NAT10 may affect TSR3 activity and m1acp3ψ deposition.

To test this possibility, we generated a NAT10 clone, K993A, that shows preferential localization to the cytoplasm by systematically mutating all lysine residues in the C-terminal nucleolar localization signal of NAT10. we identified a NAT10 mutant (Supplementary Fig. 12g-h). To directly assess the impact of cytoplasmic NAT10 on m1acp3ψ levels, we overexpressed this cytoplasmic variant, $NAT10^{K993A}$, in wild-type Hep3B and K562 cells. Our data show that expression of $NAT10^{K993A}$ increases the fraction of unmodified ribosomes, indicating that cytoplasmic NAT10 impairs m1acp3ψ deposition (Fig. 7j).

Additionally, we observed that NAT10 overexpression decreases TSR3 expression in Hep3B cells (Supplementary Fig. 11g). Reduction of TSR3 expression could also lead to lower m1acp3ψ levels in cells expressing $NAT10^{WT}$. However, the effect of NAT10 on TSR3 expression is likely indirect.

Collectively, our findings support a mechanism by which cytoplasmic localization of NAT10 decreases m1acp3ψ deposition by interfering with TSR3 binding to 18S rRNA. It is important to clarify that the effect of cytoplasmic NAT10 on m1acp3ψ deposition is independent, but complementary, of the essential function of NAT10 in the nucleolus, where it promotes ribosome biogenesis via A1 cleavage. In other words, when NAT10 or its 18S rRNA binding capacity is ablated through mutations of the RNA helicase domain, there is a significant deficiency in 40S production that leads to impaired cell proliferation and cell death. On the other hand, when NAT10 expression is increased and/or translocated to the cytoplasm, it can further promote the formation of unmodified ribosomes (lacking (m1acp3ψ) by interfering with TSR3 binding to 18S rRNA or by indirectly altering TSR3 expression. In the end, there is a NAT10-dependent increase in both mature ribosomes and unmodified ribosomes, each contributing to enhanced cell proliferation.

On another note, we respectfully disagree with the experiment proposed by the reviewer(s): "site-directed mutagenesis or programmable editing of m1acp3ψ sites." Human cells contain hundreds of rDNA loci (somewhere around 200–600 rDNA),[44] making it virtually impossible to mutate or edit all loci. Furthermore, existing RNA editing strategies are limited to A→I or C→U substitutions and cannot target the relevant U residue. Even adding an exogenous

plasmid encoding a mutated rDNA would be competed off by the hundreds of endogenous rDNA copies. Additionally, mutating rRNA bases carries unpredictable consequences compared to simply modulating the deposition of the modification. Thus, a more appropriate approach is to eliminate m1acp3ψ by knocking out TSR3, as we have done (Fig. 7e; Fig. 8c,d). Notably, TSR3 knockout, resulting in the complete loss of the acp3 moiety, enhances cell proliferation, a phenotype consistent with the reduced m1acp3ψ levels observed in NAT10^{WT}-overexpressing cells.

It is unclear what the reviewer implies by this comment: "The downstream translational targets influenced by m1acp3ψ modification and their potential relevance to cancer growth remain unexplored." While a comprehensive Ribo-seq analysis in TSR3 knockout cells is outside the scope of this manuscript, we note that others have conducted similar studies.[45] These studies indicated that loss of m1acp3ψ, resulting from TSR3 knockout, leads to increased translation of ribosomal proteins, thereby establishing a positive feedback loop that promotes ribosome biogenesis.[45]

It is also important to emphasize that NAT10 does not catalyze the m1acp3ψ modification. The biosynthetic pathway for m1acp3ψ is well established, as depicted in Figure 7c. Furthermore, our data show that NAT10 expression reduces the level of this modification, which is contrary to the function of a writer enzyme.

10. The study would be strengthened by a more direct test of the necessity of the helicase domain. Can the phenotype be rescued by specifically expressing the wild-type helicase domain in NAT10-depleted cells?

It is unclear what the reviewer(s) comment refers to. We rescued the phenotype by expressing wild-type NAT10 and acetyltransferase mutants, both of which retain the wild-type RNA helicase domain. We also showed that expressing the RNA helicase mutant in wild-type cells has dominant negative effects.

If the reviewer is suggesting that we overexpress the RNA helicase domain of NAT10 as a stand-alone protein, we argue that this approach would be unfeasible due to multiple side effects arising from protein instability or altered subcellular localization. First, our deep mutational scanning shows that truncating the protein has significant negative effects across all domains of NAT10 (Supplementary Fig. 2d,e), likely because truncated versions of NAT10 are unstable. Second, the DUF1726 domain is likely required for proper folding of the ATPase binding pocket and ATP binding, as shown for the bacterial ortholog of NAT10.[30, 31] Third, nuclear and nucleolar localization signals are present in both the N-terminal and C-terminal regions of NAT10.

Reviewer #2, ECR (Remarks to the Author):

I co-reviewed this manuscript with one of the reviewers who provided the listed reports. This is part of the Nature Communications initiative to facilitate training in peer review and to provide appropriate recognition for Early Career Researchers who co-review manuscripts.

Reviewer #3, expertise in RNA modifications and long-read sequencing (Remarks to the Author):

Summary

Dalhat et al. present an impressive and technically sophisticated study dissecting the oncogenic functions of the dual-activity enzyme NAT10. Using a PROTAC system (dTAGV-1) for acute protein degradation, deep mutational scanning coupled to Nanopore sequencing (DMS-Nseq), and in vivo xenograft models, they conclude that the RNA helicase domain, rather than the acetyltransferase domain, is essential for tumor growth. Mechanistically, the authors show that helicase activity promotes the biogenesis of ribosomes deficient in $m^1acp^3\Psi$, a hypomodification characteristic of cancer ribosomes. These hypomodified ribosomes remain translationally competent and enhance proliferation. The work is conceptually bold and methodologically rigorous, redefining NAT10's oncogenic mechanism. However, several mechanistic gaps and validation steps should be addressed before publication.

We thank the reviewer(s) for the constructive comments and for considering our manuscript conceptually bold and methodologically rigorous. We provide a point-by-point response below.

Major Comments

1. Direct evidence for RNA helicase activity of NAT10

The central claim is that NAT10's helicase activity, rather than its acetyltransferase function, drives tumorigenesis. However, the manuscript lacks direct biochemical evidence showing that the tested mutants are helicase-deficient.

We therefore advise the authors to either temper the title and conclusions—referring to the RNA helicase domain as the principal driver of the observed phenotypes—or include additional biochemical data (e.g., ATPase or duplex-unwinding assays) confirming loss of enzymatic helicase activity in the mutants.

We thank the reviewer(s) for the constructive feedback. To assess the effect of RNA helicase mutants on RNA binding, we performed an eCLIP-seq experiment. A detailed explanation is provided in the response to Reviewer 1, comment 8. In summary, we found that mutations in the RNA helicase domain of NAT10 impair 18S rRNA binding, providing a mechanistic

explanation for how NAT10 regulates 40S biogenesis (Revised Fig. 6) and $m^1acp^3\Psi$ levels (Revised Fig. 7 and Supplementary Fig. 12). We, therefore, have modified the title and the text to focus the manuscript on the RNA helicase domain and its RNA binding activity, removing reference to the RNA helicase activity.

2. Link between helicase activity and $m^1acp^3\Psi$ deposition
The study demonstrates that helicase mutants alter $m^1acp^3\Psi$ levels, but the mechanistic connection remains speculative. The authors should investigate whether NAT10 physically or functionally interacts with TSR3, EMG1, or SNORA13 (e.g., co-IP or proximity-labeling assays). Such data would directly link NAT10 helicase activity to $m^1acp^3\Psi$ modulation.

We thank the reviewer for the suggestion and acknowledge that our first submission lacked a mechanism for how NAT10 regulates m1acp3ψ deposition. We provide additional data that sheds light on such a mechanism. In summary, we found that increased expression of NAT10 in the cytoplasm interferes with TSR3 binding to 18S rRNA, resulting in reduced $m^1acp^3\Psi$ levels. Please refer to Reviewer 1, comment 9, as well as revised Fig. 7 and Supplementary Fig. 12 for more details.

These findings do not rule out the possibility that NAT10 affects the initial steps of m1acp3ψ biogenesis in the nucleolus, such as SNORA13-guided pseudouridylation or EMG1-mediated methylation. However, a recent manuscript by the Mendell group showed that pseudouridylation is not required for acp3 deposition,[46] and thus an alternative pathway resulting in acp3U or m1acp3U, rather than m1acp3ψ, exists. Thus, even if NAT10 affects SNORA13-guided pseudouridylation or EMG1-mediated methylation in the nucleolus, it could still affect acp3 transfer in the cytoplasm. In the end, NAT10 contributes to ribosome heterogeneity at this particular residue.

It is important to clarify, however, that the effect of cytoplasmic NAT10 on acp3 deposition is independent of the essential function of NAT10 in the nucleolus, where it promotes ribosome biogenesis via A1 cleavage. In other words, when NAT10 or its 18S rRNA binding capacity is ablated through mutations of the RNA helicase domain, there is a significant deficiency in 40S production that leads to impaired cell proliferation and cell death. On the other hand, when NAT10 expression is increased and/or translocated to the cytoplasm, it can further promote the formation of ribosomes lacking m1acp3ψ. In the end, there is a NAT10-dependent increase in both mature ribosomes and unmodified ribosomes, each contributing to enhanced cell proliferation.

3. Quantification of $m^1acp^3\Psi$
Because $m^1acp^3\Psi$ occurs primarily in 18S rRNA, its stoichiometry should be validated orthogonally by LC–MS/MS. Alternatively, Nanopore cDNA-based approaches such as

Nano3P-seq (Begik et al., Nat Methods 2023; Nat Protoc 2025) or FIRST-seq (Begik et al., bioRxiv 2025) could provide site-specific quantification. In addition, the HinFI assay assumes SuperScript II always produces a C-mismatch at $m^1acp^3\Psi$, which is not universally true; this limitation should be acknowledged or validated using an alternative reverse transcriptase.

We thank the reviewer for the thoughtful insights. As the reviewer has noted, the HinFI digestion method does not provide absolute quantitation of $m^1acp^3\Psi$, but rather relative changes. As mentioned above, this site supports heterogeneity, potentially having acp^3U, m^1acp^3U, or $m^1acp^3\Psi$ at this residue.[46] Thus, any thorough LC-MS/MS assay should consider all these possibilities. While we did not have access to $m^1acp^3\Psi$ standards and LC-MS/MS methods for absolute quantitation, we have provided evidence that NAT10 regulates the relative modification levels by three independent methods. These methods include Nanopore direct RNA sequencing, RT-stop assays, and the HinFI digestion assays (Fig. 7 and Supplementary Fig. 11). From these methods, we pursued the HinFI digestion in all mechanistic assays because it was the most reliable and cost-effective, showing the expected dynamic range in the TSR3 knock-out and overexpression controls (Fig. 7e and Fig. 8c). It is important to note that both the HinFI digestion and RT-stop assays only measure the presence of the acp3 moiety, as the other components of this modification, pseudouridine and the methyl- group, do not alter base pairing. Since the acp3 moiety is transferred in the cytoplasm, it is crucial to perform measurement of this modification in cytoplasmic fractions, as we have done in our experiments, to exclude 18S rRNA derived from the 90S processome. In this manner, we consider the polysome fractionation and HinFI assay the most convincing, given that this assay reliably looks into the pre-40S/40S subunits while excluding the nucleolar 90S processome.

Acknowledging that no method employed in this manuscript provides absolute quantitation of the $m^1acp^3\Psi$ or any of the other biosynthetic intermediaries, we are now referring to it throughout the manuscript as relative $m^1acp^3\Psi$ (or unmodified) fraction.

Minor Comments

• Clarify whether all helicase mutants (K290A, E389L) abolish both ATP binding and unwinding activity, or if residual ATPase activity remains. The eCLIP-seq provides evidence that mutations in the RNA helicase domain abolish 18S rRNA binding. Please refer to the response to Reviewer 1 – comment 8 for a detailed explanation.

• Cite Milenkovic & Novoa, Trends Cell Biol 2024 earlier in the Introduction when discussing rRNA-modification heterogeneity (currently only cited later). The reference is currently cited in the first paragraph in this sentence: "Although once viewed as invariant machines, recent studies have demonstrated that ribosomes exhibit heterogeneity in both protein composition and ribosomal RNA (rRNA) modifications,[2,3]"

• The Discussion could briefly consider helicase-specific inhibitors (e.g., ATP-pocket allosteric blockers) and the feasibility of selective targeting.

We thank the reviewer(s) for the suggestion and have added a paragraph discussing RNA helicase inhibitors:

"Selective inhibition of RNA helicases has been achieved, and efforts to therapeutically target them are advancing from preclinical validation to early-phase human trials. For instance, an inhibitor of DHX9 (ATX-559) was recently evaluated in a Phase 1/2 trial (NCT06625515), which was terminated due to adverse effects. In contrast, an inhibitor of eIF4A (Zotatifin/eFT226) remains in Phase 1/2 trials (NCT04092673) for solid tumors. Nonetheless, no RNA helicase inhibitor has yet received FDA approval for oncology. In the case of NAT10, the deep mutational scanning assay revealed a critical functional cluster within the ATP-binding pocket of the RNA helicase domain. This suggests that ATP-competitive inhibitors or ATP-pocket allosteric blockers could be used to target NAT10. Furthermore, because the RNA-binding activity of NAT10 is required for ribosome biogenesis and cell proliferation, inhibitors that disrupt this binding may offer an alternative therapeutic strategy."

• Reference 3 (Milenkovic & Novoa, 2024) lacks pagination; update DOI or page numbers before publication.

We apologize for the oversight. The reference has been corrected.

• DUF1726 domain: While DMS-Nseq shows depletion of DUF1726 mutants, this domain is not characterized. A short discussion of predicted structure or possible function would strengthen the paper.

To clarify, the ATP-binding pocket of NAT10 is located at the interface between the RNA helicase domain and the DUF1726 domain (Reviewer Fig. 2a and 2b), and thus, the DUF1726 domain is likely part of the RNA helicase function, as previously suggested for the bacterial ortholog of NAT10.[30, 31] We agree that further structural characterization of this domain would be interesting, especially in light of potential inhibitor development. Accordingly, we have revised the results and discussion sections to address this comment. Please refer to the response to Reviewer 1, comment 4, for more details.

• Cell-type differences: Some GNAT mutants show proliferation defects only in K562 but not Hep3B. Discuss potential lineage-specific dependencies (myeloid vs hepatic) and whether distinct tRNA ac^4C patterns between these lines could explain this behavior.

We have added an extensive discussion about cell-type differences. Regarding differences in tRNA acetylation, we noted that it is more prevalent in K562 than in Hep3B (Fig. 1d), potentially

creating a major dependency for the acetyltransferase activity of NAT10 in K562. It could also work the other way around: liver cells are more dependent on ribosome biogenesis, and therefore more sensitive to disruption of the RNA helicase domain.

• Software transparency: Specify exact versions and parameters for GraphMap and Dorado used in alignment and base-calling.

We apologize for the oversight. The software versions are included in the Methods section.

• Figure 1a – Header clarity

I suggest changing the panel header to "PROTAC system" or "NAT10 PROTAC construct/system" to clearly indicate the nature of the experimental design.

Thank you for the suggestion. We have changed the figure title to "targeted degradation of NAT10".

• Clarify the acronym "DMS-Nseq"
The current acronym is potentially confusing, as "DMS-seq" commonly refers to dimethyl sulfate–based RNA structure probing (see, e.g., Nat Methods 2017, Nat Methods 2023, bioRxiv 2025).
To avoid ambiguity, I recommend adopting a more explicit abbreviation such as DeepMutS-seq (Deep Mutational Scanning via Nanopore sequencing) or similar, and updating figure legends and text accordingly.

While we agree that DMS-seq could be confusing in the RNA biology field, this acronym has long been used in the site-saturation mutagenesis and protein characterization field. In the revised version, we have tried to remove the acronym from the text as much as possible, instead referring to it as "deep mutational scanning," to avoid any confusion.

• M1 mutation interpretation (Fig. 3a–b)
The authors identify M1 as critical for proliferation, consistent with total loss of protein. However, it is unclear why M1 mutants do not show the highest DMS score in Fig. 3a–b. Please elaborate on this apparent discrepancy — for example, whether normalization, barcode representation, or statistical filtering could understate the signal for this residue.

We believe the most likely explanation is biological. One plausible explanation is that NAT10 has two alternative initiation sites, each derived from a different transcript isoform. The longer isoform contains exons 2 and 3, with the AUG start codon located in exon 2. In contrast, the shorter isoform excludes exons 2 and 3, so its start codon (AUG) is located in exon 4. Both

isoforms have been reported as functional.[47] To test which start codon is more relevant, we intentionally included both start sites (M1 and M73 in full-length NAT10) in our mutational library. The results indicate that M1 is more biologically relevant in the context of deep mutational scanning. However, based on the general mechanisms of translation initiation, if the upstream start codon is absent, translation will begin at the next available AUG—provided it is in an optimal context. Thus, if M1 is mutated, translation may still initiate at the downstream AUG (M73), producing a shorter NAT10 isoform.

Another plausible explanation is that translation still occurs from near-cognate codons that may have been introduced by mutations at M1. This means the functional impact of M1 mutations could vary depending on the specific change. In summary, both near-cognate initiation and downstream initiation at M73 could reduce the apparent significance of the M1 site. Nevertheless, our results show that altering M1 still has significant implications for cell proliferation.

• Reduced protein levels in GNAT mutants. The GNAT-domain variants show consistently lower protein abundance. Similar to the reduced NAT10-PROTAC levels attributed to decreased mRNA abundance, the authors should provide experimental evidence or a plausible mechanistic hypothesis (e.g., altered folding, stability, or translation efficiency) explaining these differences.

We conducted a protein stability assay by treating cells with the translation inhibitor cycloheximide at different time points, followed by Western blot analysis. This experiment did not reveal any changes in protein stability across clones, and thus, we believe the data is not needed in the manuscript. We do not consider translation efficiency to be a likely explanation. In fact, any changes in translation efficiency would be expected to have a more significant impact on the RNA helicase mutants, which have major defects in ribosome biogenesis.

One possible explanation involves the autoacetylation activity of NAT10. Loss of this event could lead to reduced protein levels. However, we did not test this possibility, as it falls outside the scope of this study. Consequently, we reduced the tone to avoid linking differences in GNAT function to variability in protein expression.

• Supporting citations for sequence-context effects (line 287)
The statement "Because adjacent nucleotides are known to influence the Nanopore signal…" would benefit from supporting references — for example, prior benchmarking of context-dependent current or dwell-time effects in Nanopore sequencing studies.

The appropriate reference has been added.

Overall Assessment
This manuscript provides compelling genetic and mechanistic evidence that NAT10's helicase domain—rather than its acetyltransferase function—drives tumorigenesis by promoting the formation of hypomodified ribosomes. Addressing the biochemical validation of helicase activity and clarifying the $m^1acp^3\Psi$ quantification methodology would substantially strengthen the conclusions.

Reviewer #4, ECR (Remarks to the Author):

I co-reviewed this manuscript with one of the reviewers who provided the listed reports. This is part of the Nature Communications initiative to facilitate training in peer review and to provide appropriate recognition for Early Career Researchers who co-review manuscripts.

Reviewer #5, expertise in site-saturation mutagenesis, high throughput screens, protein structure and functon(Remarks to the Author):

In this manuscript, Dalhat et al., combine PROTAC degradation with deep mutational scanning to access the functionality of distinct domains of NAT10, an RNA helicase. The authors use both myeloid and liver cancer models and find that NAT10's RNA helicase activity is critical for sustaining cell proliferation and tumor growth. Mechanistically, this activity promotes the generation of ribosomes with reduced $m^1acp^3\Psi$ modification, thereby enhancing proliferative capacity. These findings uncover a previously unrecognized, acetylation-independent function of NAT10 in regulating ribosome biogenesis and suggest that its RNA helicase activity represents a promising therapeutic vulnerability in cancer.

Overall, the figures are clear and well-organized, and the data generally support the authors claims. However, the current version of this manuscript lacks validation in other cell culture and in vivo models, as well as clarifying statements such that it doesn't warrant publication in Nature Communications at this time. Could the authors address the following:

We thank the reviewer for the constructive comments and for considering our manuscript clear and well-organized. We provide a point-by-point response below.

1. General.
a. Could the authors provide a stronger rationale for using HCC and myeloid cell lines for this study.

Below, we explain the rationale for selecting hepatocellular carcinoma (HCC) and myeloid models.

- By analyzing databases such as UALCAN and cBioPortal, which utilize TCGA data, we found that NAT10 overexpression is strongly associated with reduced patient survival across several cancer types. Notably, the most significant effects were observed in hepatocellular carcinoma and acute myeloid leukemia. These findings were published in Dalhat et al. (2024), Oncogene.[48]
- Moreover, NAT10 was initially identified as an oncogenic driver in hepatocellular carcinoma through a Sleeping Beauty insertional mutagenesis screen.[49] In this study, NAT10 promoted cancer progression in a p53-deficient background. Importantly, several follow-up studies have confirmed the oncogenic activity of NAT10 in HCC.[11, 12, 33, 50-53] Therefore, both the literature and clinical data support our selection of HCC as a model.
- In parallel, a previous CRISPR screen conducted by Tzelepis et al.,[54] identified NAT10 as a potential therapeutic vulnerability in AML, a finding experimentally corroborated by Zhang et al. (2024).[15] Together, this experimental evidence and TCGA data provide strong support for the oncogenic role of NAT10 in AML.
- Given that HCC and AML represent the strongest candidates for solid and liquid malignancies, we deliberately moved away from the conventional single-model approach to test whether NAT10 functions are conserved across fundamentally different biological contexts. This strategy increases the impact and relevance by focusing on conserved mechanisms rather than effects that may be restricted to specific cancer lineages.

In summary, we have revised our introduction to more comprehensively explain the rationale for selecting the liver and myeloid models.

b. Could the others explain in more detail why they chose to pick mutations based on phylogenetic conservation. How has what we have learned from the study relevant to other species?

NAT10 is a highly conserved enzyme in sequence, structure, and function. We thus reasoned that focusing on conservation would yield the most relevant residues. The essential role of the RNA helicase domain of NAT10 in ribosome biogenesis and cell proliferation is conserved across all eukaryotes, particularly in highly proliferative unicellular organisms.

c. In the discussion, could the authors discuss helicases in general as a therapeutic targets. Any successes or failures? Have any drugs made it to the clinic?

We have added the following statement to the discussion:

"Selective inhibition of RNA helicases has been achieved, and efforts to therapeutically target them are advancing from preclinical validation to early-phase human trials. For instance, an inhibitor of DHX9 (ATX-559) was recently evaluated in a Phase 1/2 trial (NCT06625515), which was terminated due to adverse effects. In contrast, an inhibitor of eIF4A (Zotatifin/eFT226) remains in Phase 1/2 trials (NCT04092673) for solid tumors. Nonetheless, no RNA helicase inhibitor has yet received FDA approval for oncology. In the case of NAT10, the deep mutational scanning assay revealed a critical functional cluster within the ATP-binding pocket of the RNA helicase domain. This suggests that ATP-competitive inhibitors or ATP-pocket allosteric blockers could be used to target NAT10. Furthermore, because the RNA-binding activity of NAT10 is required for ribosome biogenesis and cell proliferation, inhibitors that disrupt this binding may offer an alternative therapeutic strategy."

d. Do you see these mutations in human genetic data? If so, what is the frequency of alterations?

NAT10 is not mutated in cancers, and no human disease has been associated with mutations in NAT10. Its carcinogenic activity has consistently been associated with overexpression or mislocalization of the protein. This information has been reviewed in our previous publication[48] and incorporated into the introduction of the revised version.

e. Are there specific genetic backgrounds (KRAS, RTK, MYC, TP53 exc) that NAT10 is a good target for?

The first evidence that NAT10 was involved in carcinogenesis emerged from a Sleeping Beauty Insertional Mutagenesis screen in hepatocellular carcinoma.[49] In this model, NAT10 was found to be essential for disease progression in vivo when co-expressed with a mutated TP53.

2. Experimental Design.

a. The authors use one HCC (HepB3) and one myeloid (K562) cell culture model. This reviewer would like to see the authors validate their findings in additional HCC and myeloid models both in vitro and in vivo.

As an additional in vivo model, we performed K562 xenograft experiments (Revised Fig. 5e). As additional cellular systems, we generated NAT10$^{FKBP-F36V}$ in another HCC cell line, SNU-449, and validated in this system that NAT10 regulates cell proliferation and ribosome biogenesis through its RNA helicase domain (Supplementary Fig. 5e and Supplementary Fig. 10a). We would like to note that we initially attempted to generate the NAT10$^{FKBP-F36V}$ system in several leukemia cell lines (MOLM13, MV-4-11, U937, and K562), succeeding only in K562. Other methods to generate stable knockout or knockdown systems in AML cell lines were unsuccessful due to system leakiness, the essentiality of NAT10 in cancer cells, and the

dominant-negative nature of the RNA helicase mutants. Thus, as an additional AML cell line, we used wild-type OCI-AML3. We transduced wild-type OCI-AML3 cells with YFP, $NAT10^{WT}$, or $NAT10^{E389L}$ expression vectors and assessed cell proliferation. Remarkably, expression of the RNA helicase mutant $NAT10^{E389L}$ significantly decreased cell proliferation in Wild-type cells (Supplementary Fig. 5g-i).

b. Since this is a cancer vulnerability, the authors should determine whether NAT10 is also important for cellular transformation in these models. Is the helicase activity relevant in that context?

NAT10 is required for cancer cell proliferation, and its overexpression can further increase cell proliferation in already transformed cells (Revised Supplementary Fig. 5f). Others have shown that NAT10 overexpression, coupled with TP53 mutation, is sufficient to induce oncogenic transformation in a model of hepatocellular carcinoma induced by fatty liver disease.[49] However, NAT10 itself is not an oncogenic driver but a co-dependency factor. The vulnerability of NAT10 stems from the fact that cancer cells are more susceptible to NAT10 targeting than non-carcinogenic cells, as we observed in isogenic models of leukemia and non-carcinogenic primary cells (Revised Supplementary Fig. b-f).

3. Figures

a. Supp Fig. 1a – Show SNORD13 from DepMap, since you draw a conclusion.

SNORD13 is a non-coding small nucleolar RNA that is not typically included in CRISPR screens, and therefore, it is not available in DepMap. The conclusion that SNORD13 ablation does not affect cell proliferation comes from published literature.[4, 27, 29] This information has been included in the figure legend of Supplementary Fig. 1a

b. Fig. 1d – Label northern blot with dTAGV-1 so it's clear that NAT10FKBP12-F36V worked
i. Explain why you don't see tRNA in the HepB3 vs K562.

The figure has been labeled to include dTAG-V1 on top of the gels. We have replaced the Hep3B image with another replicate in which tRNA acetylation is observed. The reason why Hep3B exhibits less signal in the small RNA fractions than K562 is likely because Hep3B has a lower ratio of small RNAs compared to 18S/28S rRNA. tRNA acetylation was detected in the ac4C mapping experiments (Supplementary Data 5), but at a lower stoichiometry than the 18S rRNA sites, which are known to have modification levels of ~75-100% acetylation.

c. Fig. 1e,f – What is relative proliferation describing? Relative to time 0.

The y-axis title has been changed to "Proliferation (rel. to day 0)" for clarity.

d. Fig. 1h,I – Add the time point (d) for the rescues.

The figure legend includes the time point: "Cell proliferation at day 8 post-dTAG^{V}-1 treatment in K562 and Hep3B NAT10$^{FKBP12\text{-}F36V}$ cells reconstituted with exogenous NAT10^{WT} or YFP [NAT10(–)]."

e. Fig. 4c,d – One graph for all conditions.

We have modified the graph for clarity, unifying all conditions in one plot and representing the y axis as "MTS signal (rel. to NAT10^{WT})"

f. Fig. 4d,e – The images look stretched out. We have corrected the images.

i. Show the rescue for K562 in the colony formation assay.

K562 colony formation was included in Revised Fig. 4f.

g. Supp. Fig. 6d – What does plating mean here? Why do the graphs decrease after plating? Explain in the text.

In revised Supplementary Fig. 7e-f (former Supp. Fig. 6d). Cells were collected from colonies after seven days, re-counted, and replated. The same replating strategy was repeated weekly for up to three rounds. The clonogenic capacity of non-leukemic cells decreased after each replating, resulting in a reduced number of colonies (revised Supplementary Fig. 7e). In contrast, the clonogenic capacity of malignant AML cells remained consistently high after several rounds of replating (revised Supplementary Fig. 7f, *Empty gRNA*). Importantly, CRISPR/Cas9 targeting NAT10 reduced the clonogenic capacity of malignant AML cells but not of non-leukemic cells (revised Supplementary Fig. 7e,f, *Nat10 gRNA*).

h. Fig. 6a – Explain in greater detail the differences between the two helicase mutations, since the phenotypes are distinct in the assay (NAT10K2790A vs NAT10E389L).

The K290 residue corresponds to a highly conserved lysine in the Walker A motif required for ATP binding and hydrolysis.[5, 30, 31] Structural prediction with AlphaFold3 confirmed that K290 directly contacts ATP, and substitutions such as K290V or K290A abrogate this interaction (Supplementary Fig. 3d-f). The E389 residue forms part of the unusual DEAA (Walker B) motif that couples ATP hydrolysis with RNA binding and helicase function[30] (Supplementary Fig. 3d,g). In our eCLIP-seq assay, both E389 and K290 mutants substantially reduced 18S rRNA

binding. Yet, traces of 18S rRNA binding persisted in the K290 mutants, but not in the E389 mutants, potentially explaining the more severe phenotype in E389 mutants.

Reviewer #6, expertise in RNA modifications in AML (Remarks to the Author):

In the present manuscript, Dalhat MH et al. show that the RNA helicase activity of NAT10, rather than its acetyltransferase function, drives tumor growth. Using PROTACs, deep mutational scanning with Nanopore sequencing, and in vivo models, they reveal that NAT10 alters rRNA modifications, leading to reduced $m^1acp^3\Psi$ levels, a feature common in cancer. This loss promotes cancer cell proliferation by generating aberrant ribosomes.

The findings are intriguing and the paper is well organized. However, some issue this limit its publication

We thank the reviewer for the constructive comments and for considering our manuscript well-organized. We provide a point-by-point response below:

1) The western blot in Figure 1C is not convincing. It remains unclear whether dTAGV-1 treatment specifically degrades NAT10^FKBP12-F36V or also affects endogenous NAT10 protein levels in wild-type (non-tagged) cells. If the latter, what is the underlying mechanism? Furthermore, the gel appears curved, which distorts the migration pattern and compromises the accurate assessment of molecular weight and band intensity.

We appreciate the reviewer's concern and have provided quantification from three independent replicates for both Hep3B and K562 cells in the newly revised Supplementary Fig. 1d, indicating no significant change in NAT10 expression in wild-type cells treated with dTAGV-1. This small molecule is highly selective for the mutated form of $FKBP12^{F36V}$.[55, 56] However, residual binding to endogenous $FKBP12^{WT}$ could still occur during prolonged treatment. While we did not observe a significant effect on cell proliferation in wild-type cells treated with dTAGV-1, we clarify that, in all experiments with wild-type cells, untransduced $NAT10^{FKBP12\text{-}F36V}$ cells, and $NAT10^{FKBP12\text{-}F36V}$ cells reconstituted with NAT10 clones, all cells were treated equally with dTAGV-1 to control for potential confounding effects of this small molecule.

2) Throughout the sections describing the DMS-Nseq screen, the authors claim reduced variance in log-fold change (logFC) with decreased cell viability, which is conceptually unclear. Was cell proliferation directly inferred from sequencing read counts? If so, this should be explicitly stated and justified. Alternatively, if a proliferation assay was conducted in parallel, the authors should provide this information. Otherwise, the claims regarding cell viability should be revised for accuracy.

We thank the reviewer(s) for the suggestion and have added the following paragraph for clarity:

"In deep mutational dropout assays, cell proliferation is inferred from changes in mutant frequency over time.[57] Since cells carrying deleterious variants divide more slowly or fail to survive relative to other mutants in the population, reductions in frequency (dropouts) can be interpreted as impaired proliferative capacity.[57] When grouped by NAT10 domain, we observed that representation of mutants in the RNA helicase and DUF1726 domains was significantly reduced relative to the GNAT and tRBD domains (Fig. 2b,c)."

3) In the third paragraph of the Results section, the authors assert that "The ATP-dependent RNA helicase activity of NAT10 is essential for cell proliferation." However, this conclusion is not substantiated experimentally. No biochemical assays (such as ATPase, helicase activity assays or RNA binding assay w/o ATP)are provided to demonstrate that this specific enzymatic function is required for the observed phenotype. Including such experiments would significantly strengthen the claim.

We thank the reviewer(s) for the constructive feedback. To assess the effect of RNA helicase mutants on RNA binding, we performed an eCLIP-seq experiment. A detailed explanation is provided in the response to Reviewer 1, comment 8. In summary, we found that mutations in the RNA helicase domain of NAT10 impair 18S rRNA binding, providing a mechanistic explanation for how NAT10 regulates 40S biogenesis (Revised Fig. 6) and $m^1acp^3\Psi$ levels (Revised Fig. 7 and Supplementary Fig. 12). We, therefore, have modified the title and the text to focus the manuscript on the RNA helicase domain and its RNA binding activity, removing the reference to "ATP-dependent RNA helicase activity".

4) Regarding Figure 1D, the authors conclude that "ablation of 18S rRNA and tRNA ac^4C acetylation confirmed the loss of NAT10 activity in NAT10^FKBP12-F36V cells treated with dTAGV." However, in Hep3B cells, the tRNA ac^4C signal appears absent both in the wild-type and dTAGV-treated conditions, raising questions about the sensitivity and specificity of the readout. Additionally, the status of ac^4C modification on mRNA following NAT10 degradation is not addressed. Clarifying whether mRNA ac^4C is also affected would provide a more comprehensive picture of NAT10's enzymatic activity in this context.

We have replaced the Hep3B image with another replicate in which tRNA acetylation is observed. The reason why Hep3B exhibits less signal in the small RNA fractions than K562 is likely because Hep3B has a lower ratio of small RNAs compared to 18S/28S rRNA.

We performed ac^4C mapping in Hep3B cells using a combination of RetraC:T-seq and ac4C-seq.[36, 37] Please refer to the response to Reviewer 1, comment 7, for a detailed description. In summary, ac^4C mapping further demonstrated that the RNA helicase domain of NAT10 is required for all detected ac^4C sites, including those in rRNA, tRNA, and a small subset of

snoRNAs and snRNAs. Importantly, these findings reinforce our initial conclusion that ac^4C deposition cannot account for the specific anti-proliferative effect observed with RNA helicase mutants.

Our ac^4C mapping was performed on total RNA with limited Ribodepletion to capture well-established ac^4C sites in non-coding RNAs; thus, the only "mRNA sites" we identified were in intronic regions home to snoRNAs. Nevertheless, our data indicate that the specific substrate of the RNA helicase domain of NAT10 is the biogenesis of the 40S ribosome subunit, a mechanism independent of RNA acetylation. Thus, the significance of mRNA acetylation, or rRNA and tRNA acetylation for that matter, would be better evaluated in systems in which NAT10 depletion or disruption of its RNA helicase domain does not significantly impair ribosome biogenesis, as we previously reported in isolated clones of HeLa cells.[1]

5) The claim that NAT10 knockdown (KD) is safe in normal cells is intriguing, but the evidence is limited to CD34+ hematopoietic stem and progenitor cells (HSPCs). Given that in vivo experiments in Figure 5 utilize Hep3B hepatocellular carcinoma cells, it is important to evaluate the effect of NAT10 KD in normal hepatocytes to assess tissue-specific toxicity and better inform therapeutic relevance.

To include an additional normal model, we used Intrahepatic Cholangiocyte Organoids (ICOs) derived from surgical resections of healthy livers. Induction of NAT10 knockdown in this normal system had no effect on organoid growth (Revised Supplementary Fig. 8), indicating that NAT10 knockdown is not toxic in normal liver cells.

6) The reduction of $m^1acp^3\Psi$ upon NAT10 depletion is shown only in Hep3B cells, however some points regarding the molecular mechanism remain unclear. Is the reduction due to a direct interaction between NAT10 and TSR3? Does NAT10 regulate TSR3 expression at the transcriptional, post-transcriptional, or epigenetic level? Furthermore, does a similar modulation of $m^1acp^3\Psi$ occur in other cell types such as K562? Additional mechanistic insight and broader validation would help contextualize this observation.

We thank the reviewer for the suggestion and acknowledge that our first submission lacked a mechanism for how NAT10 regulates m1acp3ψ deposition. We provide additional data that sheds light on such a mechanism. In summary, we found that increased expression of NAT10 in the cytoplasm interferes with TSR3 binding to 18S rRNA, resulting in reduced $m^1acp^3\Psi$ levels. Please refer to the response to Reviewer 1, comment 9, for more details. We also added additional data for K562 (Supplementary Fig. 7f, 8f, and Supplementary Fig. 12i).

7) The abstract would benefit from clearer organization. The central findings are not immediately evident, and it is not clear which specific cancer types were studied. Moreover, the abstract omits mention of NAT10's previously established oncogenic role via ac^4C RNA modification, which would help position the current findings within the broader literature.

We thank the reviewer(s) for the suggestion. The abstract has been redacted to include the additional findings in the revision.

8) While the manuscript centers on the oncogenic function of NAT10 in AML and HCC, it does not acknowledge earlier reports linking NAT10 to additional malignancies such as pancreatic ductal adenocarcinoma (PDAC), breast cancer, and gastric cancer. Incorporating citations to recent studies demonstrating NAT10's involvement in these cancers would better situate the work in the existing field and underscore its translational potential.

These references have now been included in the introduction.

9) Finally, the manuscript lacks a schematic or figure summarizing the proposed molecular mechanism.

A final schematic is now shown in Fig. 9.

Reviewer #7, ECR (Remarks to the Author):

I co-reviewed this manuscript with one of the reviewers who provided the listed reports. This is part of the Nature Communications initiative to facilitate training in peer review and to provide appropriate recognition for Early Career Researchers who co-review manuscripts.

Rebuttal References

1. Arango, D. *et al*. Acetylation of Cytidine in mRNA Promotes Translation Efficiency. *Cell* **175**, 1872-1886 e1824 (2018).
2. Larrieu, D., Britton, S., Demir, M., Rodriguez, R. & Jackson, S.P. Chemical inhibition of NAT10 corrects defects of laminopathic cells. *Science* **344**, 527-532 (2014).
3. Taoka, M. *et al*. RNA cytidine acetyltransferase of small-subunit ribosomal RNA: identification of acetylation sites and the responsible acetyltransferase in fission yeast, Schizosaccharomyces pombe. *PLoS One* **9**, e112156 (2014).
4. Sharma, S. *et al*. Specialized box C/D snoRNPs act as antisense guides to target RNA base acetylation. *PLoS Genet* **13**, e1006804 (2017).
5. Sharma, S. *et al*. Yeast Kre33 and human NAT10 are conserved 18S rRNA cytosine acetyltransferases that modify tRNAs assisted by the adaptor Tan1/THUMPD1. *Nucleic Acids Res* **43**, 2242-2258 (2015).
6. Deng, M. *et al*. Helicobacter pylori-induced NAT10 stabilizes MDM2 mRNA via RNA acetylation to facilitate gastric cancer progression. *J Exp Clin Cancer Res* **42**, 9 (2023).

7. Wang, G. *et al.* NAT10-mediated mRNA N4-acetylcytidine modification promotes bladder cancer progression. *Clin Transl Med* **12**, e738 (2022).
8. Liao, L. *et al.* Lysine 2-hydroxyisobutyrylation of NAT10 promotes cancer metastasis in an ac4C-dependent manner. *Cell Res* **33**, 355-371 (2023).
9. Liu, Y. *et al.* m(6)A-driven NAT10 translation facilitates fatty acid metabolic rewiring to suppress ferroptosis and promote ovarian tumorigenesis through enhancing ACOT7 mRNA acetylation. *Oncogene* **43**, 3498-3516 (2024).
10. Liu, S. *et al.* NAT10 Phase Separation Regulates YTHDF1 Splicing to Promote Gastric Cancer Progression. *Cancer Res* **84**, 3207-3222 (2024).
11. Liu, H. *et al.* Targeting N4-acetylcytidine suppresses hepatocellular carcinoma progression by repressing eEF2-mediated HMGB2 mRNA translation. *Cancer Commun (Lond)* **44**, 1018-1041 (2024).
12. Wang, Y. *et al.* Chemotherapy-induced acetylation of ACLY by NAT10 promotes its nuclear accumulation and acetyl-CoA production to drive chemoresistance in hepatocellular carcinoma. *Cell Death Dis* **15**, 545 (2024).
13. Ding, M. *et al.* N-acetyltransferase 10 facilitates tumorigenesis of diffuse large B-cell lymphoma by regulating AMPK/mTOR signalling through N4-acetylcytidine modification of SLC30A9. *Clin Transl Med* **14**, e1747 (2024).
14. Wei, W. *et al.* NAT10-mediated ac4C tRNA modification promotes EGFR mRNA translation and gefitinib resistance in cancer. *Cell Rep* **42**, 112810 (2023).
15. Zhang, S. *et al.* NAT10-mediated mRNA N(4)-acetylcytidine reprograms serine metabolism to drive leukaemogenesis and stemness in acute myeloid leukaemia. *Nat Cell Biol* **26**, 2168-2182 (2024).
16. Wang, H. *et al.* NAT10 triggers colorectal cancer progression via promoting PPAN-regulated DNA damage repair. *Oncogene* **45**, 558-576 (2026).
17. Huang, F. *et al.* Nat10-mediated ac4C epitranscriptomics orchestrates hematopoietic stem/progenitor cell fate determination via translation control. *Sci Adv* **12**, eady0301 (2026).
18. Li, H. *et al.* Mechanistic study of N-acetyltransferase 10 deficiency enhancing olaparib sensitivity in triple negative breast cancer by inhibiting RAD51 N4-acetylcytidine modification. *iScience* **28**, 112860 (2025).
19. Huang, L. *et al.* N(4)-acetylcytidine modification of ITGB5 mRNA mediated by NAT10 promotes perineural invasion in pancreatic ductal adenocarcinoma. *J Exp Clin Cancer Res* **44**, 103 (2025).
20. Wang, Q. *et al.* Glucose homeostasis controls N-acetyltransferase 10-mediated ac4C modification of HK2 to drive gastric tumorigenesis. *Theranostics* **15**, 2428-2450 (2025).
21. Yu, C. *et al.* NAT10 promotes vascular remodelling via mRNA ac4C acetylation. *Eur Heart J* **46**, 288-304 (2025).
22. Chen, J.F. *et al.* An in vivo screen identifies NAT10 as a master regulator of brain metastasis. *Sci Adv* **11**, eads6021 (2025).
23. Shi, L. *et al.* NAT10 regulates heart development and function by maintaining the expression of genes related to fatty acid beta-oxidation and heart contraction. *Cell Death Differ* **33**, 358-373 (2026).

24. Amin, R. *et al.* NAT10 promotes cancer metastasis by modulating p300/CBP activity through chromatin-associated tRNA. *Mol Cell* **85**, 4526-4544 e4518 (2025).
25. Zhou, M. *et al.* Molecular Basis for RNA Cytidine Acetylation by NAT10. *bioRxiv* (2024).
26. Bortolin-Cavaille, M.L. *et al.* Probing small ribosomal subunit RNA helix 45 acetylation across eukaryotic evolution. *Nucleic Acids Res* **50**, 6284-6299 (2022).
27. Thalalla Gamage, S. *et al.* Antisense pairing and SNORD13 structure guide RNA cytidine acetylation. *RNA* **28**, 1582-1596 (2022).
28. Thalalla Gamage, S. *et al.* Transfer RNA acetylation regulates in vivo mammalian stress signaling. *Sci Adv* **11**, eads2923 (2025).
29. Hafner, J. *et al.* Sni445 recruits box C/D snoRNPs snR4 and snR45 to guide ribosomal RNA acetylation by Kre33. *Nucleic Acids Res* **54** (2026).
30. Chimnaronk, S. *et al.* RNA helicase module in an acetyltransferase that modifies a specific tRNA anticodon. *EMBO J* **28**, 1362-1373 (2009).
31. Ikeuchi, Y., Kitahara, K. & Suzuki, T. The RNA acetyltransferase driven by ATP hydrolysis synthesizes N4-acetylcytidine of tRNA anticodon. *EMBO J* **27**, 2194-2203 (2008).
32. Sleiman, S. & Dragon, F. Recent Advances on the Structure and Function of RNA Acetyltransferase Kre33/NAT10. *Cells* **8** (2019).
33. Tan, Y. *et al.* Loss of nucleolar localization of NAT10 promotes cell migration and invasion in hepatocellular carcinoma. *Biochem Biophys Res Commun* **499**, 1032-1038 (2018).
34. Lee, B.H. *et al.* FLT3 mutations confer enhanced proliferation and survival properties to multipotent progenitors in a murine model of chronic myelomonocytic leukemia. *Cancer Cell* **12**, 367-380 (2007).
35. Balmus, G. *et al.* Targeting of NAT10 enhances healthspan in a mouse model of human accelerated aging syndrome. *Nat Commun* **9**, 1700 (2018).
36. Sas-Chen, A. *et al.* Dynamic RNA acetylation revealed by quantitative cross-evolutionary mapping. *Nature* **583**, 638-643 (2020).
37. Relier, S., Schiffers, S., Beiki, H. & Oberdoerffer, S. Enhanced ac4C detection in RNA via chemical reduction and cDNA synthesis with modified dNTPs. *RNA* **30**, 938-953 (2024).
38. Hwang, S.P. & Denicourt, C. The impact of ribosome biogenesis in cancer: from proliferation to metastasis. *NAR Cancer* **6**, zcae017 (2024).
39. Cheng, J., Kellner, N., Berninghausen, O., Hurt, E. & Beckmann, R. 3.2-A-resolution structure of the 90S preribosome before A1 pre-rRNA cleavage. *Nat Struct Mol Biol* **24**, 954-964 (2017).
40. Kornprobst, M. *et al.* Architecture of the 90S Pre-ribosome: A Structural View on the Birth of the Eukaryotic Ribosome. *Cell* **166**, 380-393 (2016).
41. Cheng, J. *et al.* Thermophile 90S Pre-ribosome Structures Reveal the Reverse Order of Co-transcriptional 18S rRNA Subdomain Integration. *Mol Cell* **75**, 1256-1269 e1257 (2019).
42. Singh, S., Vanden Broeck, A., Miller, L., Chaker-Margot, M. & Klinge, S. Nucleolar maturation of the human small subunit processome. *Science* **373**, eabj5338 (2021).

43. Shrimp, J.H. *et al.* Remodelin Is a Cryptic Assay Interference Chemotype That Does Not Inhibit NAT10-Dependent Cytidine Acetylation. *ACS Med Chem Lett* **12**, 887-892 (2021).
44. Rothschild, D. *et al.* Diversity of ribosomes at the level of rRNA variation associated with human health and disease. *Cell Genom* **4**, 100629 (2024).
45. Babaian, A. *et al.* Loss of $m^1acp^3\Psi$ Ribosomal RNA Modification Is a Major Feature of Cancer. *Cell Rep* **31**, 107611 (2020).
46. Cheng, Y. *et al.* A non-canonical role for a small nucleolar RNA in ribosome biogenesis and senescence. *Cell* **187**, 4770-4789 e4723 (2024).
47. Lv, J. *et al.* Molecular cloning of a novel human gene encoding histone acetyltransferase-like protein involved in transcriptional activation of hTERT. *Biochem Biophys Res Commun* **311**, 506-513 (2003).
48. Dalhat, M.H., Narayan, S., Serio, H. & Arango, D. Dissecting the oncogenic properties of essential RNA-modifying enzymes: a focus on NAT10. *Oncogene* **43**, 1077-1086 (2024).
49. Tschida, B.R. *et al.* Sleeping Beauty Insertional Mutagenesis in Mice Identifies Drivers of Steatosis-Associated Hepatic Tumors. *Cancer Res* **77**, 6576-6588 (2017).
50. Li, Q. *et al.* NAT10 is upregulated in hepatocellular carcinoma and enhances mutant p53 activity. *BMC Cancer* **17**, 605 (2017).
51. Zhang, X. *et al.* High expression of N-acetyltransferase 10: a novel independent prognostic marker of worse outcome in patients with hepatocellular carcinoma. *Int J Clin Exp Pathol* **8**, 14765-14771 (2015).
52. Ma, R. *et al.* Up regulation of NAT10 promotes metastasis of hepatocellular carcinoma cells through epithelial-to-mesenchymal transition. *Am J Transl Res* **8**, 4215-4223 (2016).
53. Pan, Z. *et al.* Role of NAT10-mediated ac4C-modified HSP90AA1 RNA acetylation in ER stress-mediated metastasis and lenvatinib resistance in hepatocellular carcinoma. *Cell Death Discov* **9**, 56 (2023).
54. Tzelepis, K. *et al.* A CRISPR Dropout Screen Identifies Genetic Vulnerabilities and Therapeutic Targets in Acute Myeloid Leukemia. *Cell Rep* **17**, 1193-1205 (2016).
55. Nabet, B. *et al.* The dTAG system for immediate and target-specific protein degradation. *Nat Chem Biol* **14**, 431-441 (2018).
56. Nabet, B. *et al.* Rapid and direct control of target protein levels with VHL-recruiting dTAG molecules. *Nat Commun* **11**, 4687 (2020).
57. Hietpas, R., Roscoe, B., Jiang, L. & Bolon, D.N. Fitness analyses of all possible point mutations for regions of genes in yeast. *Nat Protoc* **7**, 1382-1396 (2012).

We thank all the reviewers for their thoughtful and supportive comments. We are excited to learn that our revised version satisfactorily addressed all the initial concerns, with six out of seven reviewers having no additional comments and suggesting publication to *Nature Communications*. Below, we specifically address the minor comments from Reviewer #5:

1. Label Fig. 4 with dTAGv-1. It's not clear from the figure layout that cells were treated with the dTAGv-1 and then NAT mutants were expressed in the NAT10 (-) background.

We thank the reviewer for the suggestion. To clarify the experimental design, we added the following statement at the beginning of the Fig. 4 legend:

"The '*wild-type*' and '*untransd.*' conditions refer to parental and $NAT10^{FKBP12\text{-}F36V}$ cells, respectively, with no transduction or clone reconstitution. NAT10(–) refers to $NAT10^{FKBP12\text{-}F36V}$ cells reconstituted with the non-oncogenic protein YFP. All other lanes refer to $NAT10^{FKBP12\text{-}F36V}$ cells reconstituted with $NAT10^{WT}$, $NAT10^{L114E}$, $NAT10^{G143H}$, $NAT10^{K290A}$, $NAT10^{K290V}$, $NAT10^{E389L}$, $NAT10^{H537A}$, $NAT10^{R628A}$, or $NAT10^{G641E}$. All cells were treated with 100 nM $dTAG^{V}$-1."

2. Label Fig. 5 with the cell line that was transduced (Hep3B). I would consider showing some of the AML data in the main figure set, since it supports the observations in the liver model.

We thank the reviewer for the suggestion. We labeled Fig. 5b-d with "Hep3B" and Fig. 5e with "K562". Regarding moving the AML data from the current Fig. S7 into the main figure set, we can discuss this possibility with the editorial team, as such re-arrangement would result in 10 main figures.

The authors do acknowledge inconsistencies within their models, however, despite this acknowledgment, the variability across their models could remain a potential impediment to publication. For example, although several comparisons between mutations reach statistical significance, the magnitude of the effects raise concerns regarding biological significance (Fig. 4 and Supp Fig. 5). In a specific example, the authors discuss H537A by commenting "For example, the GNAT mutant H537A caused a significant reduction in metabolic activity when the MTS assay was assessed 8 days after transduction in both Hep3B and K562 cells (Supplementary Fig. 5a,b), but not at 10 days after transduction (Fig. 4c,d)." This statement doesn't support the data found in the figures. There are also some inconsistencies with their sucrose gradient data (Fig. 6 and Supp. Fig 10).

We agree with the reviewer that the magnitude of the effects raises concerns regarding the biological significance of the acetyltransferase mutants. However, **it is important to emphasize that the impact of RNA helicase mutants on cancer cell proliferation and tumor growth, which is the primary focus of this study, is robust and highly reproducible across all orthogonal assays. Thus, the experimental validity,**

reproducibility and the statistical significance of the findings with RNA helicase mutants strongly and unequivocally support the conclusion that the RNA helicase domain of NAT10 is essential for cancer cell proliferation and tumor growth.

Accordingly, we have replaced the following statement on Lanes 231-232:

"mutations in the GNAT and DUF1726 domains produced inconsistent and generally less significant effects than the RNA helicase mutants."

with this one:

“mutations in the GNAT and DUF1726 domains produced diverse results, which were generally less significant, and the magnitude of the effect was less biologically relevant, compared to the RNA helicase mutants."

We believe the following comment was accurate: “For example, the GNAT mutant H537A caused a significant reduction in metabolic activity when the MTS assay was assessed 8 days after transduction in both Hep3B and K562 cells (Supplementary Fig. 5a,b), but not at 10 days after transduction (Fig. 4c,d).”

The magenta arrow in the screenshot from Supplementary Fig. 5a,b, shown below, exhibits a significant decrease in H537A. However, the magnitude and significance of the decrease were much smaller than in the RNA helicase mutants.

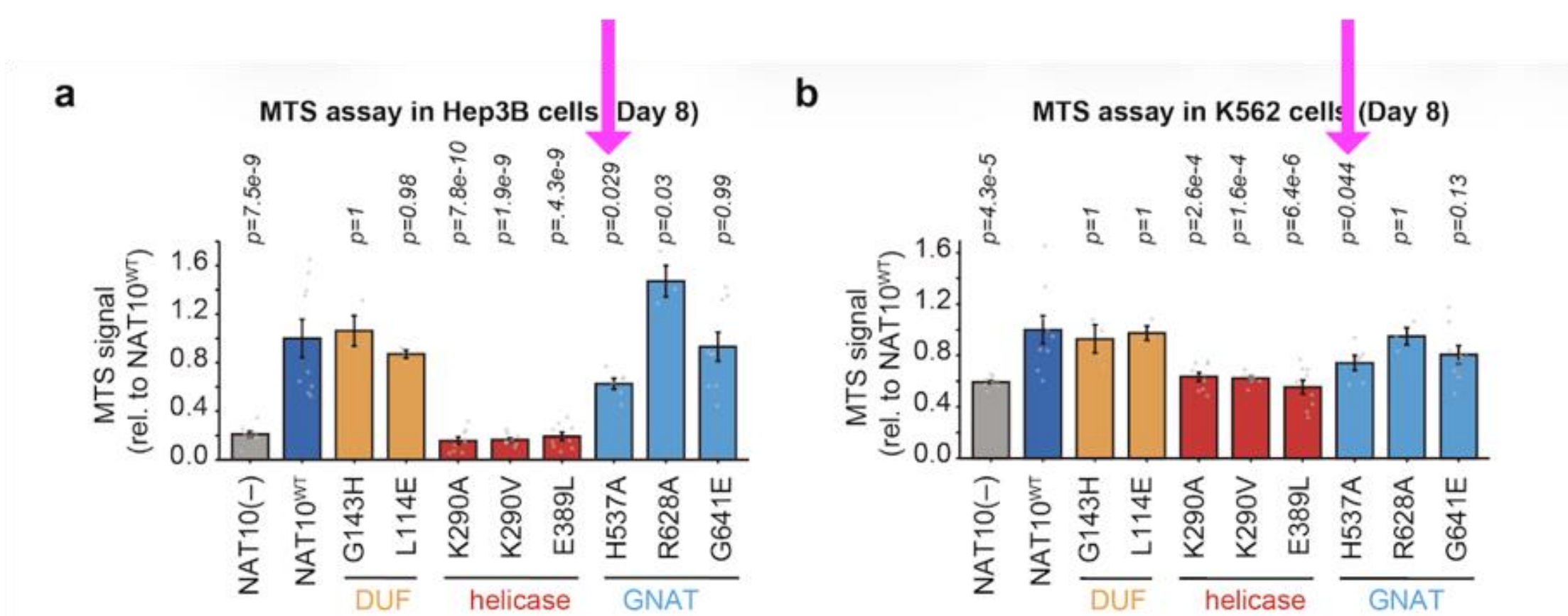

Screenshot from Supplementary Fig. 5a and b. The arrow in magenta indicates a minor significant effect for H537A at day 8 post-transduction

In contrast, the green arrow in the screenshot from Fig. 4c,d, shown below, indicates no significant effect for H537A at day 10 post-transduction.

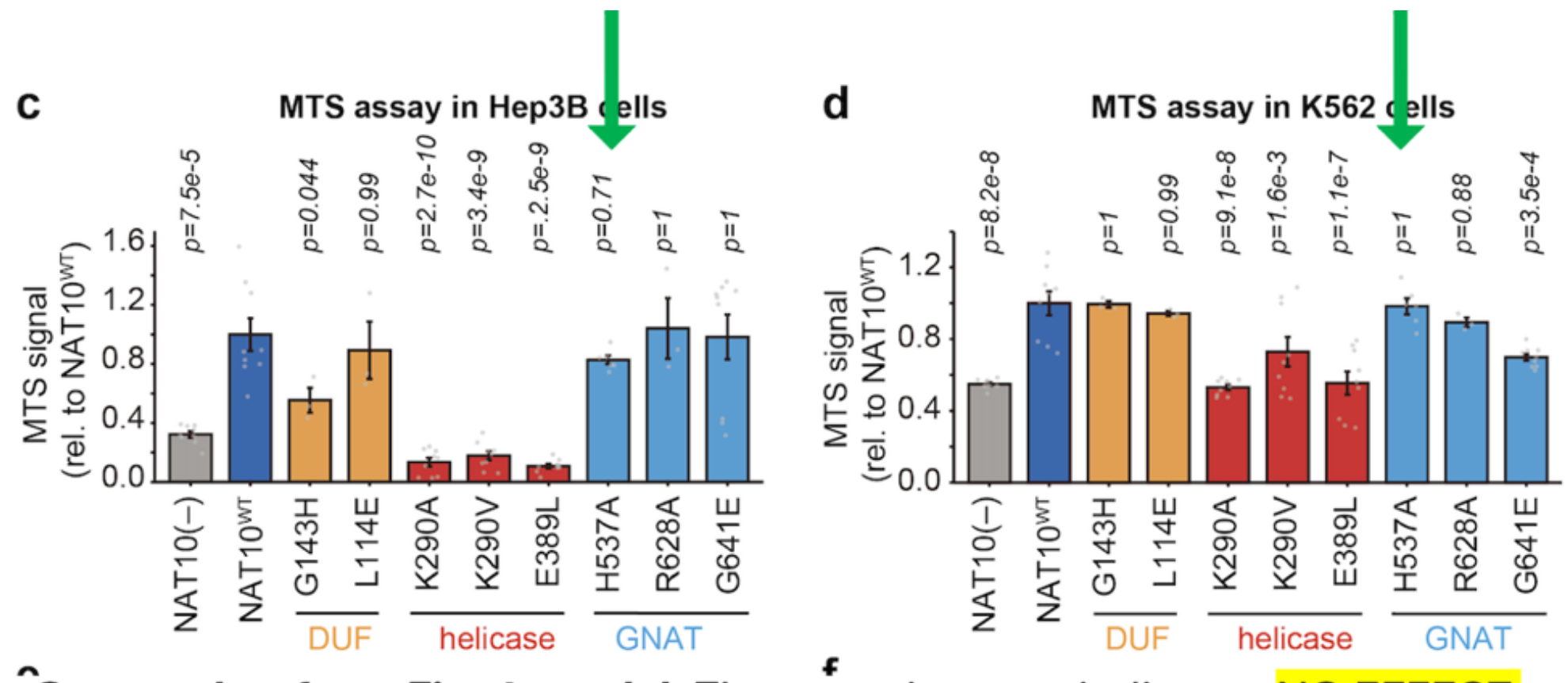

Screenshot from Fig. 4c and d. The arrow in green indicates NO EFFECT for H537A at day 10 post-transduction

Nevertheless, we have changed the statement as follows:

"For example, the GNAT mutant H537A caused a minor but significant reduction in metabolic activity when the MTS assay was assessed 8 days after transduction in both Hep3B and K562 cells (Supplementary Fig. 5a,b), but not at 10 days after transduction (Fig. 4c,d). However, the magnitude and significance of the H537A effect at day 8 were much smaller than in the RNA helicase mutants."

Regarding inconsistencies in sucrose gradient data (Fig. 6 and Supp. Fig. 10): while the reviewer does not specify which inconsistency they are referring to, it is expected that different cell lines exhibit different polysome profiles in sucrose density gradients as the profile depends on the overall translation activity of the cell type, and the availability of the ribosome subunits. For example, SNU-449 cells (Supplementary Fig. S10a) exhibit beautiful, easily detectable polysomes that were not well appreciated in K562 cells (Supplementary Fig. S10b). Similarly, some cell lines, such as Hep3B and K562, may have high-density granules or other complexes that leak into the free ribosome subunit fraction, making it a bit more challenging to separate 40S from the free RNA population at the top of the gel. Nevertheless, across all sucrose density gradients, a robust decrease in the density of 40S subunits was observed in cells lacking NAT10 or expressing RNA helicase mutants, compared with their wild-type counterparts.

In the present manuscript, Dalhat MH et al. show that the RNA helicase activity of NAT10, rather than its acetyltransferase function, drives tumor growth. Using PROTACs, deep mutational scanning with Nanopore sequencing, and in vivo models, they reveal that NAT10 alters rRNA modifications, leading to reduced $m^1acp^3\Psi$ levels, a feature common in cancer. This loss promotes cancer cell proliferation by generating aberrant ribosomes.

The findings are intriguing and the paper is well organized. However, some issue this limit its publication

Minor

 1) The western blot in Figure 1C is not convincing**.** It remains unclear whether dTAGV-1 treatment specifically degrades NAT10^FKBP12-F36V or also affects endogenous NAT10 protein levels in wild-type (non-tagged) cells. If the latter, what is the underlying mechanism? Furthermore, the gel appears curved, which distorts the migration pattern and compromises the accurate assessment of molecular weight and band intensity.

2) Throughout the sections describing the DMS-Nseq screen, the authors claim reduced variance in log-fold change (logFC) with decreased cell viability, which is conceptually unclear. Was cell proliferation directly inferred from sequencing read counts? If so, this should be explicitly stated and justified. Alternatively, if a proliferation assay was conducted in parallel, the authors should provide this information. Otherwise, the claims regarding cell viability should be revised for accuracy.

3) In the third paragraph of the Results section, the authors assert that "The ATP-dependent RNA helicase activity of NAT10 is essential for cell proliferation." However, this conclusion is not substantiated experimentally. No biochemical assays (such as ATPase, helicase activity assays or RNA binding assay w/o ATP)are provided to demonstrate that this specific enzymatic function is required for the observed phenotype. Including such experiments would significantly strengthen the claim.

4) Regarding Figure 1D, the authors conclude that "ablation of 18S rRNA and tRNA ac^4C acetylation confirmed the loss of NAT10 activity in NAT10^FKBP12-F36V cells treated with dTAGV." However, in Hep3B cells, the tRNA ac^4C signal appears absent both in the wild-type and dTAGV-treated conditions, raising questions about the sensitivity and specificity of the readout. Additionally, the status of ac^4C modification on mRNA following NAT10 degradation is not addressed. Clarifying whether mRNA ac^4C is also affected would provide a more comprehensive picture of NAT10's enzymatic activity in this context.

5) The claim that NAT10 knockdown (KD) is safe in normal cells is intriguing, but the evidence is limited to CD34+ hematopoietic stem and progenitor cells (HSPCs). Given that in vivo experiments in Figure 5 utilize Hep3B hepatocellular carcinoma cells, it is important to evaluate the effect of NAT10 KD in normal hepatocytes to assess tissue-specific toxicity and better inform therapeutic relevance.

6) The reduction of $m^1acp^3\Psi$ upon NAT10 depletion is shown only in Hep3B cells, however some points regarding the molecular mechanism remain unclear. Is the reduction due to a direct interaction between NAT10 and TSR3? Does NAT10 regulate TSR3 expression at the transcriptional, post-transcriptional, or epigenetic level? Furthermore, does a similar modulation of $m^1acp^3\Psi$ occur in other cell types such as K562? Additional mechanistic insight and broader validation would help contextualize this observation.

7) The abstract would benefit from clearer organization. The central findings are not immediately evident, and it is not clear which specific cancer types were studied. Moreover, the abstract omits mention of NAT10's previously established oncogenic role via ac^4C RNA modification, which would help position the current findings within the broader literature.

8) While the manuscript centers on the oncogenic function of NAT10 in AML and HCC, it does not acknowledge earlier reports linking NAT10 to additional malignancies such as pancreatic ductal adenocarcinoma (PDAC), breast cancer, and gastric cancer. Incorporating citations to recent studies demonstrating NAT10's involvement in these cancers would better situate the work in the existing field and underscore its translational potential.

9) Finally, the manuscript lacks a schematic or figure summarizing the proposed molecular mechanism.
