# Supplementary material for: The RNA helicase domain of NAT10 promotes the biogenesis of hypomodified ribosomes to enhance cancer cell proliferation
Source: Nat Commun. 2026 Aug 8;17:9529. doi: 10.1038/s41467-026-76383-w (PMC13547379; doi:10.1038/s41467-026-76383-w)

Supplementary Information

The RNA helicase domain of NAT10 promotes the biogenesis of hypomodified ribosomes to enhance cancer cell proliferation

Mahmood H. Dalhat[1,2†], Sharath Narayan[1,2,3†], Maria Eleftheriou[4,5,6], James Russell[4,5,6], James Heslop[4], Hannah Serio[1,2], Khulood A. Alzahrani[1,2], Adam Suh[1,2], Ajay Edakkara[4,5,6], Sweta Raikundalia[1,2], Lea Landmann[1,2], Ryan Asby[4,5], Evangelia Stamou[4,5,6], Judith López[4,5,6], Yogeshkumar Malam[4,5], Demetrios Aspris[7], Emmely A. Patrasso[1,2], Stephanie Mou[1,2,3], George Giotopoulos[4,5], Walid T. Khaled[4,8], Brian J. P. Huntly[4,5], Fotios Sampaziotis[4], Konstantinos Tzelepis[4,5,6*], Daniel Arango[1,2*]

[1]Department of Pharmacology, Feinberg School of Medicine, Northwestern University, Chicago, IL, USA

[2]Robert H. Lurie Comprehensive Cancer Center, Feinberg School of Medicine, Northwestern University, Chicago, IL, USA

[3]Driskill Graduate Program in Life Sciences, Feinberg School of Medicine, Northwestern University, Chicago, IL, USA

[4]Cambridge Stem Cell Institute, University of Cambridge, Cambridge, UK

[5]Department of Haematology, University of Cambridge, Cambridge, UK

[6]Milner Therapeutics Institute, University of Cambridge, Puddicombe Way, Cambridge, CB20AW, UK

[7]MRC Toxicology Unit, University of Cambridge, Cambridge, CB2 1QR, UK

[8]Department of Pharmacology, University of Cambridge, Cambridge, UK

[†]These authors contributed equally

*Corresponding Authors:

Daniel Arango (email: daniel.arangotamayo@northwestern.edu)

Konstantinos Tzelepis (email: kt404@cam.ac.uk)

This PDF file includes Supplementary Figures 1-12 and uncropped blots and gels related to the supplementary information

Supplementary Fig. 1: Generation of a NAT10-inducible degradation system.

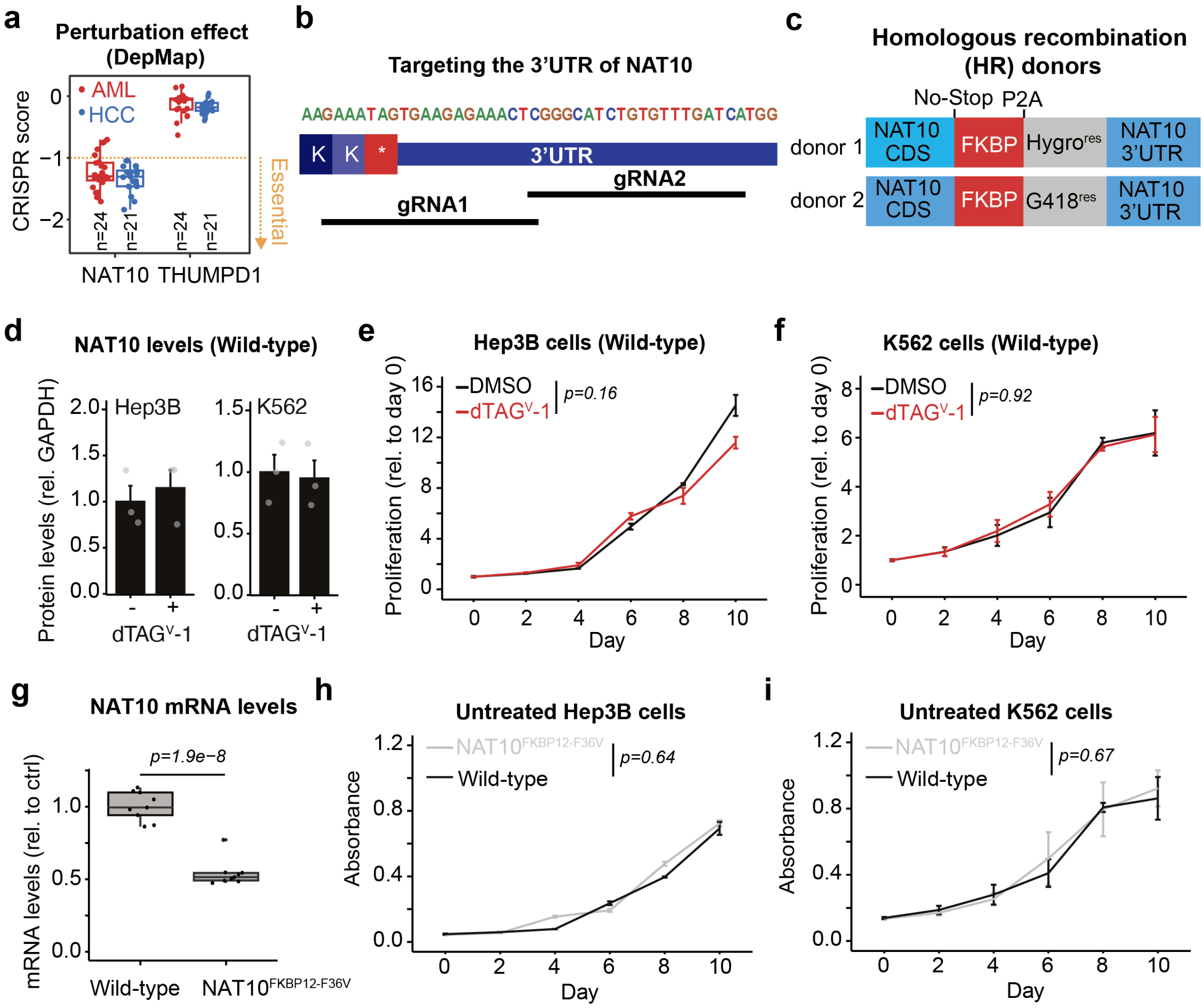

a CRISPR-based gene dependency scores for NAT10 and THUMPD1 were retrieved from DepMap and filtered to include hepatocellular carcinoma (HCC) and acute myeloid leukemia (AML) cell lines. SNORD13 is a non-coding small nucleolar RNA that is not typically included in CRISPR screens and therefore is not available in DepMap. Data represents the interquartile range (the box), the median (the line), and whiskers extending to 1.5 times the interquartile range. **b** Schematic of gRNAs targeting the C-terminal region of NAT10. **c** Schematic of homologous recombination donor plasmids. Cells were co-transfected with two donor plasmids, each encoding a different antibiotic resistance gene. Successful tagging of both alleles with FKBP12^{F36V} was selected by resistance to hygromycin and G418. **d** Quantitation of Western blots in wild-type cells treated with 100 nM dTAG^{V}-1 for 24 h. Data represent mean ± SEM from three independent biological replicates. **e,f** Cell proliferation over time in wild-type cells treated with 100 nM dTAG^{V}-1. Data represent mean ± SEM from three independent biological replicates. p-values were calculated by two-way ANOVA. dTAG^{V}-1 treatment did not significantly affect cell proliferation in wild-type cells. **g** NAT10 mRNA levels assessed by RT-qPCR in untreated wild-type and NAT10$^{FKBP12-F36V}$ Hep3B cells. mRNA levels were normalized to GAPDH and expressed relative to wild-type cells. Box plots show the median, the interquartile range (the box), and whiskers extending to 1.5 times the interquartile range. n = 8 independent biological replicates. p-value calculated by a two-sided Wilcoxon test. **h,i** Cell proliferation over time in untreated NAT10$^{FKBP-F36V}$ or wild-type cells. Data represent mean ± SEM from three independent biological replicates.

p-values were calculated by two-way ANOVA. Tagging endogenous NAT10 with $FKBP^{F36V}$ did not significantly affect cell proliferation in untreated cells. For (a) and (e-i), source data are provided as a Source Data File.

Supplementary Fig. 2: Generation and analysis of the site saturation mutational library.

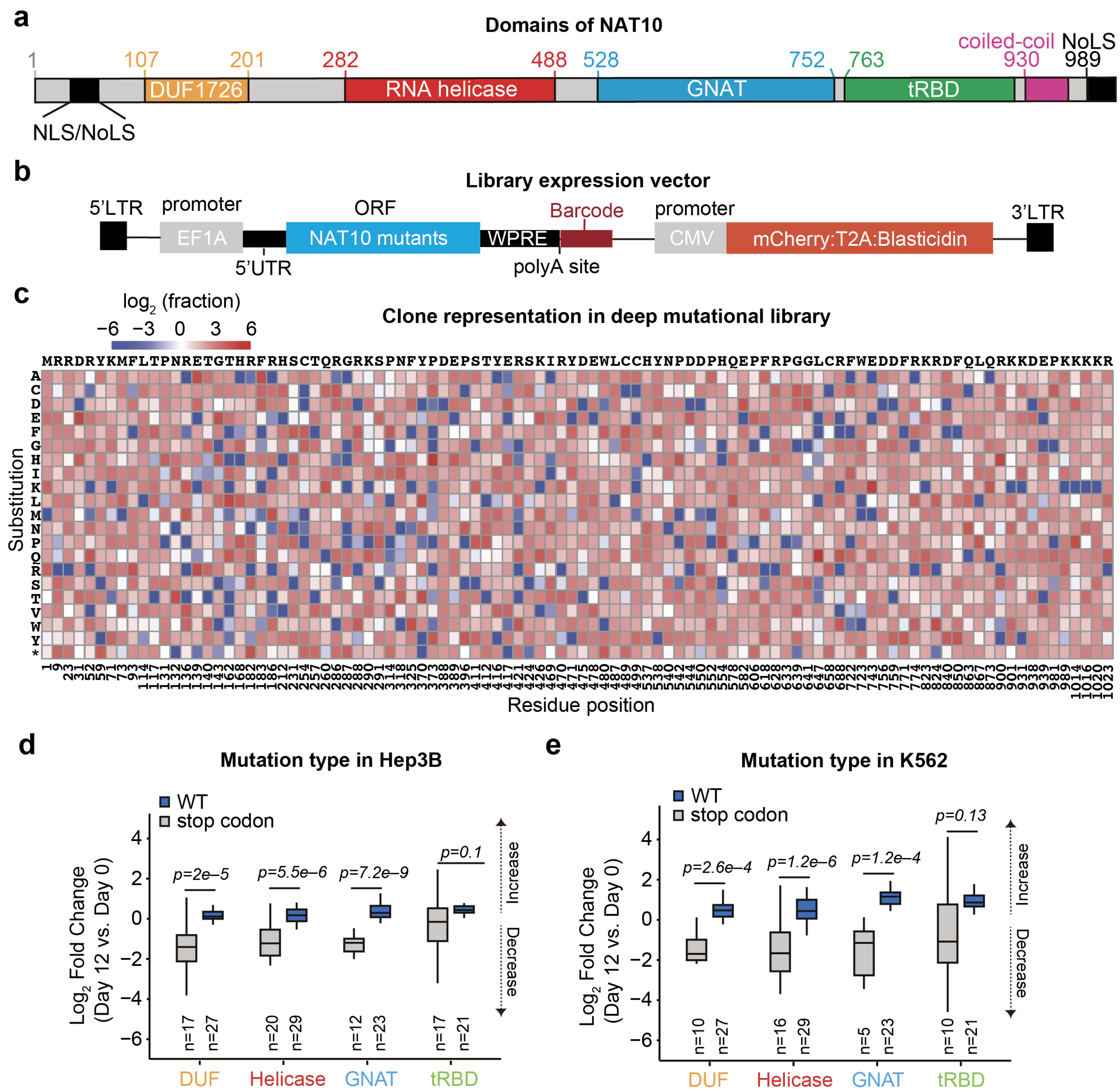

a Schematic of NAT10 protein domains, highlighting the conserved N-acetyltransferase (GNAT) domain, the RNA helicase domain, the predicted tRNA-binding domain (tRBD), the domain of unknown function (DUF1726), a coiled-coil (CC) motif, and the nuclear/nucleolar localization signals (NLS/NoLS). **b** Schematic of the lentiviral expression vector. NAT10 mutants were expressed under the control of an EF1A promoter; a unique 13-nucleotide barcode was incorporated downstream of the polyadenylation signal. Mutations were introduced at 100 residues across the NAT10 coding sequence. **c** Heatmap showing the relative representation ($\log_{10}$ fraction) of each amino acid substitution per residue in the original mutational library as reported by Twist Bioscience. The x-axis indicates residue positions; the y-axis shows possible substitutions. Red indicates highly represented clones; blue indicates low representation. **d,e** Box plots showing the $\log_2$(fold change) for nonsense mutations (premature stop codons) and wild-type (WT) clones within each NAT10 domain in Hep3B (d) and K562 (e) cells. Box plots show the median, the interquartile range (the box), and whiskers extending to 1.5 times the interquartile

range. p-values were calculated by two-sided Wilcoxon rank-sum tests (WT vs. nonsense). *n* corresponds to the number of independent clones per condition. The data is derived from three biological replicates. For (c-e), source data are provided as a Source Data File.

Supplementary Fig. 3: Analysis of single RNA helicase variants.

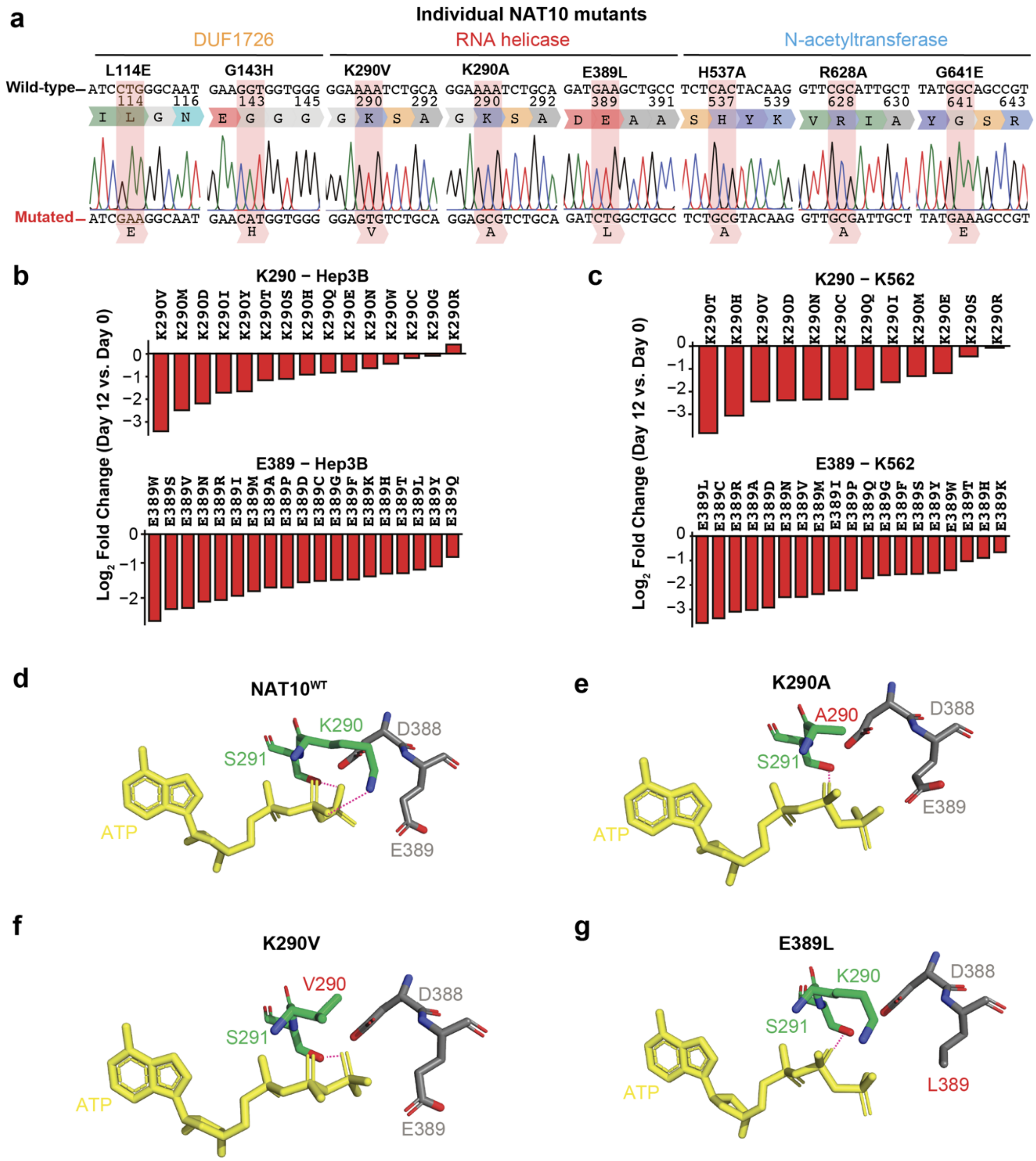

a Verification of single-site mutants by Sanger sequencing. **b,c** Bar plots showing the $\log_2$(fold change) in variant abundance (day 12 vs. day 0) from the deep mutational scanning assay for residues K290 and E389 in Hep3B (**b**) and K562 (**c**) cells. The data is derived from three biological replicates. **d-g** AlphaFold3 models depicting potential hydrogen bonding interactions between ATP and key residues (S291, K290, D388, and E389) in wild-type NAT10 (**d**), or the K290A (**e**), K290V (**f**), and E389L (**g**) mutants. For (b) and (c), source data are provided as a Source Data File.

Supplementary Fig. 4: Analysis of single DUF1726 and GNAT variants.

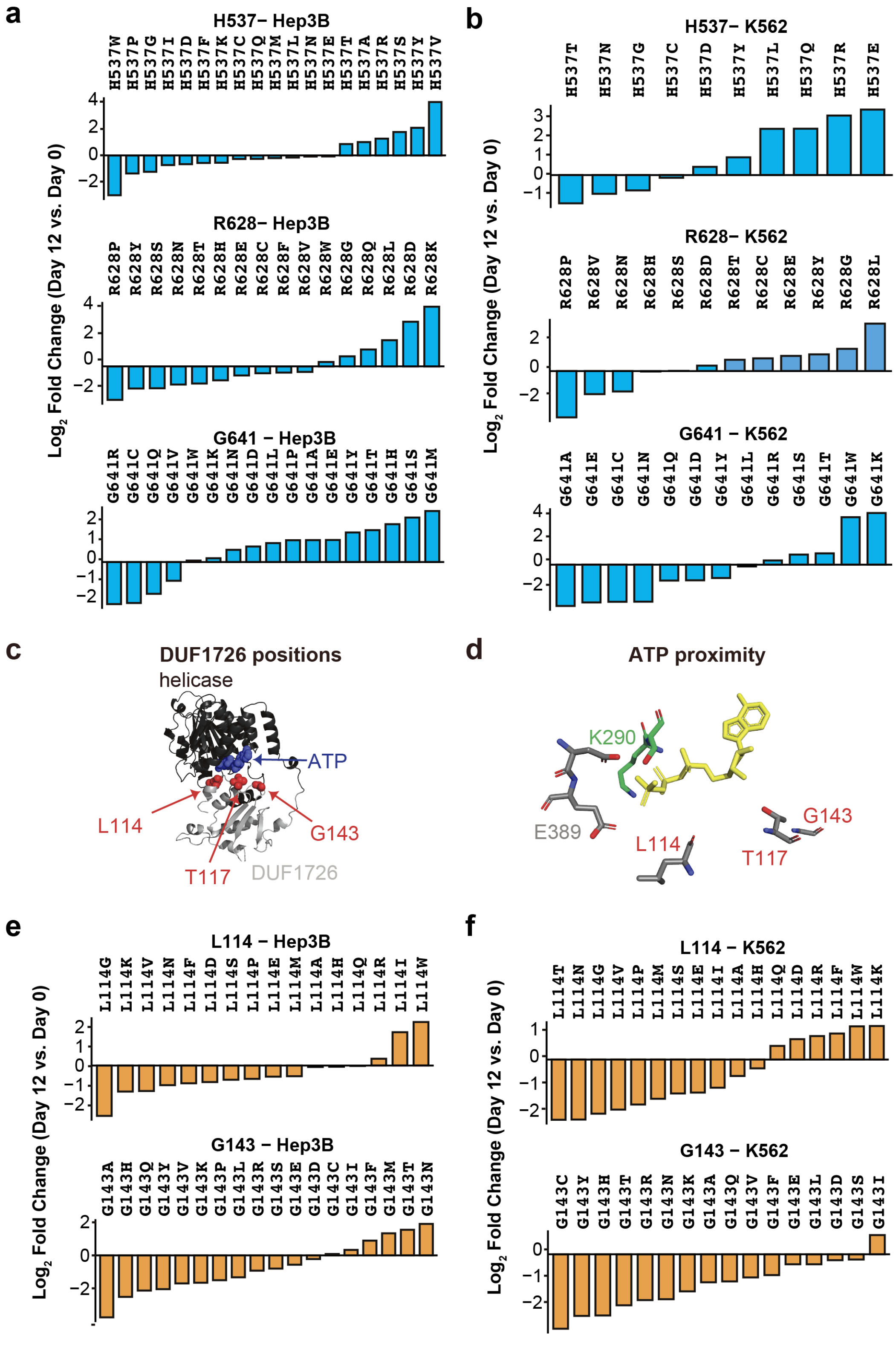

a-b Bar plots showing the log_2(fold change) in variant abundance (day 12 vs. day 0) for all detected substitutions at GNAT residues H537, R628, and G641 in Hep3B (**a**) and K562 (**b**) cells.

The data is derived from three biological replicates. **c** AlphaFold3 model showing the DUF1726 domain (gray) and RNA helicase domain (black) of NAT10 in complex with ATP (blue). Red spheres highlight residues L114, T117, and G143. **d** AlphaFold3 models depicting the relative positions of ATP (yellow) and residues K290 and E389 in the RNA helicase domain, as well as residues L114 and G143 in the DUF1726 domain. **e-f** Bar plots showing the log_2(fold change) in variant abundance (day 12 vs. day 0) for all detected substitutions at DUF1726 residues L114 and G143 in Hep3B (**e**) and K562 (**f**) cells. The data is derived from three biological replicates. For (a), (b), (e), and (f), source data are provided as a Source Data File.

Supplementary Fig. 5: Effect of single mutants on cell proliferation and viability.

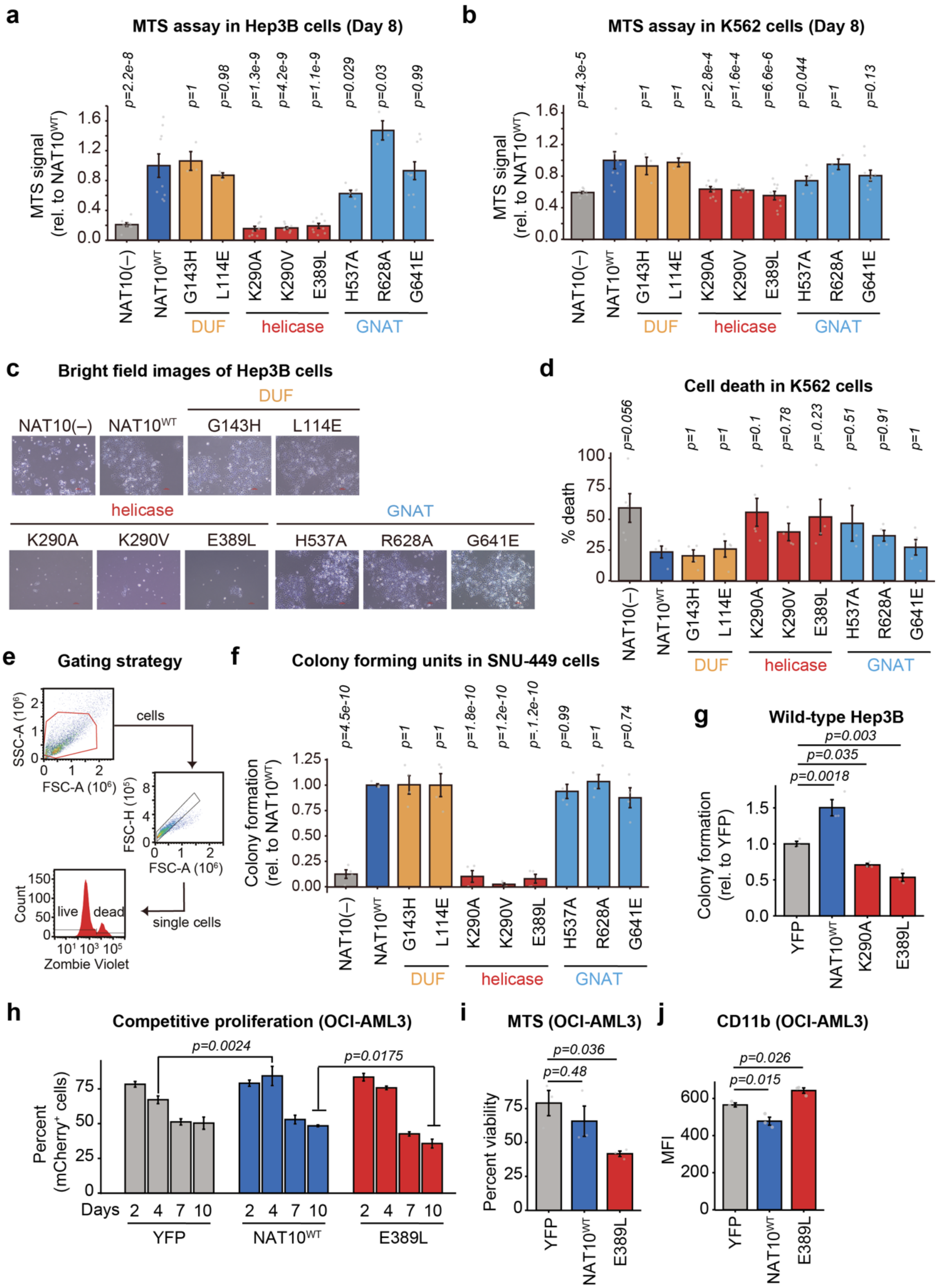

a,b Cell proliferation was evaluated as in Fig. 4c,d eight days after dTAG^{V}-1 treatment. Data represent mean ± SEM. n = 9 for NAT10(-), NAT10^{WT}, K290A, K290V, E389L, and G641E; n = 6

for H537A; n = 3 for G143H, L114E, and R628A. Each replicate corresponds to an independent transduction. p-values were calculated by one-way ANOVA with Dunnett's post-hoc test. **c** Bright-field microscopy images of $NAT10^{FKBP12-F36V}$ Hep3B cells expressing exogenous $NAT10^{WT}$ or the indicated mutants. The red scale bar is equivalent to 100 μm. Images are representative of three biological replicates. **d** Cell viability was measured via flow cytometry using the Zombie violet assay in $NAT10^{FKBP12-F36V}$ K562 cells expressing exogenous $NAT10^{WT}$ or the indicated NAT10 mutants 10 days after $dTAG^{V}$-1 treatment. Data represent mean ± SEM. n = 5 for NAT10(-), K290A, and G641E; n = 4 for $NAT10^{WT}$, G143H, L114E, K290V, E389L, and R628A; n=3 for H537A. Each biological replicate represents an independent transduction. *p*-values were calculated by one-way ANOVA with Dunnett's post-hoc test. **e** Gating strategy for cell death analysis via Zombie violet staining. Related to Figures 4g-h and Supplementary Figures 5d. **f** Colony formation assays in $NAT10^{FKBP12-F36V}$ SNU-449 cells expressing exogenous $NAT10^{WT}$ or the indicated NAT10 mutants. Data represent mean ± SEM. *n* = 4. Each biological replicate represents an independent transduction. *p*-values were calculated by one-way ANOVA with Dunnett's post-hoc test. **g** Colony formation assays in wild-type Hep3B cells expressing exogenous YFP, $NAT10^{WT}$, or the indicated NAT10 mutants. Data represent mean ± SEM. *n* = 3. Each biological replicate represents an independent transduction. *p*-values were calculated by one-way ANOVA with Dunnett's post-hoc test. **h** Wild-type OCI-AML3 cells were transduced with YFP, $NAT10^{WT}$, or $NAT10^{E389L}$ expressing vectors, followed by Blasticidin selection. The percent of mCherry+ cells was assessed by flow cytometry at different time points post-selection. Data represent mean ± SEM. *n* = 3 from three biological replicates. *p*-values were calculated by two-way ANOVA with Holm's post-hoc corrections. **i** Wild-type OCI-AML3 cells were transduced with YFP, $NAT10^{WT}$, or $NAT10^{E389L}$ expressing vectors, followed by Blasticidin selection. MTS viability assays were performed three days post-selection. Data represent mean ± SEM of percent viability relative to untransduced cells. *n* = 3 from three biological replicates. *p*-values were calculated by one-way ANOVA with Dunnett's post-hoc test. **j** Median Fluorescence Intensity (MFI) of CD11b expression in wild-type OCI-AML3 cells expressing exogenous YFP, $NAT10^{WT}$, or $NAT10^{E389L}$. Data represent mean ± SEM of percent viability relative to untransduced cells. *n* = 3 from three biological replicates. *p*-values were calculated by one-way ANOVA with Dunnett's post-hoc test. For (a-b) and (d-i), source data are provided as a Source Data File. For (c), images are presented as raw files.

Supplementary Fig. 6: Effect of NAT10 depletion on cancer growth *in vivo*.

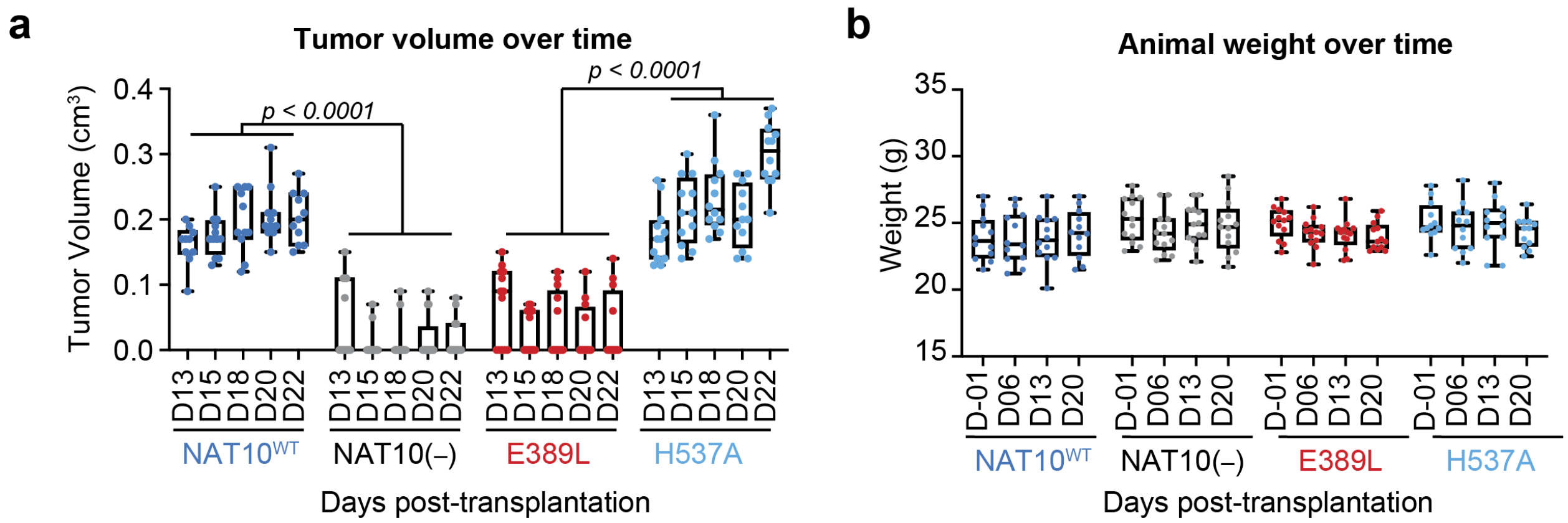

a Tumor volume over time in NSG mice injected with Hep3B cells expressing $NAT10^{WT}$, YFP [NAT10(–)], $NAT10^{E389L}$, or $NAT10^{H537A}$, as shown in Fig. 5a ($NAT10^{WT}$, n = 11; $NAT10^{E389L}$, n = 13; $NAT10^{H537A}$, n = 12; NAT10(–), n = 12). Cells were pre-treated with 100 nM $dTAG^{V}$-1 for 48 h prior to transplantation, and $dTAG^{V}$-1 was co-administered on the day of injection. Tumor volume was calculated as (length × width²)/2 and plotted as mean ± SD. Growth curves were analyzed using a mixed-effects model with Greenhouse-Geisser correction. Tukey's post hoc test was used for pairwise comparisons at each time point. Box plots show the median, the interquartile range (the box), and whiskers and whiskers extending to the minimum and maximum values. **b** Body weights of the mice described in (**a**), monitored prior to transplantation (day –1) and at serial intervals post-injection (days 6, 13, and 20). Two-way repeated measures ANOVA with Greenhouse-Geisser correction revealed no significant effect of genotype (p = 0.3152), time (p = 0.0591), or genotype × time interaction (p = 0.4591). Inter-subject variability accounted for the majority of variance (p < 0.0001), indicating stable body weights across groups. Box plots show the median, the interquartile range (the box), and whiskers and whiskers extending to the minimum and maximum values. For a-b, source data are provided as a Source Data File.

Supplementary Fig. 7: Effect of NAT10 depletion in hematopoietic cells.

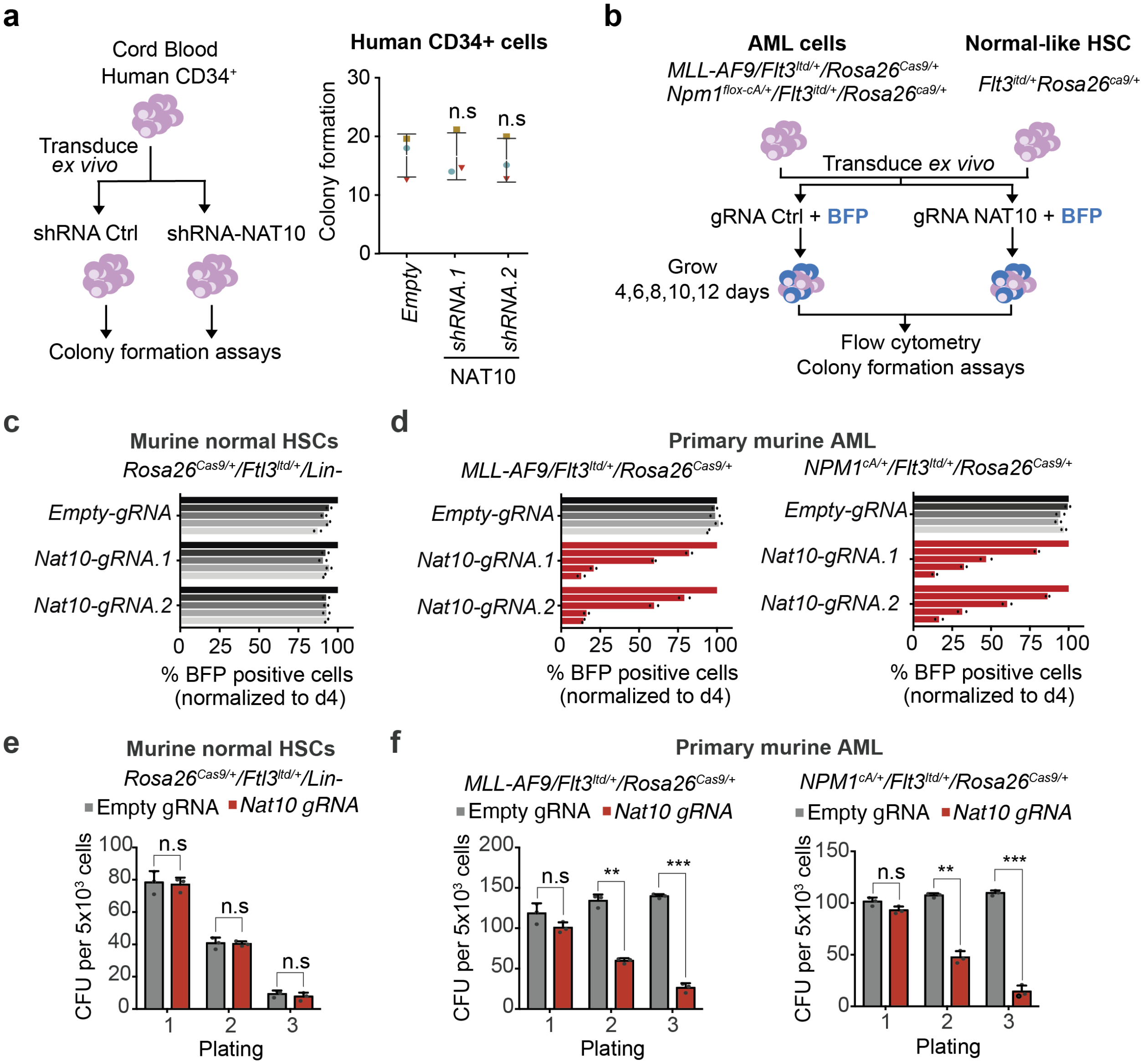

a Colony-forming efficiency of human CD34^{+} cord blood cells transduced *ex-vivo* with NAT10-targeting shRNAs or a scrambled control (mean ± SD, $n = 3$, independent biological replicates). No significant differences were detected by one-way ANOVA with repeated measures. n.s., not significant. **b** Schematic of colony formation and competitive proliferation assays in murine non-cancerous (*Flt3$^{itd/+}$/Rosa26$^{cas9/+}$*) or leukemic (*NPM1$^{flox\text{-}cA/+}$/Flt3$^{itd/+}$/Rosa26$^{cas9/+}$* and *MLL-AF9 /Flt3$^{itd/+}$/Rosa26$^{cas9/+}$*) hematopoietic stem cells transduced *ex-vivo* with NAT10-targeting gRNAs or a control gRNA. **c,d** Competitive proliferation assays were performed by calculating the percentage of BFP+ cells via flow cytometry at various time points post-transduction. The order of the bars within each group is day 4, 6, 8, 10, and 12, respectively. Data represent the Mean from two independent biological replicates. **e-f** CFU, colony-forming units; n=3, independent biological replicates. n.s., not significant; $*p < 0.05$, two-tailed unpaired t-test. NAT10 ablation decreased cell proliferation in leukemic cells (d and f) but not in non-cancer cells (c and e). For (a) and (c-f), source data are provided as a Source Data File.

Supplementary Fig. 8: Effect of NAT10 depletion in normal liver cells.

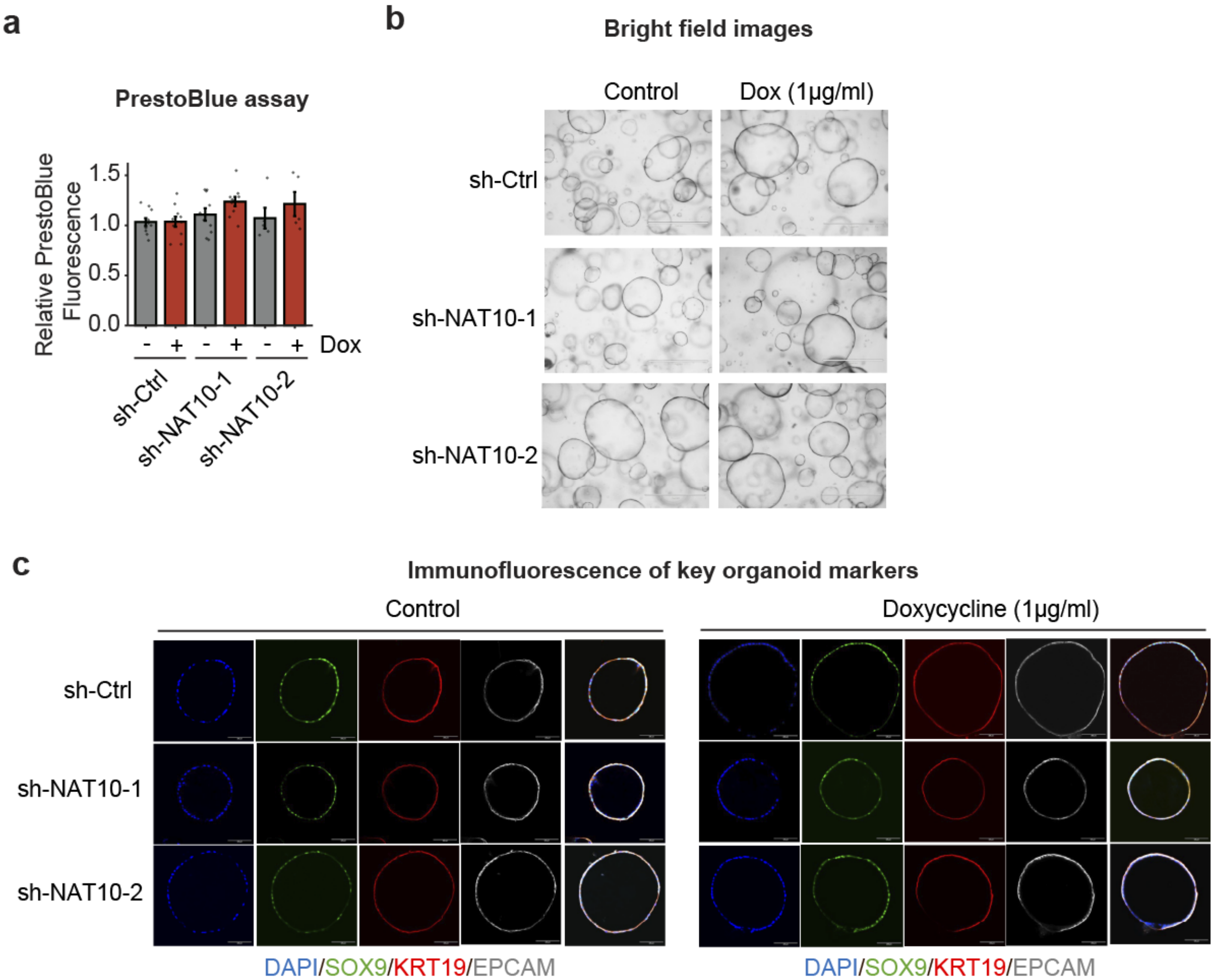

a Intrahepatic Cholangiocyte Organoids (ICOs) were transduced with two different doxycycline-inducible shRNAs against NAT10 and a shRNA-control, followed by puromycin selection. shRNA expression was induced by doxycycline treatment five days after splitting, and the PrestoBlue assay was used to assess organoid size at day seven. Data represent mean ± SEM. n = 10 for sh-Ctrl and shNAT10-1, n=5 for shNAT10-2. Source data are provided as a Source Data File. **b** Bright field images of ICOs from (**a**). Scale bar: 1 mm. Images are representative of three biological replicates. **c** Confirmation of organoid differentiation by Immunofluorescence of key organoid markers after seven days of culture. Scale bar: 100 µm. Immunofluorescence images were captured from two independent biological replicates.

Supplementary Fig. 9: ac⁴C mapping.

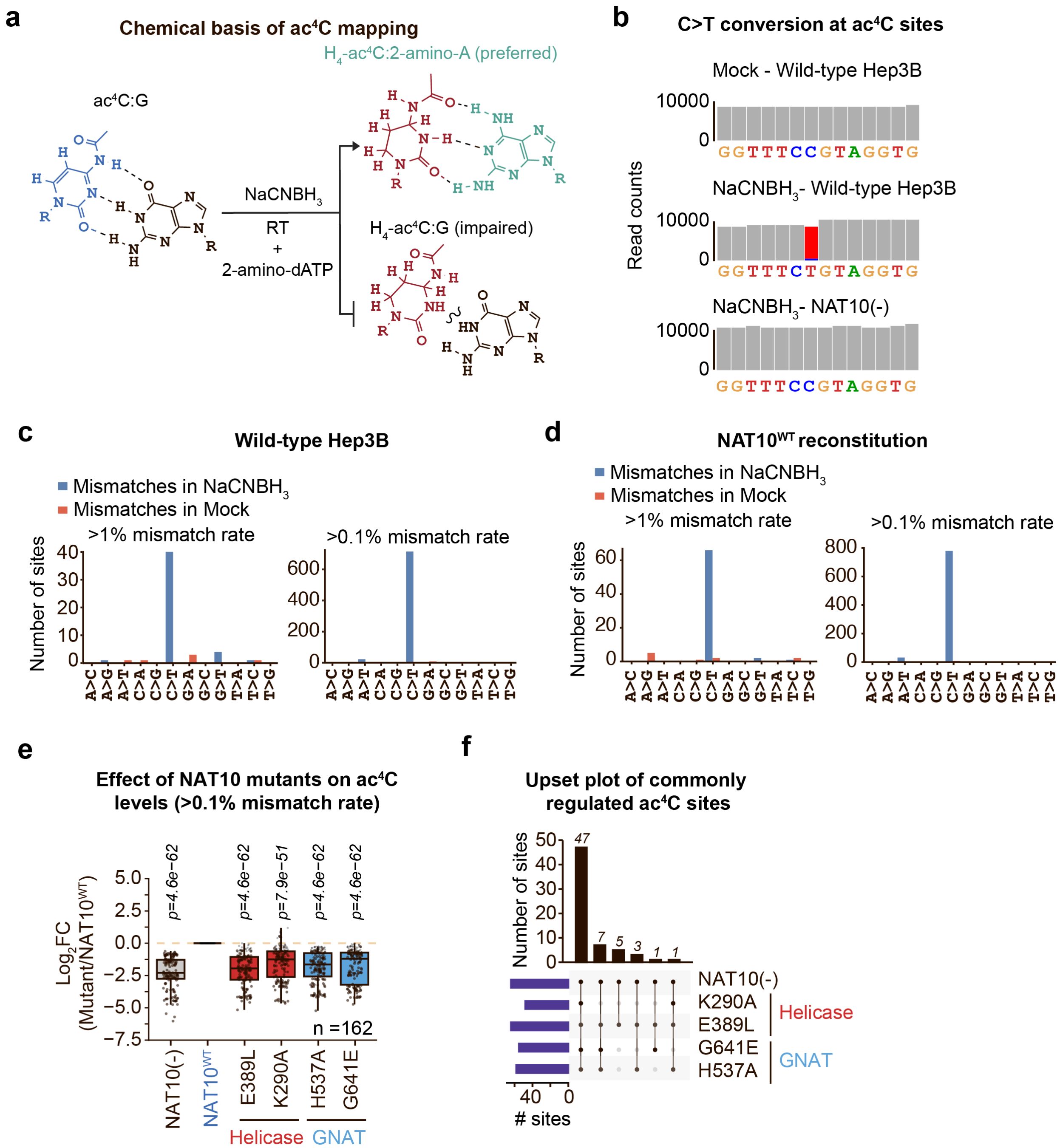

a Base pair preference of tetrahydro-ac⁴C following reduction with $NaCNBH_3$ and reverse transcription with 2-amino-dATP. **b** Browser view indicating a conversion rate of ~90% in the ac⁴C site of helix 45 in 18S rRNA from wild-type Hep3B cells treated with $NaCNBH_3$. Total RNA from NAT10(–) cells or mock-treated wild-type cells is used as a control. This site is known to have near 100% stoichiometry in mature 18S rRNA. **c,d** Frequency of all mismatch types significantly enriched in $NaCNBH_3$-treated or mock-treated samples relative to each other. Sites must be independently obtained in two biological replicates to be included in these plots. Two thresholds were applied: a mismatch rate greater than 1% or greater than 0.1%. A specific enrichment for C>T mismatches was observed in both filtering schemes. Importantly, the results were

reproducible in Wild-type Hep3B cells, and NAT10$^{FKBP12\text{-}F36V}$ cells reconstituted with NAT10^{WT}. **e** ac^{4}C mapping was performed in two independent biological replicates per clone in two independent clones per group. Sites significantly decreased in NAT10(–) cells compared to NAT10$^{FKBP12\text{-}F36V}$ cells reconstituted with NAT10^{WT} were selected as candidate ac^{4}C sites. This analysis identified 162 ac^{4}C loci with mismatch rates higher than 0.1%. Box plots represent the relative ac^{4}C levels of the 162 sites in the RNA helicase and GNAT mutants relative to NAT10^{WT}. Box plots show the median, the interquartile range (the box), and whiskers extending to 1.5 times the interquartile range. p-values were calculated by a two-sided Wilcoxon rank-sum test. **f** Upset plot comparing the number of ac^{4}C sites significantly decreased in the NAT10(–), the RNA helicase mutants, and the GNAT mutant conditions. ac^{4}C mapping was performed in two independent biological replicates per clone in two independent clones per group. For (b-f), source data are provided as a Source Data File.

Supplementary Fig. 10: The RNA helicase domain of NAT10 is required for 40S biogenesis.

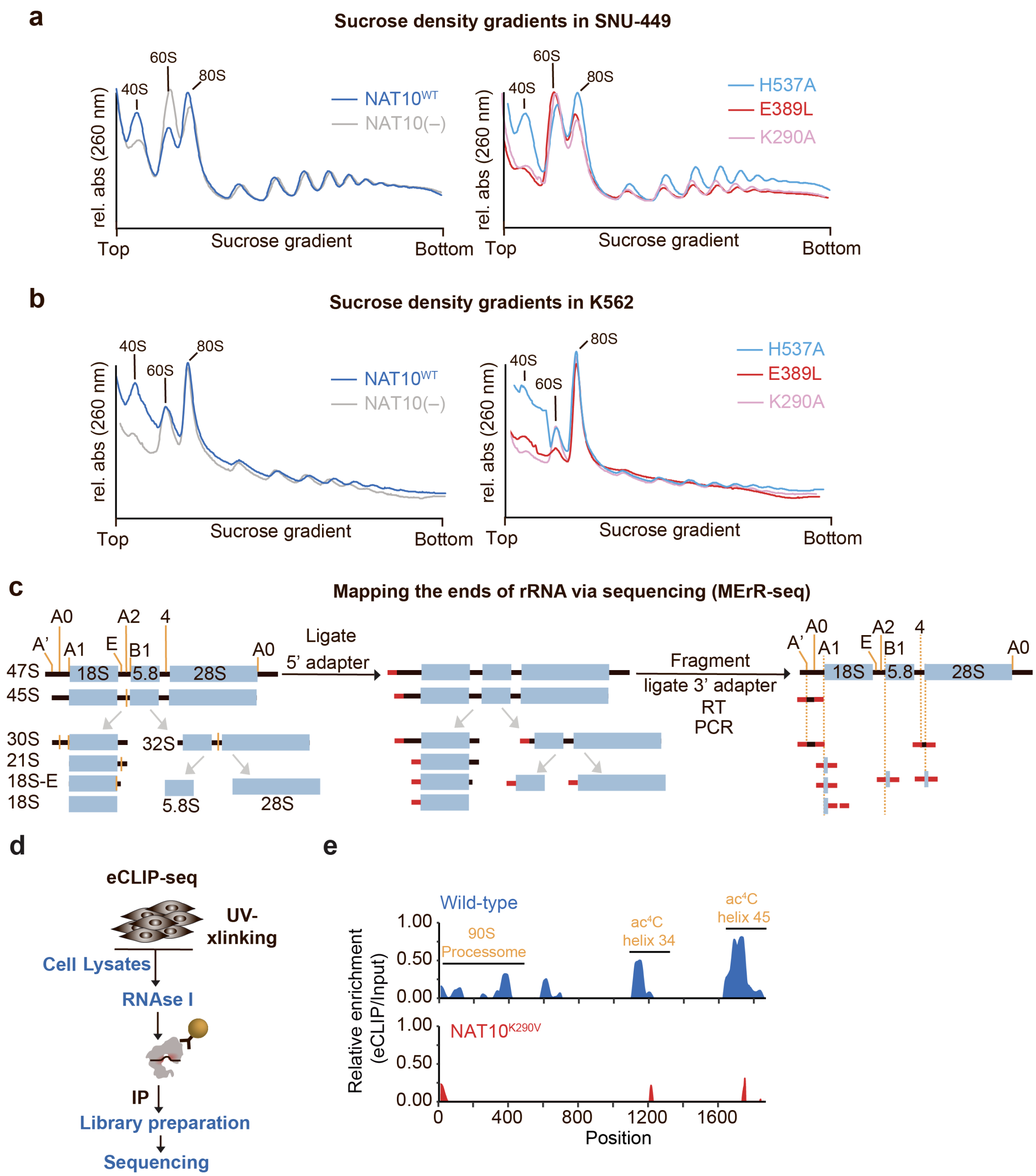

a-b Ribosome profiles from sucrose density gradients in NAT10$^{FKBP12\text{-}F36V}$ SNU-449 (**a**) or K562 (**b**) cells expressing NAT10^{WT} or the indicated mutants. **c** Schematic of MErR-seq workflow. A 5′ RNA adapter was ligated to intact rRNA prior to fragmentation, followed by 3′ adapter ligation, reverse transcription, and PCR amplification. **d** Schematic of eCLIP-seq workflow. **e** Genome browser views showing eCLIP-seq density peaks from NAT10$^{FKBP12\text{-}F36V}$ Hep3B cells expressing exogenous NAT10^{K290V} or wild-type Hep3B cells. The values correspond to the average of three independent biological replicates. For a, b, and e, source data are provided as a Source Data File.

Supplementary Fig. 11: NAT10 regulates the deposition of m^{1}acp^{3}Ψ.

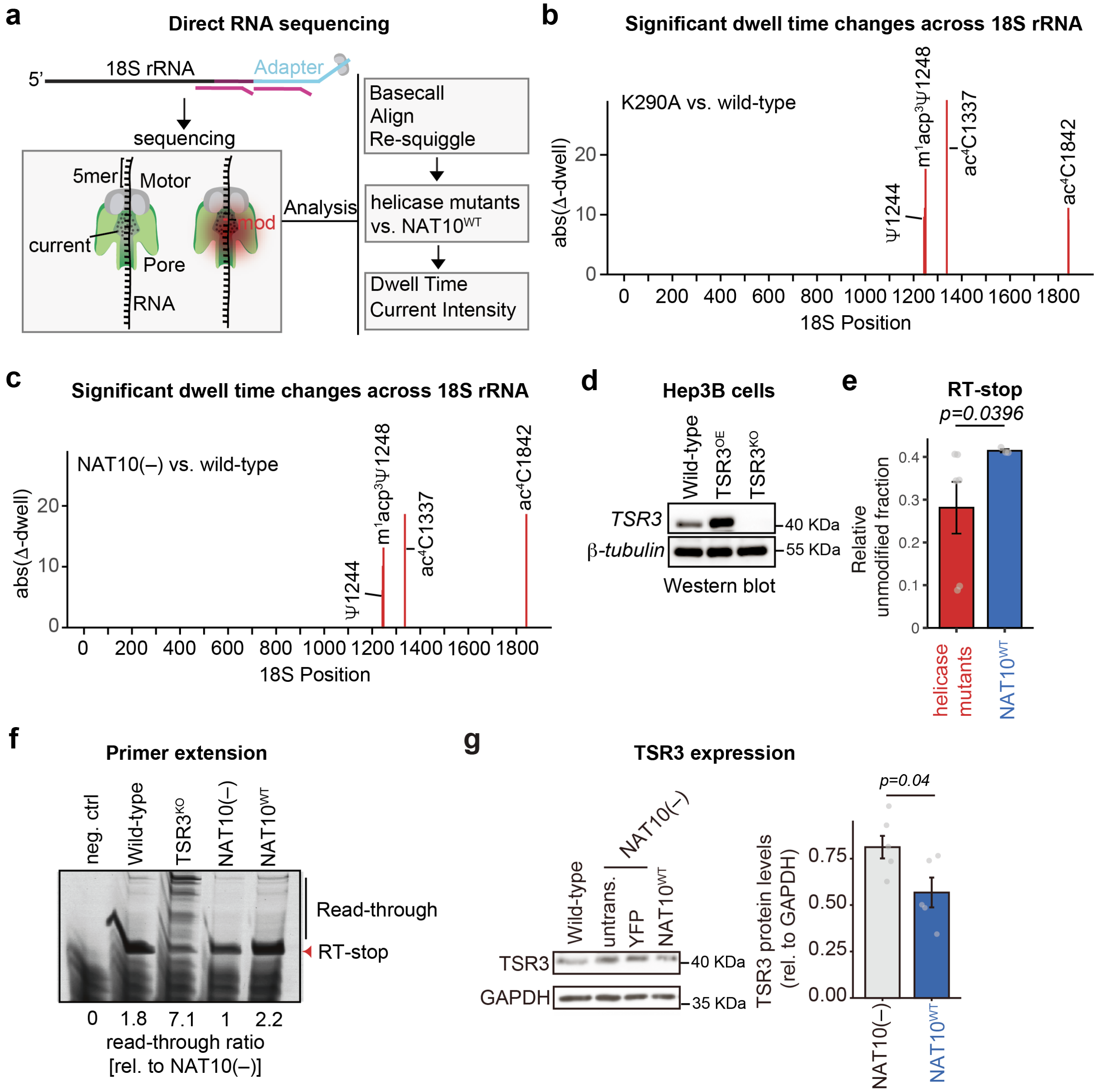

a Schematic of direct RNA sequencing of 18S rRNA in NAT10$^{FKBP12-F36V}$ Hep3B cells expressing wild-type NAT10 or the indicated mutants. **b,c** Absolute differences in dwell time across the 18S rRNA transcript, comparing wild-type NAT10 and either the helicase mutant K290A (**b**) or NAT10(–) (**c**). Red Positions show sites that exhibited consistent, statistically significant differences across three independent biological groups [NAT10^{K290A}, NAT10^{E389L}, and NAT10(–)] relative to NAT10^{WT} in two independent biological replicates per group. Exact *p*-values are reported in Supplementary Data 6. **d** Western blot verification of TSR3 overexpression (OE) and knockout (KO) in Hep3B cells. Blots are representative of three biological replicates. **e** Quantitation of the eCLIP-seq data from Fig. 7h. All RNA helicase data points were grouped together. *p*-values were calculated using a one-tailed Welch's t-test. n=3 for NAT10^{WT}, n=6 for helicase mutatns. **f** RT-stop assay using a fluorescently labeled primer downstream of the

$m^1acp^3\Psi$ site. The primer extends until it encounters $m^1acp^3\Psi$. A higher proportion of read-through indicates a higher ratio of unmodified 18S rRNA. The read-through ratio [relative to NAT10(–)] is shown at the bottom. The experiment was performed in two biological replicates. **g** Western blot verification of TSR3 expression in $NAT10^{FKBP12\text{-}F36V}$ Hep3B cells expressing exogenous $NAT10^{WT}$ or YFP [NAT10(–)]. Wildtype or untransduced $NAT10^{FKBP12\text{-}F36V}$ Hep3B cells were used as controls. Bar plots represent mean ± SEM. $n = 5$. p-values were calculated using a two-tailed Student's t-test. For (b-g), source data are provided as a Source Data File.

Supplementary Fig. 12: Regulation of m¹acp³Ψ by cytoplasmic NAT10

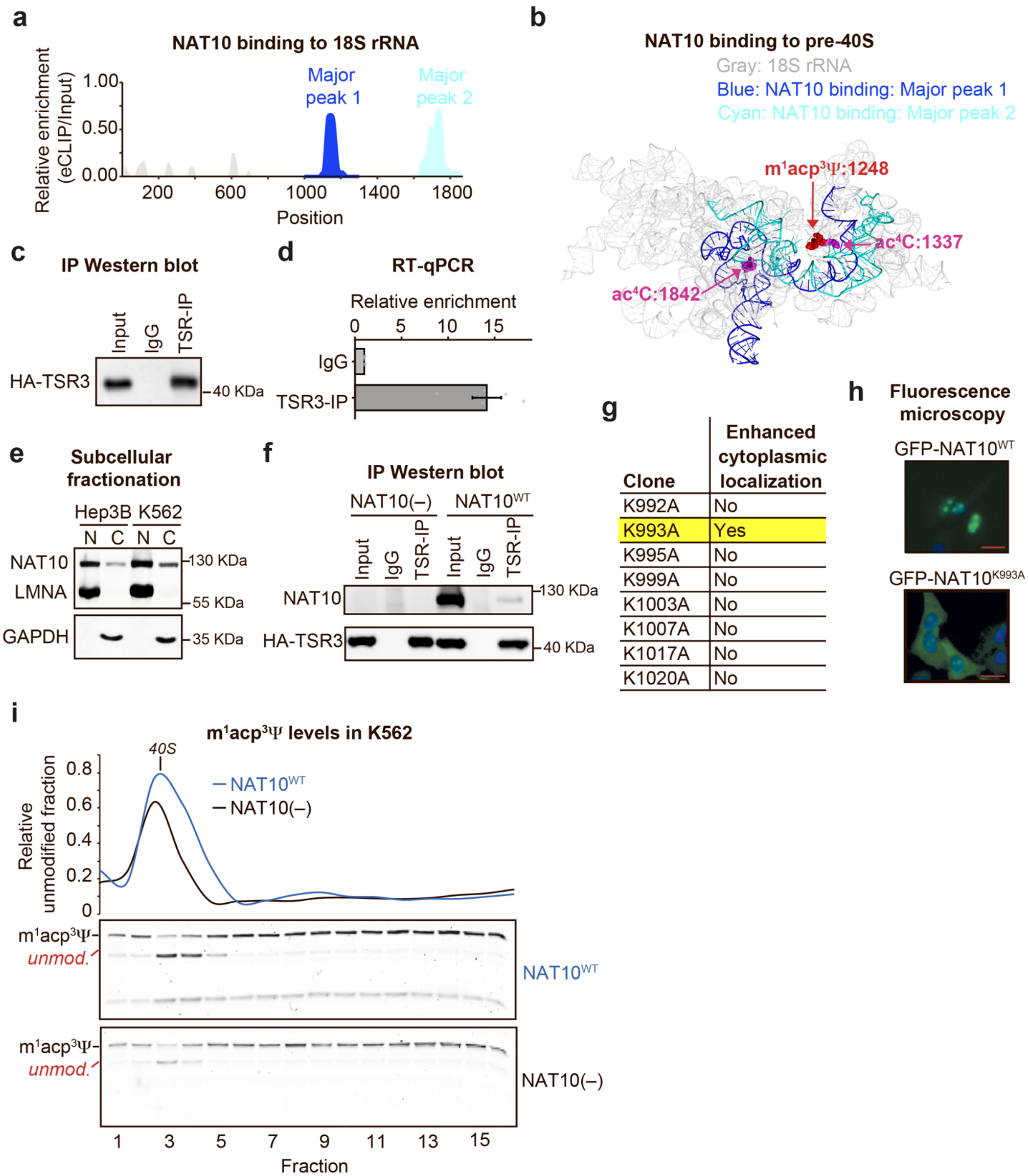

Clone	Enhanced cytoplasmic localization
K992A	No
K993A	Yes
K995A	No
K999A	No
K1003A	No
K1007A	No
K1017A	No
K1020A	No

a Browser views from NAT10^{WT} cells shown in Fig. 7h, colored to highlight the two major peaks across 18S rRNA. **b** structural visualization of the two major NAT10 binding peaks on the pre-40S structure from PDB: 6ZXH,[84] highlighting the two ac⁴C sites and the m¹acp³Ψ site in 18S rRNA. **c,d** RIP-RT-qPCR was performed by immunoprecipitating (IP) HA-TSR3 with anti-HA antibodies from knockout cells expressing exogenous HA-TSR3. Isotypic IgG was used as a control.

Western blot with anti-HA antibodies was used to determine the efficiency of IP (**c**), whereas RT-qPCR was used to determine enrichment of 18S rRNA relative to the IgG control (**d**). Data in (**d**) represent mean ± SEM. *n* = 5. **e** Subcellular fractionation was performed to isolate nuclear and cytoplasmic fractions. The efficiency of fractionation was assessed by Western blotting with anti-LMNA antibodies as a nuclear (N) marker and anti-GAPDH antibodies as a cytoplasmic (C) marker. anti-NAT10 antibodies were used to visualize the relative levels of NAT10 in either fraction. The blots are representative of three biological replicates. **f** Co-immunoprecipitation in TSR3 knockout cells expressing exogenous HA-TSR3, $NAT10^{WT}$ or depleted of NAT10. IPs were performed with anti-HA antibodies. Isotypic IgG was used as a control. Western blot with anti-HA antibodies was used to assess the efficiency of the IP. anti-NAT10 antibodies were used to determine NAT10 co-immunoprecipitation with TSR3. Experiments were performed in two biological replicates for NAT10(–) and $NAT10^{WT}$ clones, and two additional biological replicates in NAT10(–) and wild-type Hep3B cells. **g-h** a GFP-tagged NAT10 was systematically mutated at multiple lysine residues in the nucleolar localization signal of NAT10. Clones were transfected into HEK293T cells and scanned via fluorescence microscopy to evaluate changes in the subcellular localization of NAT10. K993A was the only mutation that enhanced the cytoplasmic localization of NAT10. The screen on (g) was performed once. Verification of K993A in (g) and (h) by fluorescence microscopy was performed in three biological replicates. Scale bar: 50 μm. **i** Relative unmodified levels calculated by densitometry as unmodified/(modified + unmodified) in $NAT10^{FKBP12\text{-}F36V}$ K562 cells expressing exogenous $NAT10^{WT}$ or YFP [NAT10(–)]. Lower panels: *HinFI* digestion of 18S rRNA amplicons from each gradient fraction. Each lane represents a different fraction. The experiment was performed with two biological replicates for K562 cells and three biological replicates for Hep3B cells. Uncropped blots are provided as a Source Data File.

Uncropped blot from Supplementary Fig. 11d

Dotted red region is the image used in manuscript

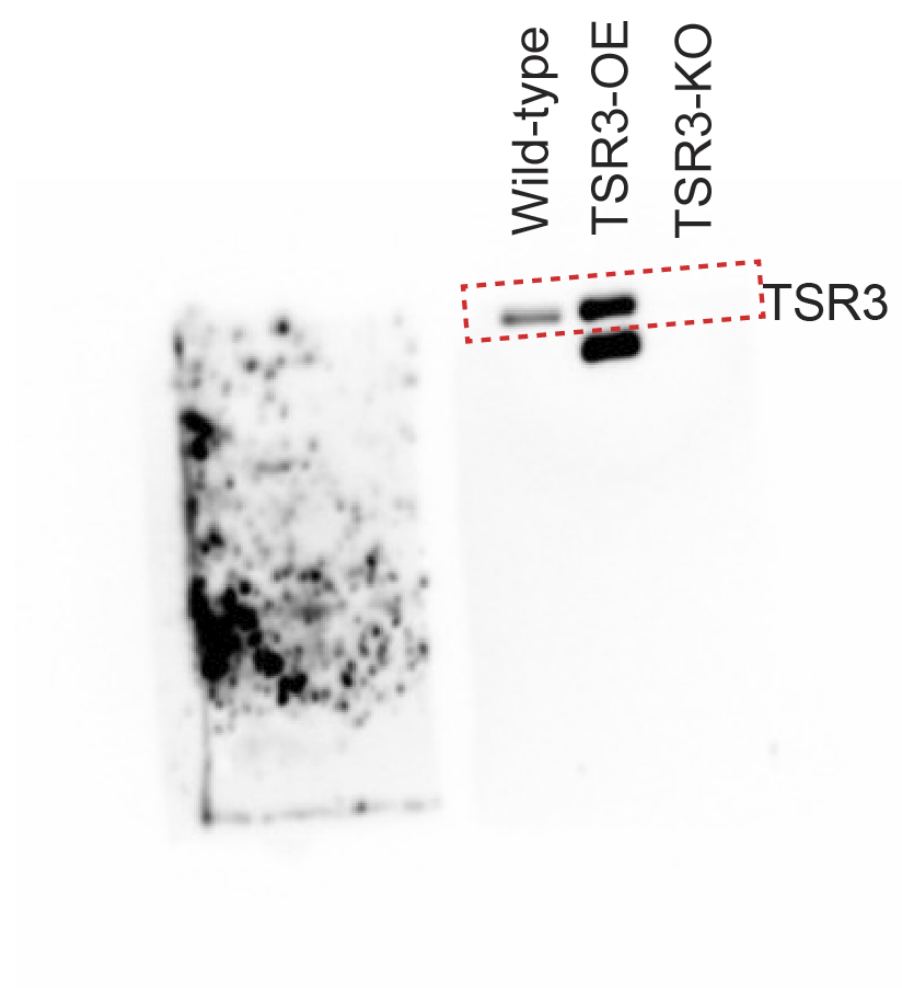

Overlay with ladder

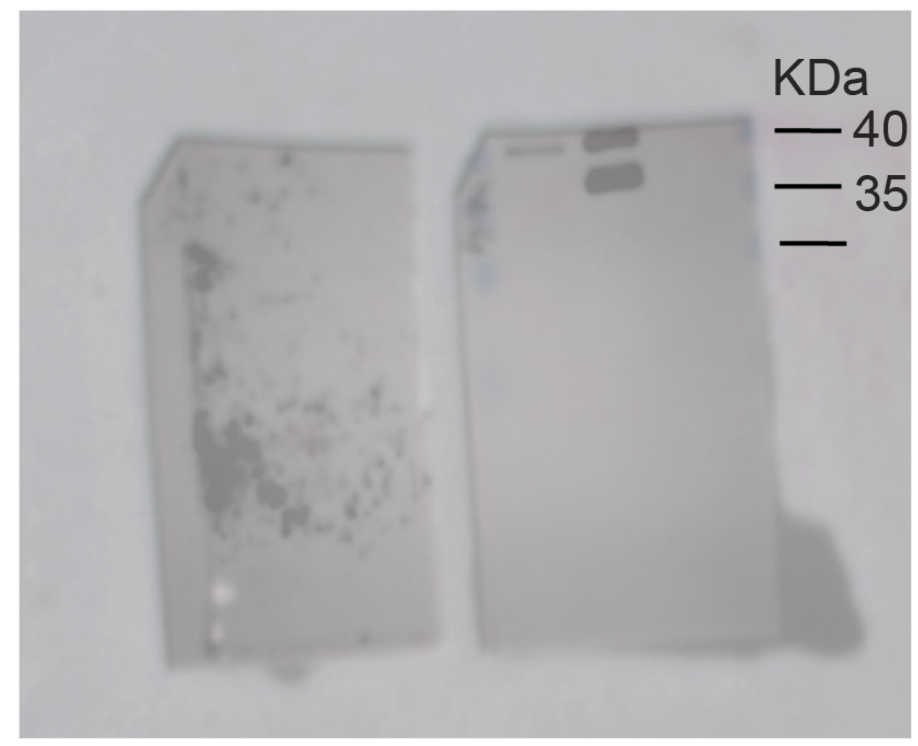

Dotted red region is the image used in manuscript

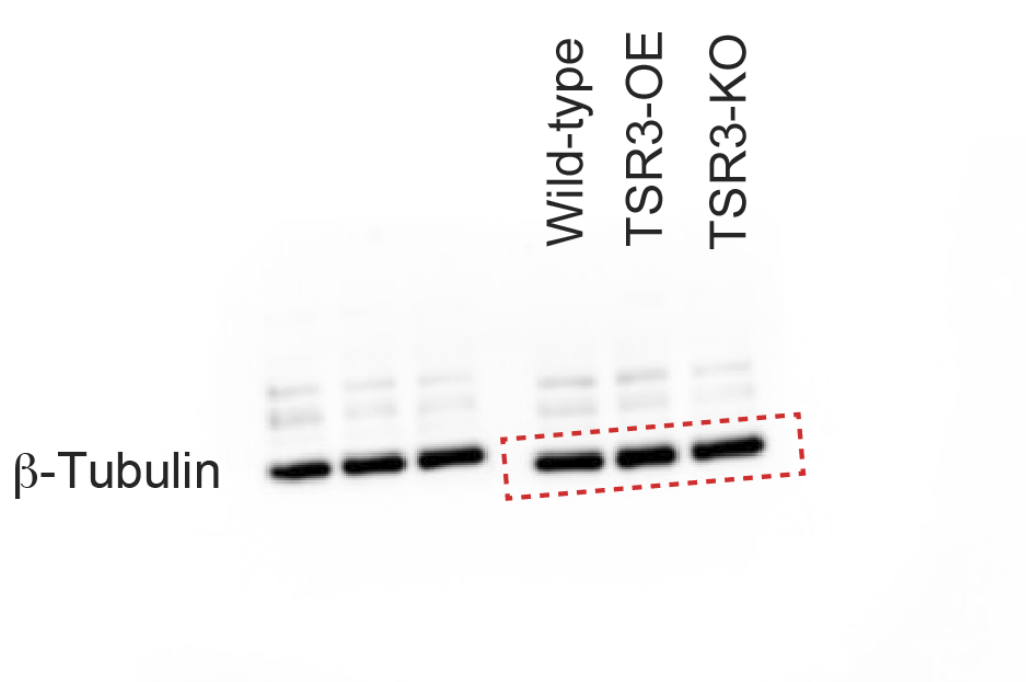

Overlay with ladder

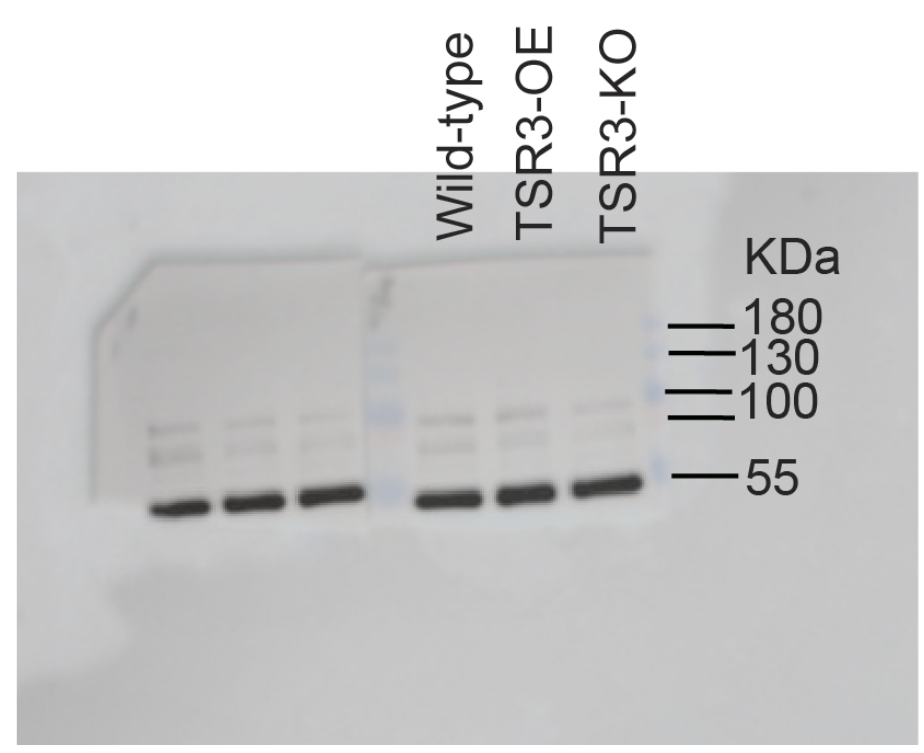

Uncropped gel from Supplementary Fig. 11f

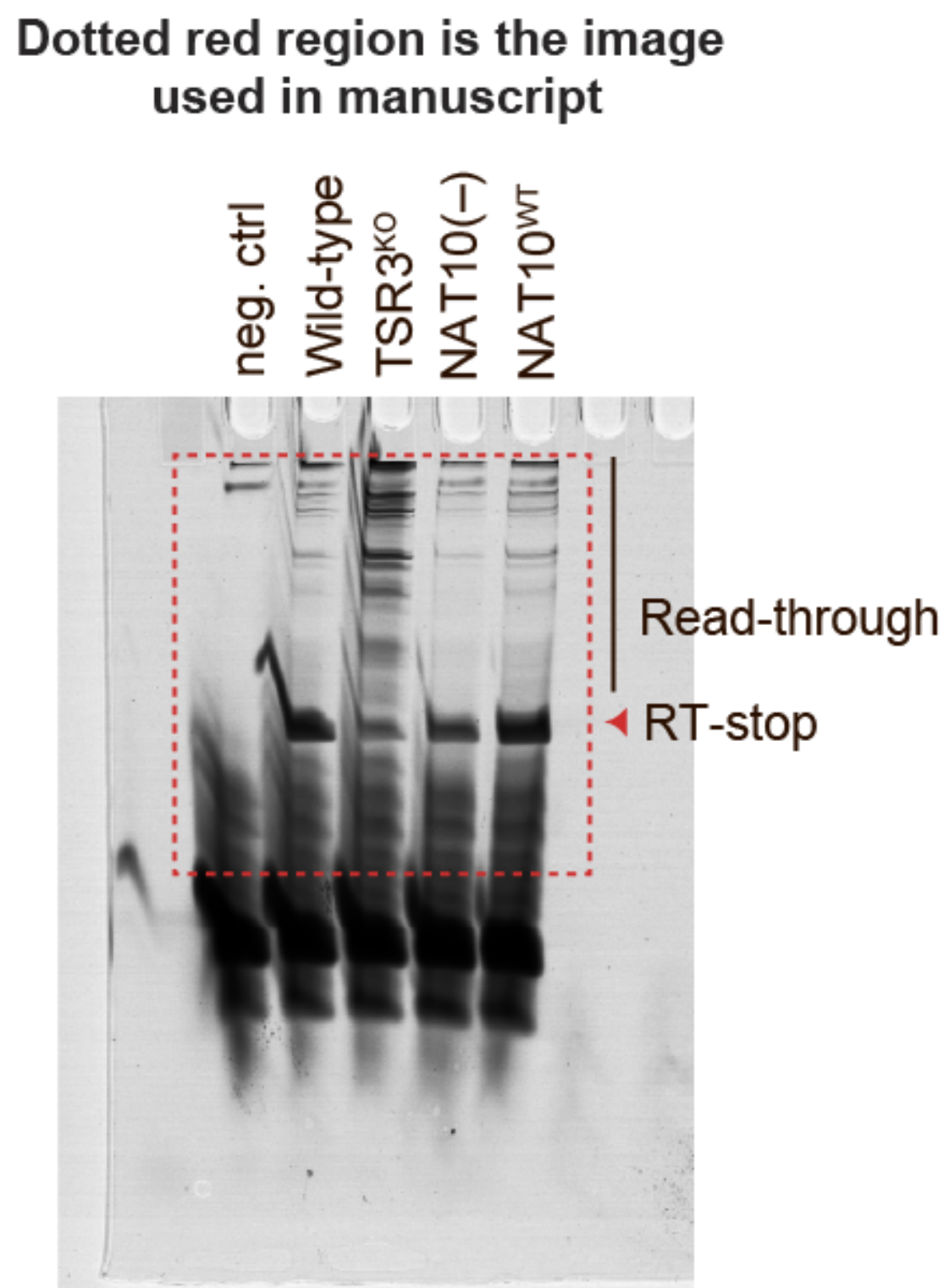

Uncropped blot from Supplementary Fig. 11g

Dotted red region is the image used in manuscript

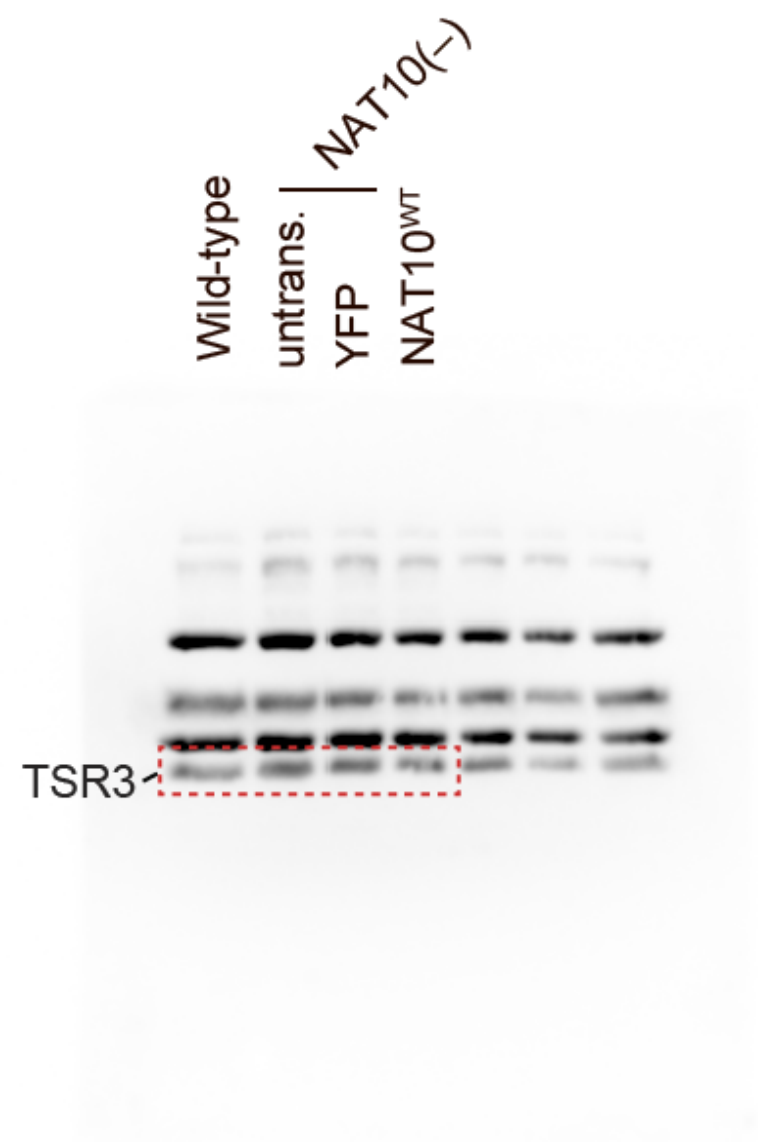

Overlay with ladder

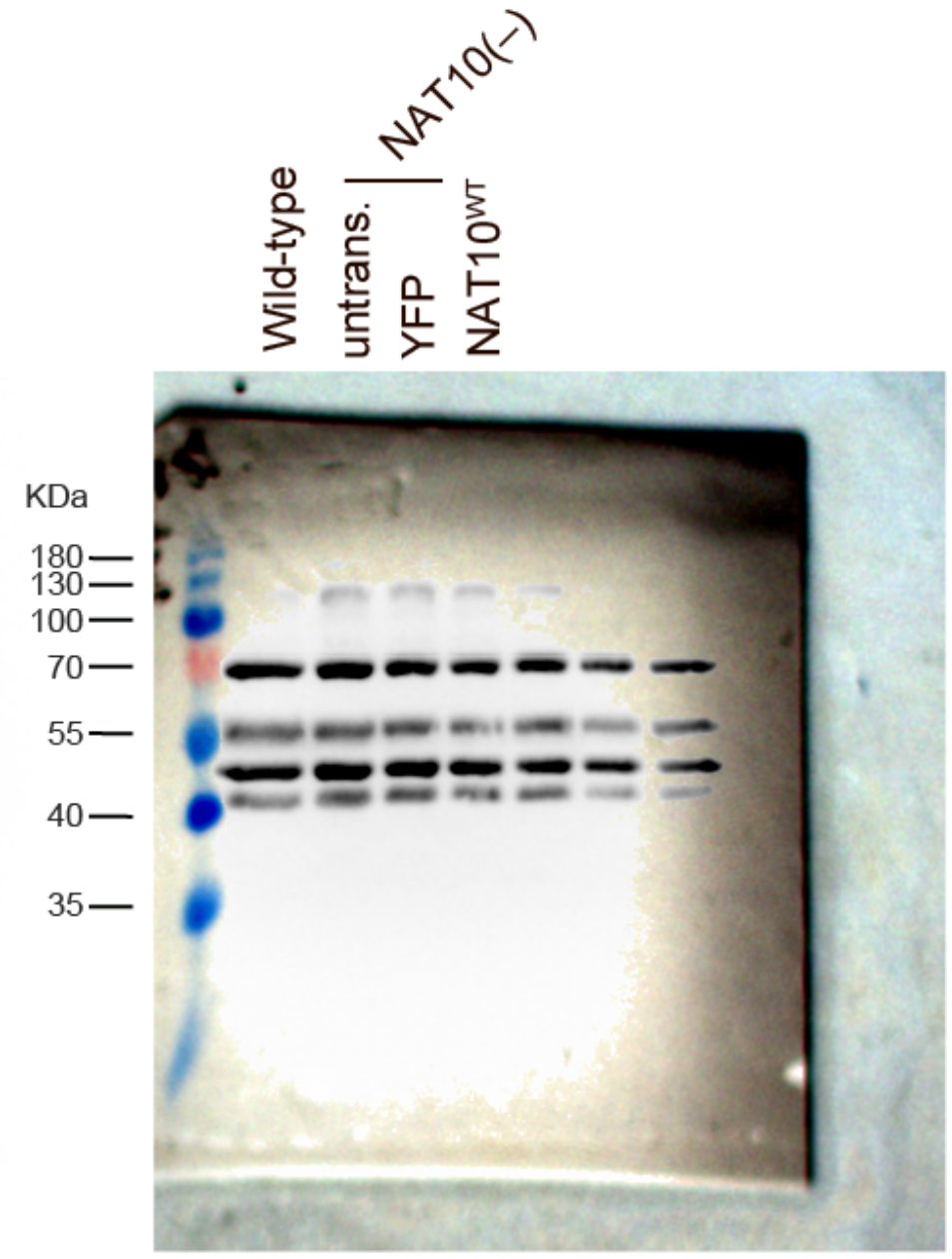

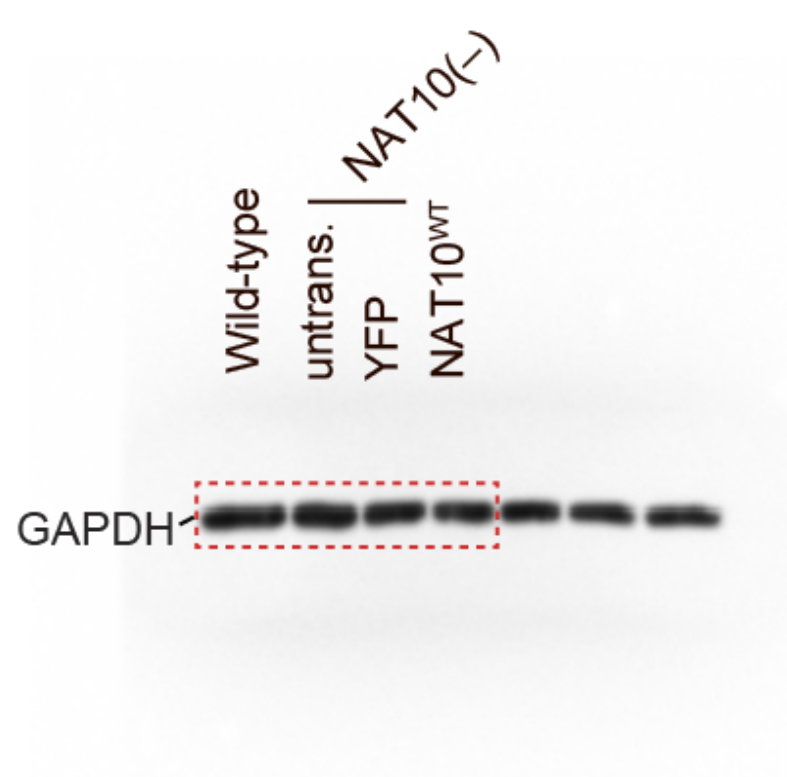

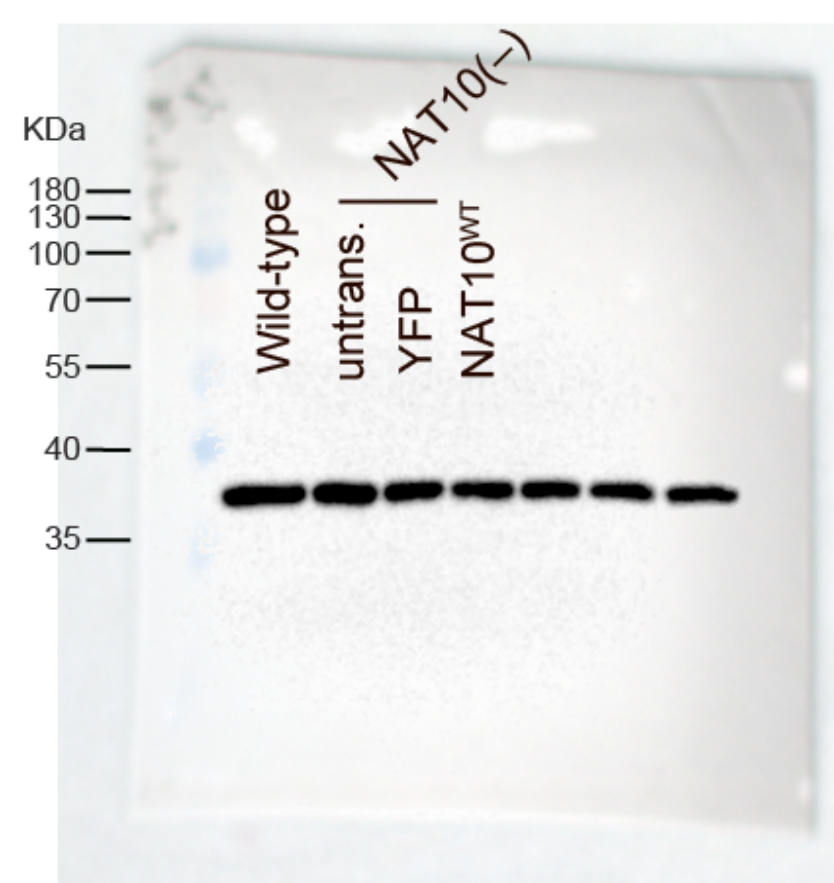

Validation of TSR3 antibody

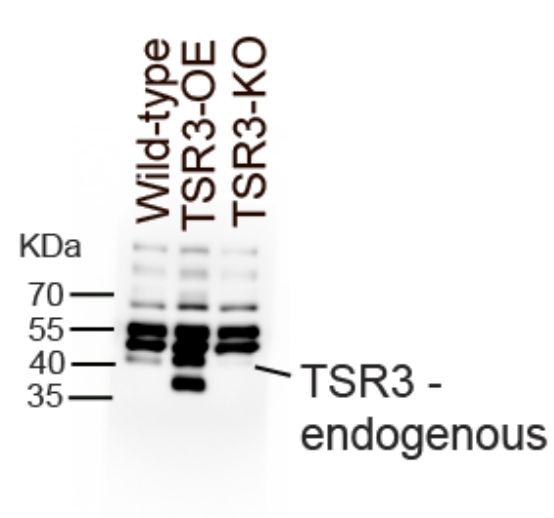

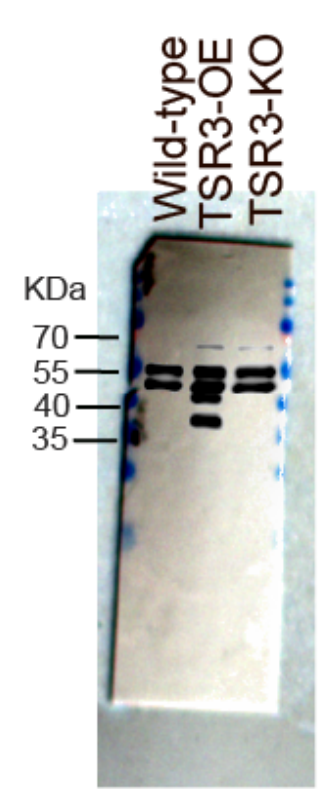

Uncropped blot from Supplementary Fig. 12c

Dotted red region is the image used in manuscript

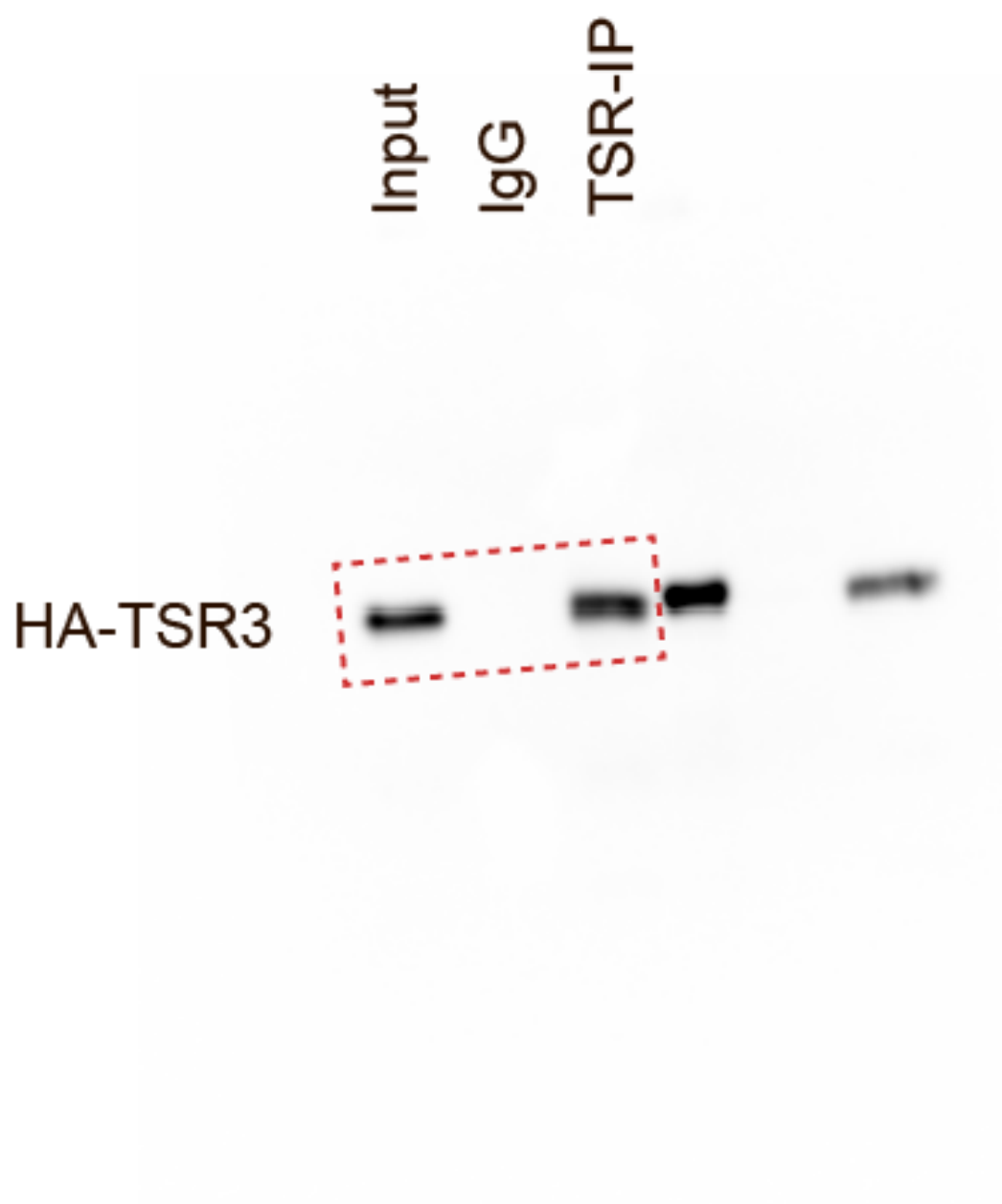

Overlay with ladder

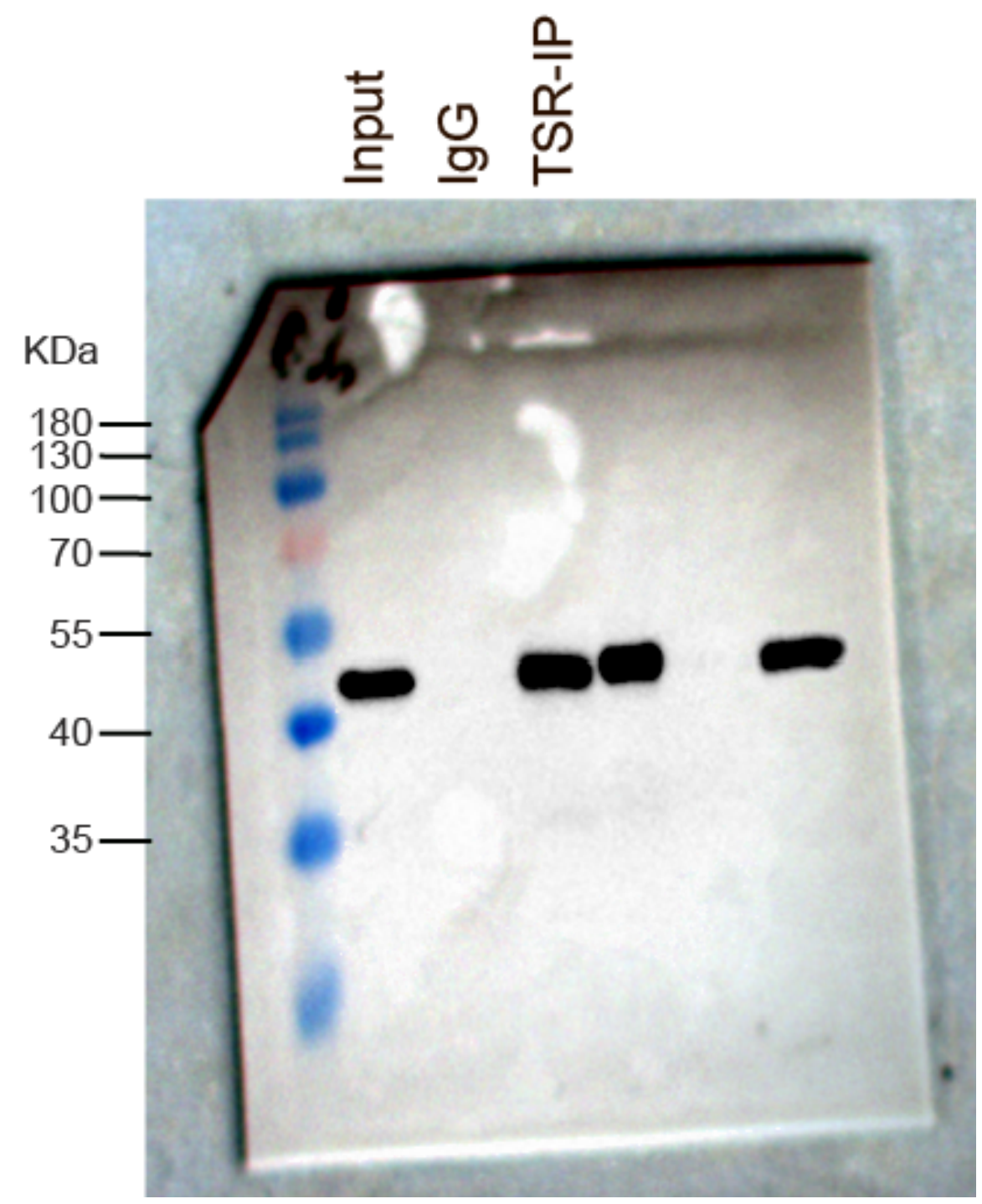

Uncropped blot from Supplementary Fig. 12e

Overlay with ladder

Dotted red region is the image used in manuscript

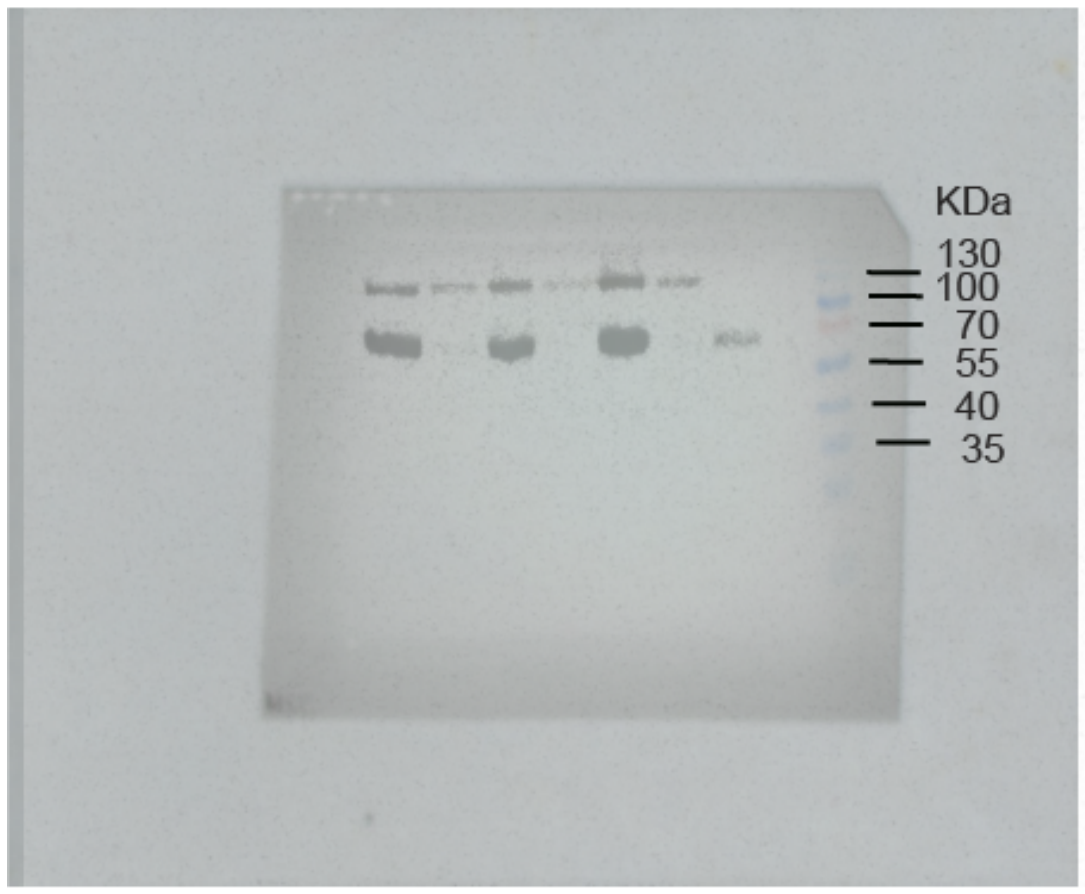

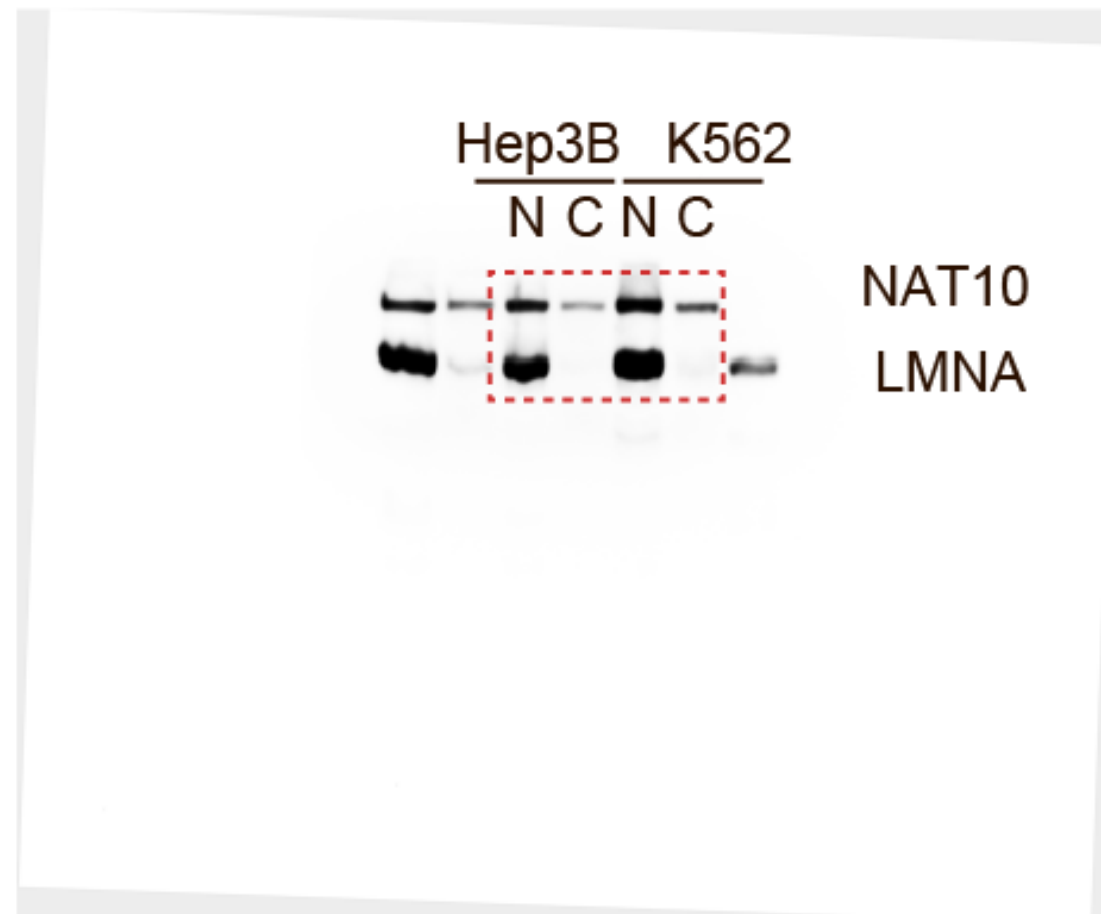

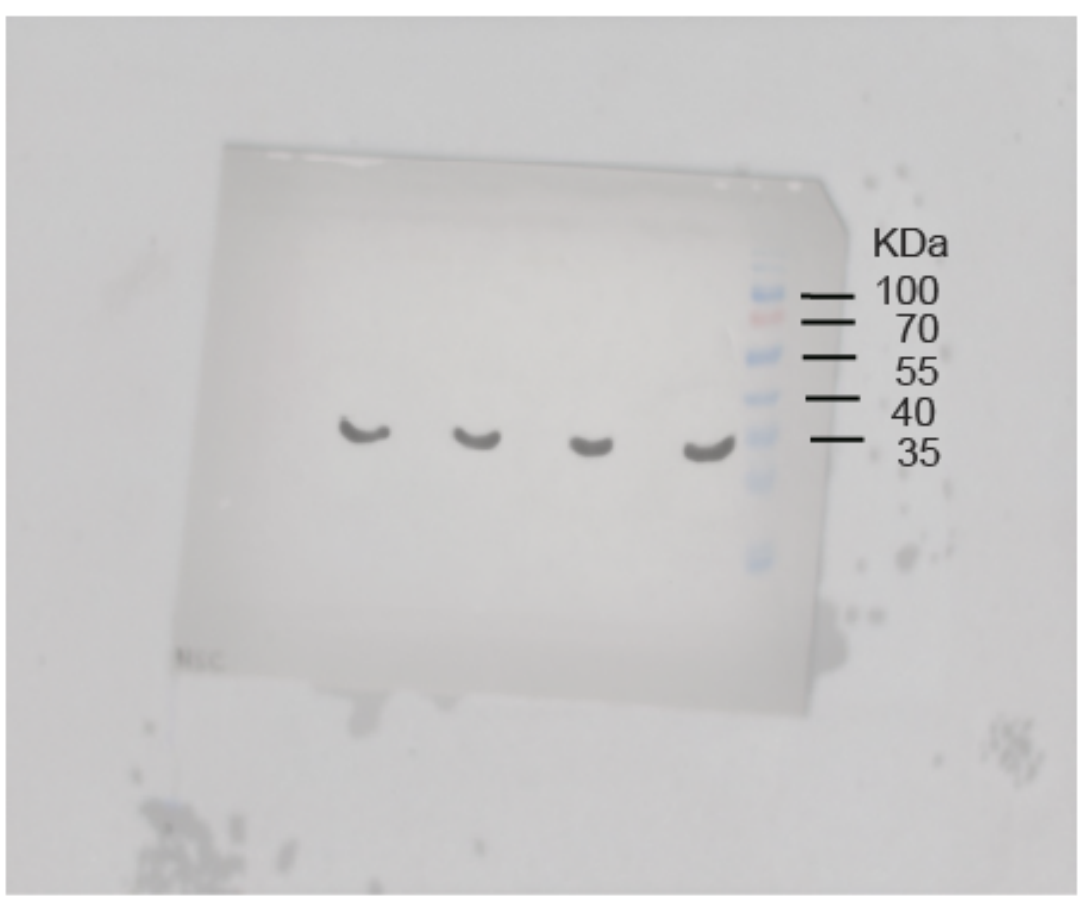

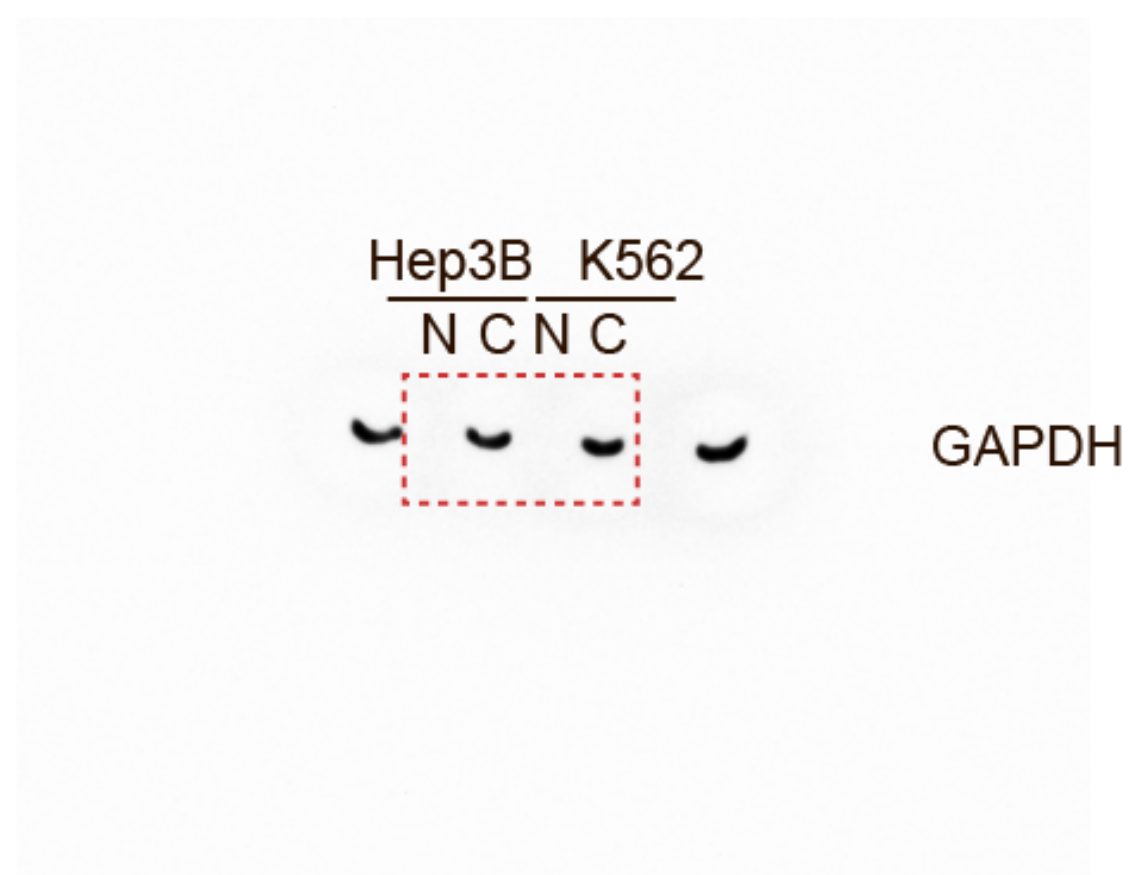

Uncropped blot from Supplementary Fig. 12f

Dotted red region is the image used in manuscript

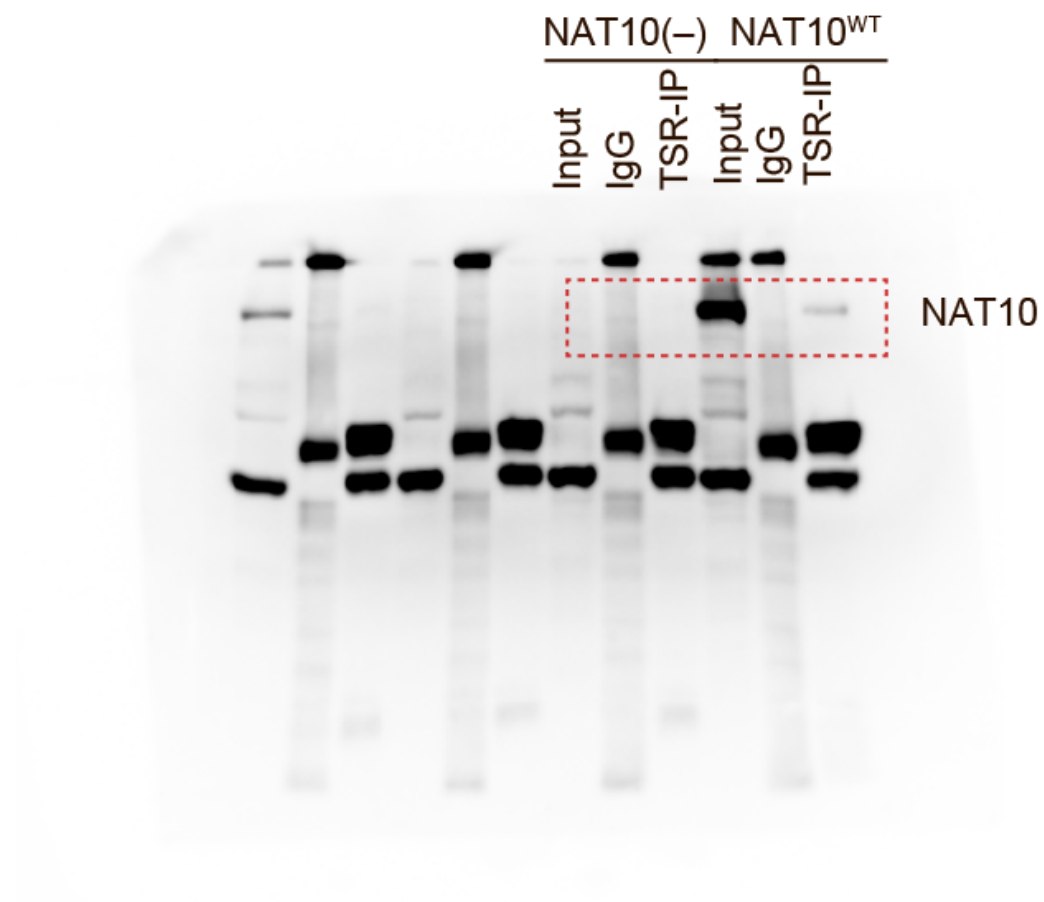

Overlay with ladder

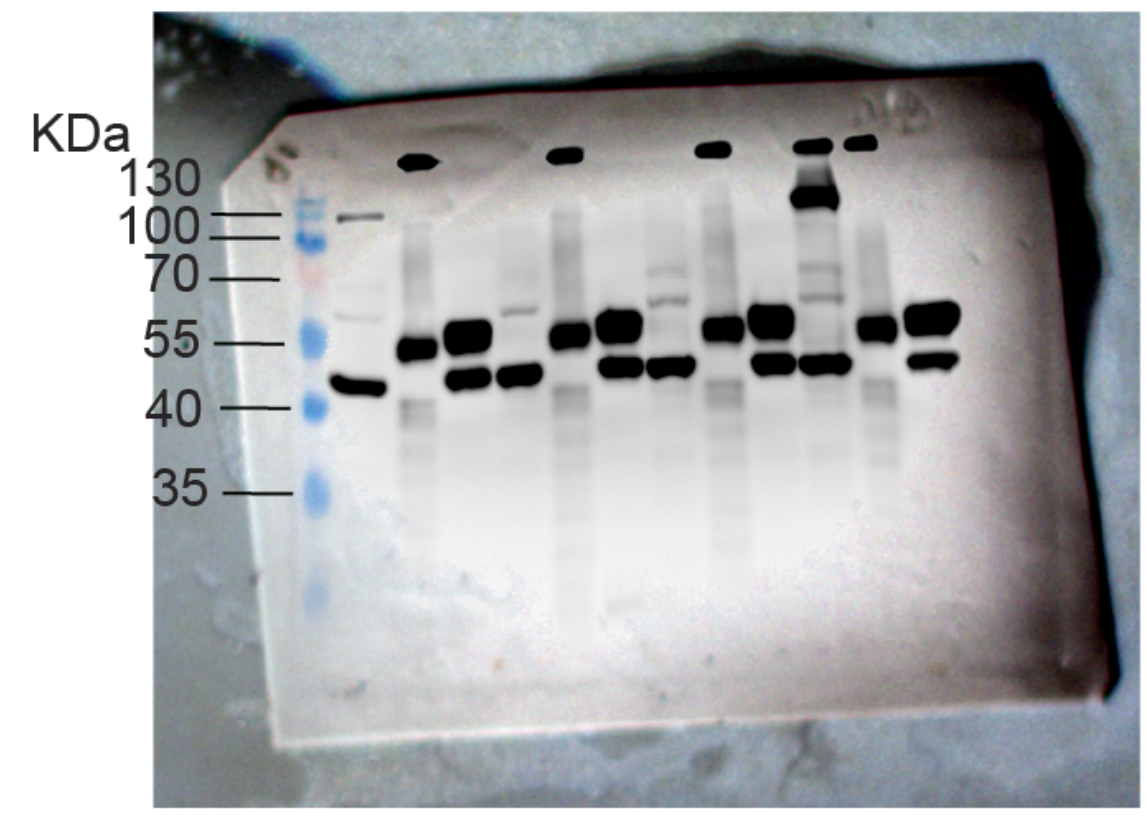

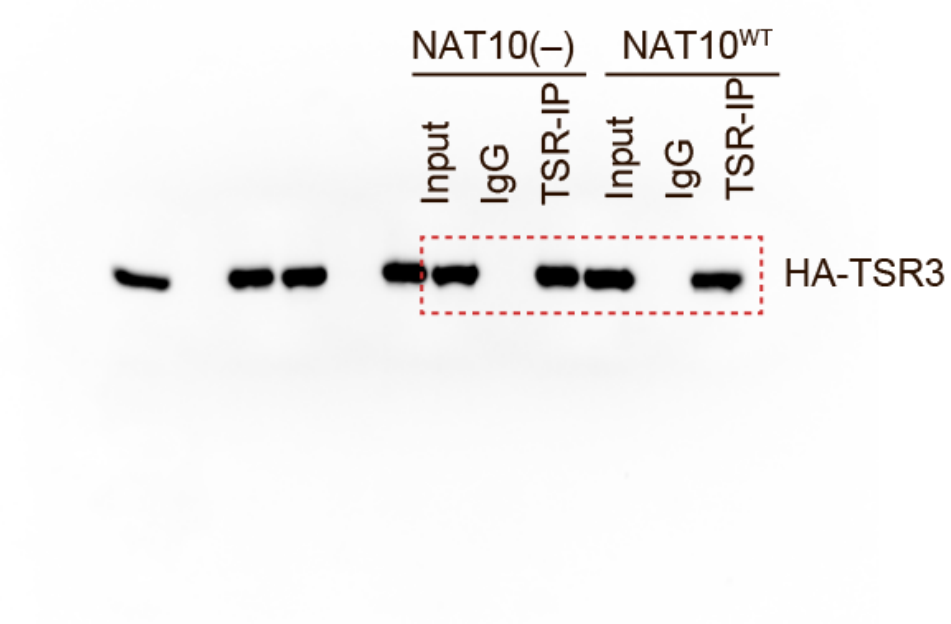

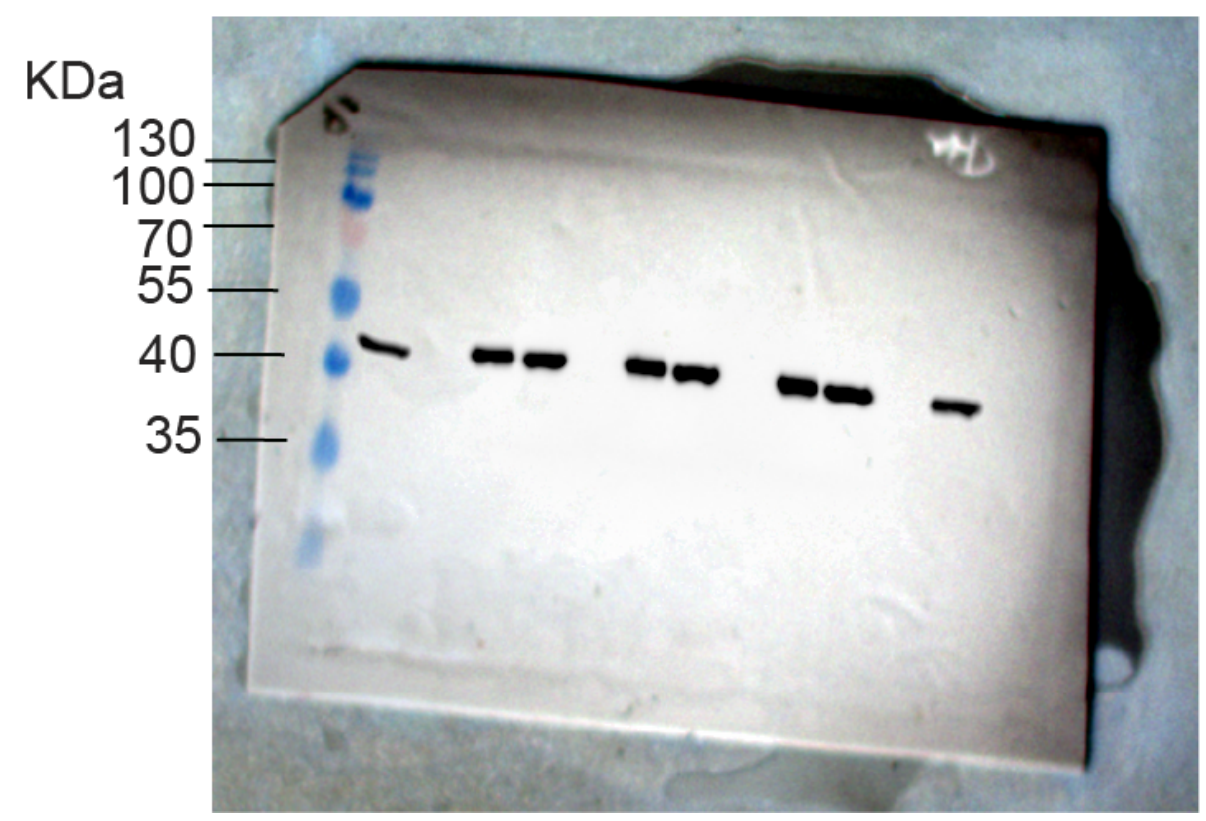

Uncropped gel from Supplementary Fig. 12i

Dotted red region is the image used in manuscript

NAT10^{WT}

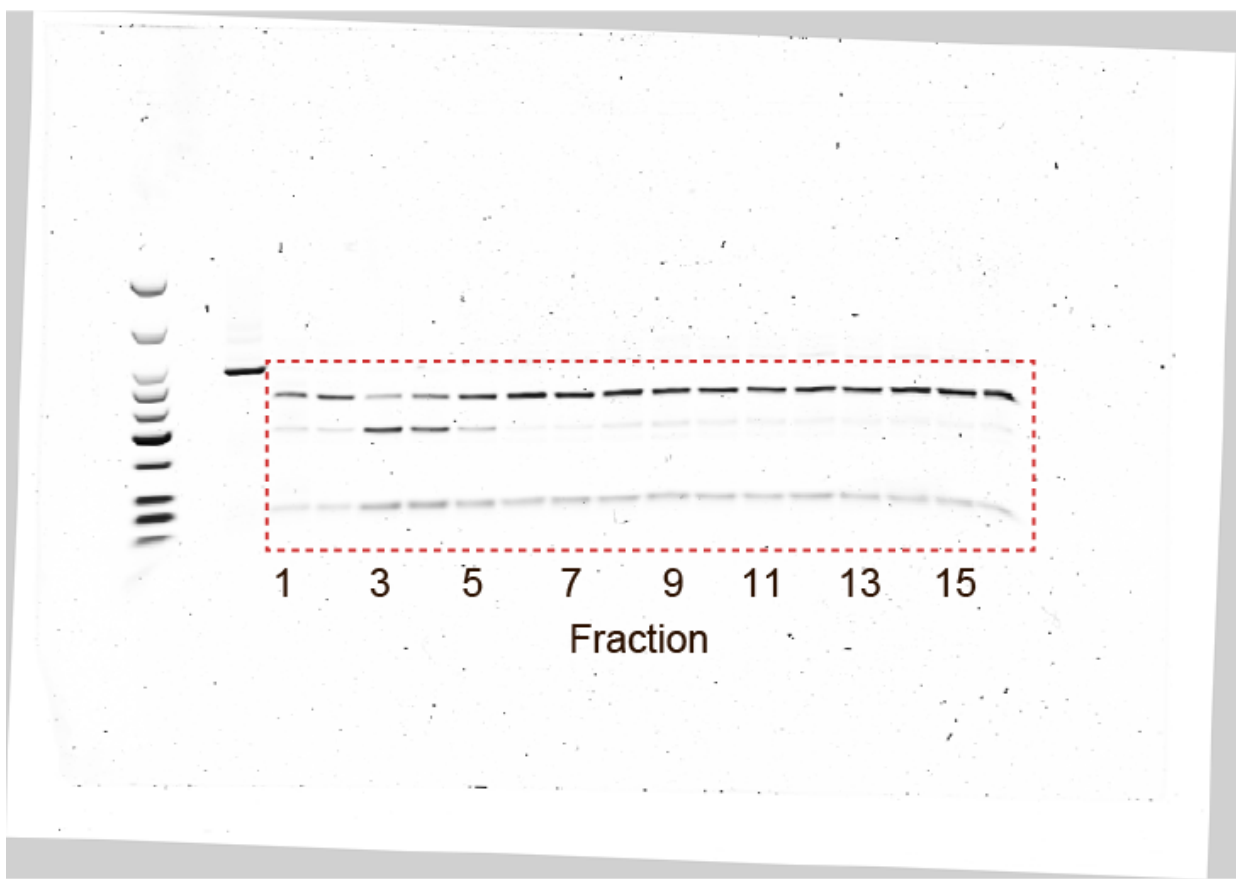

NAT10(–)

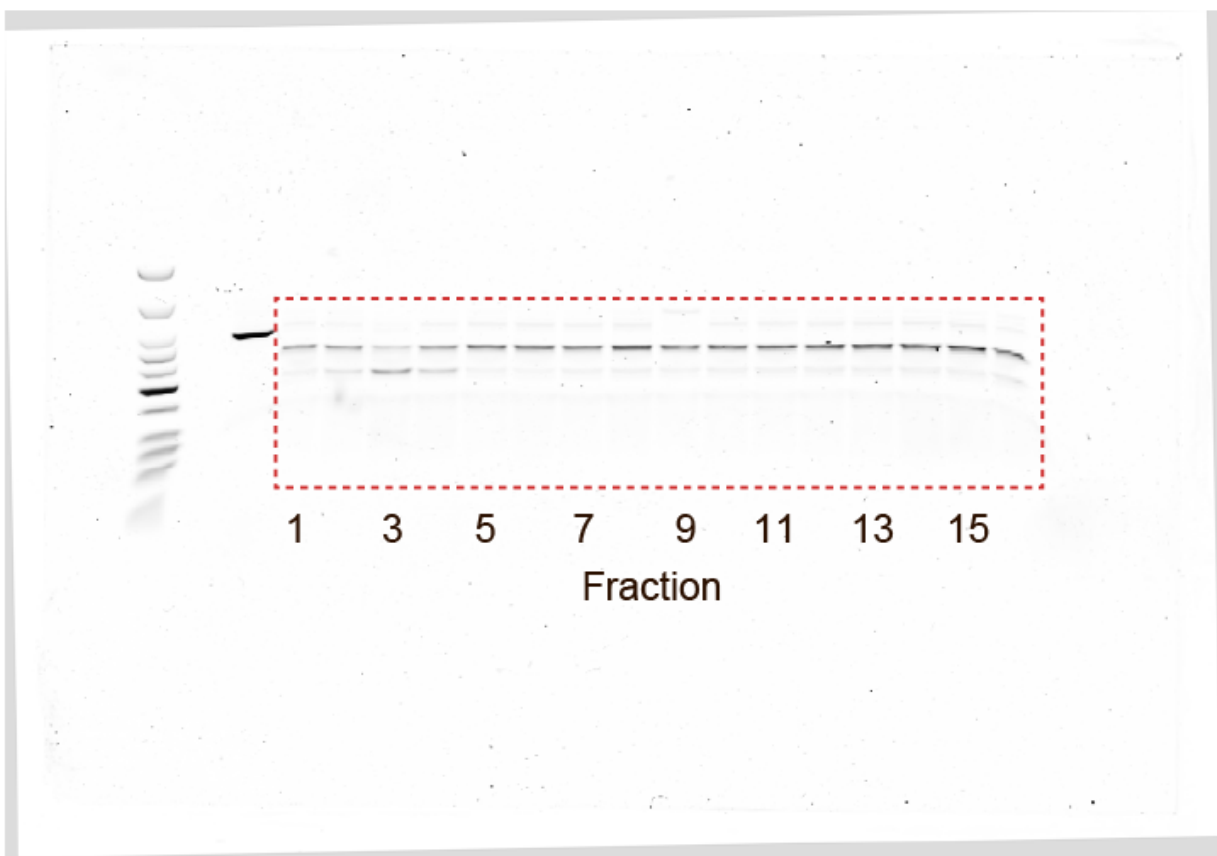

Supplement: Supplementary file 3 [file 41467_2026_76383_MOESM1_ESM.pdf]
