# Supplementary material for: The RNA helicase domain of NAT10 promotes the biogenesis of hypomodified ribosomes to enhance cancer cell proliferation
Source: Nat Commun. 2026 Aug 8;17:9529. doi: 10.1038/s41467-026-76383-w (PMC13547379; doi:10.1038/s41467-026-76383-w)
Supplement: Supplementary file 4 [file 41467_2026_76383_MOESM2_ESM.pdf]

Supplementary Movie 1. AlphaFold3 model showing the DUF1726 domain (gray) and RNA helicase domain (black) of NAT10 in complex with ATP (blue). Red spheres highlight residues essential for proliferation.

Supplementary Data 1. Description of NAT10 residues selected for site saturation mutagenesis.

Supplementary Data 2. List of barcodes associated with each mutated residue.

Supplementary Data 3. Complete list of analyzed variants from the deep mutational scanning assay. Data is shown as individual variants.

Supplementary Data 4. Complete list of analyzed residues from the deep mutational scanning assay. Data is shown as individual residues.

Supplementary Data 5. The list of ac^4C loci significantly decreased in each mutant compared to NAT10-WT.

Supplementary Data 6. Direct RNA Nanopore sequencing. List of modified 18S rRNA modifications analyzed.

Supplementary Data 7. List of oligonucleotides and plasmids used in this study.
