# Supplementary material for: The RNA helicase domain of NAT10 promotes the biogenesis of hypomodified ribosomes to enhance cancer cell proliferation
Source: Nat Commun. 2026 Aug 8;17:9529. doi: 10.1038/s41467-026-76383-w (PMC13547379; doi:10.1038/s41467-026-76383-w)
Supplement: Supplementary file 5 [file 41467_2026_76383_MOESM4_ESM.docx]

**Supplementary Data 1: Description of mutated residues.**

```
Yellow: Highly conserved
Red – Translation initiation site
Green – Modified or mutated in cancers
Magenta – No conservation

sp|Q9UZ78|TMCA_PYRAB                MTIKIRFPKDVREYARKEKVKESIIKLTETSLAEAITNFHRRMIILQGDTLEKAK--LAG	58
tr|Q5JHC6|Q5JHC6_THEKO              MTVKVRFDKEVREYAKGEKVKDSVLKITETALAQALEKFHRRMIVIEGDTLKKAE--LAG	58
tr|B7R4L8|B7R4L8_9EURY              MTVKVRFDKEVRDYAKGEKVKDSILKLTETALAQALENFHRRMIVIEGDTLRKAE--LAG	58
tr|A0A804QQK4|A0A804QQK4_MAIZE      ---------------MRKKVDERI----RTMIENGVRQRQRSMFVIVGDKSRDQIVNLNY	41
sp|Q9M2Q4|NT102_ARATH               ---------------MRKKVDERI----RTLIENGVKLRHRSMFVIIGDKSRDQIVNLHH	41
sp|Q9XIK4|NT101_ARATH               ---------------MRKKVDERI----RTLIENGVKLRHRSMFVIIGDKARDQIVNLHH	41
sp|Q55EJ3|NAT10_DICDI               --------------MVRKKVDTRI----RTLIENGVATNHRSFFVIVGDNGKDQVPNLHY	42
sp|Q9W3C1|NAT10_DROME               --------------MVKKKIDNRI----RVMIENGVKLGHRTMFIVIGDKARDQVPILYD	42
sp|O01757|NAT10_CAEEL               ---------------MRTKLDGRI----RTQIENGVASGHRSMFAVVGDKARDQVPILYH	41
tr|Q6PFQ5|Q6PFQ5_DANRE              ------------MATFRKKVDNRI----RVQIENGVAEHHRSMFVIVGDRGRDQVVILHH	44
tr|A0A8J0SC30|A0A8J0SC30_XENTR      --------------MQRKKIDNRI----RVLIENGVAQKHRSFFVVVGDQGKDQVVILHH	42
sp|Q9H0A0|NAT10_HUMAN               --------------MHRKKVDNRI----RILIENGVAERQRSLFVVVGDRGKDQVVILHH	42
sp|Q8K224|NAT10_MOUSE               --------------MNRKKVDNRI----RILIENGVAERQRSLFVVVGDRGKDQVVILHH	42
sp|P87115|NAT10_SCHPO               --------------MPKKALDSRI----PTLIKNGCQEKQRSFFVVVGDRARDQVVNLHW	42
sp|P53914|NAT10_YEAST               --------------MAKKAIDSRI----PSLIRNGVQTKQRSIFVIVGDRARNQLPNLHY	42
tr|G8BFD3|G8BFD3_CANPC              --------------MGKKAIDARI----PALIRNGVQEKQRSFFVIVGDKAKNQLPNLHY	42
                                                       :.  :       : :.    :* :: : **  ..    *

sp|Q9UZ78|TMCA_PYRAB                ILAGGVARILSEYIPEFLDRKLRD--EDKIEVLYATDALGEDTYGRKRFEEFRKH-----	111
tr|Q5JHC6|Q5JHC6_THEKO              ILAGASARVLSDIVGELIEKRLRDESEDKIEVLYATDALGEETFGRKRYEAFRKH-----	113
tr|B7R4L8|B7R4L8_9EURY              ILAGASARVLSGILEELMKKRLRDESEDKIEVLYATDALGEETFGRKRYEAFRKH-----	113
tr|A0A804QQK4|A0A804QQK4_MAIZE      MLTKSRVKS-----------------RPSVLWCYRDK-LEISSHKKKRGKQIKKLMQRGL	83
sp|Q9M2Q4|NT102_ARATH               MLSKAVIKC-----------------NPSVLWCYKDK-LDISSHKQKRSKQLKRLRERGQ	83
sp|Q9XIK4|NT101_ARATH               ILSKSVVKS-----------------NPSVLWCYKNR-LDISSHNKKRAKQLKKMKERGQ	83
sp|Q55EJ3|NAT10_DICDI               ILSKSIVKA-----------------RPSVLWCYKED-LGFSKHKKKKMKLKEKSKSSGI	84
sp|Q9W3C1|NAT10_DROME               ILTKSTVKA-----------------RPTVLWCYKNKDEAISNHGKKRAK----KIAVGK	81
sp|O01757|NAT10_CAEEL               ILSKSTVSA-----------------RPNVLWCYKKE-LSFSTHRAKKAKKMKKATTTIS	83
tr|Q6PFQ5|Q6PFQ5_DANRE              MLSKATVRA-----------------RPSVLWCYKKE-LGFSSNRKKRMRQLQKKIKTGT	86
tr|A0A8J0SC30|A0A8J0SC30_XENTR      MLSKATVRA-----------------RPSVLWCYKKE-LGFSSNRKKKMRKLQKRIKSGS	84
sp|Q9H0A0|NAT10_HUMAN               MLSKATVKA-----------------RPSVLWCYKKE-LGFSSHRKKRMRQLQKKIKNGT	84
sp|Q8K224|NAT10_MOUSE               MLSKATVKA-----------------RPSVLWCYKKE-LGFSSHRKKRMRQLQKKIKSGT	84
sp|P87115|NAT10_SCHPO               LLSQSKVAA-----------------RPNVLWMYKKDLLGFTSHRKKRENKIKKEIKRGI	85
sp|P53914|NAT10_YEAST               LMMSADLKM-----------------NKSVLWAYKKKLLGFTSHRKKRENKIKKEIKRGT	85
tr|G8BFD3|G8BFD3_CANPC              LMMSANLKM-----------------NKSVLWAYKKKLLGFTSHRQKREAKIKKDIKRGI	85
                                    ::  .                     . .:   *        .   *:

sp|Q9UZ78|TMCA_PYRAB                --------FSVLAP-NAELTSVTFKYSRDILGRTFDILVLDLSYDYSPNDLGRIIETVRG	162
tr|Q5JHC6|Q5JHC6_THEKO              --------FDVLAGSEVNVTAVTFKHTREILGRTYDLLILDMSYDYSPNDLGRIIETVRG	165
tr|B7R4L8|B7R4L8_9EURY              --------FDVLAGSNVEVKAVTFKHTRDILGRTYDLLILDMSYDYSPNDLGRIIETVRG	165
tr|A0A804QQK4|A0A804QQK4_MAIZE      LDPEKADPFSLF-METSDITYCLYRDSERVLGNTFGMCILQDFEALTPNLLARTIETVEG	142
sp|Q9M2Q4|NT102_ARATH               LDPEKLDAFSRL-LDVGRVTHCLYKDSERILGNTFGMCILQDFEALTPNLLARTIETVEG	142
sp|Q9XIK4|NT101_ARATH               LDPEKLDAFSLF-LDVVDVTHCLYKDSERILGNTFGICILQDFEALTPNLLARTIETVEG	142
sp|Q55EJ3|NAT10_DICDI               DSVNQEDPFDVF-ISTTNIRYSYYSESHKILGNTFGMLVLQDFEAITPNLLARTIETVEG	143
sp|Q9W3C1|NAT10_DROME               VDVNEADLFDSF-RVATTIHGRYYSETHAVLGRTYGVCVLQDFEALTPNLLARTVETVEG	140
sp|O01757|NAT10_CAEEL               GSLPDADPFDVF-ISSTQIRYCYYNETEKILGNTFGVLVLQDFEAMTPNLLARTIETIEG	142
tr|Q6PFQ5|Q6PFQ5_DANRE              LNLNQDDPFELF-VAATNIRYCYYNETHKILGNTFGMCVLQDFEALTPNLLARTVETVEG	145
tr|A0A8J0SC30|A0A8J0SC30_XENTR      LNIKEDDPFELF-VAATNIRYCYYNETHKILGNTYGMCVLQDFEALTPNLLARTVETVEG	143
sp|Q9H0A0|NAT10_HUMAN               LNIKQDDPFELF-IAATNIRYCYYNETHKILGNTFGMCVLQDFEALTPNLLARTVETVEG	143
sp|Q8K224|NAT10_MOUSE               LNLKQDDPFELF-VAATNIRYCYYNETHKILGNTFGMCVLQDFEALTPNLLARTVETVEG	143
sp|P87115|NAT10_SCHPO               RDPNSEDPFELF-CSITNIRYCYYKESEKILGQTYGMLVLQDFEALTPNLLARTIETVEG	144
sp|P53914|NAT10_YEAST               REVNEMDPFESF-ISNQNIRYVYYKESEKILGNTYGMCILQDFEALTPNLLARTIETVEG	144
tr|G8BFD3|G8BFD3_CANPC              REVNDQDPFEAF-ISNQHIRYVYYKETDKILGNTYGMCILQDFEAMTPNLLARTIETVEG	144
                                            *. :      :    :  :  :**.*:.: :*:     :** *.* :**:.*

sp|Q9UZ78|TMCA_PYRAB                GGLIFILTNPFEKWKDMWTGF---HKSLVTPPYTIDDVKKRFNRRLIRKFTEHKGIYIVD	219
tr|Q5JHC6|Q5JHC6_THEKO              GGLIFILAHPFEKWKDMWTGF---HKSLVTPPYTIEDVKKRFNRRLIRKFTQHDGIYIIT	222
tr|B7R4L8|B7R4L8_9EURY              GGLIFILAHPFEKWKNMWTGF---HKSLVTPPYTIDDVKKRFNRRLIRKFTEHDGIYIIT	222
tr|A0A804QQK4|A0A804QQK4_MAIZE      GGLIILLLRSLSSLTSLYTMVMDVHERFRTE-S-HTQAAARFNERFLLSIASCKACVVMD	200
sp|Q9M2Q4|NT102_ARATH               GGLVVLILRSLTSLTSLCTMVMDVHDRFRTE-S-HSEAAGRFNERFLLSLASCKACVVMD	200
sp|Q9XIK4|NT101_ARATH               GGLVVLLLQSLASLTSLCTMVMDVHDRFRTE-S-HSEASGRFNERFLLSLASCKACVVMD	200
sp|Q55EJ3|NAT10_DICDI               GGLIVLLLKTMDSLKQLYTMTMDVHTRFRSE-NSKGEVVCRFNERFLLSLGKSEQCLVMD	202
sp|Q9W3C1|NAT10_DROME               GGLIILLLKTLQSLKQLYTMSMDVHKRFRTE-A-HQTVTCRFNERLILSLADCKRCLVVN	198
sp|O01757|NAT10_CAEEL               GGMVILLMQSVRSLRQLYTISMDVHNRYRTE-A-HNEITARFNERFILSLASCSSVLVLD	200
tr|Q6PFQ5|Q6PFQ5_DANRE              GGIVVILLRTMNSLKQLYTMTMDVHSRYRTE-A-HQDVIGRFNERFILSLSSCKSCVVID	203
tr|A0A8J0SC30|A0A8J0SC30_XENTR      GGIVVLLLRTMSSLKQLYTMTMDVHSRYRTE-A-HQDVVVRFNERFILSLASCKTCMVID	201
sp|Q9H0A0|NAT10_HUMAN               GGLVVILLRTMNSLKQLYTVTMDVHSRYRTE-A-HQDVVGRFNERFILSLASCKKCLVID	201
sp|Q8K224|NAT10_MOUSE               GGLVVILLRTMNSLKQLYTMTMDVHSRYRTE-A-HQDVVGRFNERFILSLASCKKCLVID	201
sp|P87115|NAT10_SCHPO               GGIVVLLLHKLNSLKQLYTMSMDIHSRYRTE-A-HSDVTARFNERFILSLGNCENCLVID	202
sp|P53914|NAT10_YEAST               GGIVVILLKSMSSLKQLYTMTMDVHARYRTE-A-HGDVVARFNERFILSLGSNPNCLVVD	202
tr|G8BFD3|G8BFD3_CANPC              GGLVVILLKNMTSLKQLYTMSMDIHSRYRTE-A-HDDVVARFNERFLLSLGSCENCLVVD	202
                                    **::.:: . . .  .: *     *    :          ***.*:: .: .     ::

sp|Q9UZ78|TMCA_PYRAB                ADKK---------KIERRPRKNKSQ----AKLPEREKVEIPRDIKFPRELYELCLTRGQV	266
tr|Q5JHC6|Q5JHC6_THEKO              ESG----------KAKKKPKKSKSQ----AKIRARKGVEIPEETLFPKELYEMALTRGQV	268
tr|B7R4L8|B7R4L8_9EURY              EGG----------KARKKPKRSKSQ----ARIKARKGVPIPEETLFPKELYEMALTEGQV	268
tr|A0A804QQK4|A0A804QQK4_MAIZE      DELNILPISSHMKFIQPVTNNEDSEGLSERERELKELKDQFREDFPVGPLIGKCCTMDQG	260
sp|Q9M2Q4|NT102_ARATH               DELNILPLSSHIRSITQVPTEKDSEGLSEAERDLKSLKEDLSDDFPVGPLIKKCCTLDQG	260
sp|Q9XIK4|NT101_ARATH               DELNLLPLSSHIKSITKVPTKEDSEALSEAERDLKSLKDALNDDFPVGPLINKCCTLDQG	260
sp|Q55EJ3|NAT10_DICDI               DELNILPISSQSRSIEAKQ----QILETPEQVELREFKQQVKDTDIAGALIENTKTMDQA	258
sp|Q9W3C1|NAT10_DROME               DDLTVLPLSSKTINVEPVNPAG--AGRSPNEASLKELKESLLTVQPAGALVNLCKTYDQA	256
sp|O01757|NAT10_CAEEL               DQLRVLPISSHIENVEAIPASQ-KKIQSESDAELASLKEAMKETKPIGPLLSRARTACQA	259
tr|Q6PFQ5|Q6PFQ5_DANRE              DQLNILPISSHATNIKPIPPKKQDDGLSPREQELKDLKESLQDTQPVGVLVDGCKTLDQA	263
tr|A0A8J0SC30|A0A8J0SC30_XENTR      DQLNILPISSHIANIKPIPPKAQDASLSPEEQELQDVKLSLQDTQPVGVLVENCKTLDQA	261
sp|Q9H0A0|NAT10_HUMAN               DQLNILPISSHVATMEALPPQTPDESLGPSDLELRELKESLQDTQPVGVLVDCCKTLDQA	261
sp|Q8K224|NAT10_MOUSE               DQLDILPISSHVASIEALPPQAPDENLSPAALELLELKESLQDTQPVGVLVDCCKTLDQA	261
sp|P87115|NAT10_SCHPO               DELNVLPISGG-KNVKALPPTL-EEDNS-TQNSIKELQESLGEDHPAGALVGVTKTLDQA	259
sp|P53914|NAT10_YEAST               DELNVLPLSGA-KNVKPLPPKE-DDELPPKQLELQELKESLEDVQPAGSLVSLSKTVNQA	260
tr|G8BFD3|G8BFD3_CANPC              DELNVLPISGG-KHVKPLPPRD-DDDLSPRQVELKELKDSLADVQPAGSLVNLAKTINQA	260
                                                                       .             *     *  *

sp|Q9UZ78|TMCA_PYRAB                EVLKALEDLIE-N--PGMVVLTADRGRGKSVSVGIASIGLAITSKKKNFRIVVTAPELEN	323
tr|Q5JHC6|Q5JHC6_THEKO              EVLKAFEELVD-K--EGMLVLTADRGRGKSVSVGIAAIGLAVALK-KRTRIVVTAPELEN	324
tr|B7R4L8|B7R4L8_9EURY              EVLKAFEELVE----GGMLVLTADRGRGKSVSVGIGAIGLALALK-KRTRIVVTAPELEN	323
tr|A0A804QQK4|A0A804QQK4_MAIZE      KAVINFLDSILDKSLRSTVALLAARGRGKSAALGLAIAGA---VAAGYSNIFVTAPSPEN	317
sp|Q9M2Q4|NT102_ARATH               KAVVTFFDAILDKALRSIVALIASRGRGKSAALGLAVAGA---VAAGYSNIYITAPSPDN	317
sp|Q9XIK4|NT101_ARATH               KAVVTFFDAILDKTLRSIVALIASRGRGKSAALGLAVAGA---VAAGYSNIYVTAPSPDN	317
sp|Q55EJ3|NAT10_DICDI               TALLTFIDSISEKTLRSTVTLTAGRGRGKSAALGLAISAA---VAFGYSNIFVSSPTPEN	315
sp|Q9W3C1|NAT10_DROME               NAVAQFIEALVDKQLKPPMSLTAARGRGKSAALGLSIAAA---VAFGYVNIYVTSPHPEN	313
sp|O01757|NAT10_CAEEL               KALLRFLDVITEKQSNVTCSLTAGRGRGKSAAVGLSLAGA---IAFGYTNIFVTSPSPEN	316
tr|Q6PFQ5|Q6PFQ5_DANRE              KAVLKFIEAISEKTLRSTVALTAARGRGKSAALGLAMAGA---VAFGYSNIFVTSPSPDN	320
tr|A0A8J0SC30|A0A8J0SC30_XENTR      KAVLKFIEAVSEKTLRSTVTLTAARGRGKSAALGLAIAGA---VAFGYSNIFVTSPSPDN	318
sp|Q9H0A0|NAT10_HUMAN               KAVLKFIEGISEKTLRSTVALTAARGRGKSAALGLAIAGA---VAFGYSNIFVTSPSPDN	318
sp|Q8K224|NAT10_MOUSE               KAVLKFIEGISEKTLRSTVALTAARGRGKSAALGLAIAGA---VAFGYSNIFVTSPSPDN	318
sp|P87115|NAT10_SCHPO               RAVLTFVESIVEKSLKGTVSLTAGRGRGKSAALGLAIAAA---IAHGYSNIFITSPSPEN	316
sp|P53914|NAT10_YEAST               HAILSFIDAISEKTLNFTVALTAGRGRGKSAALGISIAAA---VSHGYSNIFVTSPSPEN	317
tr|G8BFD3|G8BFD3_CANPC              QAILTFIDVIVEKTLRSTVTLTAGRGRGKSAALGIAIAAA---VSQGYSNIFVTSPSPEN	317
                                     .:  : : :          * * ******.::*:.  .          .* :::*  :*

sp|Q9UZ78|TMCA_PYRAB                VQSLLKFAERSLKVLGYKTKTVKES----------GL--IKEVYAKGIGIRYYPPTKG-Y	370
tr|Q5JHC6|Q5JHC6_THEKO              VQSLFRFAKRALEKLGFKPYVVEEK----------GL--IKELYARKIGLRYYPPAEG-Y	371
tr|B7R4L8|B7R4L8_9EURY              VQALFRFAKRALERLGFKPYVVEER----------GL--IKELYARKIGLRYYPPADG-Y	370
tr|A0A804QQK4|A0A804QQK4_MAIZE      LKTLFDFVCKGINALEYKEHLHYDVMKSADPELKKATIQINVYKQHRQTIQYLKPNDHGK	377
sp|Q9M2Q4|NT102_ARATH               LKTFFEFVCKGFDALEYKEHLDYDVVKSANPDFKKAVVRINIFKQHRQTIQYIQPHEHEK	377
sp|Q9XIK4|NT101_ARATH               LKTVFEFVCKGFDALEYKEHLEYDVVRSVNPEFNKAIVRINIFKQHRQTIQYIQPHEHEK	377
sp|Q55EJ3|NAT10_DICDI               LNTLFQFVFKGFDSMEYVEHVDYELVKSTNPEFHDAIIRVNIFRSHRQTIQYIQPQDYQK	375
sp|Q9W3C1|NAT10_DROME               LITLFEFVLKGFDALEYQEHADYTIIRSTNADYKKAIIRINITRSSRQTIQYIAPSDTHL	373
sp|O01757|NAT10_CAEEL               LKTLFEFVVKGFDALDYQEHTDYELIQSANPEFKNCLVRINVFREHKQTIQYISPTDVQK	376
tr|Q6PFQ5|Q6PFQ5_DANRE              LHTLFEFVFKGFDALQYQEHLDYEIIQSLNPEFNKAVVRVNIFKEHRQTIQYIHPADAVK	380
tr|A0A8J0SC30|A0A8J0SC30_XENTR      LHTLFEFIFKGFDSLQYQEHMDYEIIQSLNPEFEKAVVRVNVFKEHRQTIQYIHPADAVK	378
sp|Q9H0A0|NAT10_HUMAN               LHTLFEFVFKGFDALQYQEHLDYEIIQSLNPEFNKAVIRVNVFREHRQTIQYIHPADAVK	378
sp|Q8K224|NAT10_MOUSE               LHTLFEFVFKGFDALQYQEHLDYEIVQSLNPEFNKAVIRVNVFREHRQTIQYIHPADAVK	378
sp|P87115|NAT10_SCHPO               LKTLFEFIFKGFDALNYEEHVDYDIIQSTNPAYHNAIVRVNIFRDHRQTIQYISPEDSNV	376
sp|P53914|NAT10_YEAST               LKTLFEFIFKGFDALGYQEHIDYDIIQSTNPDFNKAIVRVDIKRDHRQTIQYIVPQDHQV	377
tr|G8BFD3|G8BFD3_CANPC              LKTLFEFVFKGFDALGYTEHLDYDIIQSTNPSFNKAIVRVDIKREHRQTIQYISPTDNHV	377
                                    : :.: *  :.:. : :                      :.        ::*  * .

sp|Q9UZ78|TMCA_PYRAB                RQKADLYIVDEAAGIHVPILHRYLEKER-VVFSSTIHGYEGAGRGFSVKFLKKAKE----	425
tr|Q5JHC6|Q5JHC6_THEKO              KKNADLYILDEAAGIHVPILHKYLNKPR-VVYSSTIHGYEGAGRGFSVKFLKKARE----	426
tr|B7R4L8|B7R4L8_9EURY              KKSADLYILDEAAGIHVPILHKYLNKER-VVYSSTIHGYEGAGRGFSVKFLKRARE----	425
tr|A0A804QQK4|A0A804QQK4_MAIZE      LSQVELLVIDEAAAIPLPIVKSML-GPYLVFLSSTVNGYEGTGRSLSLKLLQQLESQSQP	436
sp|Q9M2Q4|NT102_ARATH               LSQVELLVIDEAAAIPLPVVKSLL-GPYLVFLSSTVSGYEGTGRSLSLKLLQQLDEQSRA	436
sp|Q9XIK4|NT101_ARATH               LSQVELLVIDEAAAIPLPVVKSLL-GPYLVFLSSTVSGYEGTGRSLSLKLLQQLEEQSRA	436
sp|Q55EJ3|NAT10_DICDI               LGQAELVVIDEAAAIPLPLVKNLL-GPYLVFMSSTINGYEGTGRSLSLKLIKQLREQSTV	434
sp|Q9W3C1|NAT10_DROME               LNAADLLLIDEAAAIPLPLVKKMI-GPYLVFMASTINGYEGTGRSLSLKLISQLQKDNNA	432
sp|O01757|NAT10_CAEEL               LGQCELIVIDEAAAIPLPLVKELISGPYISFLSSTINGYEGTGRSLSLKLLQQLRQQSAG	436
tr|Q6PFQ5|Q6PFQ5_DANRE              LGQAELLVIDEAAAIPLPLVKKLL-GPYLVFMASTINGYEGTGRSLSLKLIQQLRQQSSE	439
tr|A0A8J0SC30|A0A8J0SC30_XENTR      LGQAELVVIDEAAAIPLPLVKNLM-GPYLVFMASTINGYEGTGRSLSLKLIQQLRQQSAD	437
sp|Q9H0A0|NAT10_HUMAN               LGQAELVVIDEAAAIPLPLVKSLL-GPYLVFMASTINGYEGTGRSLSLKLIQQLRQQSAQ	437
sp|Q8K224|NAT10_MOUSE               LGQAELVVIDEAAAIPLPLVKSLL-GPYLVFMASTINGYEGTGRSLSLKLIQQLRQQSAQ	437
sp|P87115|NAT10_SCHPO               LGQAELVVIDEAAAIPLPLVRKLI-GPYLVFMASTINGYEGTGRSLSLKLLQQLREQSRI	435
sp|P53914|NAT10_YEAST               LGQAELVVIDEAAAIPLPIVKNLL-GPYLVFMASTINGYEGTGRSLSLKLIQQLRNQNNT	436
tr|G8BFD3|G8BFD3_CANPC              LGQAELLIIDEAAAIPLPVVKKLM-GPYLVFMASTINGYEGTGRSLSLKLIQQLRNEQQM	436
                                        :* ::****.* :*:::  :      . :**: ****:**.:*:*::.:  .

sp|Q9UZ78|TMCA_PYRAB                -------------------------KREYKEIHLSVPIRYAEGDPIERWLFDVLLLDAEP	460
tr|Q5JHC6|Q5JHC6_THEKO              -------------------------KRSFKELHMEEPIRYAYGDPIEKWLFDVLLLDAEP	461
tr|B7R4L8|B7R4L8_9EURY              -------------------------KREFKELHMDEPIRYAENDPIERWLFDVLLLDAEP	460
tr|A0A804QQK4|A0A804QQK4_MAIZE      S-V---------------QSNGPNSSRLFKKIELNESIRYASGDPVETWLNDLLCLDLAN	480
sp|Q9M2Q4|NT102_ARATH               PAT---------------GLEG--SGCLFKKIELTESIRYGSGDPIESWLNGLLCLDVAT	479
sp|Q9XIK4|NT101_ARATH               PVT---------------GVEGSLSGCLFKKIELSESIRYASGDPIESWLNGLLCLDVAN	481
sp|Q55EJ3|NAT10_DICDI               VSNS------------SNKALNSITGRMLREIELNEPIRYSARDPIERWLNELLCLDSTI	482
sp|Q9W3C1|NAT10_DROME               R----------------------------PPLKLEESIRYQENDDIEKWLINLLCLDAST	464
sp|O01757|NAT10_CAEEL               GEAKE-------------GKSASNKGKTLHEMHMEESIRYKPGDKIEKWLNRLLCLDATN	483
tr|Q6PFQ5|Q6PFQ5_DANRE              SQQN------LSAENRNTSTARLAAARSLHEVTLHESIRYGQGDHVERWLNDLLCLDCLS	493
tr|A0A8J0SC30|A0A8J0SC30_XENTR      SQVT------VTAENKSTASNRLASARTLHEVTLQESIRYAPGDPVEKWLNDMLCLDCMN	491
sp|Q9H0A0|NAT10_HUMAN               SQVS------TTAENKTTTTARLASARTLYEVSLQESIRYAPGDAVEKWLNDLLCLDCLN	491
sp|Q8K224|NAT10_MOUSE               SQVS------TTAENKTTTTARLASARTLHEVSLQESIRYAPGDAVEKWLNDLLCLDCLN	491
sp|P87115|NAT10_SCHPO               YSGS---------GNNKSDSQSHISGRTLKEISLDEPIRYAMGDRIELWLNKLLCLDAAS	486
sp|P53914|NAT10_YEAST               SGRESTQTAVVSRDNKEKDSHLHSQSRQLREISLDEPIRYAPGDPIEKWLNKLLCLDVTL	496
tr|G8BFD3|G8BFD3_CANPC              SNAPA-GTEIVSRDKKSTD-VGGSLTRNLKEVVLDEPIRYAPGDPVEKWLNKLLCLDVSL	494
                                                                   : :   ***   * :* **  :* **

sp|Q9UZ78|TMCA_PYRAB                VE---LTEEDYELIRKMEVYLE-EPDLDDWFENDREDLRHFVGIYVLAHYRNRPSDVALL	516
tr|Q5JHC6|Q5JHC6_THEKO              VE---LTEEDYELIKRKEVYLE-EPDLDDWFENDREDLRHFVGIYILAHYRNRPSDVALL	517
tr|B7R4L8|B7R4L8_9EURY              VE---LTEEDFELIEKKEVYLE-EPDLDDWFENDREDLRNFVGIYILAHYRNRPSDVALL	516
tr|A0A804QQK4|A0A804QQK4_MAIZE      S---IPNISRLPHPKECDLYYVNRDTLFSYHKESEIFLQRMMALYVASHYKNSPNDMQLM	537
sp|Q9M2Q4|NT102_ARATH               C---LPNPACHPSPSQCDLYYVNRDTLFSYHKDSELFLQRMMALCVSSHYKNSPNDLQLL	536
sp|Q9XIK4|NT101_ARATH               C---LPNPACHPLPSQCDLYYVNRDTLFSYHKDSELFLQRMMALCVSSHYKNSPNDLQLL	538
sp|Q55EJ3|NAT10_DICDI               A---KSSPTGCPHPSACQLYYVNRDTLFSYHKASEAFLQKMVGLFVSSHYKNSPNDLLLM	539
sp|Q9W3C1|NAT10_DROME               V---PSISSGCPTPDACELYYVDRDALFSYHKAAEAFLHRLVSIYVSSHYKNTPNDLQMM	521
sp|O01757|NAT10_CAEEL               CQ--LKLECGTPPPAACELYIVNRDTLFSFHDASEAFLQQVMAIFVSAHYKNSPNDLQML	541
tr|Q6PFQ5|Q6PFQ5_DANRE              V---PRIISGCPLPQTCDLYYVNRDTLFCYHKASEAFLQRLMALYVASHYKNSPNDLQML	550
tr|A0A8J0SC30|A0A8J0SC30_XENTR      I---SRIISGCPLPETCDLYYINRDTLFCYHKASESFLQRLMALYVASHYKNSPNDLQMM	548
sp|Q9H0A0|NAT10_HUMAN               I---TRIVSGCPLPEACELYYVNRDTLFCYHKASEVFLQRLMALYVASHYKNSPNDLQML	548
sp|Q8K224|NAT10_MOUSE               I---TRIVSGCPLPEACELYYVNRDTLFCYHKASEVFLQRLMALYVASHYKNSPNDLQML	548
sp|P87115|NAT10_SCHPO               -YVSRMATQGFPHPSECSLYRVSRDTLFSYHPISEAFLQRMMSLYVASHYKNSPNDLQLM	545
sp|P53914|NAT10_YEAST               IKNPRFATRGTPHPSQCNLFVVNRDTLFSYHPVSENFLEKMMALYVSSHYKNSPNDLQLM	556
tr|G8BFD3|G8BFD3_CANPC              SKNAKFATKGTPHPSQCNLFYVNRDTLFSYHPVSEAFLQKMMALYVASHYKNSPNDLQLM	554
                                                     .::   .  *  :.   .  *...:.: : :**:* *.*: ::

sp|Q9UZ78|TMCA_PYRAB                ADAPHHEARVLRL--------KNGKIVTAIQIAKEGGIPKAV-IDKMAKGYKPPGNIIPD	567
tr|Q5JHC6|Q5JHC6_THEKO              ADAPHHEARVLRL--------KNGKIVTAVQIAKEGGIPKNV-IDKMAKGYKPRGNIIPD	568
tr|B7R4L8|B7R4L8_9EURY              ADAPHHEARVLRL--------KNGKIVTAIQIAKEGGIPKKV-IEKMAKGYKPRGNIIPD	567
tr|A0A804QQK4|A0A804QQK4_MAIZE      ADAPAHHLFVLLGPVDESK-NQLPDILCVIQVCLEGQISRKSAMKSLSEGRSPSGDQIPW	596
sp|Q9M2Q4|NT102_ARATH               ADAPAHHLFVLLGPVDESQ-NKIPDILCVIQVCLEGKISENSALQSLRDGHSPYGDQIPW	595
sp|Q9XIK4|NT101_ARATH               SDAPAHHLFVLLGPVDESK-NQLPDILCVIQVCLEGQISRKSAEKSLREGHSPHGDQIPW	597
sp|Q55EJ3|NAT10_DICDI               SDAPDHHLFVLLGPIDENNTTGLPEILCAVQVSLEGEIAKESILNSIKRGYQASGDLIPW	599
sp|Q9W3C1|NAT10_DROME               SDAPAHHLFCLLGPVQRMD--ALPEILVVIQVALEGQISAQSISDSLGRGKKAAGDLIPW	579
sp|O01757|NAT10_CAEEL               SDAPAHNLFVLMAPIDKSR-KTIPEVLAVVQVCLEGRLDSDNIQNGLESGKRAAGDLLPW	600
tr|Q6PFQ5|Q6PFQ5_DANRE              SDAPAHHLFCLLPPVPPTQ-NSLPEVLAVLQVCLEGEISRQSILSSLSRGKKASGDLIPW	609
tr|A0A8J0SC30|A0A8J0SC30_XENTR      SDAPAHHLFCLLPPVPPTQ-NSVPEVLAVVQVCLEGEISRQSILSSLSRGKKASGDLIPW	607
sp|Q9H0A0|NAT10_HUMAN               SDAPAHHLFCLLPPVPPTQ-NALPEVLAVIQVCLEGEISRQSILNSLSRGKKASGDLIPW	607
sp|Q8K224|NAT10_MOUSE               SDAPAHHLFCLLPPVPPTQ-NALPEVLAVVQVCLEGEISRQSILNSLSRGKKASGDLIPW	607
sp|P87115|NAT10_SCHPO               SDAPAHQLFVLLPPVDLKN-PKLPDPICVIQLALEGSISRESIMNSLSRGQRAGGDLIPW	604
sp|P53914|NAT10_YEAST               SDAPAHKLFVLLPPIDPKDGGRIPDPLCVIQIALEGEISKESVRNSLSRGQRAGGDLIPW	616
tr|G8BFD3|G8BFD3_CANPC              SDAPAHQLFVLLPPIEEGD-NKIPDPLCVVQLALEGEISKESVRKSLSRGQRAGGDLIPW	613
                                    :*** *.   *             . : .:*:. ** :      . :  *    *: :*

sp|Q9UZ78|TMCA_PYRAB                MMVKHHYAKEFAKLRGYRIVRIATHPDAMDLGLGSKALELLVK-----------------	610
tr|Q5JHC6|Q5JHC6_THEKO              MMVKHHYLKEFARLKGYRIVRIATHPDAMDMGLGSKALELLEK-----------------	611
tr|B7R4L8|B7R4L8_9EURY              MMVKHHYLKEFAKLKGYRIVRIATHPDAMDRGLGSKALELLEK-----------------	610
tr|A0A804QQK4|A0A804QQK4_MAIZE      KFCEQFQDNVFPSLSGARIVRIAVHPSALRLGYGSAAIGLLSRYYEGQMRRLFDDDE-EE	655
sp|Q9M2Q4|NT102_ARATH               KFCEQFRDTEFPGFSGARIVRIAVHPNAMKMGYGSAAVELLTRYFEGQIAPISEAEDKVD	655
sp|Q9XIK4|NT101_ARATH               KFCEQFRDVVFPKLSGARIVRIAVHPNAMKMGYGSAAVELLTRYFEGQLASISEGDDELE	657
sp|Q55EJ3|NAT10_DICDI               TLTQQYQDEDFPRLSGVRIVRIATHPDYQKMGYGSKALELLTQYYQGEITNLDEVNQEEE	659
sp|Q9W3C1|NAT10_DROME               NVAEQYGDRDFPKLCGVRIVRVATHPNYQRMGYGKRAIQLLKDYYARKHTNLEDGPVASK	639
sp|O01757|NAT10_CAEEL               TVSQQFMDKQFGTLCGGRIVRVAVHPDYQSMGYGGRAVQLIEQYYLGLATSLDEEEKAPA	660
tr|Q6PFQ5|Q6PFQ5_DANRE              TVSEQFQDSEFAGLSGGRIVRIAVNPDYQGMGYGTRALQLLQRYYEGQFPLWDEQEQTMT	669
tr|A0A8J0SC30|A0A8J0SC30_XENTR      TVSEQFQDPDFGSLSGGRVVRIAVHPDYQGMGYGSRALRLVQMYYEGQFPCLEENATNKP	667
sp|Q9H0A0|NAT10_HUMAN               TVSEQFQDPDFGGLSGGRVVRIAVHPDYQGMGYGSRALQLLQMYYEGRFPCLEEKVLETP	667
sp|Q8K224|NAT10_MOUSE               TVSEQFQDPDFGGLSGGRVVRIAVHPDYQGMGYGSRALQLLQMYYEGKFPCLEEKVLETP	667
sp|P87115|NAT10_SCHPO               LISQQFQDENFAALGGARIVRIAVSPEHVKMGYGTRAMQLLHEYFEGKFISASEEFKAVK	664
sp|P53914|NAT10_YEAST               LISQQFQDEEFASLSGARIVRIATNPEYASMGYGSRAIELLRDYFEGKFTDMSEDVRPKD	676
tr|G8BFD3|G8BFD3_CANPC              LISQQFQDEEFASLSGARVVRIATNPEYSGMGYGSRAMELLQDYYAGKFTDISESSTLND	673
                                     . ::.    *  : * *:**:*. *.    * *  *: *:

sp|Q9UZ78|TMCA_PYRAB                -----------------------------------------------EAQEKGLDWVGSG	623
tr|Q5JHC6|Q5JHC6_THEKO              -----------------------------------------------EAREKGLDWIGSG	624
tr|B7R4L8|B7R4L8_9EURY              -----------------------------------------------EAREKGLDWIGSG	623
tr|A0A804QQK4|A0A804QQK4_MAIZE      TEEPDV---------KIAEAAEKASLLEE--TIKPRANLPPLLVHLHERRPEKLHYLGVS	704
sp|Q9M2Q4|NT102_ARATH               VEHAPI---------KVTEAAEKVSMLEE--QVKPRTNLPPLLVPLHDRRPEKLHYIGVS	704
sp|Q9XIK4|NT101_ARATH               VEPSPV---------RVTEAAAKVSLLEE--QIKPRANLPPLLVPLRDRRPERLHYIGVS	706
sp|Q55EJ3|NAT10_DICDI               EEDEEKVDQNKGSSKISTVKKDNASLLTE--VIRPKSNIPPLLFKLSERKPEKLHYMGVS	717
sp|Q9W3C1|NAT10_DROME               DAGKGI----------EEVEEEELSLLKE--QIRPRSRIPTLLQRLHERVPEHVDYIGTS	687
sp|O01757|NAT10_CAEEL               PPSKTV--------IKQVKDGHTVELLEE--RIEPRADLPPLLQRLDERKPERLDYLGVS	710
tr|Q6PFQ5|Q6PFQ5_DANRE              SAITSV--------SSEAV-----SLLEE--VVSPRKDLPPLLLKLNERRAERLDYLGVS	714
tr|A0A8J0SC30|A0A8J0SC30_XENTR      QEITSV--------SSEAV-----SLLEE--SLTPRKNLPPLLLRLSERPAEKLDYLGVS	712
sp|Q9H0A0|NAT10_HUMAN               QEIHTV--------SSEAV-----SLLEE--VITPRKDLPPLLLKLNERPAERLDYLGVS	712
sp|Q8K224|NAT10_MOUSE               QEIRTV--------SSEAV-----SLLEE--VITPRKDLPPLLLKLNERPAERLDYLGVS	712
sp|P87115|NAT10_SCHPO               HSLKRI--------GDEEI--ENTALQTEKIHVRDAKTMPPLLLKLSELQPEPLHYVGVS	714
sp|P53914|NAT10_YEAST               YSIKRV--------SDKEL--AKTNLLKDDVKLRDAKTLPPLLLKLSEQPPHYLHYLGVS	726
tr|G8BFD3|G8BFD3_CANPC              NTLTRV--------TDKEL--ANAS-LKDEIKLREASSLPPLLLKLSEKAPYYLDYLGVS	722
                                                                                   :     :.::* .

sp|Q9UZ78|TMCA_PYRAB                FGASEELIRFWVRNGFAVVHLSPTRNPVSGEYTAIVIKPI--SE--------RAKEIVKK	673
tr|Q5JHC6|Q5JHC6_THEKO              FGASEELVRFWVRNGFAVVHLSPARNPVSGEFTAIVLKPI--SK--------KAKELIKK	674
tr|B7R4L8|B7R4L8_9EURY              FGASEELVRFWVRNGFAVVHLSPARNPVSGEFTAIVLKPI--SE--------RAKKLIKK	673
tr|A0A804QQK4|A0A804QQK4_MAIZE      FGLTQELFRFWRKHNFYPFYVGQIPSAVTGEHTCMVLRPL--NSDDIEVSESNKHGFLDP	762
sp|Q9M2Q4|NT102_ARATH               FGLTLDLFRFWRKHNFAPFYVSQIPSAVTGEHTCMLLKPL--KNDELEVNESDELGFFTP	762
sp|Q9XIK4|NT101_ARATH               FGLTLDLFRFWRKHKFAPFYISQIPSAVTGEHTCMLLKPLTLSNDEFEVDESDELGFFAP	766
sp|Q55EJ3|NAT10_DICDI               YGLTQQLYQFWSKSKYLPVYLRLTSNDITGEHTCIMLREL--NNEQN--NTICKDGWLQS	773
sp|Q9W3C1|NAT10_DROME               YGLTTELLKFWKNAGFVPVYLSQKSNELTAEHSCIMLHTP--N----------ATPWLGL	735
sp|O01757|NAT10_CAEEL               FGLTVPLLKFWKRNEFVPVYIRQNSNDITGEHTCIILKGL--EHGGSDSDEEPSATWLPV	768
tr|Q6PFQ5|Q6PFQ5_DANRE              YGLTPQPLKFWKKAGFVPVYLRQTPNDLTGEHSCIMLKDL--NSE----DLTDQNQWLSA	768
tr|A0A8J0SC30|A0A8J0SC30_XENTR      YGLTPKLLKFWKRAGFVPVYLRQTPNDLTGEHSCVMLKTL--HED----EESDQEPWLTA	766
sp|Q9H0A0|NAT10_HUMAN               YGLTPRLLKFWKRAGFVPVYLRQTPNDLTGEHSCIMLKTL--TDE----DEADQGGWLAA	766
sp|Q8K224|NAT10_MOUSE               YGLTPRLLKFWKRAGFVPVYLRQTPNDLTGEHSCIMLKTL--ADE----DEAEQGAWLAA	766
sp|P87115|NAT10_SCHPO               YGLTPSLQKFWKREGYCPLYLRQTANDLTGEHTCVMLRVL--EG--------RDSEWLGA	764
sp|P53914|NAT10_YEAST               YGLTQSLHKFWKNNSFVPVYLRQTANDLTGEHTCVMLNVL--EG--------RESNWLVE	776
tr|G8BFD3|G8BFD3_CANPC              YGFTPQLHKFWKKSGFTPVYLRQTANELTGEHTAVVLNVL--PD--------RQNKWLHE	772
                                    :* :    :** .  :  .::    . ::.*.:.:::.                   .

sp|Q9UZ78|TMCA_PYRAB                ANDEFRLRLTEWLGDTHRDLEPEIARWLFETPFG---------------EAVNYPIHLTK	718
tr|Q5JHC6|Q5JHC6_THEKO              ANDEFRIRFTEWLGDTHRELEPEIARWLFETPFG---------------EAVDYPIHLTE	719
tr|B7R4L8|B7R4L8_9EURY              ANDEFRIRLTEWLGDTHRELEPEIARWLFETPFG---------------EAVDYPVHLTE	718
tr|A0A804QQK4|A0A804QQK4_MAIZE      FYQDFRQRFRRLLGTSFRHLNFKLAMSVLASKIDYSNHEPS--EYDNNSTSKLLEDMLSP	820
sp|Q9M2Q4|NT102_ARATH               FYKDFKIRFSKLLSDKFKKMDYKLAMSVLNPKINFAEVDSS--GSSSGGFLKTLNGILSP	820
sp|Q9XIK4|NT101_ARATH               FYKDFRIRFSKLLSDKFKKMDYKLAMSVLNPKINFPEVDLT--GNSPDGFLKKLDGVLSP	824
sp|Q55EJ3|NAT10_DICDI               FHQDFKKRFINLLGYEFRNFNSSIALNILYEKKVNNTVAVASSTTKNELTQNEMELLFSS	833
sp|Q9W3C1|NAT10_DROME               YYQDFRRRVLKLMGKTFREFETKLCLALLKNKSVD-----TEGSALKVLDKPMLDVYFLP	790
sp|O01757|NAT10_CAEEL               YWREFRRRIVNLLSFDFSSFPAQMALSLLQLKNKHV----EKQMKRLVIERSELAIHLSN	824
tr|Q6PFQ5|Q6PFQ5_DANRE              FWKDFRRRFLSLMSFQFSKFSSTLALNILQNKSAK-------DDSTAVLTSAELAATFTP	821
tr|A0A8J0SC30|A0A8J0SC30_XENTR      FWKDFRKRFLSLLSYQFSTFAPSLALNILQNRNIK-------KESENLISRSELEAGLTP	819
sp|Q9H0A0|NAT10_HUMAN               FWKDFRRRFLALLSYQFSTFSPSLALNIIQNRNMG-------KPAQPALSREELEALFLP	819
sp|Q8K224|NAT10_MOUSE               FWKDFRRRFLALLSYQFSTFSPALSLNIIQNRNVA-------KSALPALGREHLEALFLP	819
sp|P87115|NAT10_SCHPO               FAQNFYRRFLSLLGYQFREFAAITALSVLDACNNGTKYVV---NSTSKLTNEEINNVFES	821
sp|P53914|NAT10_YEAST               FAKDFRKRFLSLLSYDFHKFTAVQALSVIESSKKAQDLSDDEKHDNKELTRTHLDDIFSP	836
tr|G8BFD3|G8BFD3_CANPC              FSKDFHKRFLQLLSYEFKKFQASQALGIIEAAEQG-----EGEHTTKTLTKTQLDELLSP	827
                                       :*  *.   :.  .  :    .  ::                            :

sp|Q9UZ78|TMCA_PYRAB                VQRKRLEMFIKRVLTYDTVVDAVKPLVKLYFLDGWMR-PYLDDRQIALLIHRVLQAHDWK	777
tr|Q5JHC6|Q5JHC6_THEKO              IQKKRLDAFTGKVLTYDTVVDAVKPIVKLYFLDGWMK-PYLDERQIKLLIYRVLQAHSWE	778
tr|B7R4L8|B7R4L8_9EURY              IQKKRLDAFTGKVLTYDTVVDAVKPIVKLYFLDGWMK-PYLDERQIRLLIYRVLQAHSWE	777
tr|A0A804QQK4|A0A804QQK4_MAIZE      HDMKRLEAYSNNLVDYHLILDLVPILAHQYFSERLPV--SLHGAQAAVLFCLGLQDKDIG	878
sp|Q9M2Q4|NT102_ARATH               YDMERLRAYTENLTDFNLVYDICKTLAHQYFEEKLPV--SLSYVQASILLCLGLQETDFS	878
sp|Q9XIK4|NT101_ARATH               YDMERFRAYTANLVDFNLVYDICKTLAHHYFQEKLPV--SLSYVQASVLLCLGLQESDFS	882
sp|Q55EJ3|NAT10_DICDI               YDLKRLESYSNNIVDYHVVIDLLPSLSKLYFTNKLKI-EDISLIQSAILLALGLQHKTVD	892
sp|Q9W3C1|NAT10_DROME               HDLQRLESYARQQSEFRLIIDLLTDIAQLYFQGRIDG-LQLDLVQQGILLALGVQGKTVD	849
sp|O01757|NAT10_CAEEL               TDLRRMSQYGRNMVDSHIITDILPIVAKLNFEQRLPQELKLAVTQSAILLARGLQHKHFE	884
tr|Q6PFQ5|Q6PFQ5_DANRE              YDLKRLEMYSRNMVDYHLIMDMIPVIARMHFLKQFGD-ISLSVAQSALLLGIGLQHKSVD	880
tr|A0A8J0SC30|A0A8J0SC30_XENTR      YDLKRLEMYSRNMVDYHLIMDLIPSLARMYFLQQLGG-LSLSAAQSALLLGIGLQHKSVD	878
sp|Q9H0A0|NAT10_HUMAN               YDLKRLEMYSRNMVDYHLIMDMIPAISRIYFLNQLGD-LALSAAQSALLLGIGLQHKSVD	878
sp|Q8K224|NAT10_MOUSE               YDLKRLEMYSRNMVDYHLIMDLIPAISRLYFLNQLGD-LSLSAAQSALLLGIGLQHKSVD	878
sp|P87115|NAT10_SCHPO               YDLKRLESYSNNLLDYHVIVDLLPKLAHLYFSGKFPDSVKLSPVQQSVLLALGLQYKTID	881
sp|P53914|NAT10_YEAST               FDLKRLDSYSNNLLDYHVIGDMIPMLALLYFGDKMGDSVKLSSVQSAILLAIGLQRKNID	896
tr|G8BFD3|G8BFD3_CANPC              FDLKRLDSYANNLLDYHVIVDMLPLIAQLYFSKRTSTQVSLSSVQSAILLAIGLQHKNID	887
                                     : .*:  :  .      : *    :    *         :   *  :*:   :*

sp|Q9UZ78|TMCA_PYRAB                ETAKLLNRTELYTMIELRDIVRGLWYYYKHMLKDEEKDIS--------------------	817
tr|Q5JHC6|Q5JHC6_THEKO              ETAKLIDRTEMFTMIEVRDIIRGLWYYYKRLLT---------------------------	811
tr|B7R4L8|B7R4L8_9EURY              ETAKLIDRTETFTMIEVRDIIRGLWYYYKRLLKA--------------------------	811
tr|A0A804QQK4|A0A804QQK4_MAIZE      TVKEELGIEREQVLSNFIKTMKKLYGYLHNIAGKKIETTLPRLKEIE-------------	925
sp|Q9M2Q4|NT102_ARATH               SIERQMQLERGQIHSLLLKVARELYKYLNGVAGKEIKSALPRLKERE-------------	925
sp|Q9XIK4|NT101_ARATH               SIERQMQLERGQIYSLLLKVGKKLYKYLNGIATKELESTLPRLKDRV-------------	929
sp|Q55EJ3|NAT10_DICDI               NLIGELNLASNQVLSLFNQTMRKINTELKQKQEKFIQDSMPKLSIVAPRTGQFKSFKGDI	952
sp|Q9W3C1|NAT10_DROME               ALGLELNMPGNQLLAKFFDAMKRCNQCFRSVLEEHIEGGMLREADLSK------------	897
sp|O01757|NAT10_CAEEL               DISKELDLPMNQIFALLTKAIRRIGDWFDEVCETAVRENLDKEAEASAANK----P--TS	938
tr|Q6PFQ5|Q6PFQ5_DANRE              ELEKEIGLPSSQLMGLFNRIIRKVVQFFNTLQERAVEAEMVATKDIG-------------	927
tr|A0A8J0SC30|A0A8J0SC30_XENTR      QLEKEIELPSSQLMGLFNRIIRKIVQLFNRIQERAVEEEMATTKEVV-------------	925
sp|Q9H0A0|NAT10_HUMAN               QLEKEIELPSGQLMGLFNRIIRKVVKLFNEVQEKAIEEQMVAAKDVV-------------	925
sp|Q8K224|NAT10_MOUSE               QLEKEIELPSGQLMGLFNRIIRKVVKLFNDVQEKAIEEQMVAVKDVV-------------	925
sp|P87115|NAT10_SCHPO               TLEKEFNLPSNQLLAMLVKLSKKIMKCIDEIETKDIEEELGSNKKTE---------SSNS	932
sp|P53914|NAT10_YEAST               TIAKELNLPSNQTIAMFAKIMRKMSQYFRQLLSQSIEETLPNIKDDAIAEM----DGEEI	952
tr|G8BFD3|G8BFD3_CANPC              QIANELNLATNQAMAMFAKIIRKFSTYFRNVLTRAIEEELPELEDEQIKEI----EGDVM	943
                                         :          .    :

sp|Q9UZ78|TMCA_PYRAB                ------------------------------------------------------------	817
tr|Q5JHC6|Q5JHC6_THEKO              ------------------------------------------------------------	811
tr|B7R4L8|B7R4L8_9EURY              ------------------------------------------------------------	811
tr|A0A804QQK4|A0A804QQK4_MAIZE      ----MPPLSKSMDEDLAEAAKEVEEQRRAANEAPV--DPKILQKYAIGD-DNEIDKALQN	978
sp|Q9M2Q4|NT102_ARATH               ----LTAHNVSVDDDIREGAKQVEEQMKKEKIEGL--MDSELQQYVIGD-KE--AEALQH	976
sp|Q9XIK4|NT101_ARATH               ----LEPHKVSVDEDLREGAKEVEEQMRARIEELL--DPELLDQFAIGD-KE--AEALQK	980
sp|Q55EJ3|NAT10_DICDI               KETDMIPLAEDMESELEKGAEEVVNKLKHQ-----LENDSSLSKYLVKGNDEDWSKALKP	1007
sp|Q9W3C1|NAT10_DROME               -GEELQPLTLSLDKELDQTAQKLSKQQ---RKELKRLKAEQLDEFQIKGTEEDWSKALET	953
sp|O01757|NAT10_CAEEL               SLP-KAVPLANLEDELESAAKEIRARHDRDRKALLAELGNELQKYEIIQDEKELAEAYES	997
tr|Q6PFQ5|Q6PFQ5_DANRE              ----LEPTVQTLNEDLNDAAKEF-DEKHKKDIEKI--KDMNLSEYMIRGDDEEWDQVLKK	980
tr|A0A8J0SC30|A0A8J0SC30_XENTR      ----MEPTLKSLQEDLEEAGKEF-QEKHKQEVEKL--KGINLSEYVIRGDDEEWNEVLKK	978
sp|Q9H0A0|NAT10_HUMAN               ----MEPTMKTLSDDLDEAAKEF-QEKHKKEVGKL--KSMDLSEYIIRGDDEEWNEVLNK	978
sp|Q8K224|NAT10_MOUSE               ----MEPTMKTLSDDLDEAAKEF-QEKHKKEVGKL--KDMDLSQYVIRGDDEEWNEVLSK	978
sp|P87115|NAT10_SCHPO               KLPEFTPLQQSLEEELQEGADEAMLALREKQRELI--NAIDLEKYAIRGNEEDWKAAENQ	990
sp|P53914|NAT10_YEAST               KNYNAAEALDQMEEDLEEAGSEAVQAMREKQKELI--NSLNLDKYAINDNSEEWAESQKS	1010
tr|G8BFD3|G8BFD3_CANPC              EEVDYNEIEQNLHNDLEEEGDEAIAEMREKQRELI--NALNLDKYAIADDG-EWDEKSMS	1000


sp|Q9UZ78|TMCA_PYRAB                ------------------------------------------------------------	817
tr|Q5JHC6|Q5JHC6_THEKO              ------------------------------------------------------------	811
tr|B7R4L8|B7R4L8_9EURY              ------------------------------------------------------------	811
tr|A0A804QQK4|A0A804QQK4_MAIZE      ----TKVSASGIISVKSNRTKTDKKDK---HKE--TGKSKRKGTDGGRSELKKKRS----	1025
sp|Q9M2Q4|NT102_ARATH               ----SKISSSGIISVKSTKSEN----E---NGF--DKSTKKRSSDKRSSSSSKSKSSKKR	1023
sp|Q9XIK4|NT101_ARATH               ----SKISSSGLISIESTKTDNKKEKP---SGF--DKSAKKRGNDKHSSTSNKKRRA---	1028
sp|Q55EJ3|NAT10_DICDI               GS----IPN--SITIKRKSDENE---ETDKKEN--NKKSKTKNNNN---NNNNNKKVNNQ	1053
sp|Q9W3C1|NAT10_DROME               ---NGTGGGSGLLSVKSGVKRLDGPIETREDGDLAAPLSKKKKKNNPKQRRSQGKSLI--	1008
sp|O01757|NAT10_CAEEL               VNMKYANK---LVSVKSKRTAIQAAIPDAKDPANKNAK--KKKRF--SSGGR-------R	1043
tr|Q6PFQ5|Q6PFQ5_DANRE              -----AGHT-AIVSIKGDKKRKL---EGTNQKEQREGK--KMKKI--KDKRG---KFGKK	1024
tr|A0A8J0SC30|A0A8J0SC30_XENTR      -----TGQNASVISMKSDKKRKL---EQPEKEGFKQKK--NLKKN--KEKKQ---KFAK-	1022
sp|Q9H0A0|NAT10_HUMAN               -----AGPNASIISLKSDKKRKL---EAKQEPKQSK----KLKNR--ETKNKKDMKLKRK	1024
sp|Q8K224|NAT10_MOUSE               -----AGQNASIVSLKSDKKRKL---ETKQEPKQSK----KLKKR--DN-NRKDMKLKRK	1023
sp|P87115|NAT10_SCHPO               IQK-TNGKGARVVSIKGEKRKNN---SLDAS----DKK--TKEKP--SSK----KKFRK-	1033
sp|P53914|NAT10_YEAST               LE--IAAKAKGVVSLKTGKKRTT---EKAEDIYRQEMK--AMKKP--RKS----KKAAN-	1056
tr|G8BFD3|G8BFD3_CANPC              KAAKGKSNGTGVVAVKKGKRKSG---ESASDIYEQEMK--AVKKN--KKG----KK----	1045


sp|Q9UZ78|TMCA_PYRAB                -----	817
tr|Q5JHC6|Q5JHC6_THEKO              -----	811
tr|B7R4L8|B7R4L8_9EURY              -----	811
tr|A0A804QQK4|A0A804QQK4_MAIZE      -----	1025
sp|Q9M2Q4|NT102_ARATH               KSLKE	1028
sp|Q9XIK4|NT101_ARATH               -----	1028
sp|Q55EJ3|NAT10_DICDI               KSKK-	1057
sp|Q9W3C1|NAT10_DROME               -----	1008
sp|O01757|NAT10_CAEEL               -----	1043
tr|Q6PFQ5|Q6PFQ5_DANRE              T----	1025
tr|A0A8J0SC30|A0A8J0SC30_XENTR      -----	1022
sp|Q9H0A0|NAT10_HUMAN               K----	1025
sp|Q8K224|NAT10_MOUSE               K----	1024
sp|P87115|NAT10_SCHPO               -----	1033
sp|P53914|NAT10_YEAST               -----	1056
tr|G8BFD3|G8BFD3_CANPC              -----	1045
```
